# Supplementary material for: A minimalist approach to stereoselective glycosylation with unprotected donors
Source: Nat Commun. 2017 Oct 27;8:1146. doi: 10.1038/s41467-017-01073-7 (PMC5660076; doi:10.1038/s41467-017-01073-7)
Supplement: Supplementary file 1 — Supplementary Info [file 41467_2017_1073_MOESM1_ESM.pdf]

Supplementary Table 1. Optimize masking effect of boron reagent.

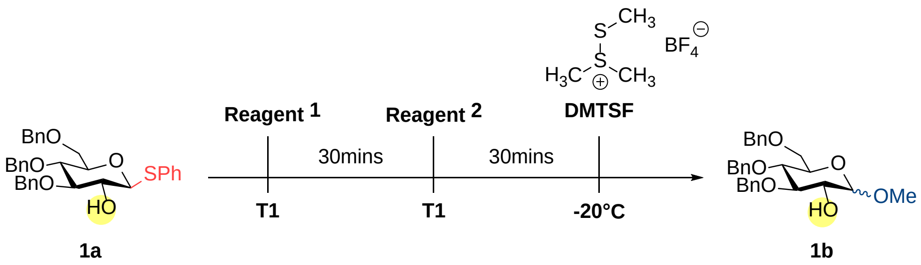

Entry[a]	Reagent 1	T1	Reagent 2	Solvent	Yield (%)[b]	α/β[c]
1	-	-20 ℃	MeOH	CH_2Cl_2	40	2.3/1
2	-	-20 ℃	Et_2BOMe	CH_2Cl_2	<5	2/1
3	Et_2BOTf	-20 ℃	Et_2BOMe[d]	CH_2Cl_2	20	α only
4	MeOH	-20 ℃	Et_2BOTf[e]	CH_2Cl_2	-	-
5	Et_2BOTf	-20 ℃	MeOH	CH_2Cl_2	51	1/1.1
6	Et_2BOTf	+20 ℃	MeOH	CH_2Cl_2	25	2.2/1
7	9-(BBN)OTf	-20 ℃	MeOH	CH_2Cl_2	59	1/1.4
8	Bu_2BOTf	-20 ℃	MeOH	CH_2Cl_2	60	1/1.5
9	$Cyhx_2BOTf$	-20 ℃	MeOH	CH_2Cl_2	63	1:2.1
10	Bu_2BOTf	-20 ℃	MeOH	MeCN	-	-
11	Bu_2BOTf	-20 ℃	MeOH	Toluene	59	1/1.1

[a] Unless otherwise specified, all reactions were carried out with 1 equivalent of **1a**, 3 eq. of TTBP, 1.1 eq. of boron reagent, 1.2 eq. of MeOH and 3 eq. of DMTSF in 2 mL of solvent for 12 hours. [b] isolated yield. [c] determined by ^{1}H-NMR integration. [d] 7 eq. was added. [e] 2.2 eq was added.

Supplementary Discussions

Optimize masking effect of boron reagent.

The feasibility to use dialkyl boron as masking reagent was studied with donor glucosyl **1a** as model substrate. In the absence of boron, addition of DMTSF into mixture of **1a** and MeOH gave desired product **1b** in only 40% yield with some α-selectivity (Supplementary Table 1, entry 1), and a complex reaction mixture based on TLC visualization. Mixture of **1b** and diethylmethoxyborane delivered only trace amount of **1b** (entry 2). Surprisingly, pre-masking **1a** with Et_2BOTf, followed by addition of excess amount of Et_2BOMe increased the yield of **1b** to 20% and α-only stereo-configuration (entry 3). In addition, the only other product isolated was the hydrolytic compound. Unfortunately, mixing of both donor and acceptor, followed by addition of Et_2BOTf gave only the hydrolytic product (entry 4). Only when Et_2BOTf was added dropwise to a solution of **1a** and TTBP prior to addition of MeOH and DMTSF thereafter that we managed to isolate product **1b** in a reasonable yield (entry 5). From these experimental results, the proposed tetravalent boron intermediate is unlikely to form under the reaction condition. Attempt to carry out the reaction at ambient temperature proved unfruitful as the mixture turned brown and darkened quickly, resulting in complicated mixture (entry 6). Hence, we

focused our effort to improve the β-selectivity in the following trials. Increasing the steric bulk of the dialkyl substituents on boron does favor the formation of β-anomer, albeit marginally (entries 7-9). Reaction under acetonitrile was unable to activate the donor (entry 10) whereas toluene exhibited less preference for β-**1b** (entry 11). From these experimental results, the order of addition of reagents was critical for consistent formation of desired product and the following protocol was established: An equimolar amount of dialkyl boron triflate was added to the glycosyl donor **1a**, followed by addition of thiophilic activator and glycosyl acceptor. Further control experiments were discussed in the main report.

Low temperature NMR experiments

The following ^{11}B-NMR samples at –40 °C were collected for standard references: (A) 0.1mL of $BF_3.OEt_2$ (0.8 mmol) dissolved in 0.4ml CD_2Cl_2 showed a singlet, calibrated as 0 ppm. (B) 0.1mL of Et_2BOMe (0.7 mmol) dissolved in 0.4ml CD_2Cl_2 showed a singlet at 54.8 ppm (C) 0.1mL of solution of Bu_2BOTf (0.1 mmol, 1M in CH_2Cl_2) diluted in 0.4ml CD_2Cl_2 showed a broad singlet at 64.0 ppm. See Supplementary Figure 93.

The following substrates were chosen for low temperature NMR experiments:

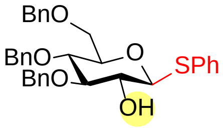

1a

54mg (0.1 mmol) of donor **1a** and 75mg (0.3 mmol) of TTBP were dried under high vacuum and dissolved in 0.4 mL of CD_2Cl_2 and transferred to a quartz 5mm NMR-tube (<0.01 ppm Boron) under an argon atmosphere. The sample was inserted into the NMR spectrometer (400MHz), and the system was tuned, matched, locked and shimmed at –40 °C. ^1H-NMR was recorded to affirm the purity of sample. The sample was briefly lifted up and 0.11mL solution of Bu_2BOTf (0.11mmol, 1M in CH_2Cl_2) was injected on the spot and the tube was quickly lowered into the NMR probe. After 1min, the NMR tube was lifted up again, shaken and quickly lowered into the NMR probe. ^1H-NMR and ^{11}B-NMR was recorded after 1min, 5mins, 10mins, 30mins. 1D-TOCSY selective irradiation and 2D-COSY were performed to assign the observed chemical shifts.

Upon addition of Bu_2BOTf, the proton of OH at C-2 disappeared and H-2 was shifted downfield to 4.20 ppm from its previous position at 3.56 ppm (Δ=0.64 ppm), whereas other protons remained relatively unchanged. The displacement of OH proton with boron complex exerted a deshielding effect on its immediate vicinal proton. This suggested the boron complex having an electron-withdrawing effect similar to acetyl, benzoyl, etc…This intermediate was found to be stable after all NMR experiments were collected (1D-TOCY, COSY, ^{11}B) and only showed decomposition at or above -10 °C. Interestingly, ^{11}B-NMR showed a broad signal with peak at about -3.0 ppm. This value was typical of a tetravalent boron species. In a second run, only 0.5 equivalent of Bu_2BOTf was added and we

observed a mixture of boron-masked and original **2.2a.** This excluded the formation of a dimer of the type RO-B(Bu_2)-OR, which led us to propose the observed intermediate to be RO-B(Bu_2)-OTf. It is noteworthy that there is usually excess amount of triflate ion in our reaction condition (e.g. from Bu_2BOTf, Tf_2O, AgOTf…). In a third run, one equivalent of BuOH was added 5 mins after introduction of one equivalent of Bu_2BOTf. We observed no change in chemical shift of boron-masked **2.2a** after 30 mins at –40 °C. However, excess amount of BuOH resulted in mixture of boron-masked and original **2.2a.** We concluded that the exchange process between different alcohol species was slow enough at low temperature to warrant the desired masking effect. See Supplementary Figure 94-96.

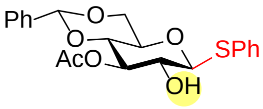

29a

^{1}H and ^{11}B NMR of donor **29a** was obtained following the protocol for **1a**. We observed the proton of OH at C-2 disappeared and H-2 was shifted downfield to 4.30 ppm from its previous position at 3.56 ppm (Δ=0.74 ppm). In addition, H-3 was shifted appreciably downfield to 5.48 ppm from its previous position at 5.26 ppm (Δ=0.22 ppm). ^{11}B-NMR showed a broad signal with peak at about -20 ppm. The change in chemical shifts of H-2, H-3 and ^{11}B can thus be attributed to the electron-withdrawing effect of boron complex and possible attenuating effect of neighboring acetyl group. See Supplementary Figure 97-99.

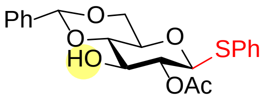

30a

^{1}H and ^{11}B NMR of donor **30a** was obtained following the protocol for **1a**. We observed the proton of OH at C-3 disappeared and H-3 was shifted downfield to 4.50 ppm from its previous position at 3.83 ppm (Δ=0.67 ppm). In addition, H-2 was shifted appreciably downfield to 5.10 ppm from its previous position at 4.94 ppm (Δ=0.16 ppm). ^{11}B-NMR showed a broad signal with peak at about -21 ppm. The change in chemical shifts of H-2, H-3 and ^{11}B can thus be attributed to the electron-withdrawing effect of boron complex and possible attenuating effect of neighboring acetyl group. See Supplementary Figure 100-102.

Supplementary Methods

Method A: Activation with DMTSF

To a magnetically stirred solution of phenyl 3,4,6-tri-*O*-benzyl-β-D-thioglucopyranoside **1a** (54.2mg, 0.1 mmol) and 2,4,6-tri-tert-butylpyrimidine (74mg, 0.3mmol) in anhydrous CH_2Cl_2 (2 mL) at -20 °C was added dropwise solution of dibutylboryl trifluoromethanesulfonate (110μl, 0.11

mmol, 1M in CH_2Cl_2). Temperature was lowered to -40 °C after 30 mins and n-butanol (110 µl, 0.12 mmol) was added. After 5 mins, dimethyl(methylthio)sulfonium tetrafluoroborate (DMTSF, 58.8mg, 0.3 mmol) was added and reaction mixture was stirred for 12 hours. Triethylamine (27µl, 0.2 mmol) was added to quench the reaction. Reaction mixture was dissolved in CH_2Cl_2 (10 mL), washed with water (20 mL), brine (20 mL). Organic eluents were filtered off anhydrous sodium sulfate and the residue recovered from rotavap, after which flash chromatography on silica gel afforded **Butyl 3,4,6-tri-O-benzyl-D-glucopyranoside 1e** (37mg, 73% yield) as a colorless oil. The anomeric selectivity was determined by ^{1}H-NMR integration.

Method B: Activation with NIS/TMSOTf

To a magnetically stirred solution of phenyl 3,4,6-tri-*O*-benzyl-β-D-thioglucopyranoside **1a** (54.2mg, 0.1 mmol) and 2,4,6-tri-tert-butylpyrimidine (74mg, 0.3mmol) in anhydrous CH_2Cl_2 (2 mL) at -20 °C was added dropwise solution of dibutylboryl trifluoromethanesulfonate (110µl, 0.11 mmol, 1M in CH_2Cl_2). Temperature was lowered to -40 °C after 30 mins and n-butanol (110 µl, 0.12 mmol) was added. After 5 mins, N-Iodosuccinimide (27mg, 0.12 mmol) and trimethylsilyl trifluoromethanesulfonate (3.6µl, 0.02 mmol) was added and reaction mixture was stirred for 12 hours. Triethylamine (27µl, 0.2 mmol) was added to quench the reaction. Reaction mixture was dissolved in CH_2Cl_2 (10 mL), washed with water (20 mL), brine (20 mL). Organic eluents were filtered off anhydrous sodium sulfate and the residue recovered from rotavap, after which flash chromatography on silica gel afforded **1e** (45mg, 89% yield) as a colorless oil. The anomeric selectivity was determined by ^{1}H-NMR integration.

Method C: Activation with AgOTf/*p*-NO_2PhSCl

To a magnetically stirred solution of phenyl 3,4,6-tri-*O*-benzyl-β-D-thioglucopyranoside **1a** (54.2mg, 0.1 mmol), AgOTf (77mg, 0.3 mmol) and 2,4,6-tri-tert-butylpyrimidine (74mg, 0.3mmol) in anhydrous CH_2Cl_2 (2 mL) at -20 °C was added dropwise solution of dibutylboryl trifluoromethanesulfonate (110µl, 0.11 mmol, 1M in CH_2Cl_2). Temperature was lowered to -60 °C after 30 mins and p-nitrobenzenesulfenyl chloride (26.5mg, 0.12 mmol) was added. After 5 mins, n-butanol (110 µl, 0.12 mmol) was added at -78 °C and reaction mixture was stirred for 12 hours. Triethylamine (27µl, 0.2 mmol) was added to quench the reaction. Inorganic materials was filtered, washed with CH_2Cl_2 (20ml) and organic eluents were washed with water (20 mL), brine (20 mL). Organic eluents were filtered off anhydrous sodium sulfate and the residue recovered from rotavap, after which flash chromatography on silica gel afforded **1e** (43mg, 85% yield) as a colorless oil. The anomeric selectivity was determined by ^{1}H-NMR integration.

Method D: Activation with Ph_2SO/Tf_2O

To a magnetically stirred solution of phenyl 3,4,6-tri-*O*-benzyl-β-D-thioglucopyranoside **1a** (54.2mg, 0.1 mmol), diphenyl sulfoxide (26mg, 0.13 mmol) and 2,4,6-tri-tert-butylpyrimidine (74mg, 0.3mmol) in anhydrous CH_2Cl_2 (2 mL) at -20 °C was added dropwise solution of dibutylboryl

trifluoromethanesulfonate (110µl, 0.11 mmol, 1M in CH_2Cl_2). Temperature was lowered to -60 ˚C after 30 mins and trifluoromethanesulfonic anhydride (150µl, 0.15 mmol, 1M in CH_2Cl_2) was added. After 5 mins, n-butanol (110 µl, 0.12 mmol) was added at -78 ˚C and reaction mixture was stirred for 12 hours. Triethylamine (27µl, 0.2 mmol) was added to quench the reaction. Reaction mixture was dissolved in CH_2Cl_2 (10 mL), washed with water (20 mL), brine (20 mL). Organic eluents were filtered off anhydrous sodium sulfate and the residue recovered from rotavap, after which flash chromatography on silica gel afforded **1e** (44mg, 86% yield) as a colorless oil. The anomeric selectivity was determined by ^{1}H-NMR integration.

Method E: One-pot protocol to synthesize 17ai

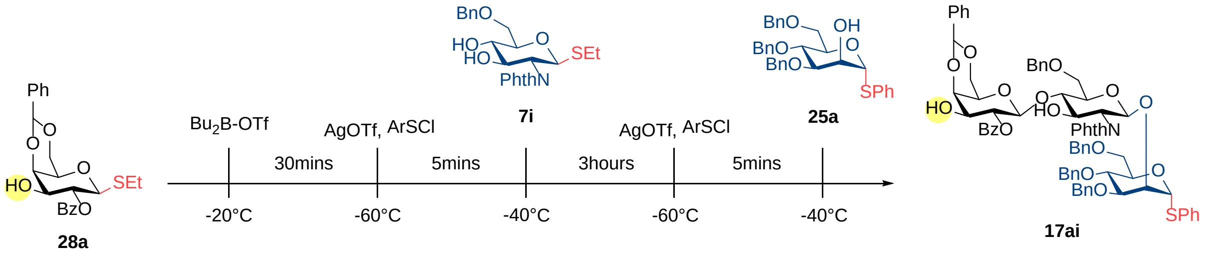

To a magnetically stirred solution of **28a** (21mg, 0.05 mmol), AgOTf (38mg, 0.15 mmol) and 2,4,6-tri-tert-butylpyrimidine (74mg, 0.3mmol) in anhydrous CH_2Cl_2 (2 mL) at -20 ˚C was added dropwise solution of dibutylboryl trifluoromethanesulfonate (55µl, 0.055 mmol, 1M in CH_2Cl_2). Temperature was lowered to -60 ˚C after 30 mins and *p*-nitrobenzenesulfenyl chloride (13.3mg, 0.06 mmol) was added. After 5 mins, compound **7i** (26mg, 0.06 mmol) was added at -40 ˚C and reaction mixture was stirred for 3 hours. Another portion of AgOTf (19mg, 0.075mg) and *p*-NO_2PhSCl (13.3mg, 0.06 mmol) was sequentially added at -60 ˚C. After 5 mins, compound **25a** (54mg, 0.1 mmol) was added at -40 ˚C and the reaction was stirred for 3 hours. Triethylamine (27µl, 0.2 mmol) was added to quench the reaction. Inorganic materials was filtered, washed with CH_2Cl_2 (20ml) and organic eluents were washed with water (20 mL), brine (20 mL). Organic eluents were filtered off anhydrous sodium sulfate and the residue recovered from rotavap, after which flash chromatography on silica gel afforded **17ai** (30mg, 50% yield from **28a**) as a colorless oil. Trace amount of **18ai** was also detected.

Methyl 3,4,6-tri-*O*-benzyl-D-glucopyranoside 1b (Method A)

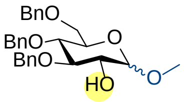

1b

(27mg, 60% yield) as a white solid (m.p. 79-82˚C). R_f 0.2 (Hexane/EtOAc 3:1).

^{1}H-NMR (500 MHz, $CDCl_3$, 1α/1.5β): δ (ppm) 7.37-7.14 (m, 30H, both isomers), 4.92-4.79 (m, 10H, both isomers), 4.64-4.49 (m, 9H, both isomers), 4.18 (d, 1H, J = 7.6 Hz), 3.77-3.41 (m, 28H, both isomers), 2.32 (s, 1H), 2.12 (d, 1H, J = 7.9 Hz). See Supplementary Figure 1.

^{13}C-NMR (125 MHz, $CDCl_3$): δ (ppm) 138.8-138.1, 128.7-127.9, 103.8, 99.6, 84.7, 83.5, 77.8, 77.7, 75.5, 75.4, 75.3, 75.2, 75.1, 74.8, 73.7, 73.1, 70.6, 69.0, 68.7, 57.3, 55.4. See Supplementary Figure 2.

IR (Nujol): ν (cm^{-1}) 3018, 2399, 1521, 1419, 1215, 1051, 927, 760, 669.

HRMS (ESI): m/z calcd for $C_{28}H_{32}O_6$ $[M+H]^+$, 465.2277, found: 465.2278.

Ethyl 3,4,6-tri-*O*-benzyl-D-glucopyranoside 1c (Method A)

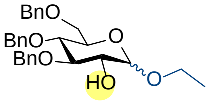

1c

(29mg, 62% yield) as a white solid (m.p. 82-83 ˚C). R_f 0.4 (CH_2Cl_2/EtOAc 10:1).

^{1}H-NMR (500 MHz, $CDCl_3$, 1α/1.8β): δ (ppm) 7.38-7.15 (m, 25H, aromatic), 4.95-4.9 (m, 2H, both isomers), 4.85-4.81 (m, 3H, both isomers), 4.64-4.48 (m, 5H, both isomers), 4.25 (d, 1H, J = 7.6 Hz), 4.02-3.94 (m, 1H), 3.82-3.45 (m, 13H, both isomers), 2.31 (s, 1H), 2.12 (d, 1H, J = 1.8 Hz), 1.27-1.22 (m, 5H, both isomers). See Supplementary Figure 3.

^{13}C-NMR (125 MHz, $CDCl_3$): δ (ppm) 138.9-138.1, 128.6-127.8, 102.7, 98.3, 84.7, 83.7, 77.8, 77.6, 75.5, 75.4, 75.3, 75.2, 74.8, 73.7, 73.2, 70.7, 67.1, 67.7, 65.5, 63.7, 15.3, 15.2. See Supplementary Figure 4.

IR (Nujol): ν (cm^{-1}) 3020, 2399, 1519, 1423, 1216, 1051, 930, 758, 669.

HRMS (ESI): m/z calcd for $C_{29}H_{34}O_6$ $[M+H]^+$, 479.2434, found: 479.2438.

Isopropyl 3,4,6-tri-*O*-benzyl-D-glucopyranoside 1d (Method A)

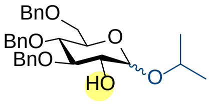

1d

(28mg, 59% yield) as a colorless oil. R_f 0.5 (CH_2Cl_2/EtOAc 10:1).

^{1}H-NMR (500 MHz, $CDCl_3$, 1α/2.5β): δ (ppm) 7.38-7.14 (m, 22H, aromatic), 4.99-4.93 (m, 2H, both isomers), 4.84-4.81 (m, 3H, both isomers), 4.64-4.47 (m, 4H, both isomers), 4.31 (d, 1H, J = 7.7 Hz), 4.02-3.46 (m, 10H, both isomers), 2.31 (s, 1H), 2.01 (d, 1H, J = 2.3 Hz), 1.28-1.17 (m, 10H, both isomers). See Supplementary Figure 5.

^{13}C-NMR (125 MHz, $CDCl_3$): δ (ppm) 138.8-138.0, 128.4-127.6, 101.2, 96.8, 84.6, 83.7, 77.6, 76.7, 75.3, 75.1, 75.0, 74.7, 73.5, 73.4, 72.9, 71.9, 70.5, 70.3, 69.1, 68.5, 23.5, 23.3, 22.0, 21.6. See Supplementary Figure 6.

IR (Nujol): ν (cm^{-1}) 3015, 2399, 1520, 1420, 1219, 1052, 933, 755, 669.

HRMS (ESI): m/z calcd for $C_{30}H_{36}O_6$ $[M+H]^+$, 493.2590, found: 493.2598.

Butyl 3,4,6-tri-*O*-benzyl-β-D-glucopyranoside 1e (Method A, B, C, D)

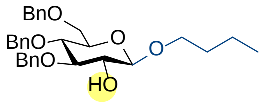

1e

(46mg, 86% yield) as a colorless oil. R_f 0.3 (Hexane/EtOAc 4:1); $[\alpha]_D^{20}$+58.2 (c 0.1, $CHCl_3$).

^{1}H-NMR (500 MHz, $CDCl_3$): δ (ppm) 7.37-7.16 (m, 15H, aromatic), 4.93 (d, 1H, *J* = 11.3 Hz), 4.83 (d, 1H, *J* = 11.2 Hz), 4.62-4.52 (m, 3H), 4.24 (d, 1H, *J* = 7.6 Hz), 3.94-3.90 (m, 1H), 3.74 (dd, 1H, *J* = 1.9 Hz, *J* = 10.8 Hz), 3.69 (dd, 1H, *J* = 4.7 Hz, *J* = 10.8 Hz), 3.61-3.53 (m, 5H), 2.31 (s, 1H), 1.62-1.60 (m, 2H), 1.40-1.38 (m, 2H), 0.93 (t, 3H, *J* = 7.0 Hz). See Supplementary Figure 7.

^{13}C-NMR (125 MHz, $CDCl_3$): δ (ppm) 138.8-127.81, 102.9, 84.7, 77.8, 75.4, 75.3, 75.2, 74.9, 73.7, 70.0, 69.1, 31.8, 19.4, 14.1. See Supplementary Figure 8.

IR (Nujol): ν (cm^{-1}) 3684, 3618, 3018, 2974, 2399, 1521, 1423, 1213, 1047, 779, 669.

HRMS (ESI): m/z calcd for $C_{31}H_{38}O_6$ $[M+Na]^+$ 529.2566 , found: 529.2568.

Butyl 3-*O*-benzyl-4,6-*O*-benzylidene-β-D-glucopyranoside 2e (Method D)

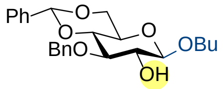

2e

(34mg, 83% yield) as a colorless oil. R_f 0.3 (Hexane/EtOAc 4:1); $[\alpha]_D^{20}$-31.6 (c 0.1, $CHCl_3$).

^{1}H-NMR (500 MHz, $CDCl_3$): δ (ppm) 7.49-7.25 (m, 10H, aromatic), 5.56 (s, 1H), 4.96 (d, 1H, *J* = 11.8 Hz), 4.81 (d, 1H, *J* = 11.7 Hz), 4.39 (d, 1H, *J* = 7.7 Hz), 3.91-3.87 (m, 1H), 3.80 (t, 1H, *J* = 10.3 Hz), 3.72-3.64 (m, 2H), 3.58-3.53 (m, 2H), 3.48-3.41 (m, 1H), 2.38 (s, 1H), 1.64-1.61 (m, 2H), 1.41-1.38 (m, 2H), 0.92 (t, 3H, *J* = 7.4 Hz). See Supplementary Figure 9.

^{13}C-NMR (125 MHz, $CDCl_3$): δ (ppm) 138.4-120.5, 103.3, 101.3, 81.4, 80.2, 74.6, 74.4, 70.2, 68.7, 66.4, 31.6, 19.1, 13.8. See Supplementary Figure 10.

IR (Nujol): ν (cm^{-1}) 3684, 3616, 3018, 2976, 2399, 1519, 1423, 1215, 1070, 927, 771, 669.

HRMS (ESI): m/z calcd for $C_{24}H_{30}O_6$ $[M+H]^+$, 415.2121, found: 415.2123.

Butyl 3-*O*-acetyl-4,6-*O*-para-methoxybenzylidene-D-glucopyranoside 3e (Method D)

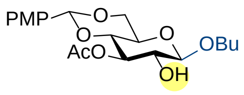

3e

(30mg, 72% yield) as a white solid (m.p. 127-128 ˚C). R_f 0.3 (Hexane/EtOAc 2:1).

^{1}H-NMR (500 MHz, $CDCl_3$, 1α/4.5β): δ (ppm) 7.38-7.35 (m, 2H, aromatic), 6.87-6.86 (m, 2H, aromatic), 5.44 (s, 1H), 5.31 (t, 1H, *J* = 9.7 Hz), 5.21 (t, 1H, *J* = 9.5 Hz), 4.87 (d, 1H, *J* = 3.8 Hz), 4.43 (d, 1H, *J* = 7.7 Hz), 4.32 (dd, 1H, *J* = 4.9 Hz, *J* = 10.5 Hz), 4.25 (dd, 1H, *J* = 4.9 Hz, *J* = 10.3 Hz), 3.91-3.46 (m, 12H, both isomers), 2.6 (s, 1H), 2.11 (s, 4H, both isomers), 1.65-1.59 (m, 2H, both isomers), 1.43-1.35 (m, 2H, both isomers), 0.92 (m, 3H, both isomers). See Supplementary Figure 11.

^{13}C-NMR (125 MHz, $CDCl_3$): δ (ppm) 170.8, 161.1, 129.5, 127.5, 113.6, 103.5, 101.4, 99.0, 78.5, 73.5, 73.4, 72.5, 71.8, 70.3, 68.8, 68.6, 66.5, 55.3, 3.6, 31.5, 20.9, 19.1, 13.8. See Supplementary Figure 12.

IR (Nujol): ν (cm^{-1}) 3431, 3018, 2962, 2935, 2875, 2399, 1741, 1616, 1517, 1369, 1215, 756, 669.

HRMS (ESI): m/z calcd for $C_{20}H_{28}O_8$ $[M+Na]^+$, 419.1682, found: 419.1692.

Butyl 2,4,6-tri-*O*-benzyl-β-D-glucopyranoside 4e (Method D)

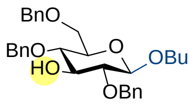

4e

(44mg, 88% yield) as a colorless oil. R_f 0.3 (Hexane/EtOAc 4:1); $[\alpha]_D^{20}$+66.0 (c 0.5, $CHCl_3$).

^{1}H-NMR (500 MHz, $CDCl_3$): δ (ppm) 7.35-7.22 (m, 15H, aromatic), 4.96 (d, 1H, *J* = 11.5 Hz), 4.83 (d, 1H, *J* = 11.1 Hz), 4.67-4.54 (m, 4H), 4.35 (d, 1H, *J* = 7.8 Hz), 3.98-3.93 (m, 1H), 3.76-3.65 (m, 3H), 3.55-3.44 (m, 3H), 3.25 (t, 1H, *J* = 8.1 Hz), 2.43 (s, 1H), 1.66-1.58 (m, 2H), 1.44-1.41 (m, 2H), 0.93 (t, 3H, *J* = 7.4 Hz). See Supplementary Figure 13.

^{13}C-NMR (125 MHz, $CDCl_3$): δ (ppm) 138.4-127.6, 103.2, 81.2, 76.8, 76.7, 74.8, 74.5, 74.3, 73.4, 69.7, 69.1, 31.8, 19.3, 13.8. See Supplementary Figure 14.

IR (Nujol): ν (cm^{-1}) 3421, 3018, 2399, 1627, 1215, 1062, 767, 669.

HRMS (ESI): m/z calcd for $C_{31}H_{38}O_6$ $[M+Na]^+$, 529.2566, found: 529.2564.

Butyl 2-*O*-benzyl-4,6-*O*-benzylidene-β-D-glucopyranoside 5e (Method D)

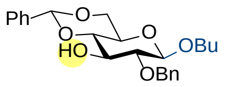

5e

(36mg, 87% yield) as a colorless oil. R_f 0.3 (Hexane/EtOAc 4:1); $[\alpha]_D^{20}$ +9.6 (c 0.1, $CHCl_3$).

^{1}H-NMR (500 MHz, $CDCl_3$): δ (ppm) 7.49-7.25 (m, 10H, aromatic), 5.52 (s, 1H), 4.95 (d, 1H, *J* = 11.4 Hz), 4.72 (d, 1H, *J* = 11.4 Hz), 4.51 (d, 1H, *J* = 7.7 Hz), 4.33 (dd, 1H, *J* = 4.9 Hz, *J* = 10.5 Hz), 3.96-3.91 (m, 1H), 3.85-3.75 (m, 2H), 3.58-3.52 (m, 2H), 3.43-3.42 (m, 1H), 3.34 (t, 1H, J = 8.0 Hz), 2.43 (s, 1H), 1.67-1.63 (m, 2H), 1.44-1.42 (m, 2H), 0.94 (t, 3H, *J* = 7.4 Hz). See Supplementary Figure 15.

^{13}C-NMR (125 MHz, $CDCl_3$): δ (ppm) 138.2-126.3, 103.8, 101.7, 81.8, 80.4, 74.7, 73.1, 70.2, 68.7, 66.1, 31.8, 19.2, 13.8. See Supplementary Figure 16.

IR (Nujol): ν (cm^{-1}) 3419, 3018, 2399, 1635, 1215, 927, 771, 669.

HRMS (ESI): m/z calcd for $C_{24}H_{30}O_6$ $[M+H]^+$, 415.2121, found: 415.2120.

Butyl 2-*O*-acetyl-4,6-*O*-para-methoxybenzylidene-β-D-glucopyranoside 6e (Method D)

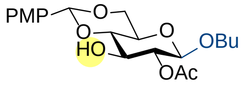

6e

(32mg, 81% yield) as a white solid (m.p. 129-132 °C). R_f 0.3 (Hexane/EtOAc 2:1).

^{1}H-NMR (500 MHz, $CDCl_3$, 1α/6β): δ (ppm) 7.54-7.39 (m, 4H, aromatic), 5.76 (s, 1H), 5.58 (s, 1H), 4.99 (d, 1H, *J* = 5.3 Hz), 4.90 (d, 1H, *J* = 3.7 Hz), 4.83 (dd, 1H, *J* = 3.7 Hz, *J* = 9.7 Hz), 4.74 (dd, 1H, *J* = 3.7 Hz, *J* = 9.9 Hz), 4.33 (dd, 1H, *J* = 4.7 Hz, *J* = 10.1 Hz), 4.23-4.19 (m, 1H), 4.15-4.09 (m, 1H), 3.94-3.68 (m, 3H, both isomers), 3.59 (t, 1H, *J* = 9.3 Hz), 3.43-3.37 (m, 6H, both isomers), 3.05 (d, 1H, *J* = 3.3 Hz), 2.74 (dd, 1H, *J* = 6.3 Hz, *J* = 8.2 Hz), 2.45 (d, 1H, *J* = 2.9 Hz), 2.19 (s, 3H), 2.16 (s, 3H), 1.68-1.62 (m, 2H, both isomers), 1.44-1.33 (m, 2H, both isomers), 0.92 (t, 3H, *J* = 7.5 Hz, both isomers). See Supplementary Figure 17.

^{13}C-NMR (125 MHz, $CDCl_3$): δ (ppm) 171.0, 160.3, 129.7, 127.7, 113.7, 103.7, 101.6, 99.2, 78.7, 73.7, 73.6, 72.6, 72.0, 70.5, 69.0, 68.8, 66.7, 55.4, 31.8, 31.7, 21.1, 19.2, 13.9. See Supplementary Figure 18.

IR (Nujol): ν (cm^{-1}) 3419, 3018, 2399, 2088, 1635, 1215, 1058, 771, 669.

HRMS (ESI): m/z calcd for $C_{20}H_{28}O_8$ $[M+Na]^+$, 419.1682, found: 419.1688.

Butyl 2,3,6-tri-*O*-benzyl-β-D-glucopyranoside 7e (Method D)

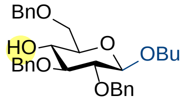

7e

(42mg, 86% yield) as a colorless oil. R_f 0.3 (Hexane/EtOAc 3:1); $[\alpha]_D^{20}$ -78.2 (c 0.5, $CHCl_3$).

^{1}H-NMR (500 MHz, $CDCl_3$): δ (ppm) 7.36-7.24 (m, 15H, aromatic), 4.93 (t, 2H, *J* = 10.4 Hz), 4.74-4.55 (m, 4H), 4.40 (d, 1H, *J* = 7.3 Hz), 3.96-3.92 (m, 1H), 3.77 (dd, 1H, *J* = 3.9 Hz, *J* = 10.4 Hz), 3.70 (dd, 1H, *J* = 5.5 Hz, *J* = 10.4 Hz), 3.59-3.46 (m, 5H), 2.52 (s, 1H), 1.66-1.62 (m, 2H), 1.58-1.43 (m, 2H), 0.93 (t, 3H, *J* = 7.4 Hz). See Supplementary Figure 19.

^{13}C-NMR (125 MHz, $CDCl_3$): δ (ppm) 138.6-127.7, 103.7, 84.1, 81.7, 75.3, 74.7, 74.0, 73.6, 71.7, 70.4, 69.8, 31.8, 19.3, 13.8. See Supplementary Figure 20.

IR (Nujol): ν (cm^{-1}) 3495, 2958, 2088, 1635, 1454, 1060, 732, 588, 526.

HRMS (ESI): m/z calcd for $C_{31}H_{38}O_6$ $[M+Na]^+$, 529.2566, found: 529.2561.

Butyl 2,3-di-*O*-benzoyl-6-*O*-benzyl-β-D-glucopyranoside 8e (Method D)

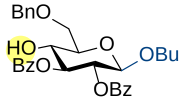

8e

(43mg, 85% yield) as a colorless oil. R_f 0.3 (Hexane/EtOAc 3:1); $[\alpha]_D^{20}$+29.4 (c 0.5, $CHCl_3$).

^{1}H-NMR (500 MHz, $CDCl_3$): δ (ppm) 7.98-7.94 (m, 4H, aromatic), 7.51-7.25 (m, 11H, aromatic), 5.46-5.38 (m, 2H), 4.67-4.59 (m, 3H), 3.96-3.85 (m, 4H), 3.71-3.67 (m, 1H), 3.52-3.48 (m, 1H), 3.26 (d, 1H, *J* = 3.2 Hz), 1.48-1.47 (m, 2H), 1.24-1.21 (m, 2H), 0.74 (t, 3H, *J* = 7.4 Hz). See Supplementary Figure 21.

^{13}C-NMR (125 MHz, $CDCl_3$): δ (ppm) 167.2, 165.2, 137.7-127.7, 101.1, 74.6, 73.8, 71.6, 71.2, 70.2, 69.8, 31.4, 18.9, 13.6. See Supplementary Figure 22.

IR (Nujol): ν (cm^{-1}) 3410, 3018, 2399, 2088, 1635, 1419, 1215, 927, 756, 669.

HRMS (ESI): m/z calcd for $C_{31}H_{34}O_8$ $[M+Na]^+$, 557.2151, found: 557.2170.

1,6-Anhydro-2,3-di-*O*-*tert*-butyldimethylsilyl-β-D-glucopyranose 9e (Method D)

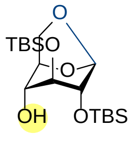

9e

(35mg, 91% yield) as a white solid (m.p. 48-50˚C). R_f 0.4 (Hexane/EtOAc 9:1); $[\alpha]_D^{20}$-30.2 (c 0.5, $CHCl_3$).

^{1}H-NMR (500 MHz, $CDCl_3$): δ (ppm) 5.25 (s, 1H), 4.49-4.47 (m, 1H), 4.28 (d, 1H, *J* = 6.9 Hz), 3.74-3.71 (m, 2H), 3.47 (d, 1H, *J* = 1.6 Hz), 3.41 (dd, 1H, *J* = 1.7 Hz, *J* = 12.3 Hz), 3.01 (d, 1H, *J* = 12.3 Hz), 0.91-0.90 (m, 18H), 0.10-0.09 (m, 12H). See Supplementary Figure 23.

^{13}C-NMR (125 MHz, $CDCl_3$): δ (ppm) 101.9, 76.2, 73.6, 71.3, 71.1, 64.7, 25.7, 25.6, 17.9, 17.8, -4.7, -4.8, -4.9, -5.0. See Supplementary Figure 24.

IR (Nujol): ν (cm^{-1}) 3433, 3018, 2399, 2088, 1643, 1215, 1099, 835, 750, 669.

HRMS (ESI): m/z calcd for $C_{18}H_{38}O_5Si_2$ $[M+Na]^+$, 413.2155, found: 413.2160.

Butyl 2,3,4-tri-*O*-benzyl-α-D-glucopyranoside 10e_alpha (Method D)

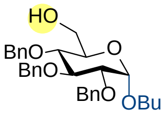

10e_alpha

10e_alpha was successfully separated from the mixture of **10e_alpha** and **10e_beta** (14mg, 27% yield) as a colorless oil. R_f 0.3 (Hexane/EtOAc 4:1); $[\alpha]_D^{20}$+82.1 (c 0.5, $CHCl_3$).

^{1}H-NMR (400 MHz, $CDCl_3$): δ (ppm) 7.39-7.28 (m, 15H, aromatic), 5.04-4.79 (m, 4H), 4.72 (d, 1H, *J* = 3.6 Hz), 4.69-4.65 (m, 2H), 4.04 (t, 1H, *J* = 9.2 Hz), 3.79-3.52 (m, 7H), 1.66-1.58 (m, 3H), 1.44-1.40 (m, 2H), 0.95 (t, 3H, *J* = 7.4 Hz). See Supplementary Figure 25.

^{13}C-NMR (100 MHz, $CDCl_3$): δ (ppm) 138.8-127.6, 96.8, 81.9, 80.3, 77.5, 77.2, 75.6, 75.1, 73.2, 70.7, 67.9, 61.9, 31.5, 19.4, 13.8. See Supplementary Figure 26.

IR (Nujol): ν (cm^{-1}) 3441, 3018, 2399, 2065, 1635, 1215, 756, 669.

HRMS (ESI): m/z calcd for $C_{31}H_{39}O_6$ $[M+H]^+$, 507.2747, found: 507.2755.

Butyl 2,3,4-tri-*O*-benzyl-β-D-glucopyranoside 10e_beta (Method D)

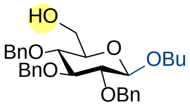

10e_beta

10e_beta was successfully separated from the mixture of **10e_alpha** and **10e_beta** (27mg, 54% yield) as a colorless oil. R_f 0.3 (Hexane/EtOAc 4:1); $[\alpha]_D^{20}$+14.5 (c 0.2, $CHCl_3$).

^{1}H-NMR (400 MHz, $CDCl_3$) δ (ppm) 7.35-7.28 (m, 15H, aromatic), 4.97-4.61 (m, 6H), 4.46 (d, 1H, *J* = 7.8 Hz), 3.98-3.87 (m, 2H), 3.74-3.66 (m, 2H), 3.61-3.55 (m, 2H), 3.45-3.38 (m, 2H), 1.68-1.64 (m, 2H), 1.47-1.41 (m, 2H), 0.96 (t, 3H, J = 7.4 Hz). See Supplementary Figure 27.

^{13}C-NMR (100 MHz, $CDCl_3$): δ (ppm) 138.5-127.6, 103.7, 84.5, 82.3, 77.7, 77.2, 75.7, 75.1, 74.9, 74.9, 70.1, 62.1, 31.8, 31.6, 29.6, 19.2, 13.8. See Supplementary Figure 28.

IR (Nujol): ν (cm^{-1}) 3441, 2088, 1635, 1215, 1068, 756, 669.

HRMS (ESI): m/z calcd for $C_{31}H_{39}O_6$ $[M+H]^+$, 507.2747, found: 507.2752.

Butyl 2,3-di-*O*-benzoyl-4-*O*-benzyl-β-D-glucopyranoside 11e (Method D)

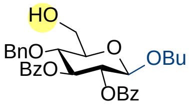

11e

(44mg, 83% yield) as a colorless oil. R_f 0.3 (Hexane/EtOAc 3:1); $[\alpha]_D^{20}$+50.2 (c 0.3, $CHCl_3$).

^{1}H-NMR (500 MHz, $CDCl_3$) δ (ppm) 7.96-7.94 (m, 4H, aromatic), 7.69-7.68 (m, 4H, aromatic), 7.67-7.46 (m, 8H, aromatic), 7.40-7.37 (m, 4H, aromatic), 7.21-7.16 (m, 5H, aromatic), 5.75 (t, 1H, *J* = 9.6 Hz), 5.35 (t, 1H, *J* = 7.7 Hz), 4.73 (d, 1H, J = 7.9 Hz), 4.62 (s, 2H), 4.01-3.82 (m, 4H), 3.64-3.61 (m, 1H), 3.55-3.52 (m, 1H), 2.01-1.99 (m, 1H), 1.52-1.49 (m, 2H), 1.26-1.23 (m, 2H), 0.76 (t, 3H, *J* = 7.4 Hz). See Supplementary Figure 29.

^{13}C-NMR (125 MHz, $CDCl_3$) δ (ppm) 165.7, 165.3, 145.6, 137.2, 133.1-124.5, 101.2, 76.7, 75.6, 75.4, 75.0, 74.8, 72.2, 70.1, 61.7, 31.4, 29.7, 29.3, 27.0, 22.7, 18.8, 14.1, 13.5. See Supplementary Figure 30.

IR (Nujol): ν (cm^{-1}) 3421, 3018, 2399, 2015, 1635, 1215, 927, 756, 669, 522 .

HRMS (ESI): m/z calcd for $C_{31}H_{34}O_8$ $[M+H]^+$, 535.2332, found: 535.2329.

1,6-Anhydro-2,3-di-*O*-*tert*-butyldimethylsilyl-4-O-*para*-methoxybenzyl-β-D-glucopyranose 12e (Method D)

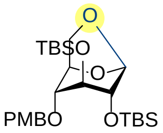

12e

(46mg, 91% yield) as a white solid (m.p. 61-63 °C). R_f 0.3 (Hexane/EtOAc 9:1); $[\alpha]_D^{20}$-30.1 (c 0.3, $CHCl_3$).

^{1}H-NMR (500 MHz, $CDCl_3$): δ (ppm) 7.30-7.26 (m, 2H, aromatic), 6.87-6.85 (m, 2H, aromatic), 5.28 (s, 1H), 4.61 (d, 1H, *J* = 12.1 Hz), 4.55-4.52 (m, 2H), 4.01 (d, 1H, *J* = 6.8 Hz), 3.8 (s, 3H),

3.75 (t, 1H, *J* = 1.5 Hz), 3.66 (t, 1H, *J* = 6.4 Hz), 3.47 (d, 1H, *J* = 1.1 Hz), 3.16 (s, 1H), 0.92-0.86 (m, 18H), 0.11-0.06 (m, 12H). See Supplementary Figure 31.

^{13}C-NMR (125 MHz, $CDCl_3$): δ (ppm) 159.2, 130.3, 129.3, 113.8, 102.3, 77.7, 76.7, 74.3, 72.3, 71.8, 70.7, 64.8, 55.3, 25.8, 25.6, 18.1, 17.7, -4.3, -4.6, -4.7, -4.8. See Supplementary Figure 32.

IR (Nujol): ν (cm^{-1}) 3421, 3018, 2956, 2929, 2399, 2063, 1635, 1514, 1251, 1215, 1107, 754, 669.

HRMS (ESI): m/z calcd for $C_{26}H_{46}O_6Si_2$ $[M+H]^+$, 511.2911, found: 511.2922.

Butyl 4,6-*O*-benzylidene-β-D-glucopyranoside 13e (Method D)

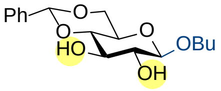

13e

(30mg, 88% yield) as a white solid (m.p. 161-163 ˚C). R_f 0.3 (Hexane/EtOAc 2:3); $[\alpha]_D^{20}$ -79.9 (c 0.9, $CHCl_3$).

^{1}H-NMR (500 MHz, $CDCl_3$): δ (ppm) 7.51-7.25 (m, 5H, aromatic), 5.53 (s, 1H), 4.39 (d, 1H, *J* = 7.8 Hz), 4.34 (dd, 1H, *J* = 4.9 Hz, *J* = 10.5 Hz), 3.93-3.88 (m, 1H), 3.84-3.77 (m, 2H), 3.59-3.52 (m, 4H), 2.76 (s, 1H), 2.56 (s, 1H), 1.64-1.59 (m, 2H), 1.41-1.37 (m, 2H), 0.93 (t, 3H, *J* = 7.4 Hz). See Supplementary Figure 33.

^{13}C-NMR (125 MHz, $CDCl_3$): δ (ppm) 136.9, 129.3, 128.3, 16.23, 103.1, 101.9, 80.6, 74.6, 73.1, 70.3, 68.7, 66.4, 31.6, 19.1, 13.8. See Supplementary Figure 34.

IR (Nujol): ν (cm^{-1}) 3429, 3018, 2399, 2065, 1635, 1215, 1103, 1072, 754, 669.

HRMS (ESI): m/z calcd for $C_{17}H_{24}O_6$ $[M+Na]^+$, 347.1471, found: 347.1471.

Butyl 4,6-di-*O*-benzyl-β-D-glucopyranoside 14e (Method D)

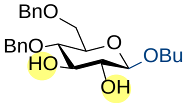

14e

(37mg, 86% yield) as a colorless oil. R_f 0.4 (CH_2Cl_2/EtOAc 2:1); $[\alpha]_D^{20}$ +15.4 (c 0.1, $CHCl_3$).

^{1}H-NMR (500 MHz, $CDCl_3$): δ (ppm) 7.38-7.27 (m, 10H, aromatic), 4.84 (d, 1H, *J* = 11.2 Hz), 4.67-4.57 (m, 3H), 4.27 (d, 1H, *J* = 7.8 Hz), 3.97-3.92 (m, 1H), 3.79 (dd, 1H, *J* = 1.8 Hz, *J* = 10.8 Hz), 3.74-3.70 (m, 3H), 3.57-3.48 (m, 3H), 3.42 (t, 1H, *J* = 7.9 Hz), 2.70 (s, 1H), 2.61 (s, 1H), 1.66-1.63 (m, 2H), 1.42-1.40 (m, 2H), 0.96 (t, 3H, *J* = 7.4 Hz). See Supplementary Figure 35.

^{13}C-NMR (125 MHz, $CDCl_3$): δ (ppm) 138.2, 138.1, 128.5-127.6, 102.4, 77.6, 75.1, 74.6, 74.1, 73.5, 69.8, 68.9, 31.7, 19.1, 13.8. See Supplementary Figure 36.

IR (Nujol): ν (cm^{-1}) 3496, 2088, 1635, 1217, 1047, 748, 580, 511, 499.

HRMS (ESI): m/z calcd for $C_{24}H_{32}O_6$ $[M+Na]^+$, 439.2097, found: 439.2096.

Butyl 3,6-di-*O*-benzyl-D-glucopyranoside 15e (Method D)

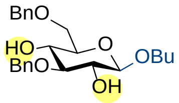

15e

(36mg, 83% yield) as a colorless oil. R_f 0.3 (Hexane/EtOAc 3:1).

^{1}H-NMR (500 MHz, $CDCl_3$, 1α/10β): δ (ppm) 7.39-7.25 (m, 11H, aromatic), 5.00 (d, 1H, *J* = 11.5 Hz), 4.96 (d, 1H, *J* = 11.6 Hz), 4.86 (d, 1H, *J* = 3.9 Hz), 4.80 (d, 1H, *J* = 11.6 Hz), 4.77 (d, 1H, *J* = 4.9 Hz), 4.62-4.54 (m, 2H, both isomers), 4.26 (d, 1H, *J* = 7.7 Hz), 3.92-3.87 (m, 1H), 3.78-3.68 (m, 2H, both isomers), 3.61 (t, 1H, *J* = 9.0 Hz), 3.57-3.48 (m, 3H, both isomers), 3.42 (t, 1H, *J* = 8.9 Hz), 2.64 (s, 1H), 2.46, (s, 1H), 2.34 (s, 1H), 2.16 (s, 1H), 1.63-1.59 (m, 2H, both isomers), 1.39-0.94 (m, 2H, both isomers), 0.92 (t, 3H, *J* = 7.4 Hz, both isomers). See Supplementary Figure 37.

^{13}C-NMR (125 MHz, $CDCl_3$): δ (ppm) 138.6, 127.6, 104.5, 103.4, 102.7, 101.7, 98.4, 83.7, 83.1, 74.9, 74.6, 74.3, 74.2, 73.7, 73.6, 72.7, 71.6, 70.7, 70.4, 70.1, 69.8, 69.7, 68.0, 31.6, 19.1, 13.8. See Supplementary Figure 38.

IR (Nujol): ν (cm^{-1}) 3421, 3018, 2399, 2088, 1635, 1215, 1047, 927, 756, 669.

HRMS (ESI): m/z calcd for $C_{24}H_{32}O_6$ $[M+Na]^+$, 439.2097, found: 439.2097.

Butyl 3,4-di-*O*-benzyl-D-glucopyranoside 16e (Method D)

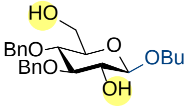

16e

(33mg, 79% yield) as a colorless oil. R_f 0.3 (Hexane/EtOAc 2:3).

^{1}H-NMR (500 MHz, $CDCl_3$, 1α/1.1β): δ (ppm) 7.39-7.25 (m, 20H, aromatic), 4.97-4.83 (m, 6H, both isomers), 4.65 (d, 2H, *J* = 10.9 Hz, both isomers), 4.29 (d, 1H, *J* = 7.7 Hz), 3.89-3.38 (m, 15H, both isomers), 1.95 (br, 4H, both isomers), 1.61-1.58 (m, 4H, both isomers), 1.41-1.36 (m, 4H, both isomers), 0.93 (t, 6H, *J* = 7.4 Hz, both isomers). See Supplementary Figure 39.

^{13}C-NMR (125 MHz, $CDCl_3$): δ (ppm) 138.7-127.7, 102.8, 98.3, 84.3, 83.4, 77.3, 77.2, 75.4, 75.3, 75.1, 74.8, 73.2, 71.1, 70.1, 68.0, 62.1, 61.9, 31.7, 31.5, 29.7, 19.4, 19.2, 13.8. See Supplementary Figure 40.

IR (Nujol): ν (cm^{-1}) 3495, 3018, 2399, 2088, 1635, 1215, 1045, 927, 758, 669.

HRMS (ESI): m/z calcd for $C_{24}H_{32}O_6$ $[M+Na]^+$, 439.2097, found: 439.2102.

Butyl 2,6-di-*O*-benzyl-D-glucopyranoside 17e (Method D)

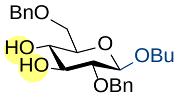

17e

(34mg, 84% yield) as a colorless oil. R_f 0.3 (Hexane/EtOAc 1:1)

^{1}H-NMR (500 MHz, $CDCl_3$, 1α/20β): δ (ppm) 7.38-7.28 (m, 10H, aromatic), 4.99 (d, 1H, *J* = 11.4 Hz), 4.79 (d, 1H, *J* = 3.5 Hz), 4.71-4.56 (m, 3H, both isomers), 4.42 (d, 1H, *J* = 7.7 Hz), 3.99-3.93 (m, 1H), 3.80 (dd, 1H, *J* = 4.2 Hz, *J* = 10.4 Hz), 3.80 (dd, 1H, *J* = 5.3 Hz, *J* = 10.4 Hz), 3.58-3.54 (m, 3H, both isomers), 3.52-3.46 (m, 1H), 3.29-3.23 (m, 1H), 2.89 (s, 1H), 2.63 (s, 1H), 1.71-1.59 (m, 2H, both isomers), 1.50-1.39 (m, 2H, both isomers), 0.96 (t, 3H, *J* = 7.4 Hz, both isomers). See Supplementary Figure 41.

^{13}C-NMR (125 MHz, $CDCl_3$): δ (ppm) 138.3, 137.8, 128.5-127.7, 103.3, 96.5, 80.7, 79.3, 76.1, 74.3, 73.9, 73.7, 73.5, 72.8, 72.7, 71.7, 71.1, 70.3, 69.8, 69.7, 67.9, 31.8, 31.5, 19.4, 19.3, 13.8. See Supplementary Figure 42.

IR (Nujol): ν (cm^{-1}) 3495, 2018, 2399, 2015, 1635, 1217, 1049, 927, 771, 669.

HRMS (ESI): m/z calcd for $C_{24}H_{32}O_6$ $[M+Na]^+$, 439.2097, found: 439.2096.

Butyl 3,4-di-*O*-benzyl-D-glucopyranoside 18e (Method D)

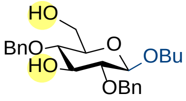

18e

(33mg, 80% yield) as a colorless oil. R_f 0.3 (Hexane/EtOAc 1:1).

^{1}H-NMR (500 MHz, $CDCl_3$, 1α/2.5β): δ (ppm) 7.41-7.28 (m, 13H, aromatic), 4.94 (d, 1H, *J* = 11.4 Hz), 4.76-4.66 (m, 5H, both isomers), 4.60 (d, 1H, *J* = 5.15 Hz), 4.15 (t, 1H, *J* = 9.2 Hz), 3.89-3.61 (m, 6H, both isomers), 3.48 (t, 1H, *J* = 9.15 Hz), 3.37-3.32 (m, 3H, both isomers), 2.52 (s, 1H), 1.61-1.58 (m, 2H, both isomers), 1.42-1.39 (m, 2H, both isomers), 0.94 (t, 3H, *J* = 7.4 Hz). See Supplementary Figure 43.

^{13}C-NMR (125 MHz, $CDCl_3$): δ (ppm) 138.3, 138.0, 128.5-127.8, 101.3, 96.3, 79.8, 70.6, 79.3, 75.3, 74.6, 73.4, 72.8, 72.1, 71.8, 70.6, 70.3, 67.9, 66.5, 62.1, 31.5, 29.7, 19.4, 13.8. See Supplementary Figure 44.

IR (Nujol): ν (cm^{-1}) 3495, 3018, 2399, 2088, 1635, 1215, 1045, 927, 767, 669, 497.

HRMS (ESI): m/z calcd for $C_{24}H_{32}O_6$ $[M+Na]^+$, 439.2097, found: 439.2097.

Butyl 2,3-di-*O*-benzyl-D-glucopyranoside 19e (Method D)

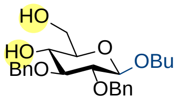

19e

(32mg, 78% yield) as a colorless oil. R_f 0.3 (Hexane/EtOAc 1:1)

^{1}H-NMR (500 MHz, $CDCl_3$, 1α/11β): δ (ppm) 7.39-7.28 (m, 11H, aromatic), 5.06 (d, 1H, *J* = 11.5 Hz), 5.01-4.97 (dd, 2H, *J* = 4.1 Hz, *J* = 11.3 Hz), 4.78-4.66 (m, 2H, both isomers), 4.48 (d, 1H, *J* = 7.4 Hz), 3.99-3.41 (m, 8H, both isomers), 3.37-3.33 (m, 1H), 2.39 (s, 1H), 2.13 (s, 1H), 1.69-1.65 (m, 2H, both isomers), 1.48-1.44 (m, 2H, both isomers), 0.96 (t, 3H, *J* = 7.4 Hz, both isomers). See Supplementary Figure 45.

^{13}C-NMR (125 MHz, $CDCl_3$): δ (ppm) 138.8-117.8, 103.8, 96.8, 83.9, 81.9, 81.3, 80.1, 75.3, 75.2, 74.8, 74.7, 72.8, 70.7, 70.6, 70.5, 70.1, 67.9, 62.7, 62.5, 31.8, 31.5, 19.4, 19.3, 13.8. See Supplementary Figure 46.

IR (Nujol): ν (cm^{-1}) 3498, 2399, 2088, 1645, 1215, 1049, 925, 748, 669.

HRMS (ESI): m/z calcd for $C_{24}H_{32}O_6$ $[M+Na]^+$, 439.2097, found: 439.2099.

Butyl 2,3-di-*O*-benzoyl-β-D-glucopyranoside 20e (Method D)

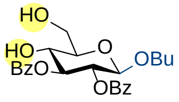

20e

(35mg, 79% yield) as a colorless oil. R_f 0.4 (Hexane/EtOAc 2:3); $[\alpha]_D^{20}$ +124.3 (c 0.9, $CHCl_3$).

^{1}H-NMR (500 MHz, $CDCl_3$): δ (ppm) 8.01-7.97 (m, 4H, aromatic), 7.55-7.51 (m, 2H, aromatic), 7.41-7.38 (m, 4H, aromatic), 5.45-5.42 (m, 2H), 4.75-4.73 (m, 1H), 4.04-3.89 (m, 4H), 3.63-3.59 (m, 1H), 3.56-3.52 (m, 1H), 3.31 (d, 1H, *J* = 3.3 Hz), 2.23 (br, 1H), 1.69 (s, 1H), 1.54-1.48 (m, 2H), 1.27-1.21 (m, 2H), 0.76 (t, 3H, *J* = 7.4 Hz). See Supplementary Figure 47.

^{13}C-NMR (125 MHz, $CDCl_3$): δ (ppm) 167.6, 165.3, 133.5, 133.1, 130.1, 129.7, 129.5, 128.8, 128.5, 128.3, 101.2, 75.8, 71.5, 70.1, 70.0, 62.3, 31.4, 18.8, 13.6. See Supplementary Figure 48.

IR (Nujol): ν (cm^{-1}) 3514, 3018, 2399, 2088, 1635, 1217, 1043, 927, 756, 669.

HRMS (ESI): m/z calcd for $C_{24}H_{28}O_8$ $[M+H]^+$, 445.1862, found: 445.1870.

Butyl 2-*O*-benzyl-D-glucopyranoside 21e (Method D)

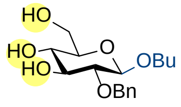

21e

(25mg, 79% yield) as a colorless oil. R_f 0.3 (Hexane/EtOAc 1:3)

^{1}H-NMR (500 MHz, $CDCl_3$, 1α/6β): δ (ppm) 7.71-7.28 (m, 7H, aromatic), 4.99 (d, 1H, *J* = 11.4 Hz, 4.77 (d, 1H, *J* = 3.4 Hz), 4.68-4.65 (m, 2H, both isomers), 4.46 (d, 1H, *J* = 7.8 Hz), 3.98-3.90 (m, 2H, both isomers), 3.84-3.81 (m, 1H, both isomers), 3.71-3.66 (m, 1H), 3.59-3.54 (m, 3H, both isomers), 3.40-3.34 (m, 2H, both isomers), 3.24-3.16 (m, 2H, both isomers), 3.8 (br, 2H), 2.2 (br, 1H), 1.68-1.64 (m, 2H, both isomers), 1.46-1.38 (m, 2H, both isomers), 0.96 (t, 3H, *J* = 7.4 Hz, both isomers). See Supplementary Figure 49.

^{13}C-NMR (125 MHz, $CDCl_3$): δ (ppm) 138.2, 137.9, 135.2, 134.4, 131.5, 131.5, 130.9, 130.3, 128.5, 128.1, 128.1, 128.0, 103.4, 96.4, 80.8, 79.4, 76.0, 75.0, 74.3, 72.8, 72.7, 70.7, 70.6, 70.5, 70.0, 67.9, 62.5, 62.4, 46.7, 31.8, 31.5, 19.4, 19.2, 13.8. See Supplementary Figure 50.

IR (Nujol): ν (cm^{-1}) 3466, 3020, 2399, 2015, 1635, 1217, 1045, 925, 769, 669.

HRMS (ESI): m/z calcd for $C_{17}H_{26}O_6$ $[M+H]^+$, 327.1808, found: 327.1809.

Butyl 3-*O*-benzyl-D-glucopyranoside 22e (Method D)

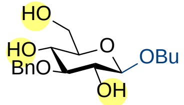

22e

(26mg, 81% yield) as a colorless oil. R_f 0.3 (Hexane/EtOAc 1:3); $[\alpha]_D^{20}$ +16.0 (c 0.05, $CHCl_3$).

^{1}H-NMR (500 MHz, $CDCl_3$): δ (ppm) 7.40-7.28 (m, 5H, aromatic), 5.05 (d, 1H, *J* = 11.7 Hz), 4.76 (d, 1H, *J* = 11.7 Hz), 4.33 (d, 1H, *J* = 7.7 Hz), 3.95-3.90 (m, 2H), 3.80 (dd, 1H, *J* = 5.2 Hz, *J* = 11.8 Hz), 3.62-3.52 (m, 3H), 3.46-3.40 (m, 2H), 2.35 (br, 2H), 2.1 (br, 1H), 1.66-1.58 (m, 2H), 1.44-1.39 (m, 2H), 0.96 (t, 3H, *J* = 7.4 Hz). See Supplementary Figure 51.

^{13}C-NMR (125 MHz, $CDCl_3$): δ (ppm) 138.5, 128.6, 128.0, 102.9, 83.6, 77.3, 77.0, 76.7, 75.1, 74.7, 74.6, 70.3, 70.1, 62.7, 31.7, 19.1, 13.8. See Supplementary Figure 52.

IR (Nujol): ν (cm^{-1}) 3498, 3018, 2399, 2065, 1635, 1215, 1045, 927, 761, 669.

HRMS (ESI): m/z calcd for $C_{17}H_{26}O_6$ $[M+H]^+$, 327.1808, found: 327.1808.

Butyl 4-*O*-benzyl-D-glucopyranoside 23e (Method D)

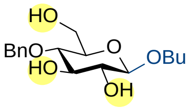

23e

(26mg, 80% yield) as a colorless oil. R_f 0.3 (Hexane/EtOAc 1:3).

^{1}H-NMR (500 MHz, $CDCl_3$, 1α/2.9β): δ (ppm) 7.38-7.28 (m, 7H, aromatic), 4.91-4.86 (m, 1H, both isomers), 4.75-4.73 (m, 1H, both isomers), 4.31 (d, 1H, *J* = 7.7 Hz), 3.92-3.41 (m, 10H, both isomers), 2.86 (s, 1H), 2.74 (s, 1H), 2.1 (br, 1H), 1.65-1.58 (m, 2H, both isomers), 1.42-1.38 (m, 2H, both isomers), 0.95 (t, 3H, *J* = 7.4 Hz, both isomers). See Supplementary Figure 53.

^{13}C-NMR (125 MHz, $CDCl_3$): δ (ppm) 138.2, 138.1, 128.6, 128.1, 128.0, 102.5, 97.9, 77.2, 77.1, 76.5, 75.3, 75.2, 74.7, 74.1, 72.8, 70.8, 70.0, 68.1, 62.0, 61.9, 31.6, 31.5, 19.4, 19.1, 13.8. See Supplementary Figure 54.

IR (Nujol): ν (cm^{-1}) 3456, 3018, 2399, 2015, 1635, 1215, 1045, 927, 771, 669.

HRMS (ESI): m/z calcd for $C_{17}H_{26}O_6$ $[M+H]^+$, 327.1808, found: 327.1799.

Butyl 6-*O*-benzyl-D-glucopyranoside 24e (Method D)

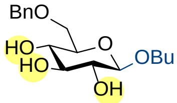

24e

(26mg, 80% yield) as a colorless oil. R_f 0.2 (Hexane/EtOAc 1:3)

^{1}H-NMR (500 MHz, $CDCl_3$, 1α/1.7β): δ (ppm) 7.70-7.28 (m, 10H, aromatic), 4.87 (d, 1H, *J* = 3.8 Hz), 4.64-4.57 (m, 3H, both isomers), 4.28 (d, 1H, *J* = 7.7 Hz), 3.91-3.86 (m, 1H), 3.78-3.44 (m, 14H, both isomers), 3.39 (t, 1H, *J* = 8.1 Hz), 3.31-3.12 (m, 2H, both isomers), 2.6 (br, 1H), 1.63-1.59 (m, 3H, both isomers), 1.41-1.36 (m, 4H, both isomers), 0.93 (t, 5H, *J* = 7.4 Hz, both isomers). See Supplementary Figure 55.

^{13}C-NMR (125 MHz, $CDCl_3$): δ (ppm) 149.9-118.9, 102.5, 98.1, 76.2, 74.7, 74.1, 73.7, 73.6, 73.5, 72.1, 71.8, 71.2, 70.2, 70.1, 69.8, 69.7, 68.1, 46.7, 31.6, 31.4, 19.3, 19.1, 13.8. See Supplementary Figure 56.

IR (Nujol): ν (cm^{-1}) 3514, 3018, 2980, 2399, 2065, 1635, 1419, 1215, 1045, 927, 769, 669.

HRMS (ESI): m/z calcd for $C_{17}H_{26}O_6$ $[M+H]^+$, 327.1808, found: 327.1813.

Methyl-*O*-((3,4,6-tri-*O*-benzyl)-β-D-glucopyranosyl)-(1→6)-2,3,4-tri-*O*-benzyl-α-D-glucopyranoside 1ai (Method D)

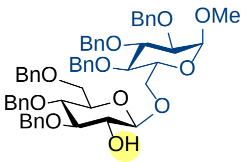

1ai

(34mg, 78% yield) as a white solid (m.p. 140-142˚C). R_f 0.3 (Hexane/EtOAc 3:1); $[\alpha]_D^{20}$ +132.0 (c 0.1, $CHCl_3$).

^{1}H-NMR (500 MHz, $CDCl_3$): δ (ppm) 7.36-7.25 (m, 28H, aromatic), 7.17-7.15 (m, 2H, aromatic), 4.98-4.50 (m, 13H), 4.22 (d, 1H, *J* = 6.9 Hz), 4.14 (dd, 1H, *J* = 2.1 Hz, *J* = 11.0 Hz), 3.99 (t, 1H, *J* = 9.2 Hz), 3.82-3.80 (m, 1H), 3.73-3.65 (m, 3H), 3.56-3.45 (m, 6H), 3.37 (s, 3H), 2.49 (s, 1H). See Supplementary Figure 57.

^{13}C-NMR (125 MHz, $CDCl_3$): δ (ppm) 138.7-127.5, 103.5, 98.1, 84.5, 82.0, 79.7, 78.0, 77.5, 77.3, 77.0, 76.7, 75.7, 75.3, 75.1, 75.0, 74.5, 73.4, 69.8, 68.9, 68.7, 55.3. See Supplementary Figure 58.

IR (Nujol): ν (cm^{-1}) 3514, 3018, 2399, 2065, 1635, 1215, 1045, 927, 752, 669, 522.

HRMS (ESI): m/z calcd for $C_{55}H_{60}O_{11}$ $[M+H]^+$, 897.4214, found: 897.4215.

Methyl-*O*-((3,4,6-tri-*O*-benzyl)-β-D-glucopyranosyl)-(1→4)-2,3,6-tri-*O*-benzyl-α-D-glucopyranoside 2ai (Method D)

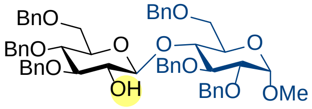

2ai

(31mg, 69% yield) as a colorless oil. R_f 0.3 (Hexane/EtOAc 3:1); $[\alpha]_D^{20}$ +77.8 (c 0.1, $CHCl_3$).

^{1}H-NMR (500 MHz, $CDCl_3$): δ (ppm) 7.38-7.15 (m, 30H, aromatic), 5.04-4.43 (m, 14H), 4.00-3.97 (m, 3H), 3.81-3.79 (m, 1H), 3.68 (dd, 1H, *J* = 1.9 Hz, *J* = 11.3 Hz), 3.61 (t, 1H, *J* = 9.3 Hz), 3.52-3.44 (m, 5H), 3.38 (s, 3H), 3.27 (d, 1H, *J* = 2.0 Hz), 3.24-3.21 (m, 1H). See Supplementary Figure 59.

^{13}C-NMR (125 MHz, $CDCl_3$): δ (ppm) 139.3-127.0, 107.6, 103.1, 98.2, 84.5, 80.9, 79.5, 75.6, 75.2, 75.1, 75.0, 74.8, 73.7, 73.5, 73.3, 69.5, 68.8, 68.5, 55.2. See Supplementary Figure 60.

IR (Nujol): ν (cm^{-1}) 3498, 3018, 2399, 2015, 1635, 1213, 1028, 927, 765, 669, 543.

HRMS (ESI): m/z calcd for $C_{55}H_{60}O_{11}$ $[M+H]^+$, 897.4214, found: 897.4218.

Methyl-*O*-((3,4,6-tri-*O*-benzyl)-β-D-glucopyranosyl)-(1→2)-3-*O*-benzyl-4,6-*O*-benzylidene-α-D-glucopyranoside 3ai (Method D)

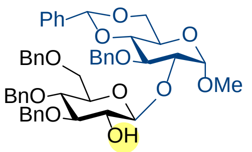

3ai

(30mg, 77% yield) as a white solid (m.p. 172-174°C), R_f 0.3 (Hexane/EtOAc 3:1); $[\alpha]_D^{20}$ +102.2 (c 0.1, $CHCl_3$).

^{1}H-NMR (400 MHz, $CDCl_3$): δ (ppm) 7.53-7.19 (m, 25H, aromatic), 5.60 (s, 1H), 4.98-4.78 (m, 6H), 4.61-4.51 (m, 4H), 4.33 (dd, 1H, *J* = 4.7 Hz, *J* = 10.0 Hz), 4.10 (t, 1H, *J* = 9.3 Hz), 3.93-3.87 (m, 1H), 3.81-3.57 (m, 8H), 3.58-2.52 (m, 4H), 2.52 (s, 1H). See Supplementary Figure 61.

^{13}C-NMR (100 MHz, $CDCl_3$): δ (ppm) 138.7-126.1, 104.7, 101.3, 100.1, 84.3, 82.7, 79.8, 77.6, 75.3, 75.1, 75.0, 74.9, 73.5, 69.1, 62.2, 55.4, 29.7. See Supplementary Figure 62.

IR (Nujol): ν (cm^{-1}) 3498, 3018, 2399, 2088, 1651, 1215, 1047, 927, 746, 621, 526.

HRMS (ESI): m/z calcd for $C_{48}H_{52}O_{11}$ $[M+Na]^+$, 827.3407, found: 827.3411.

Methyl-O-((3,4,6-tri-*O*-benzyl)-α-D-mannopyranosyl)-(1→3)-2-*O*-benzyl-4,6-*O*-benzylidene-α-D-glucopyranoside 4ai (Method D)

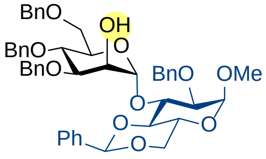

4ai

(30mg, 76% yield) as a colorless oil. R_f 0.3 (Hexane/EtOAc 3:1); $[\alpha]_D^{20}$ +44.8 (c 0.5, $CHCl_3$).

^{1}H-NMR (500 MHz, $CDCl_3$) δ (ppm) 7.47-7.18 (m, 25H), 5.52 (s, 1H), 5.43 (s, 1H), 4.84 (d, 1H, *J* = 11.0 Hz), 4.69-4.61 (m, 5H), 4.52 (t, 2H, *J* = 9.5 Hz), 4.41 (d, 1H, *J* = 12.0 Hz), 4.33-4.25 (m, 2H), 4.16-4.12 (m, 2H), 3.97 (t, 1H, *J* = 9.3 Hz), 3.91-3.89 (m, 1H), 3.87-3.81 (m, 1H), 3.73-3.56 (m, 4H), 3.48-3.45 (m, 1H), 3.39 (s, 3H), 2.34 (s, 1H). See Supplementary Figure 63.

^{13}C-NMR (125 MHz, $CDCl_3$) δ (ppm) 138.8-126.1, 101.5, 99.4, 98.7, 82.6, 79.8, 78.1, 74.9, 74.2, 73.5, 73.3, 71.6, 71.0, 69.0, 68.6, 68.2, 62.9, 55.3, 29.7. See Supplementary Figure 64.

IR (Nujol): ν (cm^{-1}) 3541, 3018, 2399, 2088, 1635, 1419, 1215, 1045, 908, 733, 671, 495.

HRMS (ESI): m/z calcd for $C_{48}H_{52}O_{11}$ $[M+Na]^+$, 827.3407, found: 827.3407.

Methyl-O-((4,6-*O*-benzylidene)-β-D-glucopyranosyl)-(1→6)-2,3-di-*O*-*tert*-butyldimethylsilyl-4-*O*-benzyl-α-D-glucopyranoside 5ai (Method D)

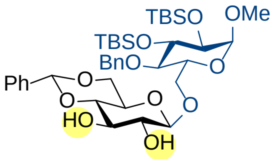

5ai

(30mg, 81% yield) as a colorless oil. R_f 0.3 (Hexane/EtOAc 3:2); $[\alpha]_D^{20}$+72.6 (c 0.5, $CHCl_3$).

^{1}H-NMR (500 MHz, $CDCl_3$) δ (ppm) 7.51-7.28 (m, 10H, aromatic), 4.93 (d, 1H, *J* = 11.8 Hz), 4.68 (d, 1H, *J* = 3.4 Hz), 4.60 (d, 1H, *J* = 11.9 Hz), 4.37 (d, 1H, *J* = 7.7 Hz), 4.33 (dd, 1H, *J* = 4.9 Hz, *J* = 10.5 Hz), 4.12 (dd, 1H, *J* = 2.0 Hz, *J* = 10.8 Hz), 3.97 (t, 1H, *J* = 8.8 Hz), 3.85-3.75 (m, 4H), 3.69-3.54 (m, 4H), 3.46-3.41 (m, 1H), 3.38 (s, 3H), 3.34 (t, 1H, *J* = 9.8 Hz), 2.70 (s, 1H), 1.87 (br, 1H), 0.95 (s, 18H), 0.14-0.02 (m, 12H). See Supplementary Figure 65.

^{13}C-NMR (125 MHz, $CDCl_3$) δ (ppm) 138.5-126.3, 103.6, 101.9, 100.3, 80.5, 79.6, 74.7, 74.3, 74.2, 74.1, 73.2, 70.0, 69.0, 68.6, 67.9, 66.5, 55.0, 26.3, 26.2, 25.6, 18.4, 18.0, -2.9, -3.2, -4.0, -4.4 See Supplementary Figure 66.

IR (Nujol): ν (cm^{-1}) 3498, 3018, 2399, 2065, 1635, 1417, 1215, 1043, 927, 746, 669, 542.

HRMS (ESI): m/z calcd for $C_{39}H_{62}O_{11}Si_2$ $[M+Na]^+$, 785.3728, found: 785.3733.

Methyl-O-((3,4,6-tri-*O*-benzyl)-β-D-glucopyranosyl)-(1→6)-2,3-di-*O*-*tert*-butyldimethylsilyl-4-*O*-benzyl-α-D-glucopyranoside 6ai (Method D)

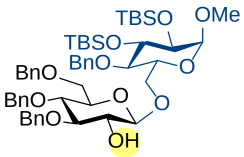

6ai

(38mg, 82% yield) as a colorless oil. R_f 0.3 (Hexane/EtOAc 3:2); $[\alpha]_D^{20}$+114.7 (c 0.9, $CHCl_3$).

^{1}H-NMR (400 MHz, $CDCl_3$) δ (ppm) 7.36-7.14 (m, 20H, aromatic), 4.92-4.78 (m, 4H), 4.63 (d, 1H, *J* = 3.4 Hz), 4.57-4.47 (m, 4H), 4.18 (d, 1H, *J* = 8.7 Hz), 4.09 (d, 1H, *J* = 13.3 Hz), 3.92 (t, 1H, *J* = 10.9 Hz), 3.79-3.77 (m, 1H), 3.70-3.50 (m, 7H), 3.42 (br, 1H), 3.33-3.25 (m, 4H), 2.51 (s, 1H), 0.90 (s, 18H), 0.08-0.01 (m, 12H). See Supplementary Figure 67.

^{13}C-NMR (100 MHz, $CDCl_3$) δ (ppm) 138.7-127.0, 103.3, 100.2, 84.5, 79.7, 77.5, 76.7, 75.4, 75.1, 74.6, 74.4, 74.3, 74.2, 73.5, 70.1, 68.9, 68.7, 54.9, 26.5, 26.2, 18.4, 18.0, -2.9, -3.2, -4.0, -4.4. See Supplementary Figure 68.

IR (Nujol): ν (cm^{-1}) 3466, 3018, 2399, 2065, 1635, 1419, 1215, 1045, 908, 761, 669.

HRMS (ESI): m/z calcd for $C_{53}H_{76}O_{11}Si_2$ $[M+H]^+$, 945.5004, found: 945.5010.

Methyl-O-((3,4,6-tri-*O*-benzyl)-α-D-mannopyranosyl)-(1→6)-2,3-di-*O*-*tert*-butyldimethyl silyl-4-*O*-benzyl-α-D-glucopyranoside 7ai (Method D)

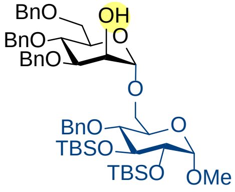

7ai

(36mg, 80% yield) as a colorless oil. R_f 0.3 (Hexane/EtOAc 3:2); $[\alpha]_D^{20}$+104.1 (c 0.9, $CHCl_3$).

^{1}H-NMR (500 MHz, $CDCl_3$) δ (ppm) 7.38-7.19 (m, 20H, aromatic), 4.89-4.86 (m, 2H), 4.77-4.44 (m, 7H), 4.11 (s, 1H), 4.07 (dd, 1H, *J* = 1.7 Hz, *J* = 10.6 Hz), 3.98 (d, 1H, *J* = 2.5 Hz), 3.93 (t, 1H, *J* = 8.8 Hz), 3.85-3.81 (m, 1H), 3.76-3.67 (m, 2H), 3.60 (dd, 1H, *J* = 3.4 Hz, *J* = 9.1 Hz), 3.52 (dd, 1H, *J* = 5.4 Hz, *J* = 10.7 Hz), 3.46 (dd, 1H, *J* = 3.0 Hz, *J* = 9.1 Hz), 3.34-3.27 (m, 5H), 2.42 (s, 1H), 0.92 (s, 18H), 0.11-0.03 (m, 12H). See Supplementary Figure 69.

^{13}C-NMR (125 MHz, $CDCl_3$) δ (ppm) 138.8-127.4, 100.3, 100.0, 81.5, 79.7, 75.6, 75.4, 75.0, 74.6, 74.5, 73.7, 71.6, 70.1, 69.4, 68.5, 68.4, 55.0, 26.6, 26.4, 18.6, 18.2, 0.2, -2.7, -2.9, -3.8, -4.2. See Supplementary Figure 70.

IR (Nujol): ν (cm^{-1}) 3495, 2018, 2399, 2065, 1635, 1215, 1045, 927, 736, 669.

HRMS (ESI): m/z calcd for $C_{53}H_{76}O_{11}Si_2$ $[M+H]^+$, 945.5004, found: 945.5012.

Methyl-O-((2,4,6-tri-*O*-benzyl)-β-D-glucopyranosyl)-(1→6)-2,3-di-*O*-*tert*-butyldimethylsilyl-4-*O*-benzyl-α-D-glucopyranoside 8ai (Method D)

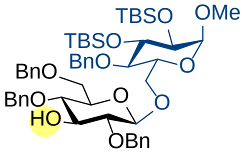

8ai

(18mg, 81% yield) as a colorless oil. R_f 0.3 (Hexane/EtOAc 3:2); $[\alpha]_D^{20}$+16.3 (c 0.5, $CHCl_3$).

^{1}H-NMR (500 MHz, $CDCl_3$) δ (ppm) 7.36-7.21 (m, 20H, aromatic), 5.30 (s, 1H), 5.05 (d, 1H, *J* = 11.4 Hz), 4.83-4.51 (m, 8H), 4.28 (d, 1H, *J* = 7.7 Hz), 4.15 (d, 1H, *J* = 10.7 Hz), 3.94 (t, 1H, *J* = 9.1 Hz), 3.82-3.80 (m, 1H), 3.73-3.67 (m, 4H), 3.61-3.56 (m, 2H), 3.47 (t, 1H, *J* = 8.7 Hz), 3.42-3.41 (m, 1H), 3.34-3.30 (m, 4H), 2.39 (s, 1H), 0.90 (s, 18H), 0.11 (s, 12H). See Supplementary Figure 71.

^{13}C-NMR (125 MHz, $CDCl_3$) δ (ppm) 138.6-126.9, 103.2, 100.2, 81.2, 79.7, 74.9, 74.6, 74.5, 74.4, 74.2, 73.4, 69.9, 69.1, 68.6, 58.5, 54.8, 29.7, 26.3, 26.2, 18.5, 18.4, 17.9, -0.1, -3.0, -3.2, -4.0, -4.4. See Supplementary Figure 72.

IR (Nujol): ν (cm^{-1}) 3541, 3018, 2399, 2065, 1635, 1419, 1217, 1028, 927, 771, 669.

HRMS (ESI): m/z calcd for $C_{53}H_{76}O_{11}Si_2$ $[M+H]^+$, 945.5004, found: 945.5004.

Methyl-O-((2,4,6-tri-*O*-benzyl)-β-D-allopyranosyl)-(1→6)-2,3-di-*O*-*tert*-butyldimethylsilyl-4-*O*-benzyl-α-D-glucopyranoside 9ai (Method D)

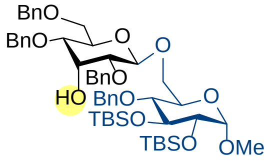

9ai

(18mg, 80% yield) as a colorless oil. R_f 0.3 (Hexane/EtOAc 3:2); $[\alpha]_D^{20}$ +37.7 (c 0.5, $CHCl_3$).

^1H-NMR (400 MHz, $CDCl_3$) δ (ppm) 7.40-7.24 (m, 20H, aromatic), 4.88-4.46 (m, 10H), 4.33 (s, 1H), 4.15 (d, 1H, *J* = 11.3 Hz), 3.97-3.91 (m, 2H), 3.81-3.74 (m, 2H), 3.68-3.61 (m, 3H), 3.50 (dd, 1H, *J* = 3.2 Hz, *J* = 12.2 Hz), 3.40-3.32 (m, 5H), 2.46 (s, 1H), 0.94 (s, 18H), 0.13-0.03 (m, 12H). See Supplementary Figure 73.

^{13}C-NMR (100 MHz, $CDCl_3$) δ (ppm) 138.7-127.0, 100.4, 100.2, 79.5, 77.3, 74.4, 74.2, 73.4, 72.6, 71.9, 71.4, 69.6, 69.3, 68.3, 66.9, 54.5, 26.4, 26.2, 18.4, 17.9, -0.1, -2.9, -3.2, -4.0, -4.4. See Supplementary Figure 74.

IR (Nujol): ν (cm^{-1}) 3495, 2018, 2399, 2088, 1635, 1417, 1217, 1045, 908, 771, 669.

HRMS (ESI): m/z calcd for $C_{53}H_{76}O_{11}Si_2$ $[M+H]^+$, 945.5004, found: 945.5005.

Methyl-O-((2,3,6-tri-*O*-benzyl)-β-D-glucopyranosyl)-(1→6)-2,3-di-*O*-*tert*-butyldimethylsilyl-4-*O*-benzyl-α-D-glucopyranoside 10ai (Method D)

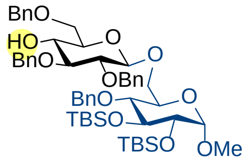

10ai

(35mg, 79% yield) as a colorless oil. R_f 0.3 (Hexane/EtOAc 3:2); $[\alpha]_D^{20}$ +90.9 (c 0.5, $CHCl_3$).

^1H-NMR (400 MHz, $CDCl_3$) δ (ppm) 7.41-7.19 (m, 20H), 5.07 (d, 1H, *J* = 11.2 Hz), 4.95 (d, 1H, *J* = 11.4 Hz), 4.83-4.51 (m, 7H), 4.34 (d, 1H, *J* = 7.0 Hz), 4.17 (d, 1H, *J* = 10.1 Hz), 3.95 (d, 1H, *J*

= 8.8 Hz), 3.83-3.70 (m, 3H), 3.64-3.59 (m 3H), 3.53-3.39 (m, 3H), 3.36-3.31 (m, 4H), 2.61 (s, 1H), 0.95-0.93 (m, 18H), 0.14-0.03 (m, 12H). See Supplementary Figure 75.

^{13}C-NMR (100 MHz, $CDCl_3$) δ (ppm) 138.6-126.9, 103.6, 100.2, 84.2, 81.4, 79.5, 77.3, 75.3, 74.7, 74.4, 74.2, 73.9, 73.7, 72.0, 70.1, 69.8, 68.5, 54.8, 26.4, 26.2, 18.4, 17.9, -3.1, -3.2, -4.1, -4.4. See Supplementary Figure 76.

IR (Nujol): ν (cm^{-1}) 3495, 3018, 2399, 2088, 1635, 1217, 1045, 927, 742, 669.

HRMS (ESI): m/z calcd for $C_{53}H_{76}O_{11}Si_2$ $[M+H]^+$, 945.5004, found: 945.5007.

Methyl-O-((2,3,6-tri-*O*-benzyl)-D-galactopyranosyl)-(1→6)-2,3-di-*O*-*tert*-butyldimethylsilyl-4-*O*-benzyl-α-D-glucopyranoside 10ai (Method D)

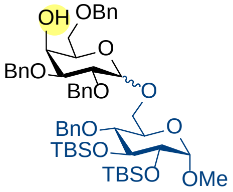

11ai

(35mg, 77% yield) as a colorless oil. R_f 0.3 (Hexane/EtOAc 3:2)

^{1}H-NMR (500 MHz, $CDCl_3$, 1α/2.5β) δ (ppm) 7.41-7.18 (m, 33H, aromatic), 5.09 (d, 1H, *J* = 3.2 Hz), 5.02 (d, 1H, *J* = 11.1 Hz), 4.83-4.68 (m, 7H, both isomers), 4.59-4.51 (m, 5H, both isomers), 4.28 (d, 1H, *J* = 7.8 Hz), 4.17 (d, 1H, *J* = 9.1 Hz), 4.04 (br, 2H, both isomers), 3.96-3.89 (m, 3H, both isomers), 3.84-3.77 (m, 4H, both isomers), 3.74-3.68 (m, 3H, both isomers), 3.65-3.58 (m, 3H, both isomers), 3.53-3.49 (m, 3H, both isomers), 3.40 (t, 1H, *J* = 9.2 Hz), 3.34-3.29 (m, 6H, both isomers), 2.66 (s, 1H), 2.45 (s, 1H), 0.97-0.92 (m, 29H, both isomers), 0.14-0.03 (m, 20H, both isomers). See Supplementary Figure 77.

^{13}C-NMR (125 MHz, $CDCl_3$) δ (ppm) 138.7-126.9, 103.7, 100.2, 80.7, 79.5, 78.6, 75.2, 74.4, 74.2, 73.6, 74.5, 73.2, 72.3, 69.9, 68.9, 68.6, 66.7, 54.8, 26.4, 26.3, 26.2, 26.1, 18.4, 18.0, 17.9, -2.9, -3.2, -4.0, -4.4. See Supplementary Figure 78.

IR (Nujol): ν (cm^{-1}) 3433, 2018, 2929, 2399, 2065, 1635, 1361, 1215, 1072, 839, 769, 669.

HRMS (ESI): m/z calcd for $C_{53}H_{76}O_{11}Si_2$ $[M+H]^+$, 945.5004, found: 945.5012.

Phenyl-S-((3,4,6-tri-*O*-benzyl)-α-D-mannopyranosyl)-(1→4)-2-deoxy-2-(2,2,2-trichloroethoxy carbonylamino-3-*O*-acetyl-6-*O*-*tert*-butyldimethylsilyl-β-D-glucopyranoside 12ai (Method C)

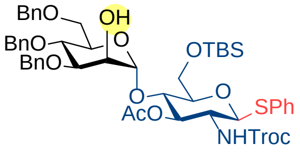

12ai

(41mg, 78% yield) as a colorless oil. R_f 0.4 (Hexane/EtOAc 2:1); $[\alpha]_D^{20}$+22.5 (c 1.5, $CHCl_3$).

^{1}H-NMR (500 MHz, $CDCl_3$) δ (ppm) 7.52-7.16 (m, 20H, aromatic), 5.18 (t, 1H, *J* = 9.6 Hz), 5.15-5.13 (m, 2H), 4.84-4.66 (m, 7H), 4.53-4.49 (dd, 2H, *J* = 5.3 Hz, *J* = 11.5 Hz), 3.94-3.90 (m, 2H), 3.86-3.81 (m, 3H), 3.78-3.65 (m, 5H), 3.42-3.39 (m, 1H), 2.11 (s, 3H), 0.93 (s, 9H), 0.06 (s, 6H). See Supplementary Figure 79.

^{13}C-NMR (125 MHz, $CDCl_3$) δ (ppm) 171.1-127.8, 117.9, 101.1, 95.6, 87.3, 80.1, 79.8, 76.4, 75.2, 75.1, 74.7, 74.0, 73.8, 72.5, 72.3, 69.1, 68.7, 62.8, 55.5, 26.2, 26.1, 21.1, 18.6, -4.9. See Supplementary Figure 80.

IR (Nujol): ν (cm^{-1}) 3456, 3018, 2978, 2399, 2065, 1747, 1635, 1519, 1419, 1219, 1045, 927, 783.

HRMS (ESI): m/z calcd for $C_{50}H_{62}NCl_3SO_{12}Si$ $[M+Na]^+$, 1056.2725, found: 1056.2735.

Ethyl-S-((2-*O*-benzoyl-4,6-*O*-benzylidene)-β-D-galactopyranosyl)-(1→3)-(2-*O*-benzoyl-4,6-*O*-benzylidene)-β-D-galactopyranoside 13ai (Method C)

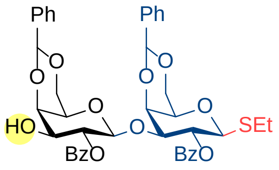

13ai

(26mg, 68% yield) as a white solid (m.p. 243-246˚C). R_f 0.5 (CH_2Cl_2/EtOAc 3:2); $[\alpha]_D^{20}$+18.8 (c 0.5, $CHCl_3$).

^{1}H-NMR (400 MHz, $CDCl_3$) δ (ppm) 8.01-7.99 (m, 1H, aromatic), 7.82-7.60 (m, 1H, aromatic), 7.58-7.27 (m, 18H, aromatic), 5.68 (t, 1H, *J* = 9.7 Hz), 5.57 (s, 1H), 5.45 (s, 1H), 5.35 (dd, 1H, *J* = 8.0 Hz, *J* = 9.8 Hz), 4.97 (d, 1H, *J* = 8.0 Hz), 4.56 (d, 1H, *J* = 9.7 Hz), 4.44 (d, 1H, *J* = 3.1 Hz), 4.35 (dd, 1H, *J* = 1.3 Hz, *J* = 12.3 Hz), 4.29-4.20 (m, 3H), 4.07-4.00 (m, 2H), 3.70 (td, J = 3.6 Hz, J = 10.8 Hz), 3.56 (s, 1H), 3.49 (s, 1H), 2.93-2.84 (m, 1H), 2.77-2.68 (m, 1H), 2.57 (d, 1H, *J* = 11.1 Hz), 1.24 (t, 1H, *J* = 7.5 Hz). See Supplementary Figure 81.

^{13}C-NMR (100 MHz, $CDCl_3$) δ (ppm) 166.5, 164.9, 137.7-126.9, 101.4, 100.9, 100.1, 83.1, 77.2, 76.4, 76.3, 75.6, 72.6, 72.1, 70.4, 69.1, 68.8, 68.7, 66.7, 29.7, 22.7, 14.7. See Supplementary Figure 82.

IR (Nujol): ν (cm^{-1}) 3456, 3018, 2399, 2088, 1869, 1635, 1417, 1215, 1043, 925, 777, 514.

HRMS (ESI): m/z calcd for $C_{42}H_{42}SO_{12}$ [M+Na]+, 793.2295, found: 793.2295.

Ethyl-S-(2-deoxy-2-phthalimido-6-*O*-benzyl-β-D-glucopyranosyl)-(1→4)-(2-deoxy-2-phthalimido-6-*O*-benzyl-β-D-glucopyranoside 14ai (Method C)

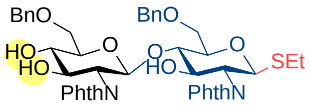

14ai

(28mg, 69% yield) as a colorless oil. R_f 0.3 (CH_2Cl_2/EtOAc 3:2); $[\alpha]_D^{20}$ +53.2 (c 0.2, $CHCl_3$).

^{1}H-NMR (400 MHz, $CDCl_3$) δ (ppm) 7.91-7.71 (m, 7H, aromatic), 7.33-7.08 (m, 11H, aromatic), 5.38 (d, 1H, *J* = 8.4 Hz), 5.23 (d, 1H, *J* = 10.5 Hz), 4.53-4.39 (m, 5H), 4.27-4.18 (m, 2H), 4.10 (s, 2H), 3.77-3.70 (m, 4H), 3.58-3.53 (m, 2H), 3.32 (s, 2H), 2.80 (d, 1H, *J* = 2.6 Hz), 2.67-2.55 (m, 2H), 2.43 (d, 1H, *J* = 4.4 Hz), 2.19 (d, 2H, *J* = 2.5 Hz), 1.16 (d, 1H, *J* = 7.4 Hz). See Supplementary Figure 83.

^{13}C-NMR (100 MHz, $CDCl_3$) δ (ppm) 168.1, 167.7, 138.2-123.2, 99.0, 81.7, 80.8, 78.0, 73.7, 73.3, 72.8, 71.3, 71.0, 69.8, 68.4, 56.2, 55.0, 53.8, 31.7, 29.7, 29.3, 23.6, 14.9. See Supplementary Figure 84.

IR (Nujol): ν (cm^{-1}) 3464, 2018, 2399, 2088, 1635, 1521, 1417, 1215, 1045, 927, 781, 669.

HRMS (ESI): m/z calcd for $C_{44}H_{44}N_2SO_{12}$ [M+H]+, 825.2693, found: 825.2699.

Ethyl-S-(2-deoxy-2-phthalimido-6-*O*-benzyl-β-D-glucopyranosyl)-(1→3)-(2-*O*-benzoyl-4,6-*O*-benzylidene)-β-D-galactopyranoside 15ai (Method C)

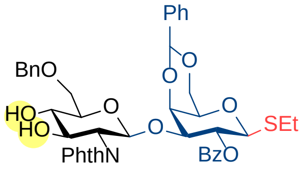

15ai

(27mg, 70% yield) as a colorless oil. R_f 0.2 (CH_2Cl_2/EtOAc 3:2)$[\alpha]_D^{20}$ -33.5 (c 0.2, $CHCl_3$).

^{1}H-NMR (400 MHz, $CDCl_3$) δ (ppm) 7.65-7.23 (m, 19H, aromatic), 5.50-5.42 (m, 3H), 4.63 (d, 1H, *J* = 11.8 Hz), 4.53 (d, 1H, *J* = 11.7 Hz), 4.43-4.41 (m, 2H), 4.25 (d, 1H, *J* = 12.2 Hz), 4.16-4.11 (m, 2H), 3.94 (dd, 1H, *J* = 3.4 Hz, *J* = 9.7 Hz), 3.85-3.81 (m, 2H), 3.78-3.74 (m, 1H), 3.70-3.65 (m, 1H), 3.52-3.50 (m, 1H), 3.43 (s, 1H), 2.96 (d, 1H, *J* = 2.5 Hz), 2.81-2.72 (m, 1H), 2.62-2.53 (m, 1H), 2.39 (br, 1H), 1.13 (t, 1H, *J* = 7.5 Hz). See Supplementary Figure 85.

^{13}C-NMR (100 MHz, $CDCl_3$) δ (ppm) 176.9, 167.9, 164.6, 137.8-123.0, 100.9, 99.5, 82.7, 79.8, 76.7, 76.1, 73.9, 73.7, 73.4, 71.8, 70.7, 70.2, 69.1, 68.3, 56.1, 29.5, 22.5, 14.7. See Supplementary Figure 86.

IR (Nujol): ν (cm^{-1}) 3541, 2018, 2399, 2065, 1772, 1712, 1635, 1388, 1215, 1045, 908, 771, 667.

HRMS (ESI): m/z calcd for $C_{43}H_{43}NSO_{12}$ $[M+H]^+$, 798.2584, found: 798.2584.

Ethyl-S-((2-*O*-benzoyl-4,6-*O*-benzylidene)-β-D-galactopyranosyl)-(1→4)-2-deoxy-2-phthalimido-6-*O*-benzyl-β-D-glucopyranoside 16ai (Method C)

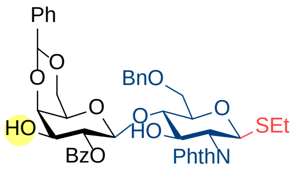

16ai

(26mg, 67% yield) as a colorless oil. R_f 0.5 (CH_2Cl_2/EtOAc 3:2); $[\alpha]_D^{20}$ -25.8 (c 0.2, $CHCl_3$).

^{1}H-NMR (400 MHz, $CDCl_3$) δ (ppm) 8.09-7.28 (m, 19H, aromatic), 5.54 (s, 1H), 5.39 (t, 1H, *J* = 8.4 Hz), 5.32 (d, 1H, *J* = 10.5 Hz), 4.68 (d, 1H, *J* = 8.0 Hz), 4.53-4.46 (m, 2H), 4.32-4.24 (m, 5H), 4.05 (d, 1H, *J* = 12.2 Hz), 3.87-3.79 (m, 2H), 3.65-3.55 (m, 4H), 2.72-2.56 (m, 3H), 1.20 (t, 1H, *J* = 7.4 Hz). See Supplementary Figure 87.

^{13}C-NMR (100 MHz, $CDCl_3$) δ (ppm) 168.1, 167.9, 165.7, 138.5-123.3, 101.5, 101.2, 81.3, 81.1, 78.1, 77.2, 75.2, 73.2, 72.6, 71.5, 70.6, 68.5, 68.2, 66.8, 55.3, 24.1, 14.9. See Supplementary Figure 88.

IR (Nujol): ν (cm^{-1}) 3498, 3018, 2399, 2065, 1770, 1635, 1386, 1217, 1045, 927, 771, 667, 514.

HRMS (ESI): m/z calcd for $C_{43}H_{43}NSO_{12}$ $[M+H]^+$, 798.2584, found: 798.2588.

Phenyl-S-((2-*O*-benzoyl-4,6-*O*-benzylidene)-β-D-galactopyranosyl)-(1→4)-(2-deoxy-2-phthalimido-6-*O*-benzyl-β-D-glucopyranosyl)-(1→2)-3,4,6-tri-*O*-benzyl)-α-D-mannopyranoside 17ai (Method C, E)

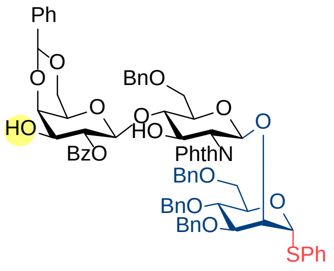

17ai

(19mg, 56% yield) as a colorless oil. R_f 0.4 (CH_2Cl_2/EtOAc 4:1); $[\alpha]_D^{20}$ +40.5 (c 0.1, $CHCl_3$).

^{1}H-NMR (500 MHz, $CDCl_3$) δ (ppm) 8.13-6.95 (m, 39H, aromatic), 5.48 (s, 1H), 5.45 (d, 1H, *J* = 8.5 Hz), 5.35 (s, 1H), 5.08 (dd, 1H, *J* = 3.6 Hz, *J* = 10.1 Hz), 4.85-4.26 (m, 13H), 4.14 (t, 1H, *J* = 9.9 Hz), 4.08-4.00 (m, 4H), 3.93-3.77 (m, 4H), 3.71 (t, 1H, *J* = 8.9 Hz), 3.64-3.60 (m, 2H), 3.49 (d, 1H, *J* = 10.8 Hz), 3.28 (s, 1H), 3.11 (dd, 1H, *J* = 6.7 Hz, *J* = 10.7 Hz), 2.20 (s, 1H). See Supplementary Figure 89.

^{13}C-NMR (125 MHz, $CDCl_3$) δ (ppm) 169.7, 169.1, 165.0, 138.3-123.0, 101.0, 100.4, 96.8, 84.6, 83.4, 81.2, 78.2, 77.1, 75.7, 74.9, 74.8, 74.2, 73.7, 73.4, 72.9, 72.5, 72.3, 71.5, 71.2, 70.9, 70.3, 69.7, 69.4, 55.9. See Supplementary Figure 90.

IR (Nujol): ν (cm^{-1}) 3498, 3018, 2980, 2399, 2065, 1770, 1635, 1215, 1045, 927, 771, 667.

HRMS (ESI): m/z calcd for $C_{74}H_{71}NSO_{17}$ $[M+Na]^+$, 1300.4340, found: 1300.4345.

Phenyl-S-(2-deoxy-2-phthalimido-6-*O*-benzyl-β-D-glucopyranoside)-(1→2)-3,4,6-tri-*O*-benzyl)-α-D-mannopyranoside 18ai (Method C)

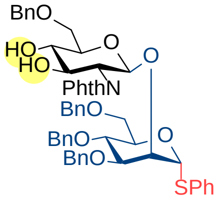

18ai

(31mg, 69% yield) as a colorless oil. R_f 0.3 (CH_2Cl_2/EtOAc 3:2); $[\alpha]_D^{20}$ +86.8 (c 1.0, $CHCl_3$).

^{1}H-NMR (400 MHz, $CDCl_3$) δ (ppm) 7.67-7.07 (m, 29H), 5.39 (d, 1H, *J* = 8.0 Hz), 5.30 (d, 1H, *J* = 2.0 Hz), 4.83 (dd, 1H, *J* = 10.9 Hz), 4.78 (d, 1H, *J* = 11.6 Hz), 4.66-4.58 (m, 3H), 4.43-4.31 (m, 4H), 4.27 (t, 1H, *J* = 2.4 Hz), 4.09-3.98 (m, 3H), 3.87-3.58 (m, 6H), 3.47 (dd, 1H, *J* = 1.5 Hz, *J* = 10.9 Hz), 3.29 (br, 1H), 3.11 (dd, 1H, *J* = 6.8 Hz, *J* = 10.9 Hz). See Supplementary Figure 91.

^{13}C-NMR (100 MHz, $CDCl_3$) δ (ppm) 169.6, 168.7, 138.6-123.3, 97.1, 84.9, 78.5, 77.4, 76.1, 75.3, 75.1, 74.5, 74.1, 73.7, 72.7, 72.6, 71.8, 71.5, 71.2, 70.0, 69.7, 56.3. See Supplementary Figure 92.

IR (Nujol): ν (cm^{-1}) 3456, 3018, 2980, 2399, 2065, 1635, 1419, 1215, 1045, 927, 771, 669.

HRMS (ESI): m/z calcd for $C_{54}H_{53}NSO_{11}$ $[M+Na]^+$, 946.3237, found: 946.3241.

	Parameter	Value
1	Solvent	$CDCl_3$
2	Spectrometer Frequency	500 MHz
3	Nucleus	^{1}H

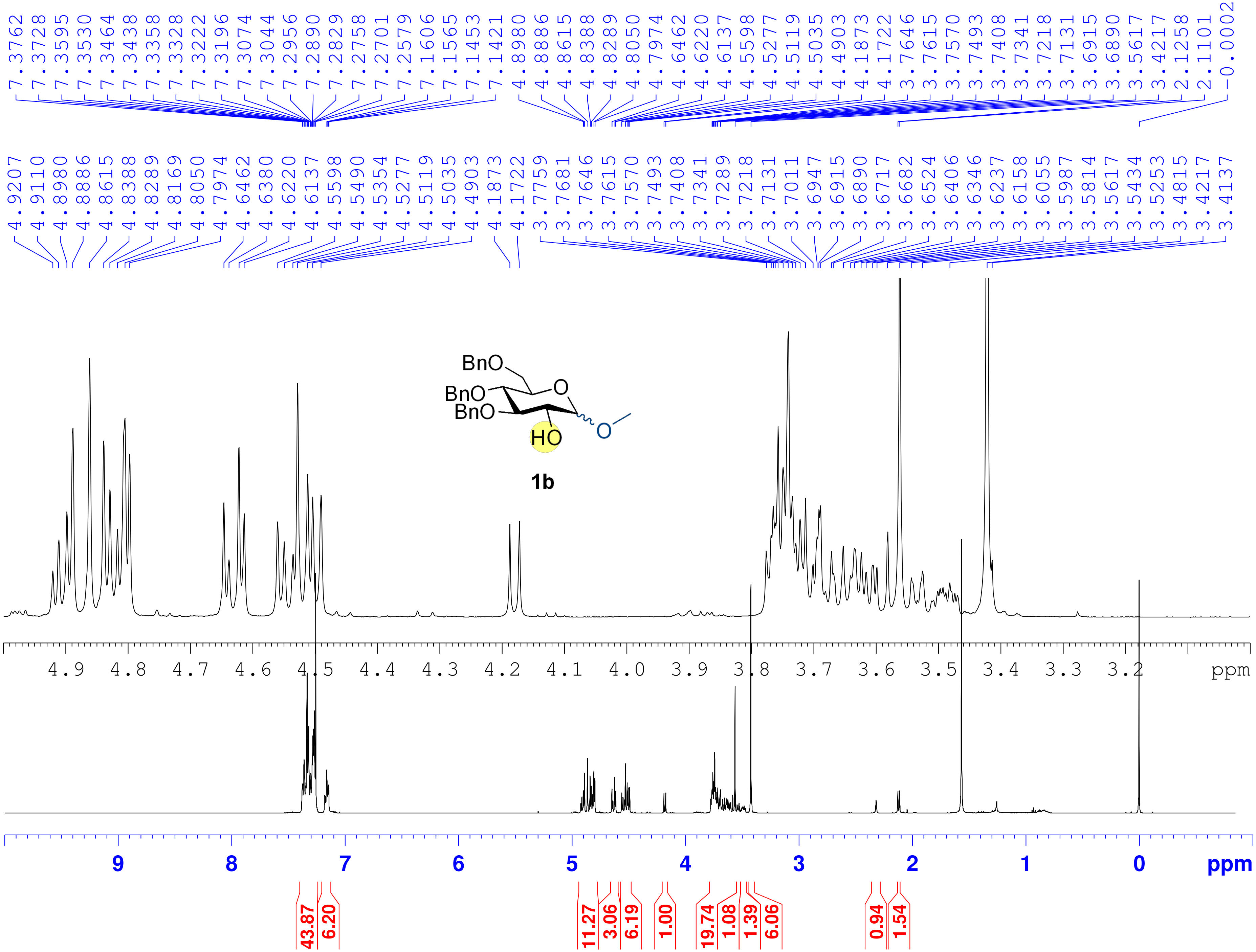

Supplementary Figure 1. 1H-NMR spectrum of compound 1b

	Parameter	Value
1	Solvent	$CDCl_3$
2	Spectrometer Frequency	125 MHz
3	Nucleus	^{13}C

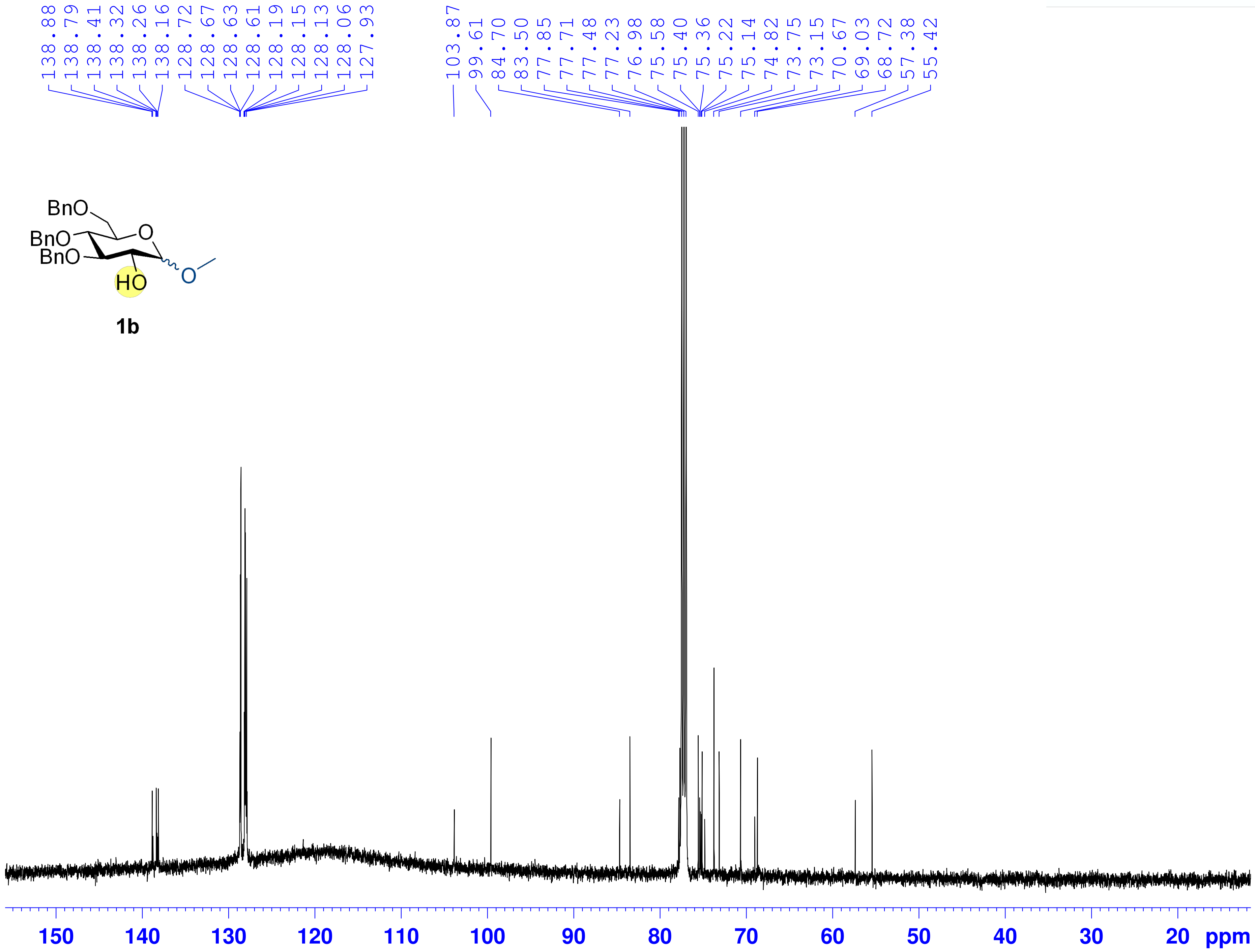

Supplementary Figure 2. 13C-NMR spectrum of compound 1b

	Parameter	Value
1	Solvent	$CDCl_3$
2	Spectrometer Frequency	500 MHz
3	Nucleus	^{1}H

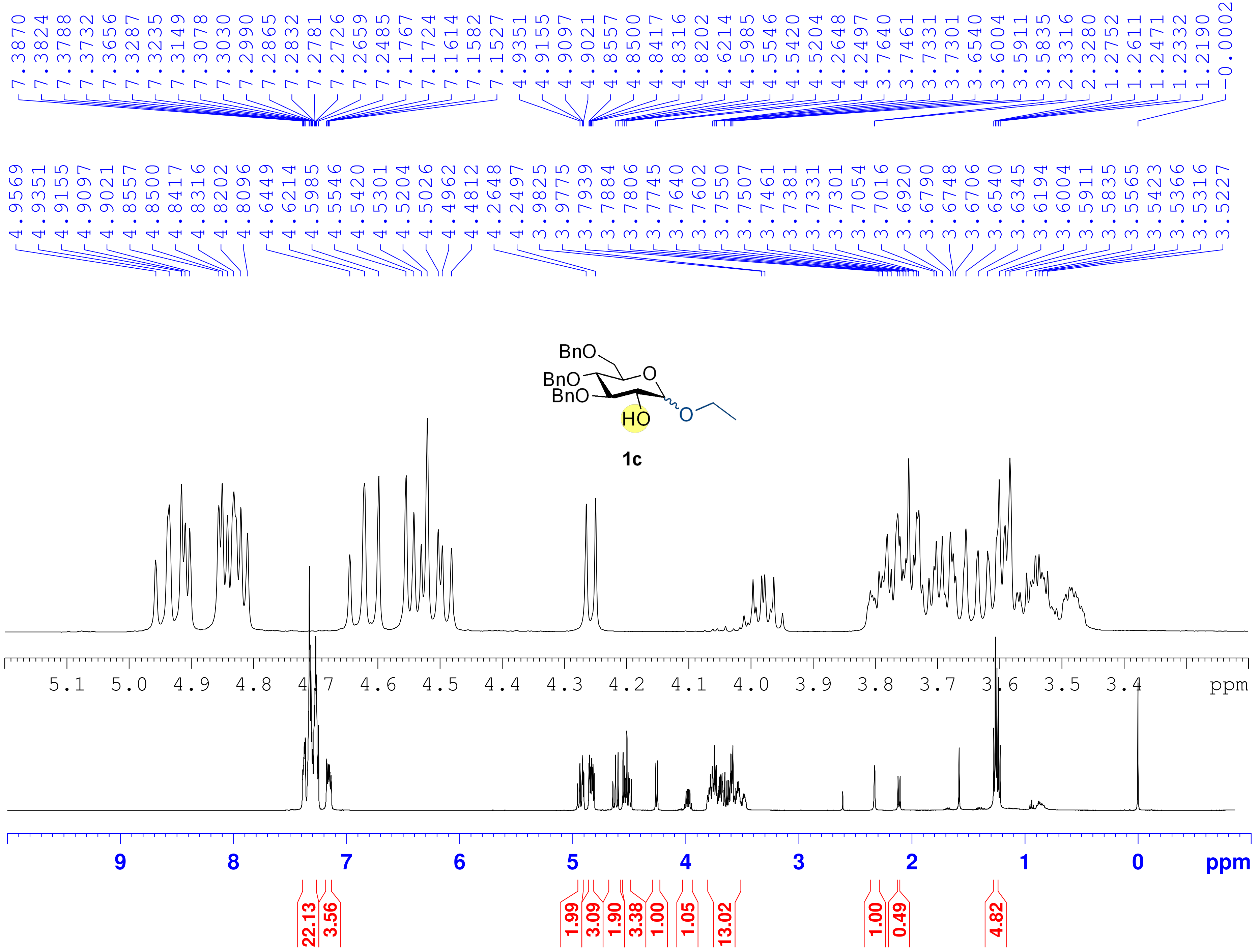

Supplementary Figure 3. 1H-NMR spectrum of compound 1c

	Parameter	Value
1	Solvent	$CDCl_3$
2	Spectrometer Frequency	125 MHz
3	Nucleus	^{13}C

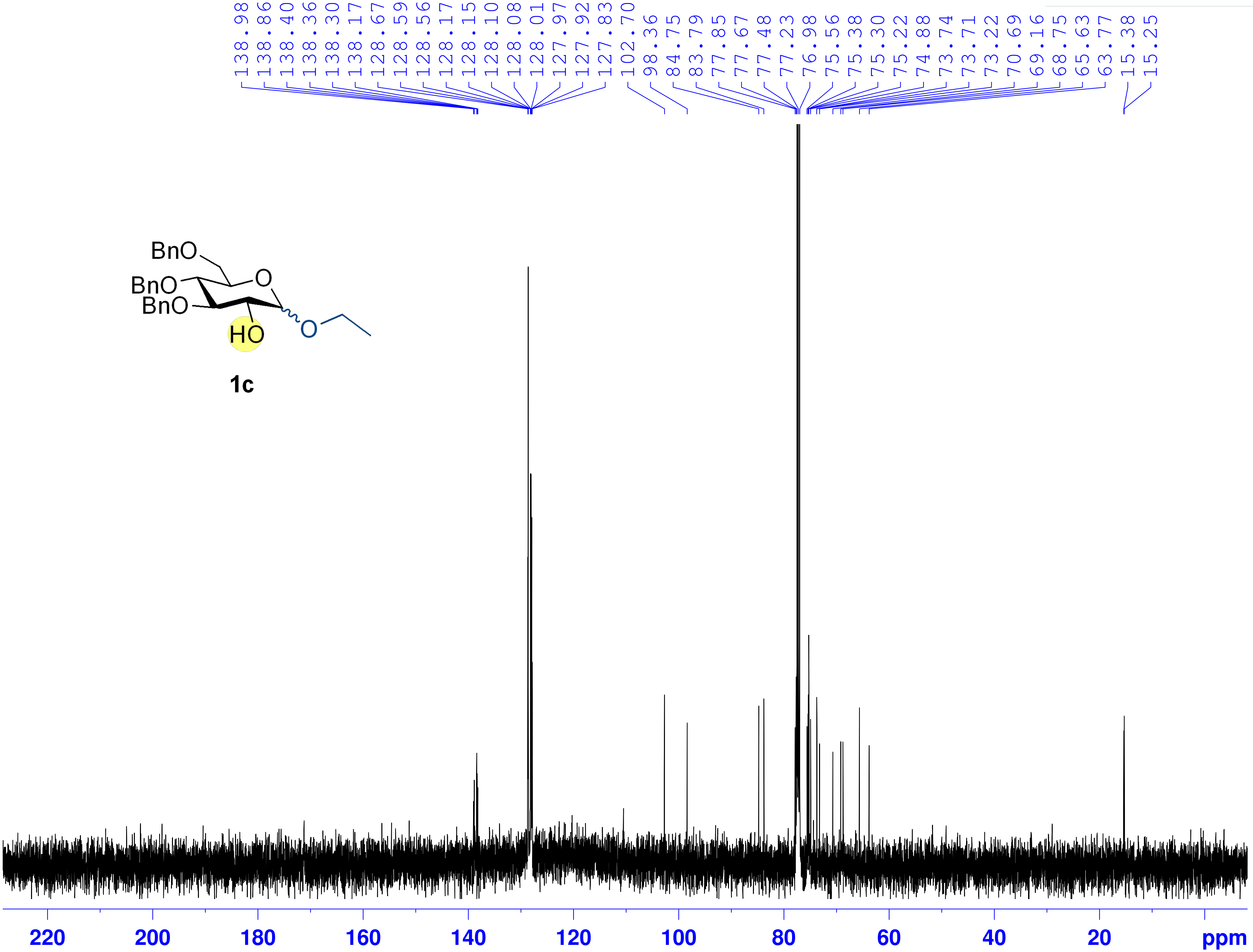

Supplementary Figure 4. 13C-NMR spectrum of compound 1c

	Parameter	Value
1	Solvent	$CDCl_3$
2	Spectrometer Frequency	500 MHz
3	Nucleus	^{1}H

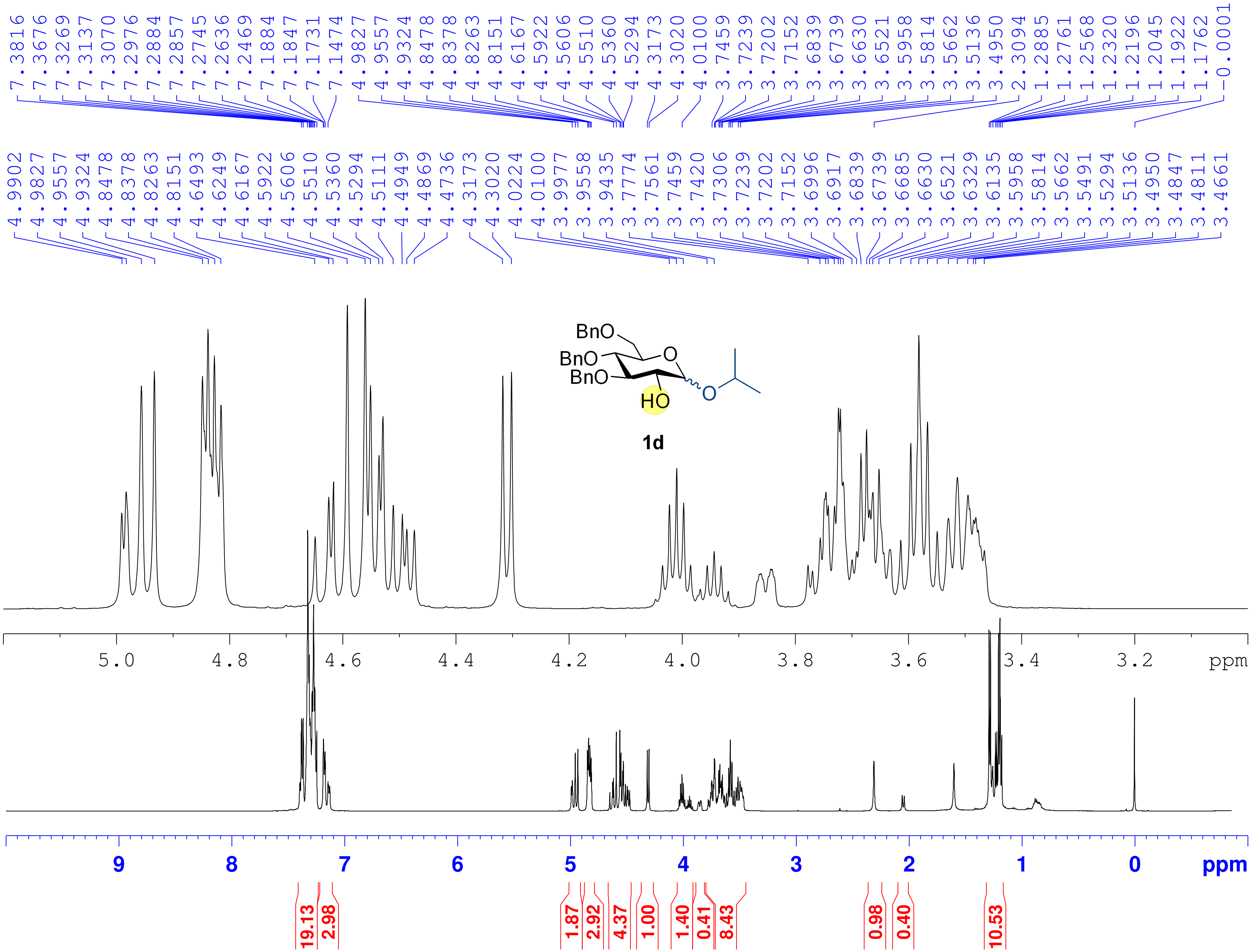

Supplementary Figure 5. 1H-NMR spectrum of compound 1d

	Parameter	Value
1	Solvent	$CDCl_3$
2	Spectrometer Frequency	125 MHz
3	Nucleus	^{13}C

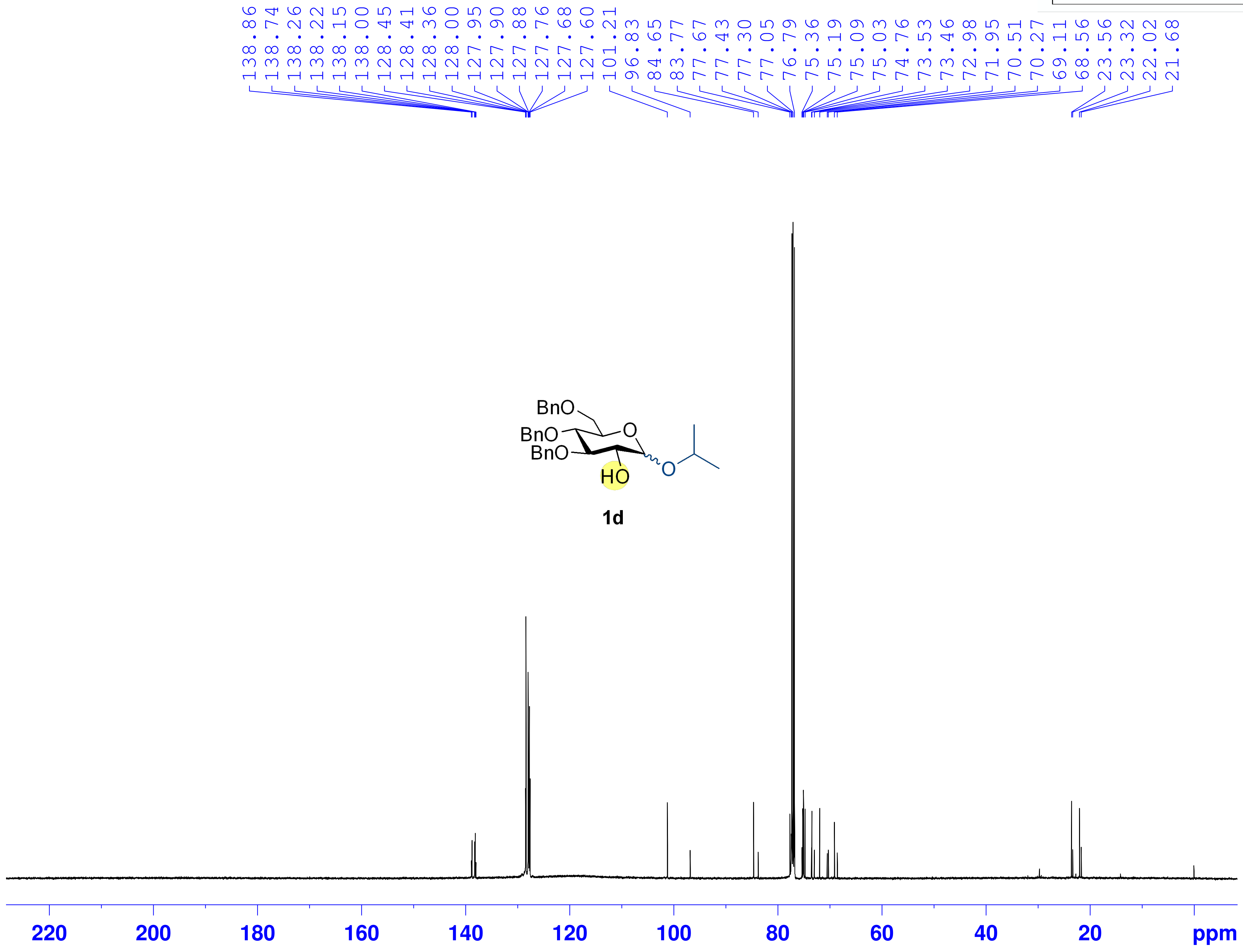

Supplementary Figure 6. 13C-NMR spectrum of compound 1d

	Parameter	Value
1	Solvent	$CDCl_3$
2	Spectrometer Frequency	500 MHz
3	Nucleus	^{1}H

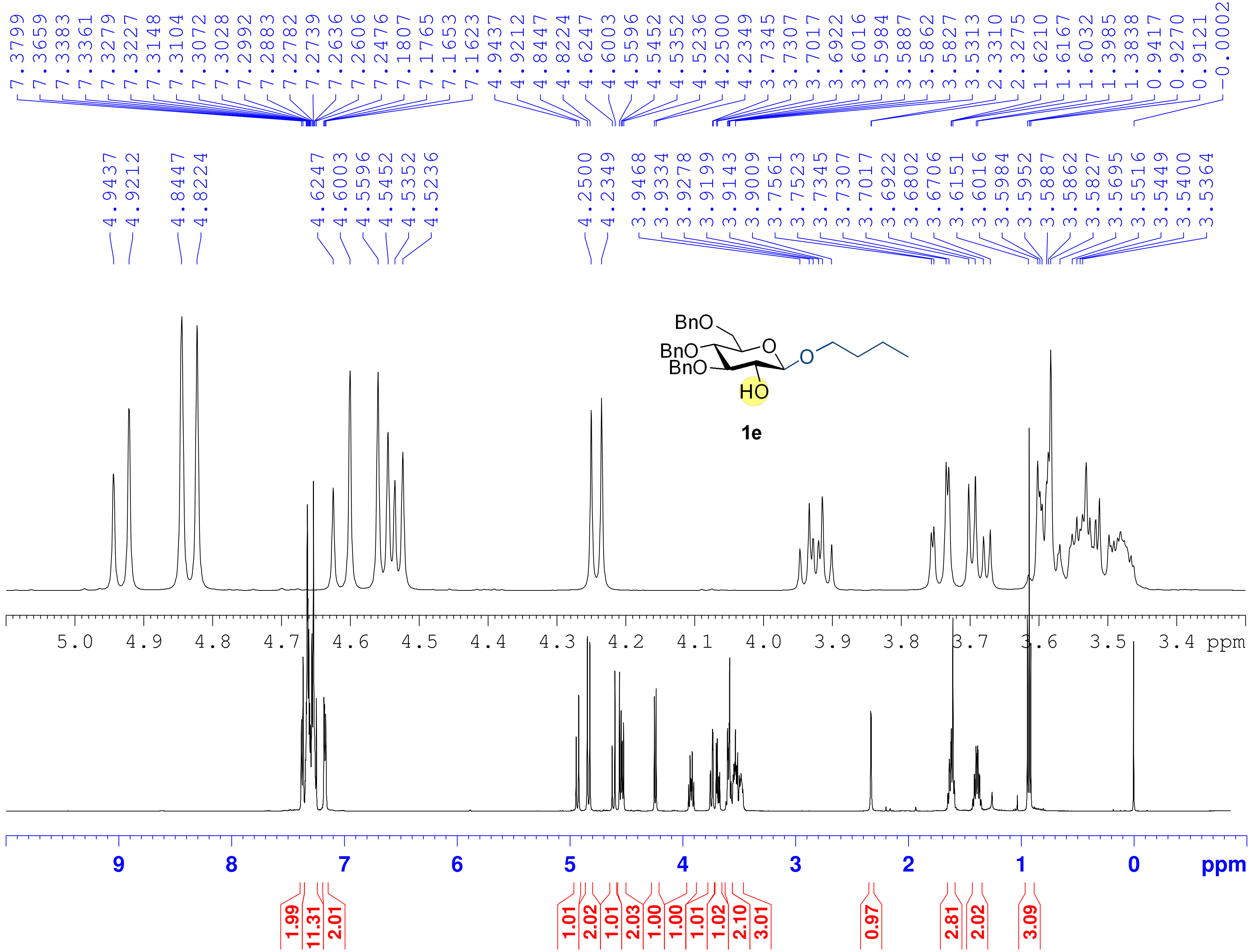

Supplementary Figure 7. 1H-NMR spectrum of compound 1e

	Parameter	Value
1	Solvent	$CDCl_3$
2	Spectrometer Frequency	125 MHz
3	Nucleus	^{13}C

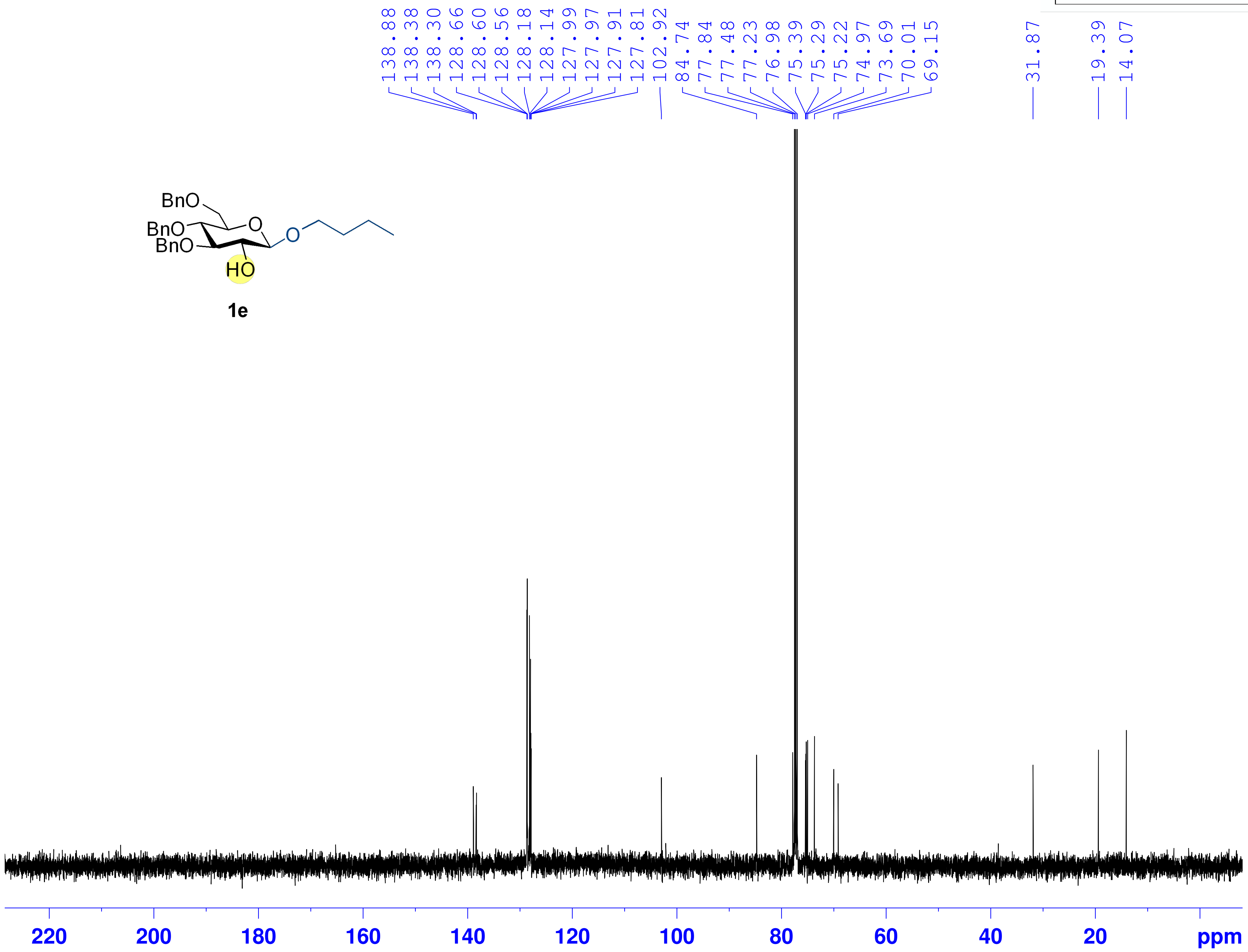

Supplementary Figure 8. 13C-NMR spectrum of compound 1e

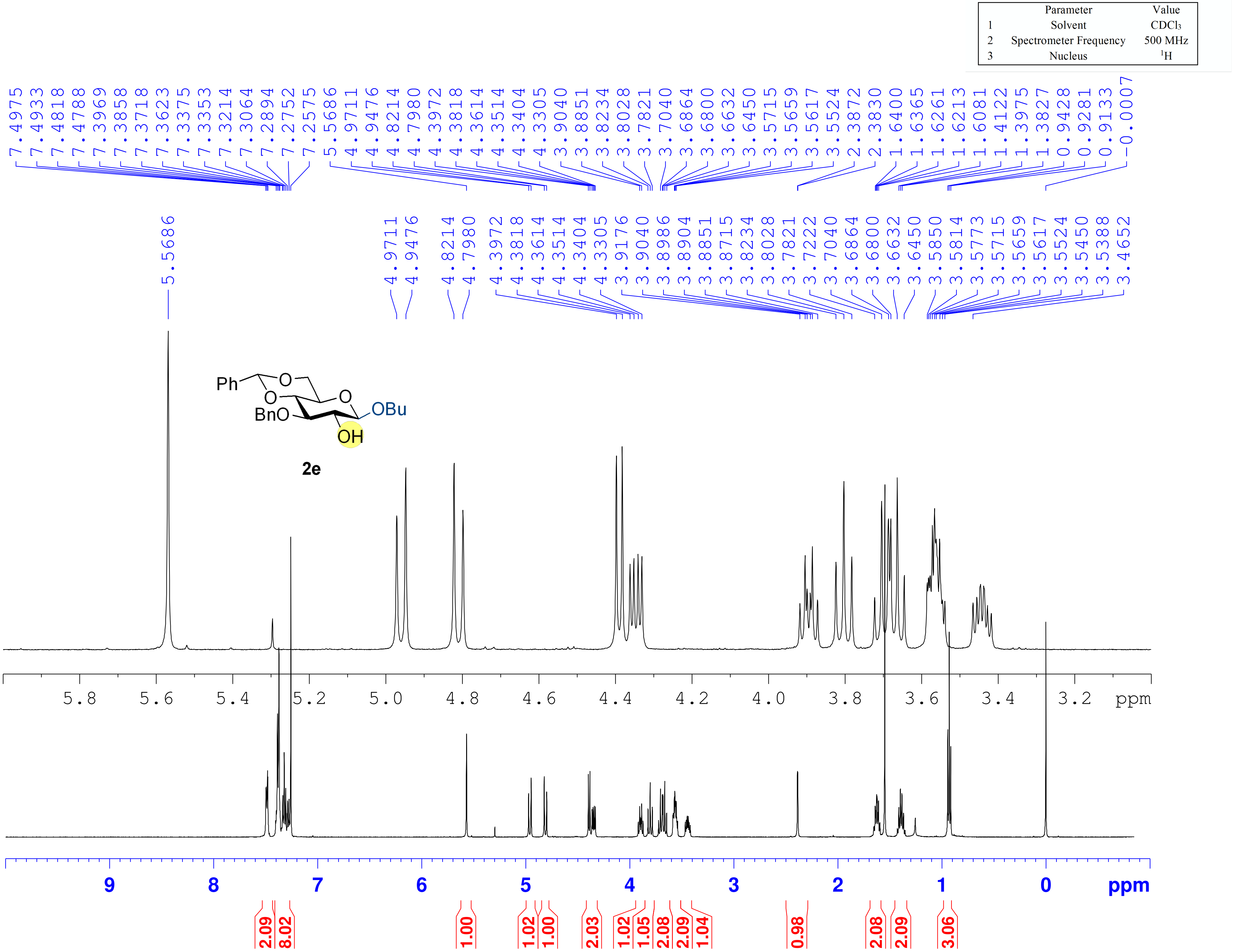

Supplementary Figure 9. 1H-NMR spectrum of compound 2e

	Parameter	Value
1	Solvent	$CDCl_3$
2	Spectrometer Frequency	125 MHz
3	Nucleus	^{13}C

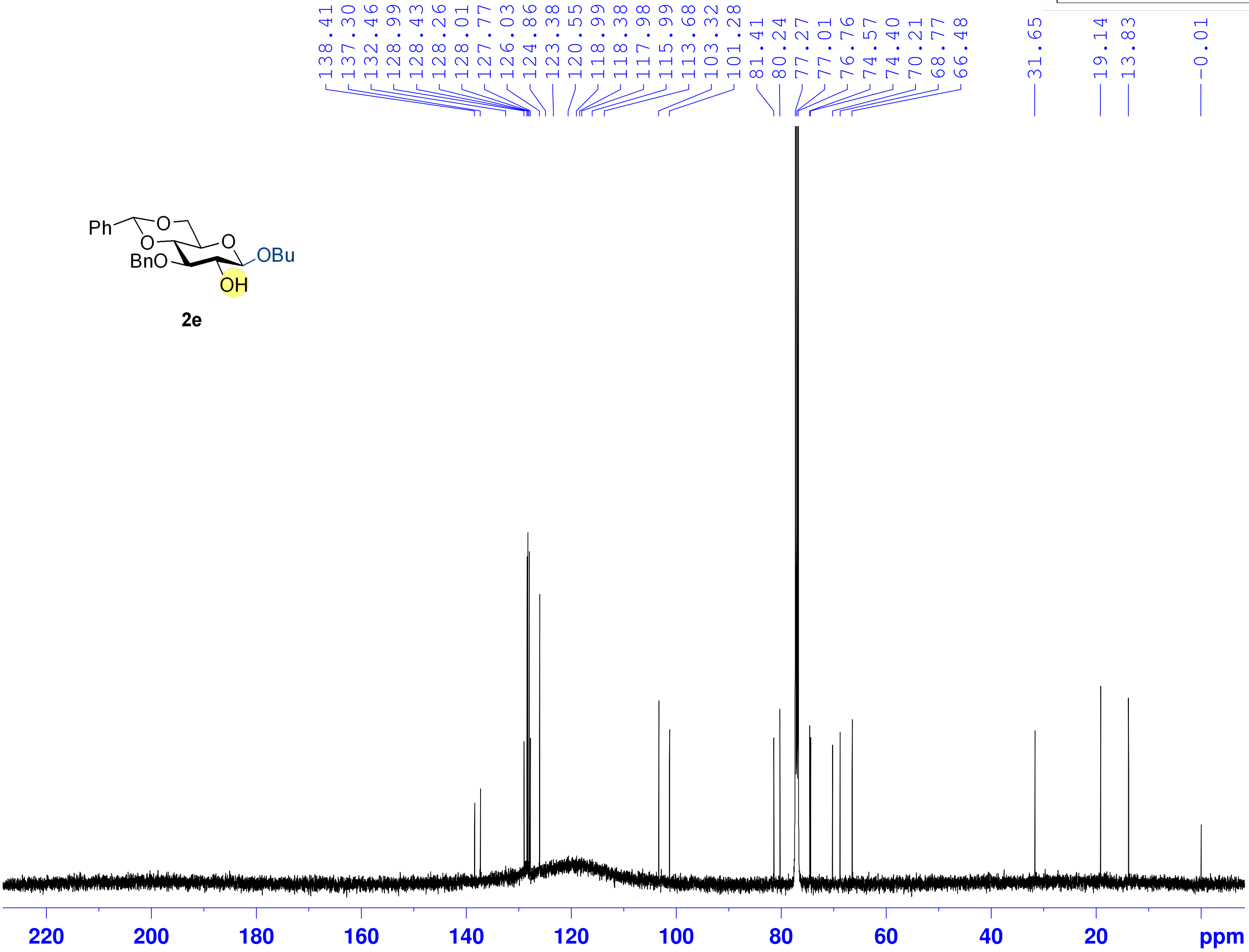

Supplementary Figure 10. 13C-NMR spectrum of compound 2e

	Parameter	Value
1	Solvent	$CDCl_3$
2	Spectrometer Frequency	500 MHz
3	Nucleus	^1H

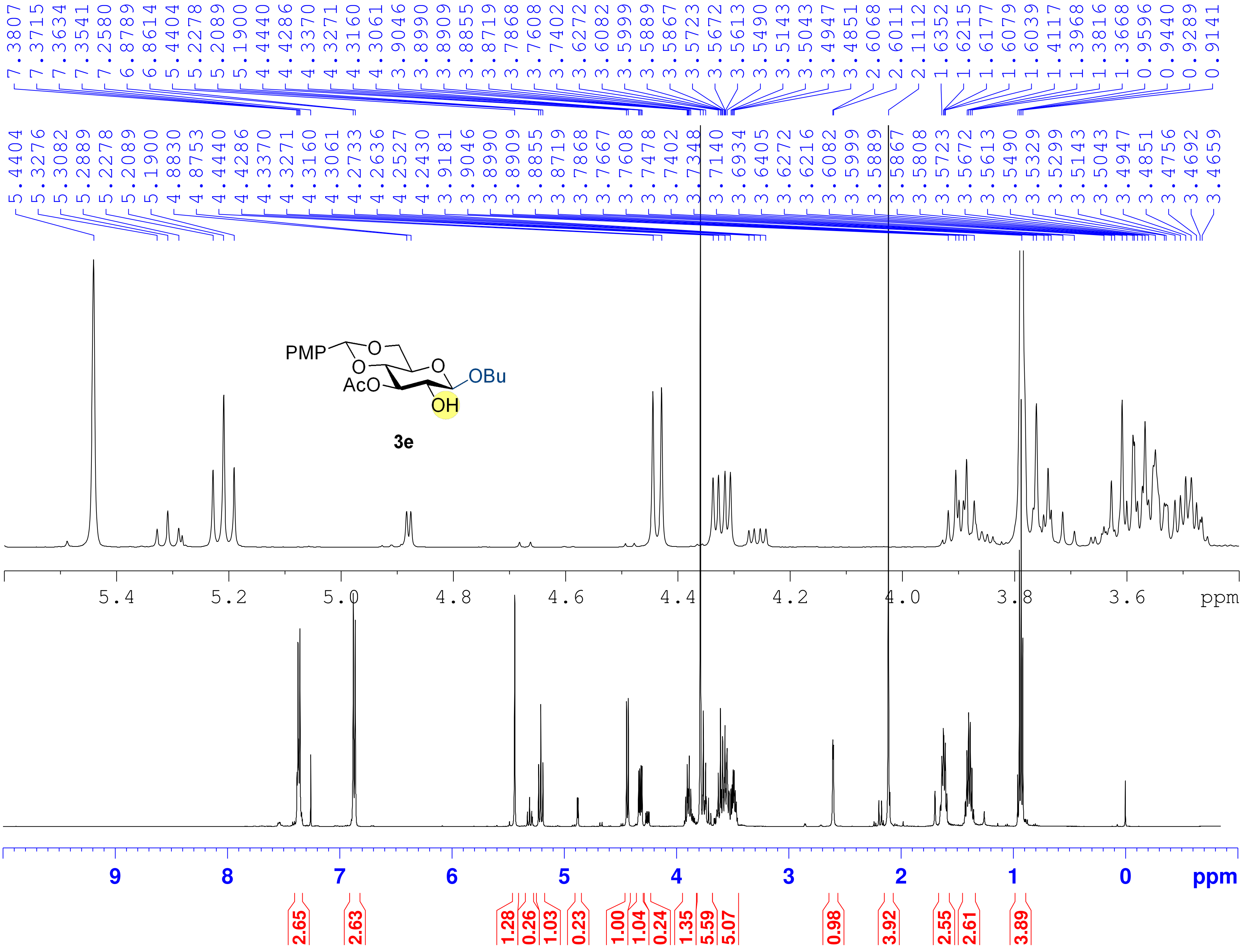

Supplementary Figure 11. 1H-NMR spectrum of compound 3e

	Parameter	Value
1	Solvent	$CDCl_3$
2	Spectrometer Frequency	125 MHz
3	Nucleus	^{13}C

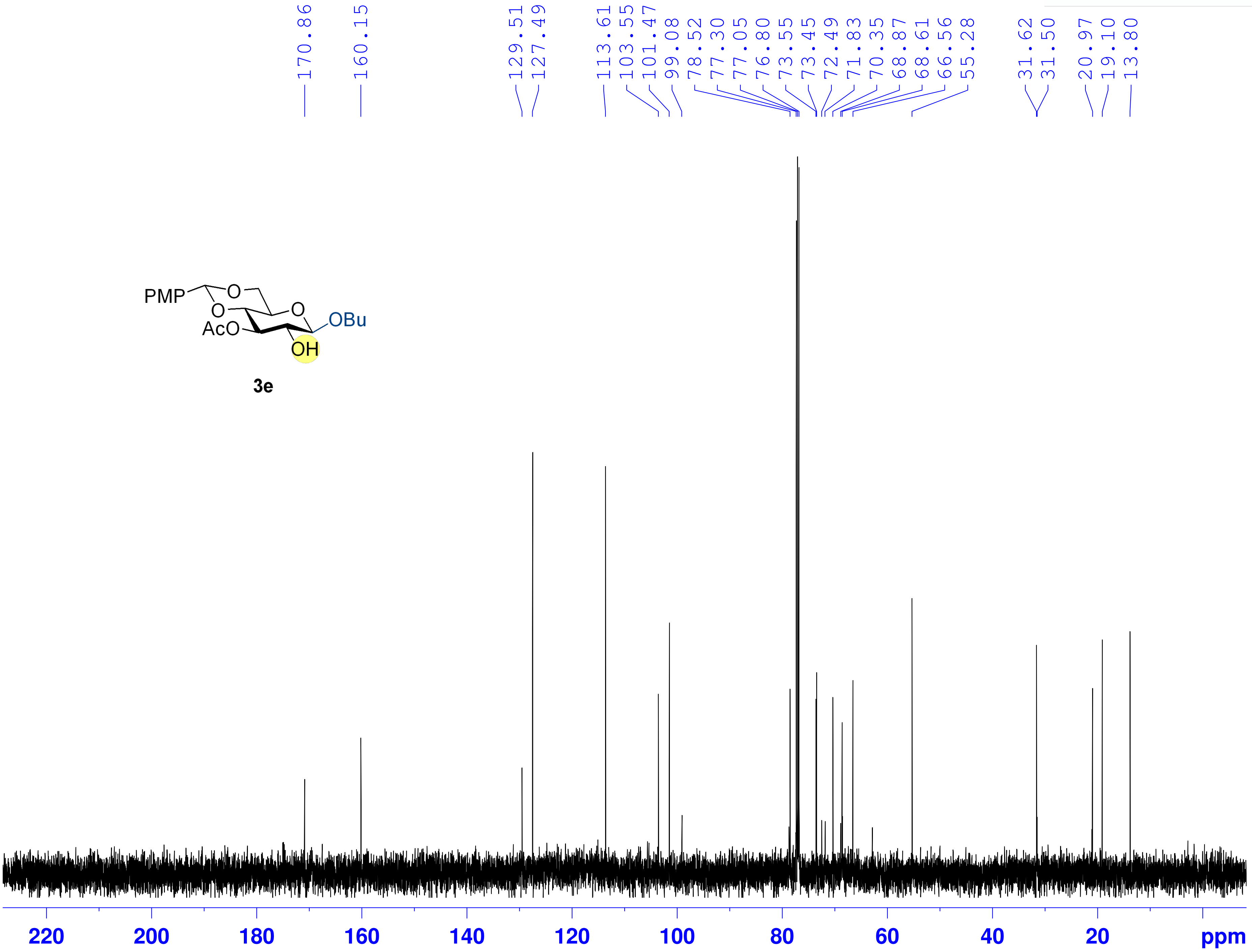

Supplementary Figure 12. 13C-NMR spectrum of compound 3e

	Parameter	Value
1	Solvent	$CDCl_3$
2	Spectrometer Frequency	500 MHz
3	Nucleus	^{1}H

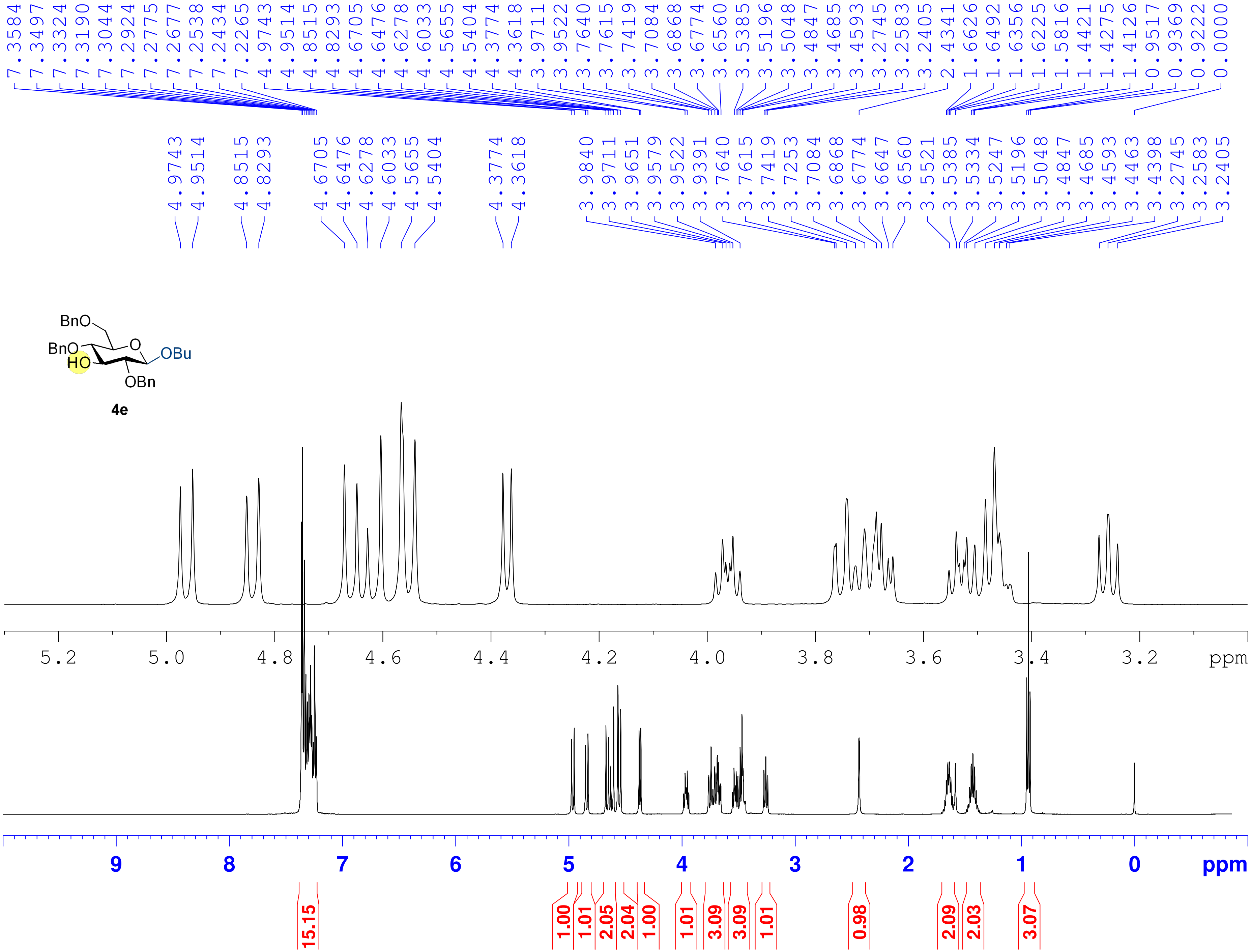

Supplementary Figure 13. 1H-NMR spectrum of compound 4e

	Parameter	Value
1	Solvent	$CDCl_3$
2	Spectrometer Frequency	125 MHz
3	Nucleus	^{13}C

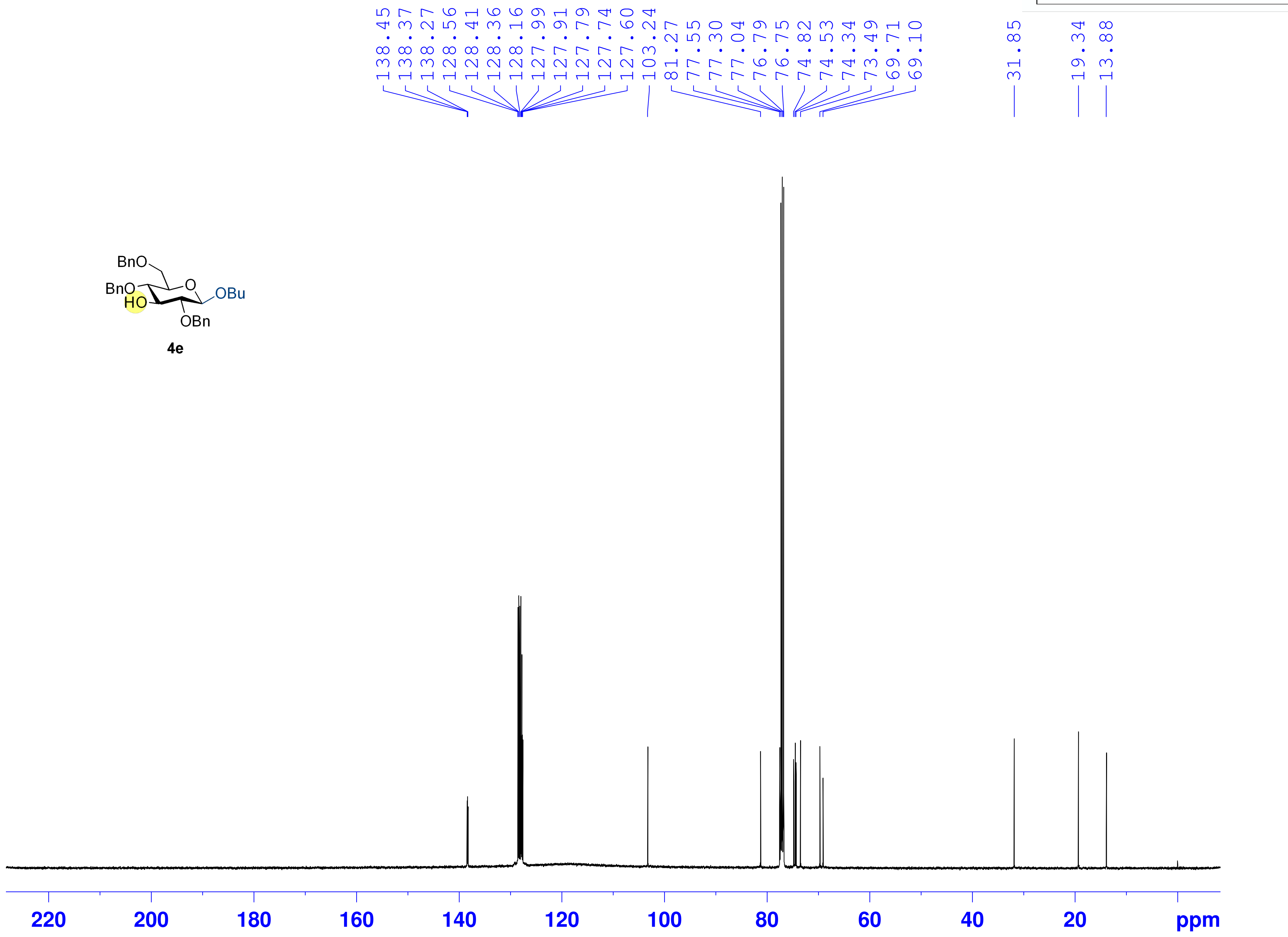

Supplementary Figure 14. 13C-NMR spectrum of compound 4e

	Parameter	Value
1	Solvent	$CDCl_3$
2	Spectrometer Frequency	500 MHz
3	Nucleus	^1H

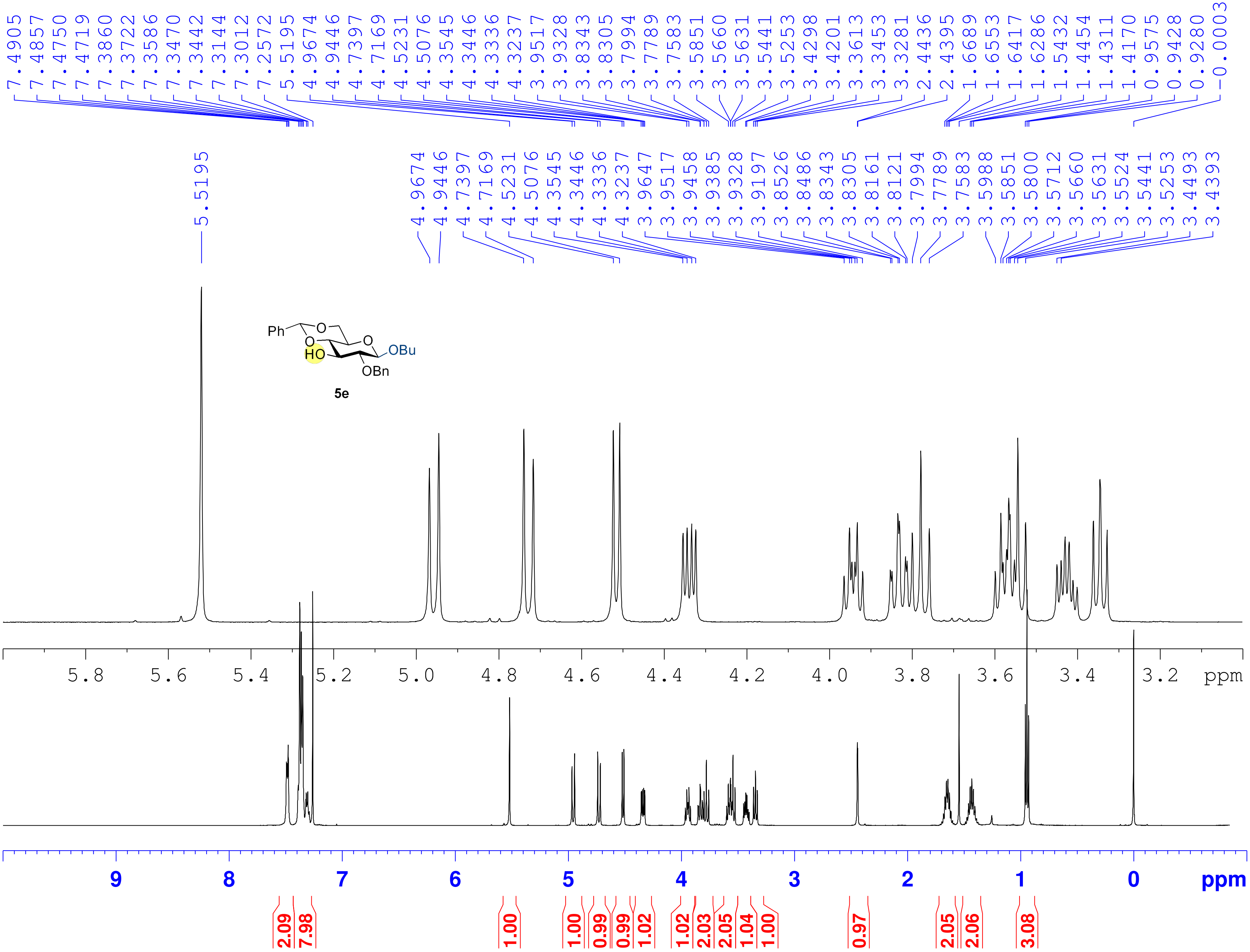

Supplementary Figure 15. 1H-NMR spectrum of compound 5e

	Parameter	Value
1	Solvent	$CDCl_3$
2	Spectrometer Frequency	125 MHz
3	Nucleus	^{13}C

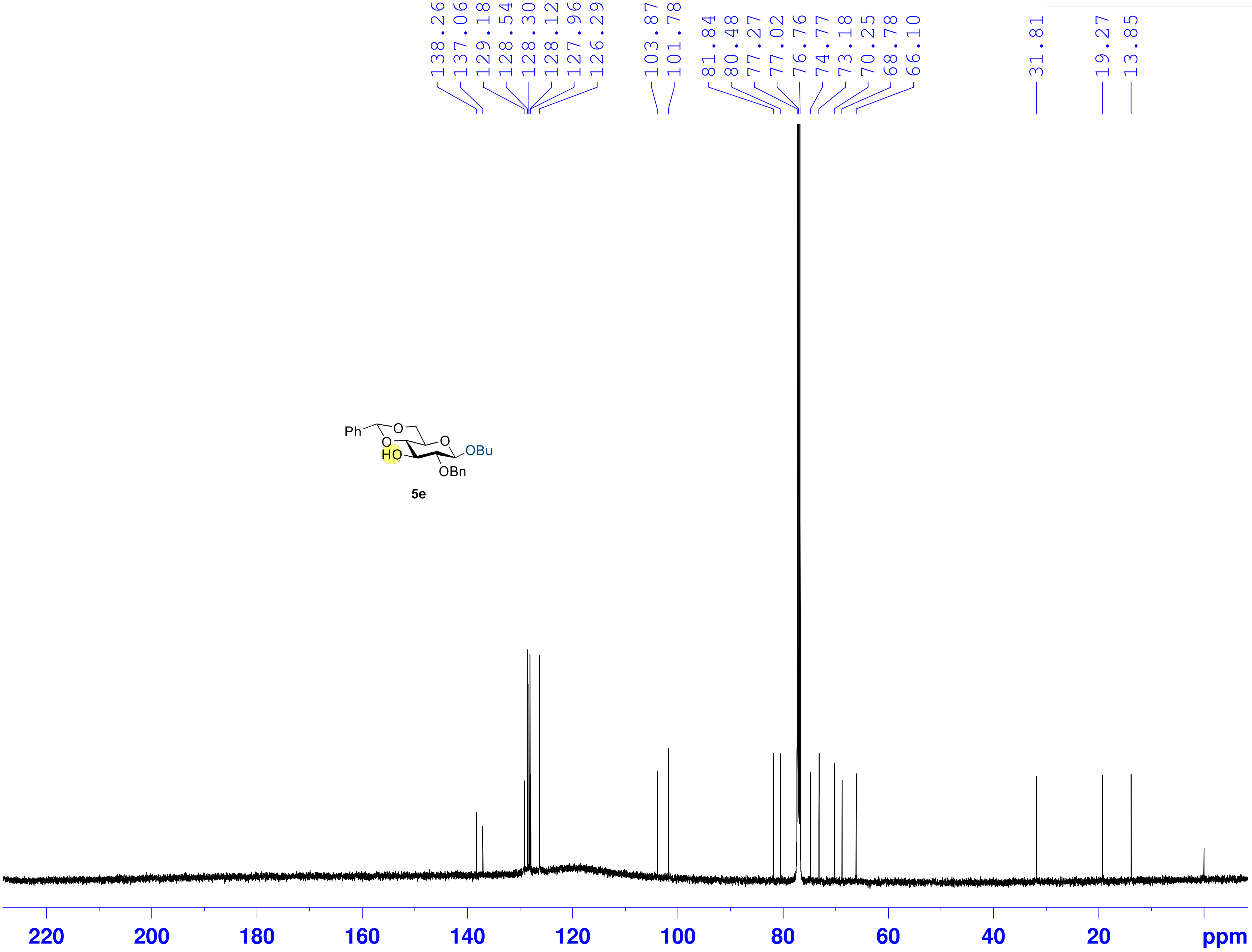

Supplementary Figure 16. 13C-NMR spectrum of compound 5e

	Parameter	Value
1	Solvent	$CDCl_3$
2	Spectrometer Frequency	500 MHz
3	Nucleus	^{1}H

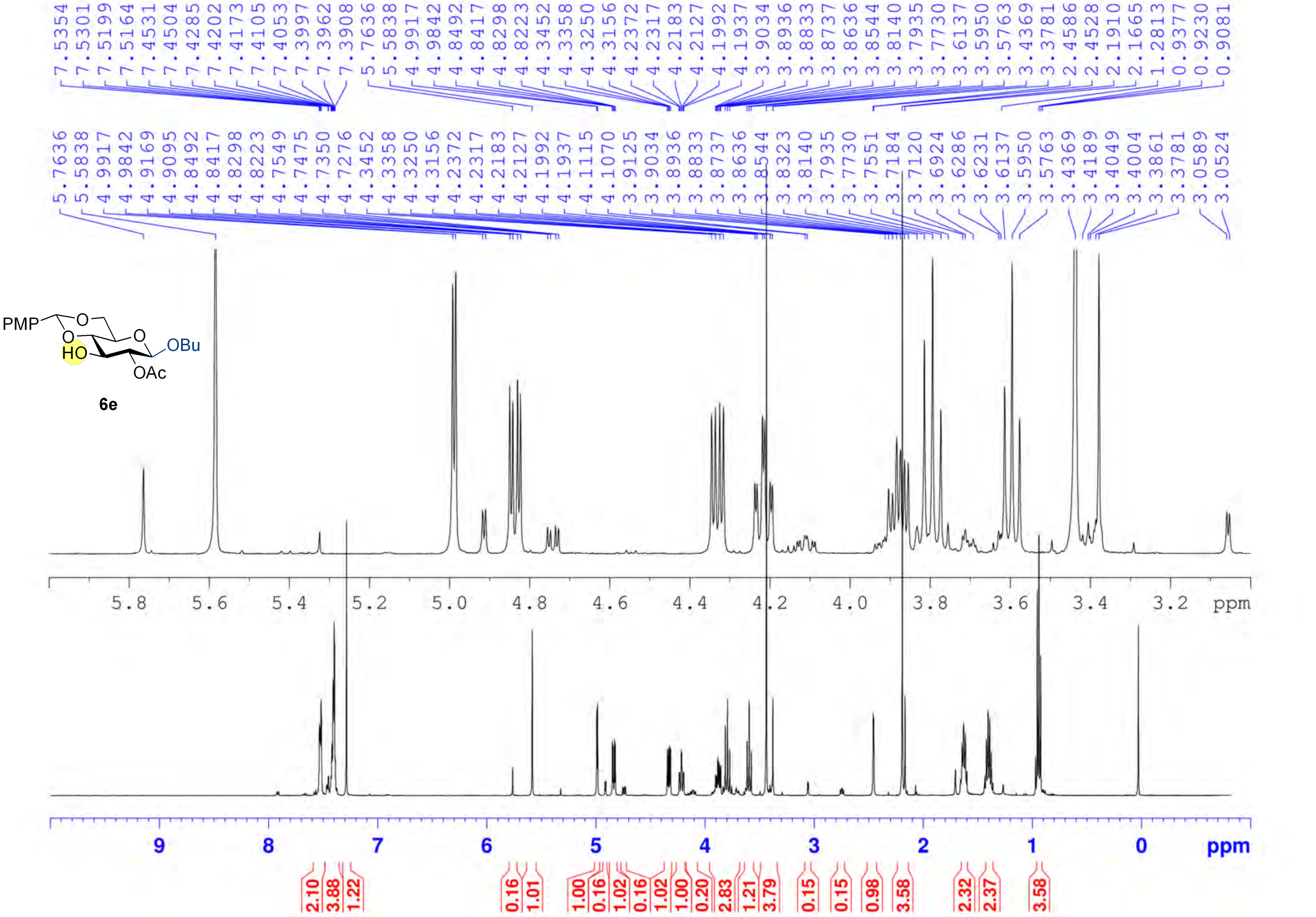

Supplementary Figure 17. 1H-NMR spectrum of compound 6e

	Parameter	Value
1	Solvent	$CDCl_3$
2	Spectrometer Frequency	125 MHz
3	Nucleus	^{13}C

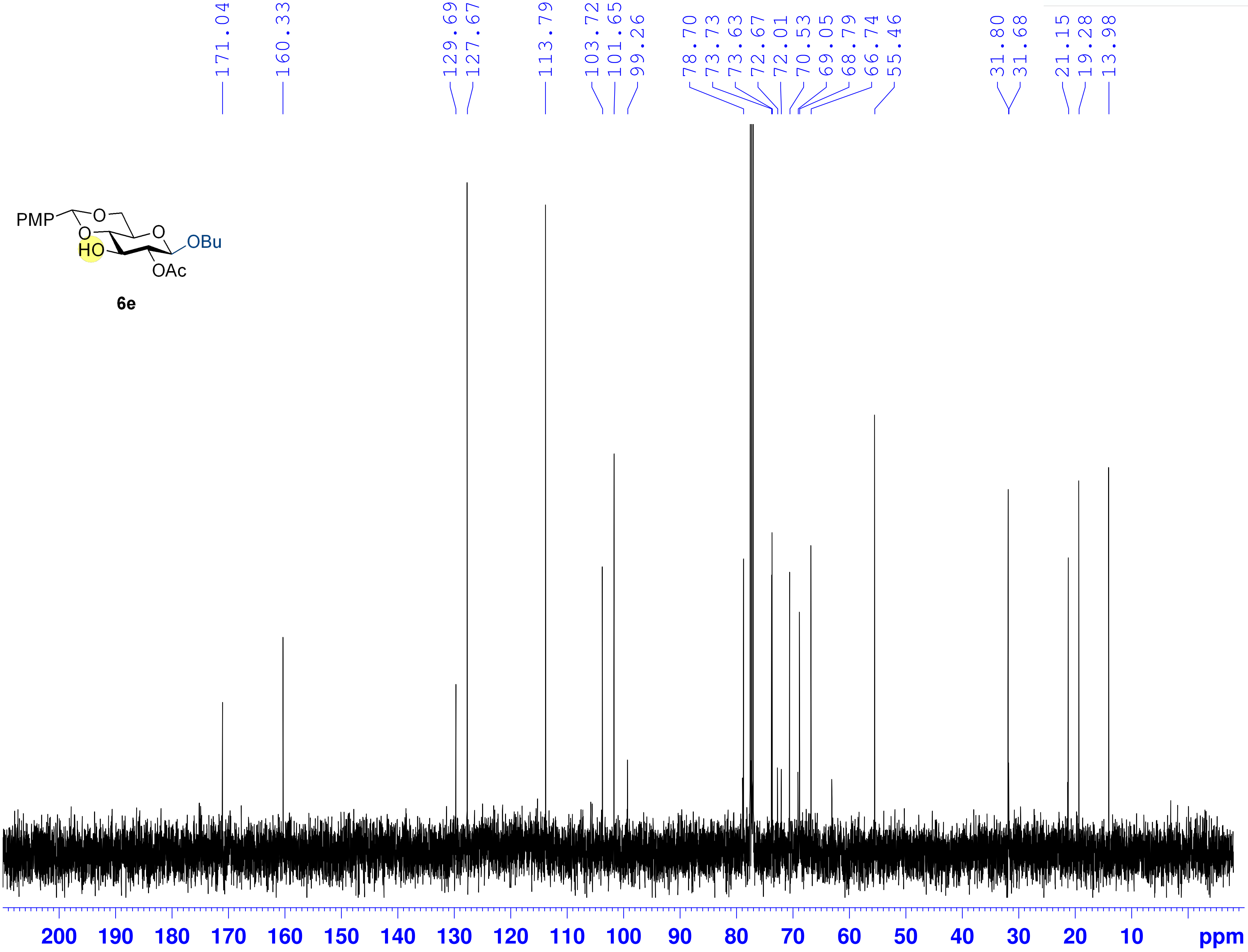

Supplementary Figure 18. 13C-NMR spectrum of compound 6e

	Parameter	Value
1	Solvent	$CDCl_3$
2	Spectrometer Frequency	500 MHz
3	Nucleus	^1H

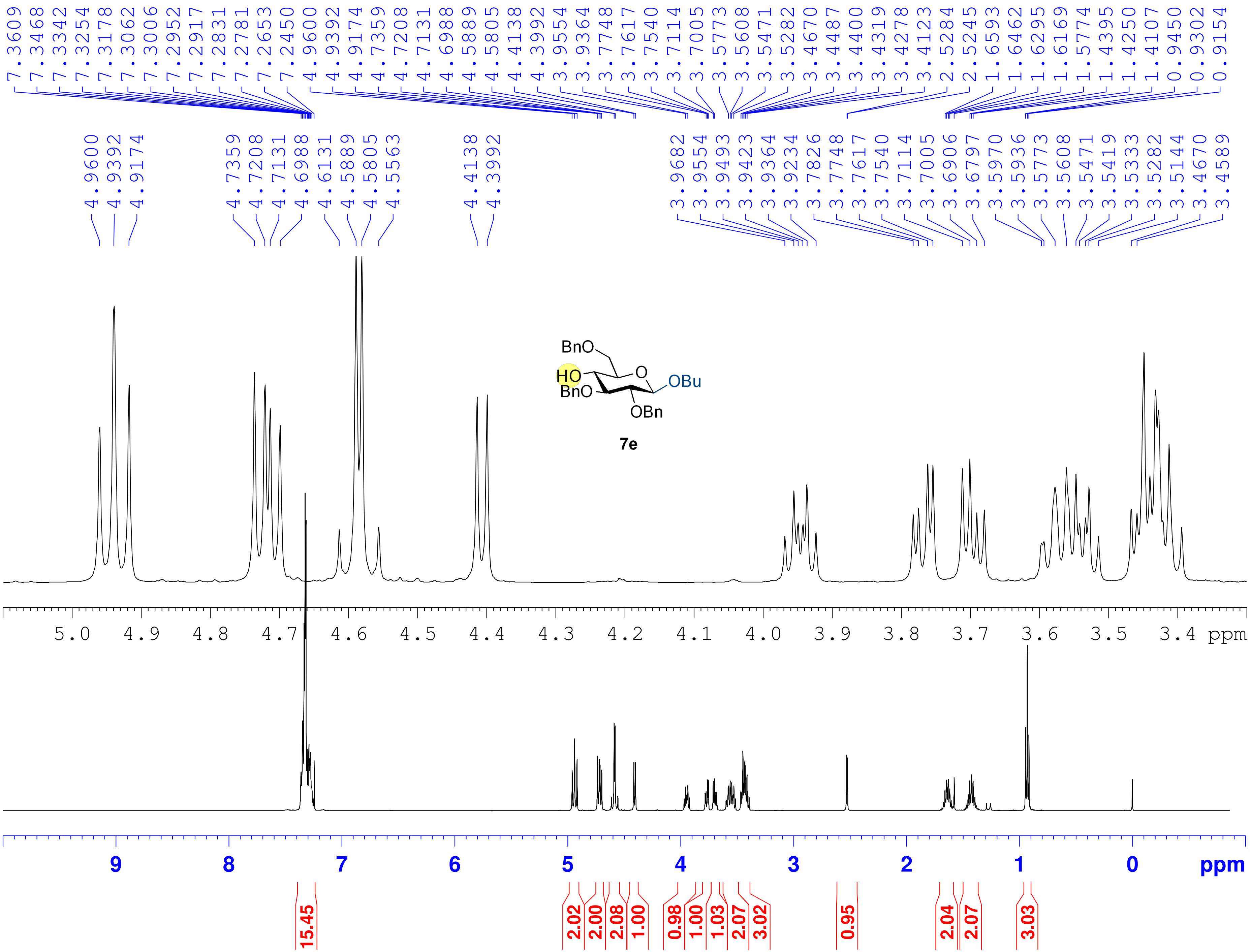

Supplementary Figure 19. 1H-NMR spectrum of compound 7e

	Parameter	Value
1	Solvent	$CDCl_3$
2	Spectrometer Frequency	125 MHz
3	Nucleus	^{13}C

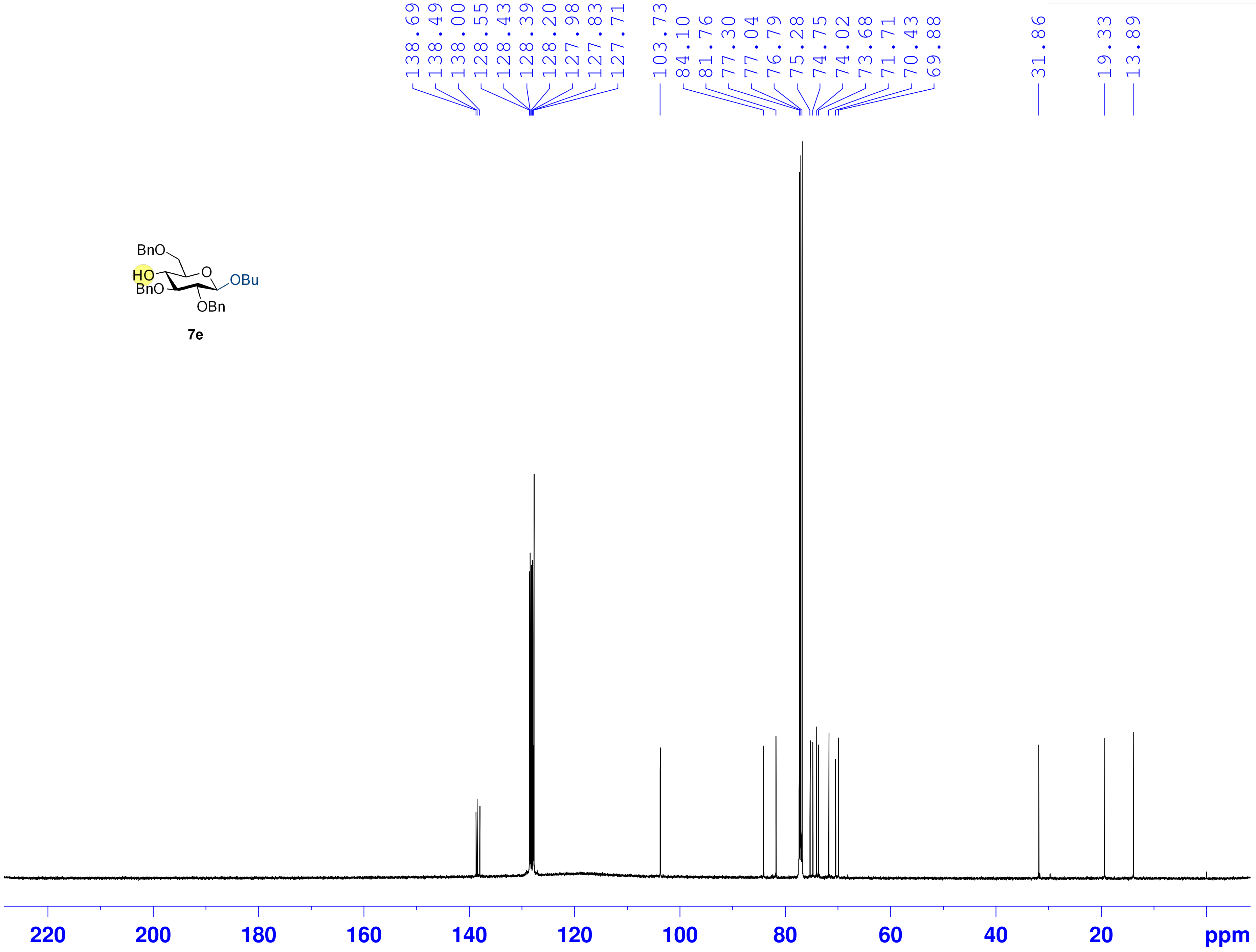

Supplementary Figure 20. 13C-NMR spectrum of compound 7e

	Parameter	Value
1	Solvent	$CDCl_3$
2	Spectrometer Frequency	500 MHz
3	Nucleus	^1H

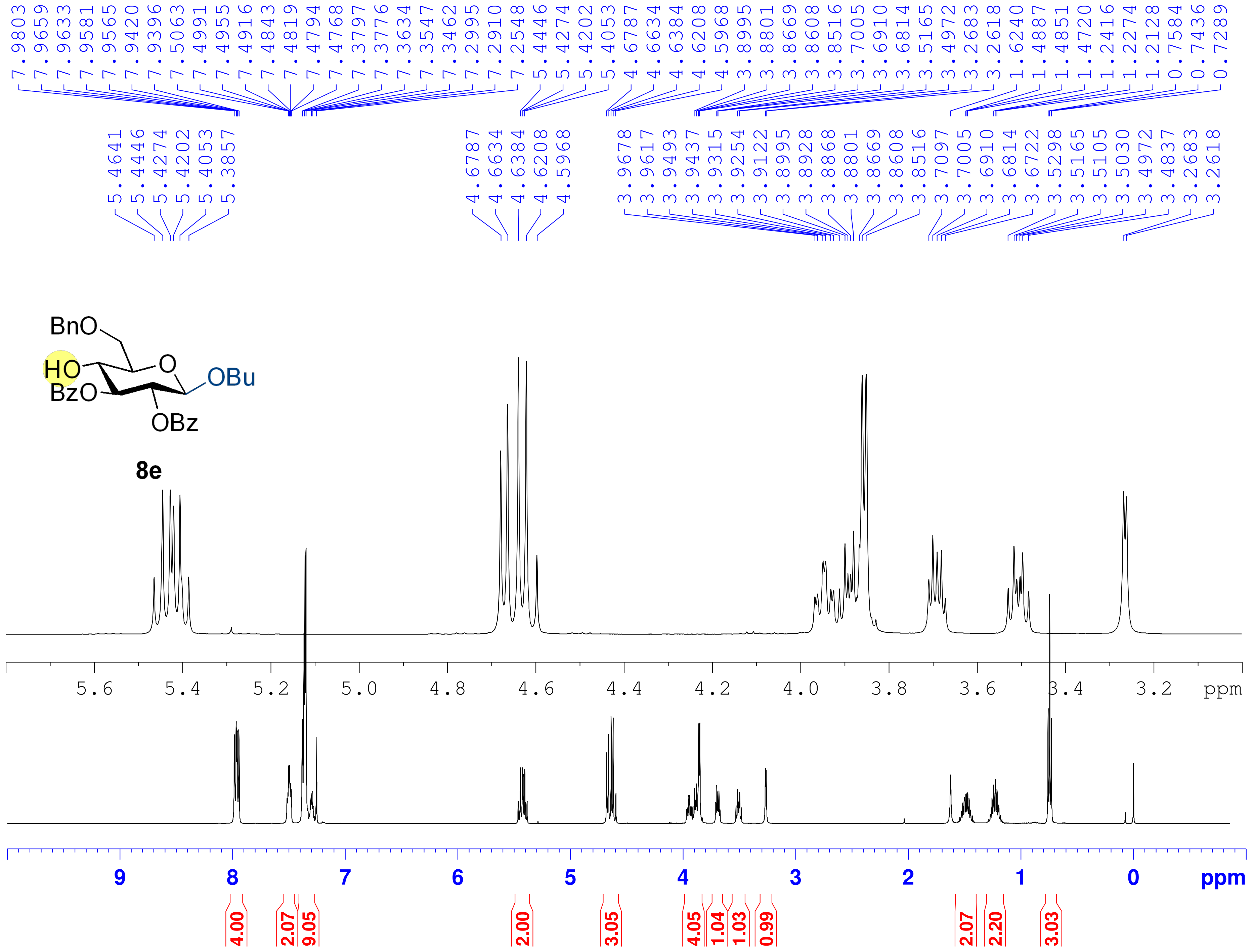

Supplementary Figure 21. 1H-NMR spectrum of compound 8e

	Parameter	Value
1	Solvent	$CDCl_3$
2	Spectrometer Frequency	125 MHz
3	Nucleus	^{13}C

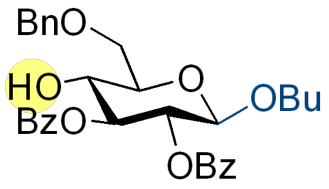

8e

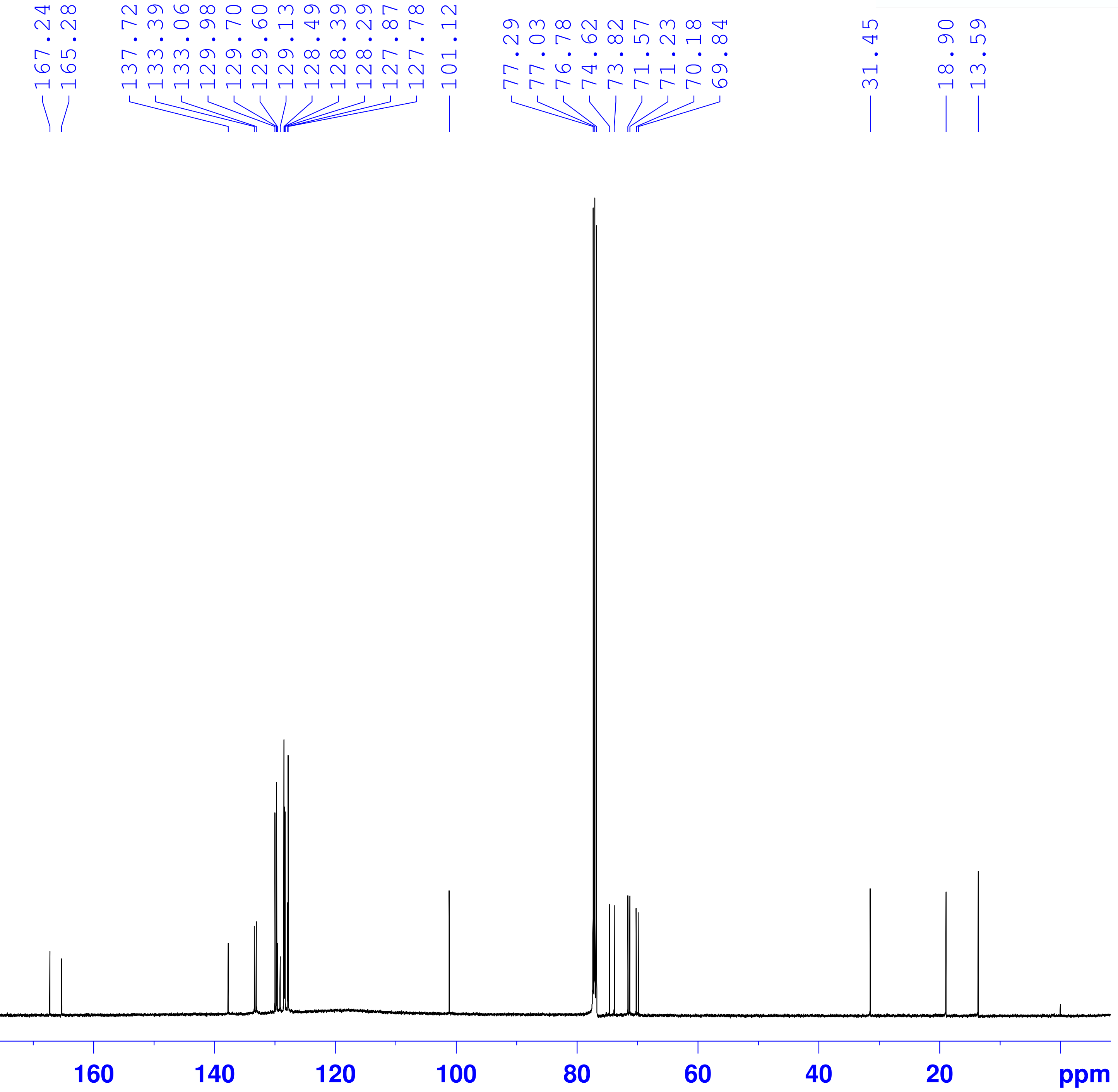

Supplementary Figure 22. 13C-NMR spectrum of compound 8e

	Parameter	Value
1	Solvent	$CDCl_3$
2	Spectrometer Frequency	500 MHz
3	Nucleus	^1H

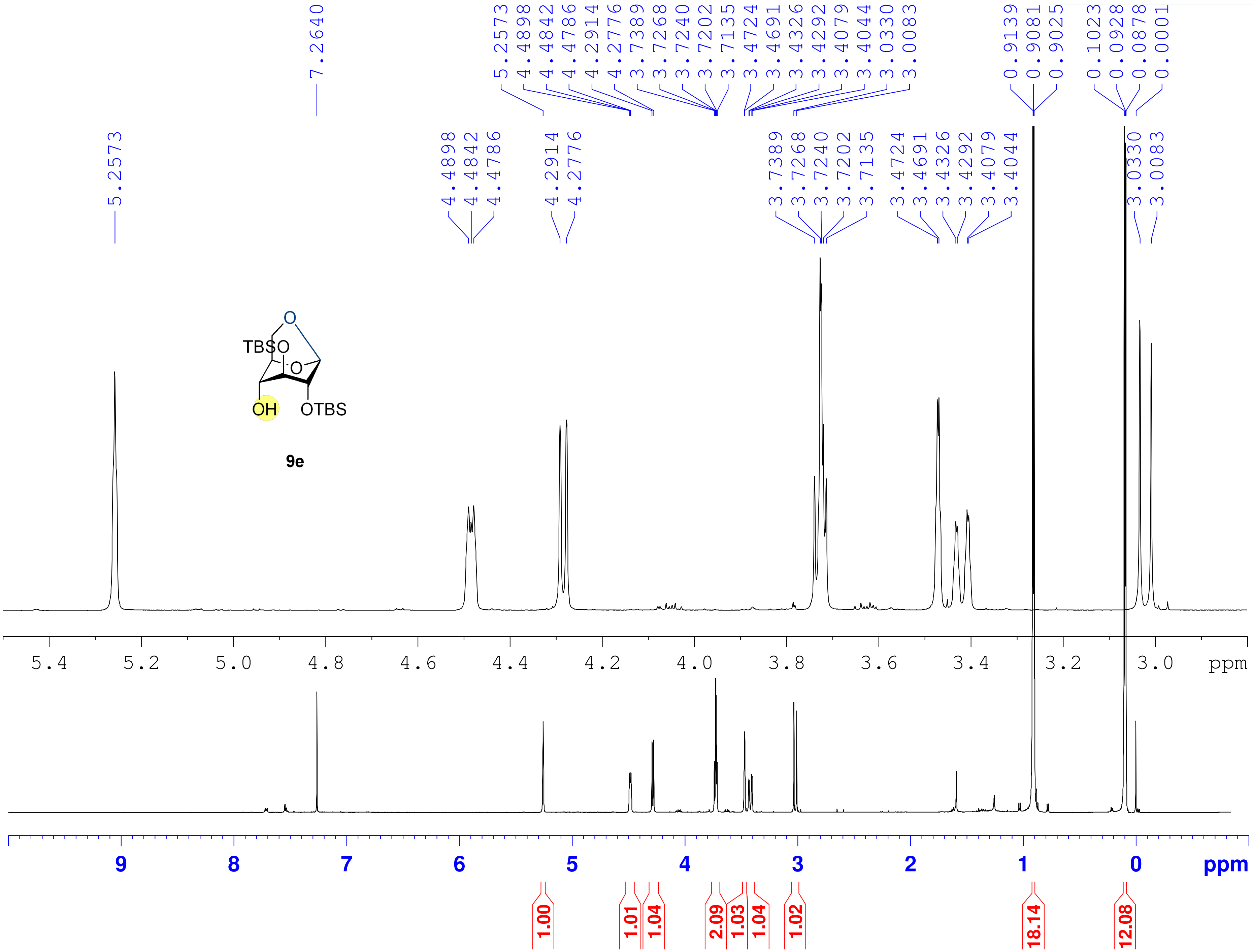

Supplementary Figure 23. 1H-NMR spectrum of compound 9e

	Parameter	Value
1	Solvent	$CDCl_3$
2	Spectrometer Frequency	125 MHz
3	Nucleus	^{13}C

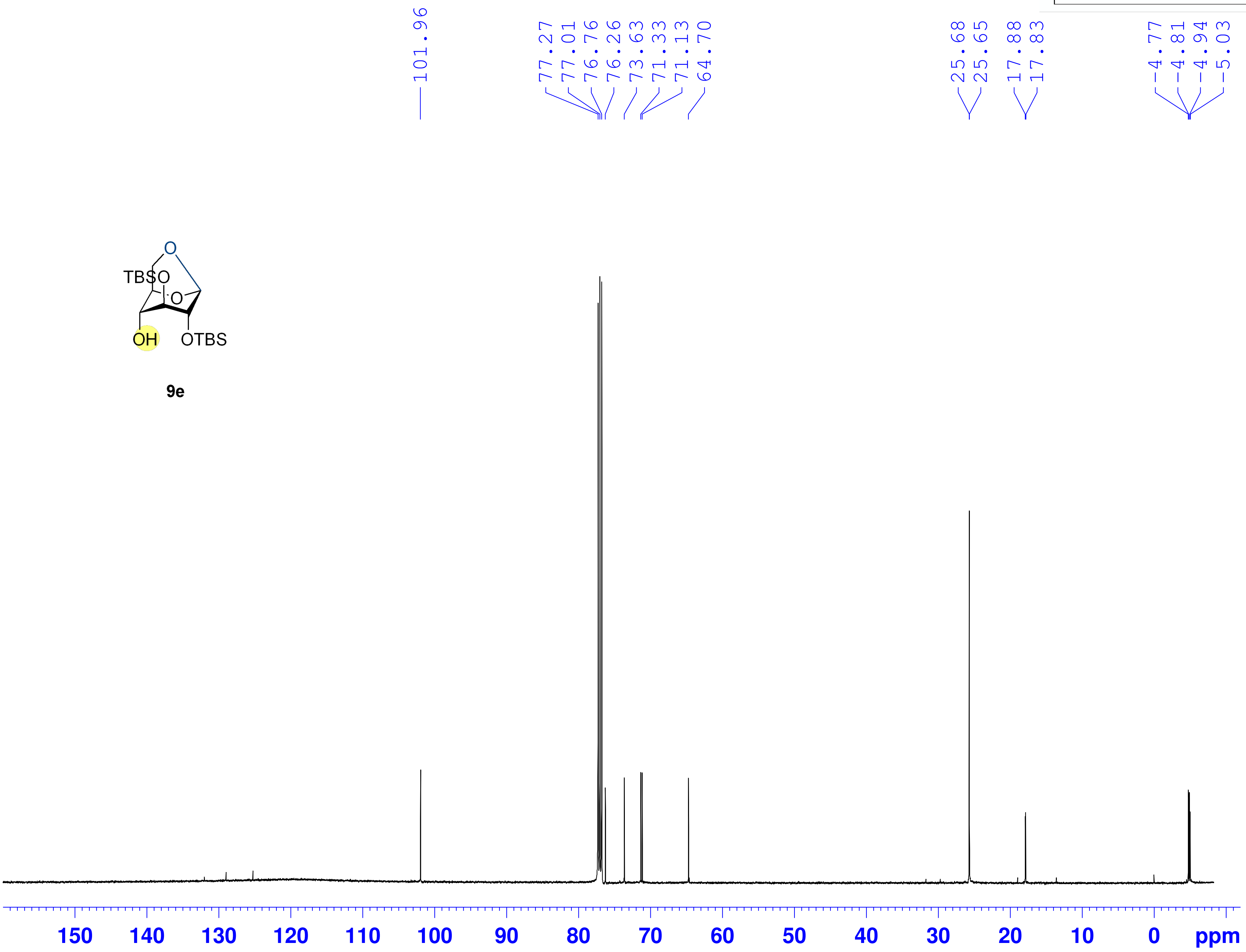

Supplementary Figure 24. 13C-NMR spectrum of compound 9e

	Parameter	Value
1	Solvent	$CDCl_3$
2	Spectrometer Frequency	400 MHz
3	Nucleus	^{1}H

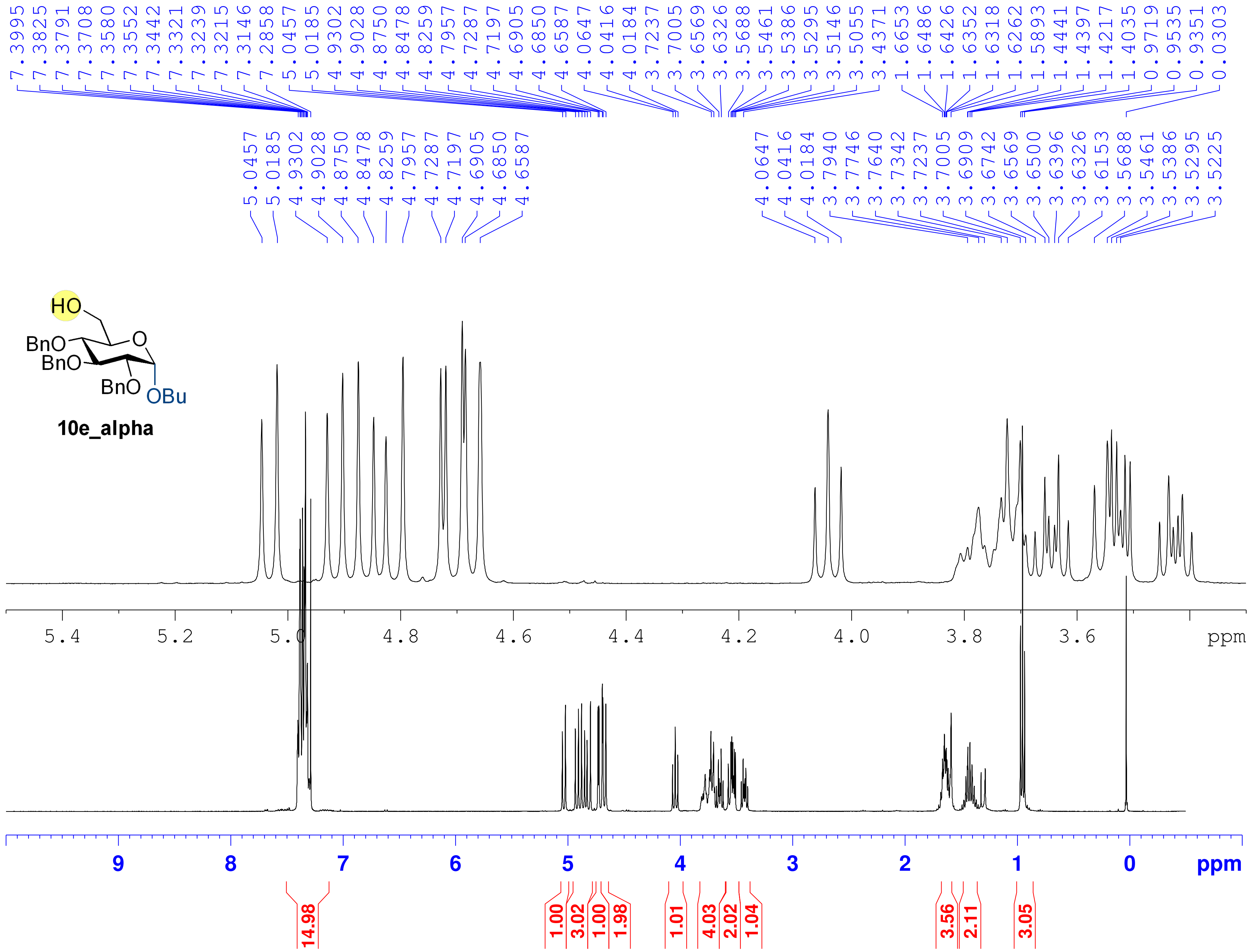

Supplementary Figure 25. 1H-NMR spectrum of compound 10e_alpha

	Parameter	Value
1	Solvent	$CDCl_3$
2	Spectrometer Frequency	100 MHz
3	Nucleus	^{13}C

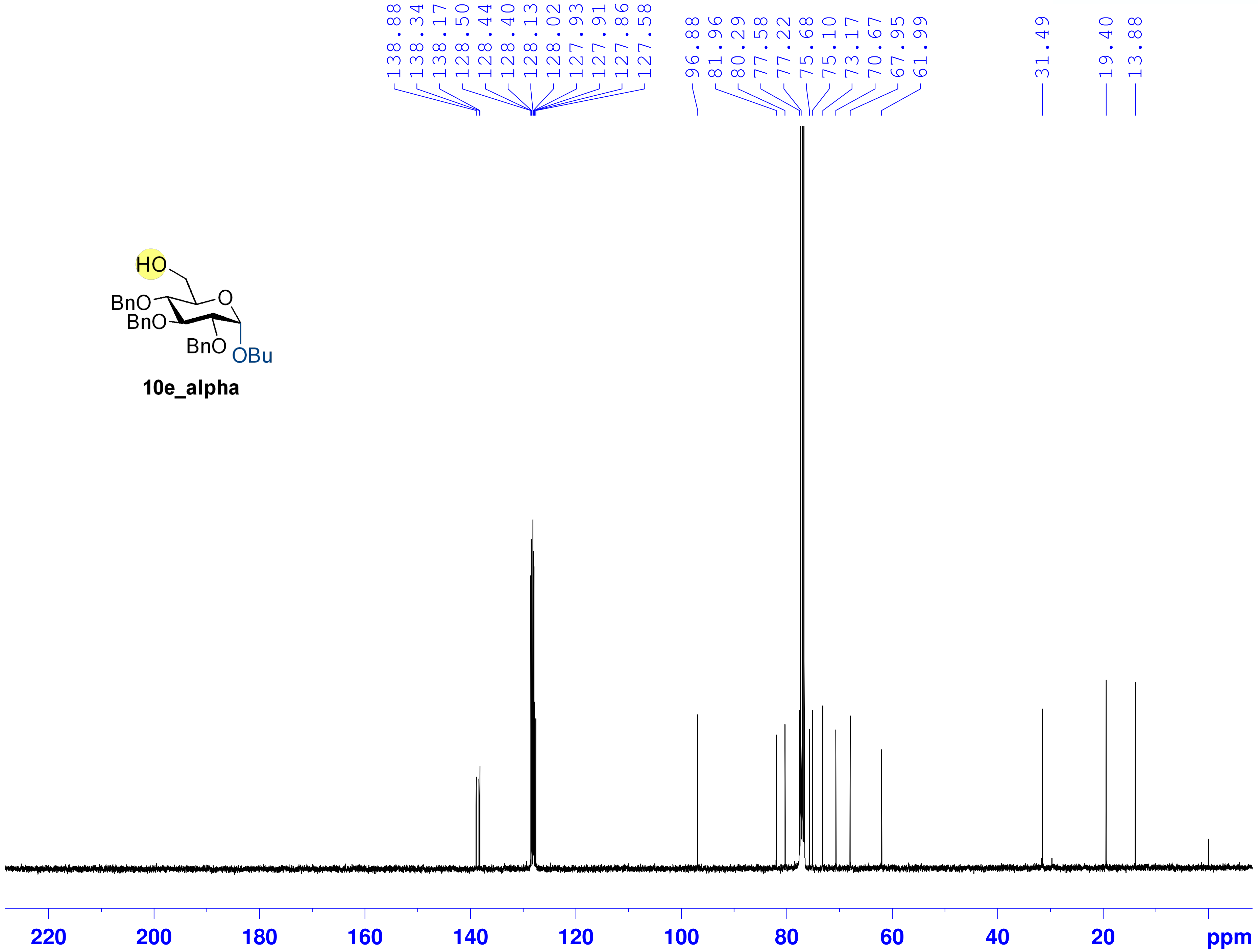

Supplementary Figure 26. 13C-NMR spectrum of compound 10e_alpha

	Parameter	Value
1	Solvent	$CDCl_3$
2	Spectrometer Frequency	400 MHz
3	Nucleus	^1H

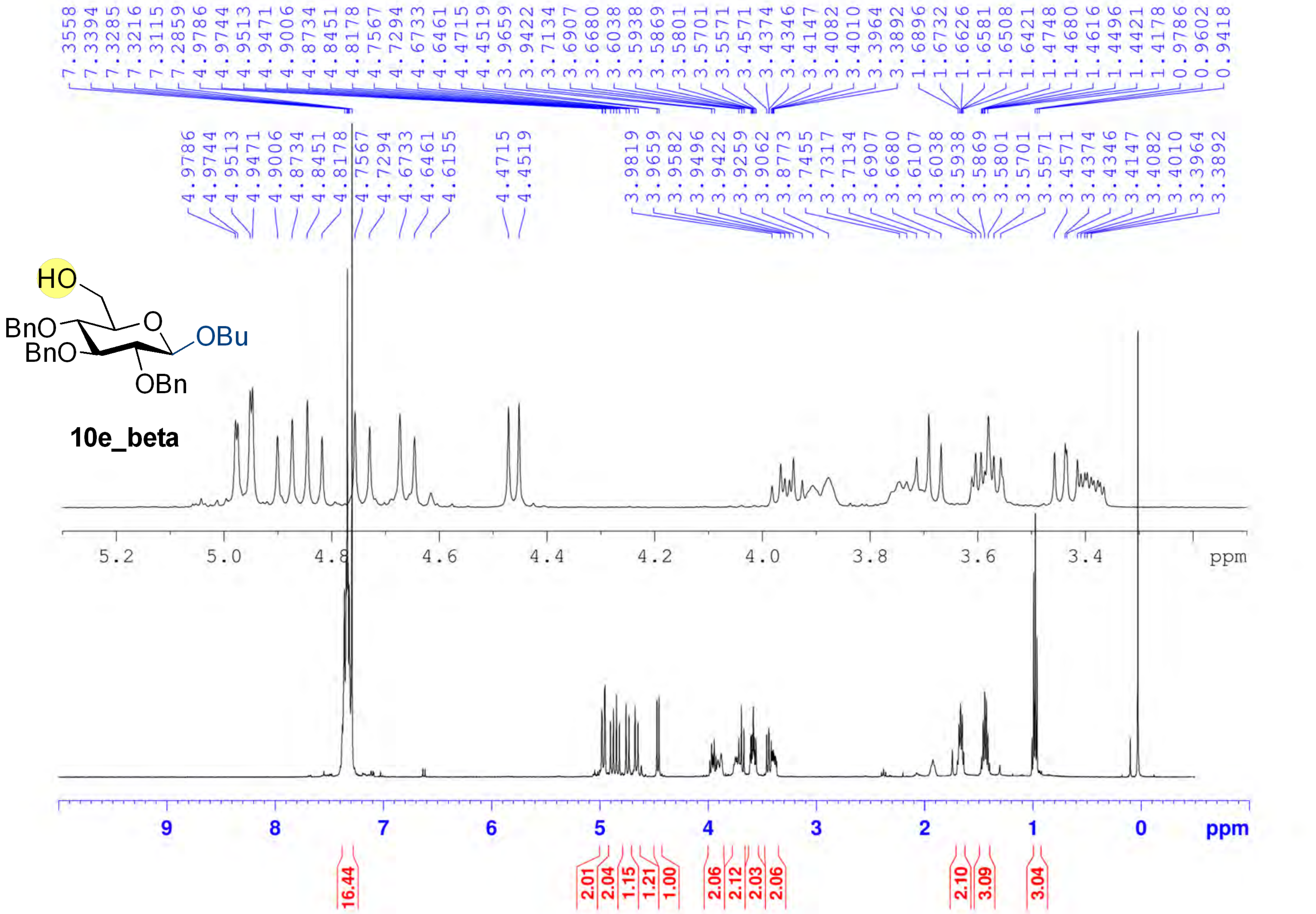

Supplementary Figure 27. 1H-NMR spectrum of compound 10e_beta

	Parameter	Value
1	Solvent	$CDCl_3$
2	Spectrometer Frequency	100 MHz
3	Nucleus	^{13}C

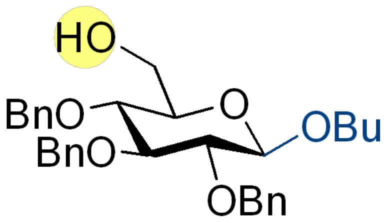

10e_beta

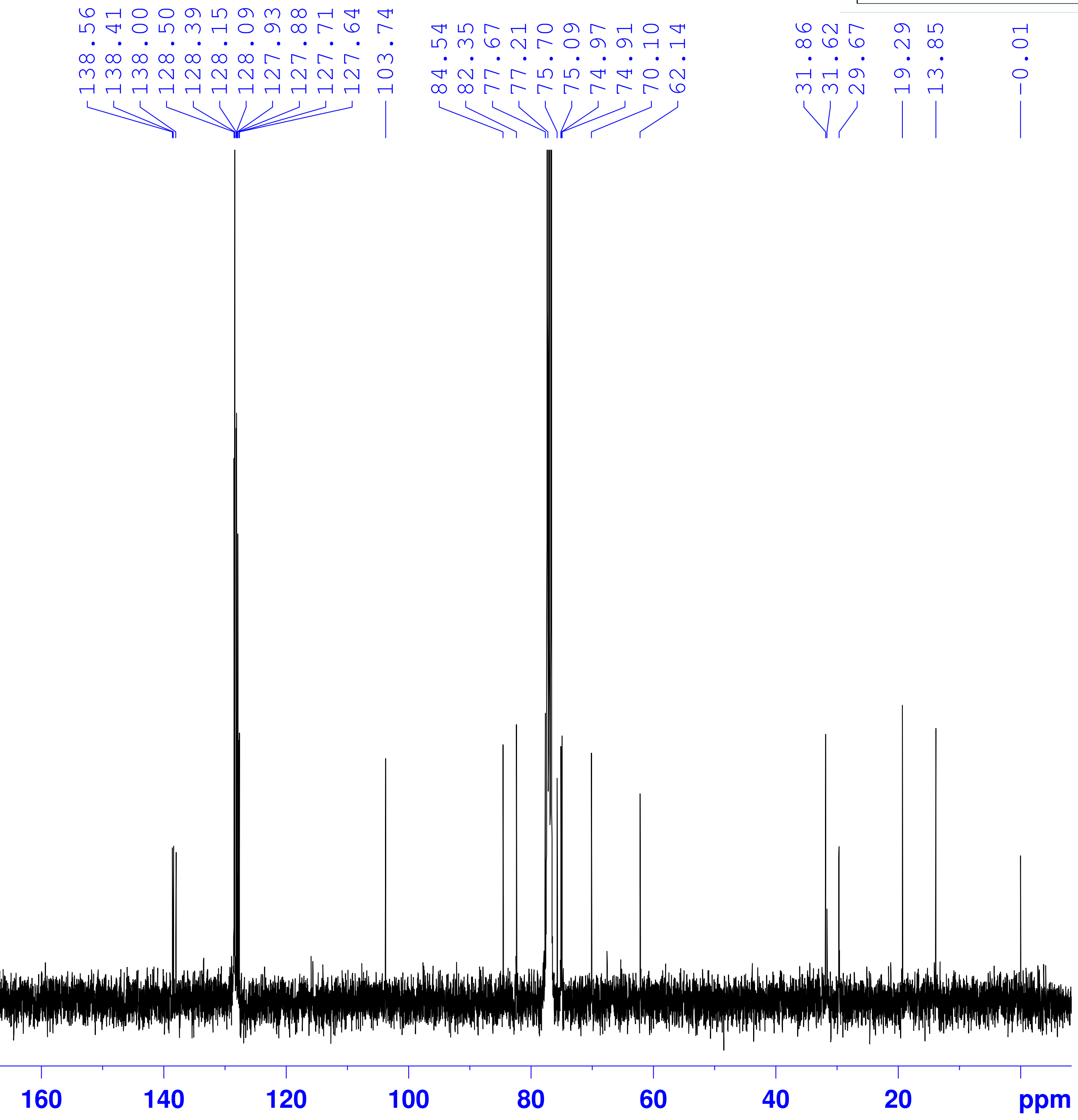

Supplementary Figure 28. 13C-NMR spectrum of compound 10e_beta

	Parameter	Value
1	Solvent	$CDCl_3$
2	Spectrometer Frequency	500 MHz
3	Nucleus	^{1}H

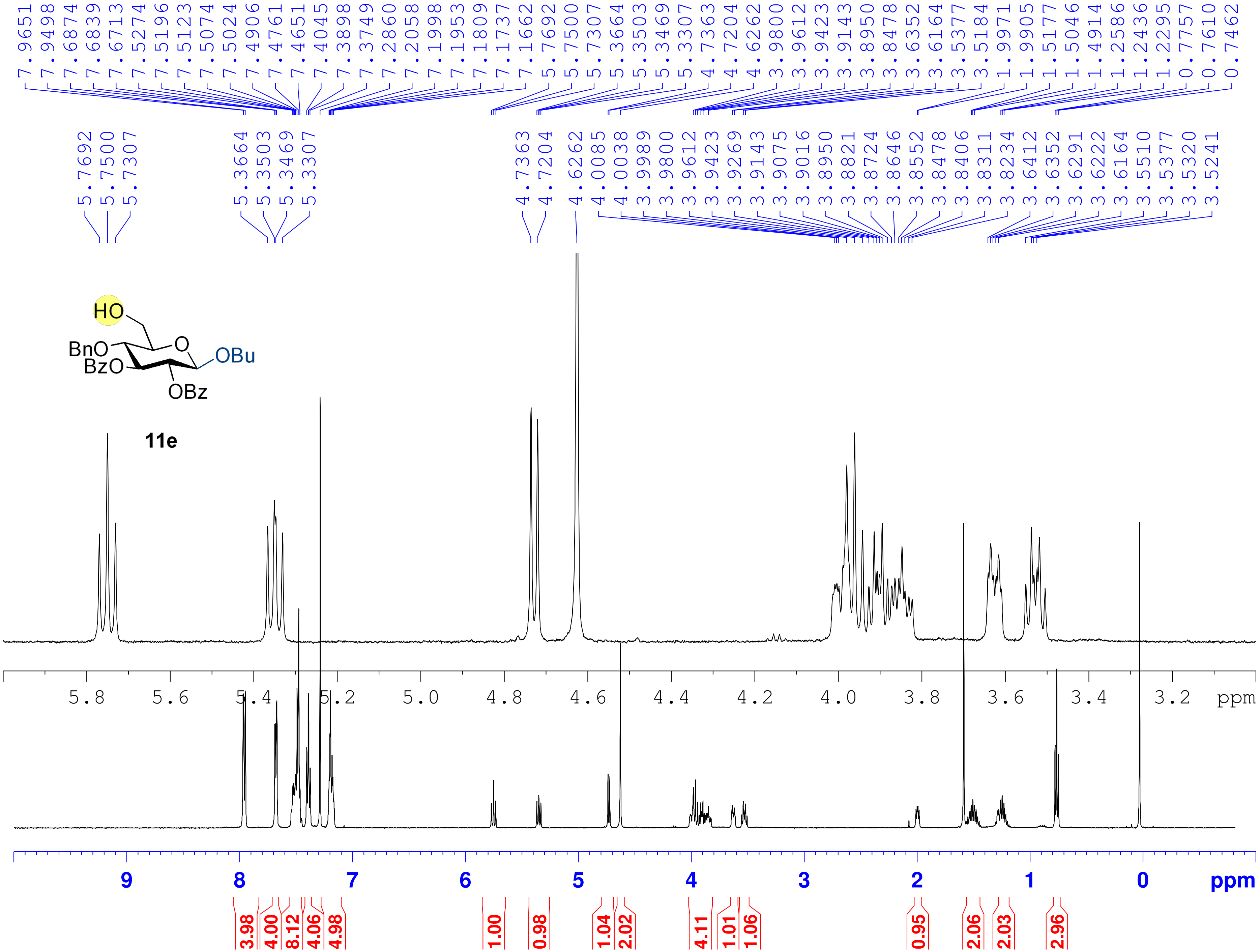

Supplementary Figure 29. 1H-NMR spectrum of compound 11e

	Parameter	Value
1	Solvent	$CDCl_3$
2	Spectrometer Frequency	125 MHz
3	Nucleus	^{13}C

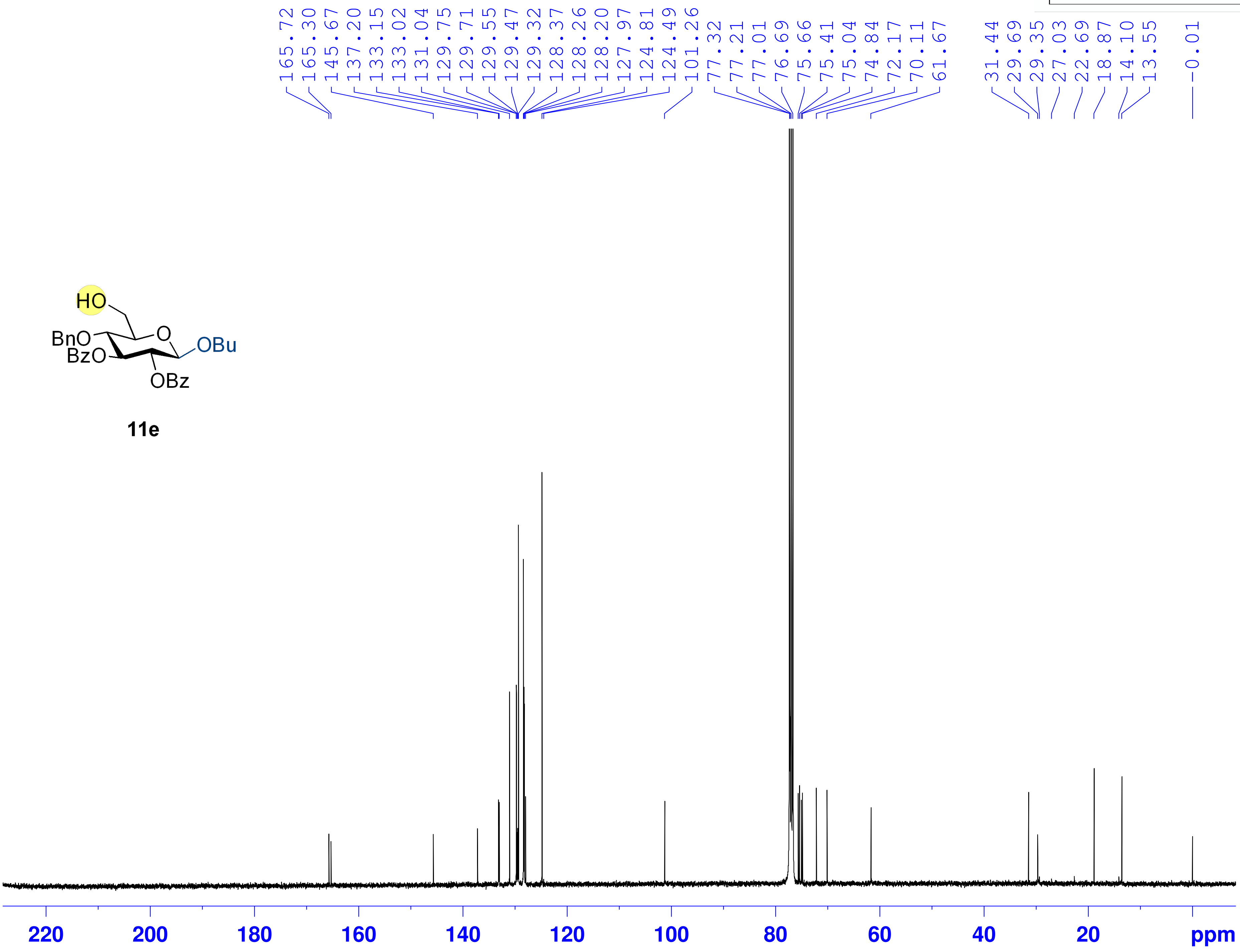

Supplementary Figure 30. 13C-NMR spectrum of compound 11e

	Parameter	Value
1	Solvent	$CDCl_3$
2	Spectrometer Frequency	500 MHz
3	Nucleus	^1H

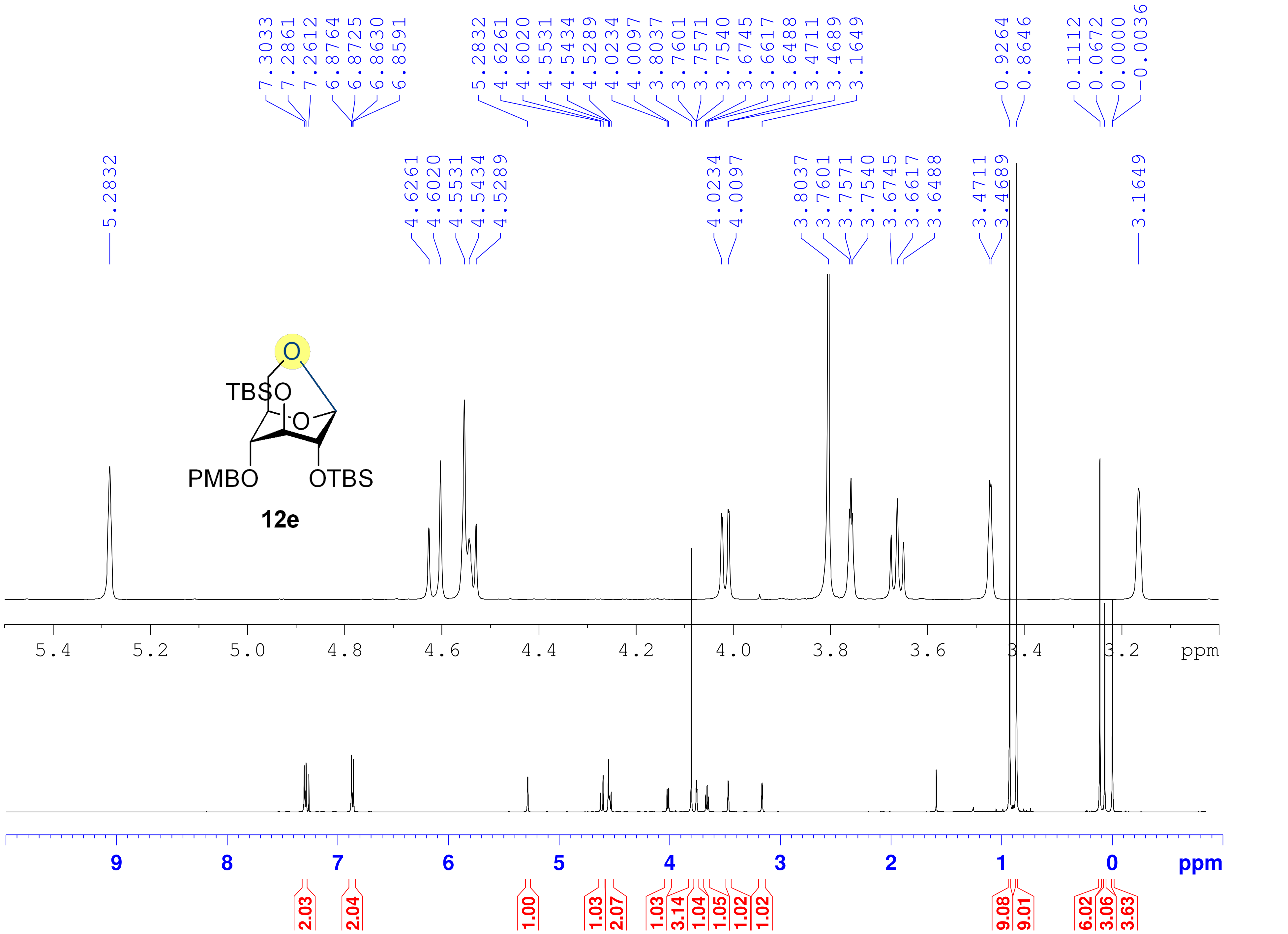

Supplementary Figure 31. 1H-NMR spectrum of compound 12e

	Parameter	Value
1	Solvent	$CDCl_3$
2	Spectrometer Frequency	125 MHz
3	Nucleus	^{13}C

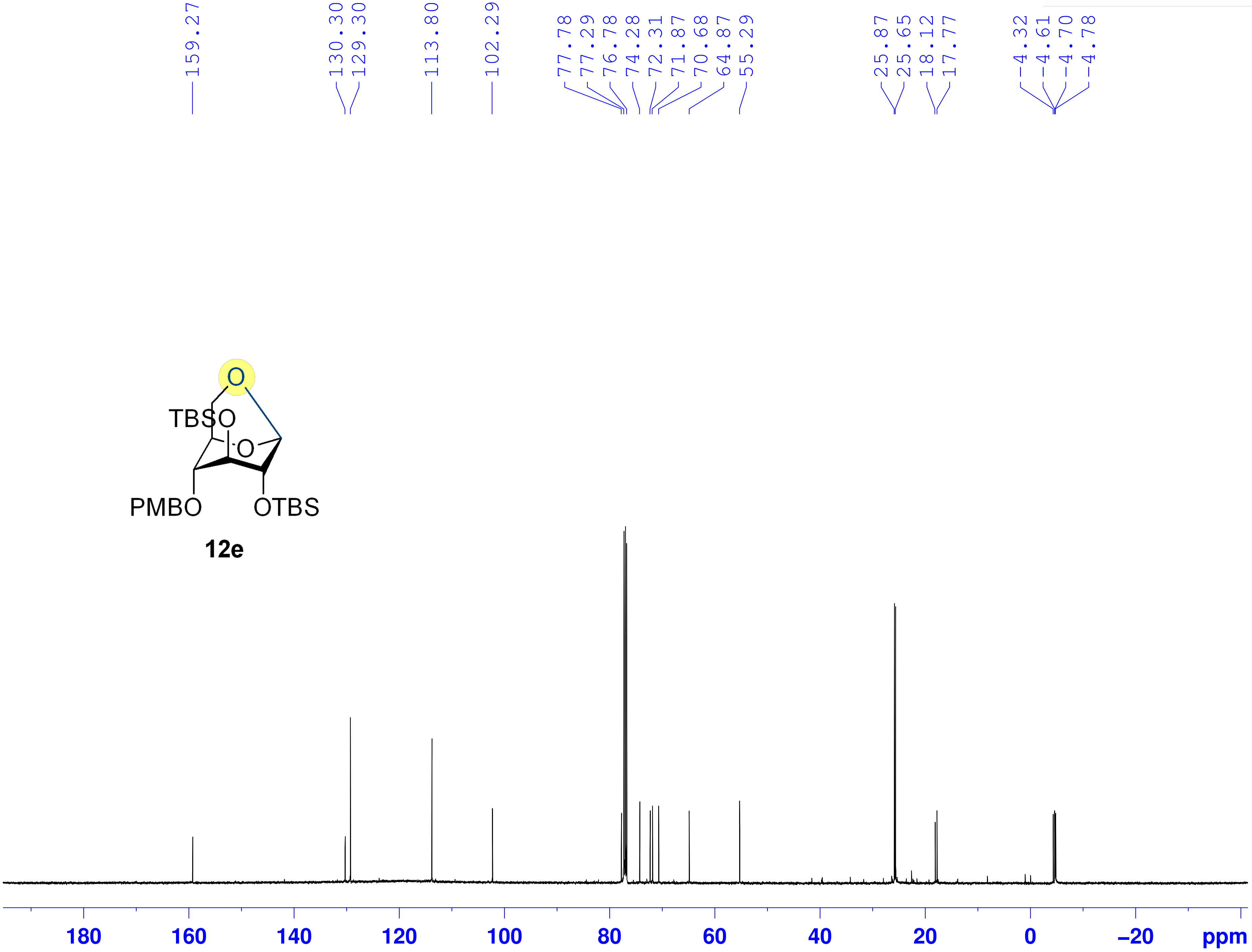

Supplementary Figure 32. 13C-NMR spectrum of compound 12e

	Parameter	Value
1	Solvent	$CDCl_3$
2	Spectrometer Frequency	500 MHz
3	Nucleus	^1H

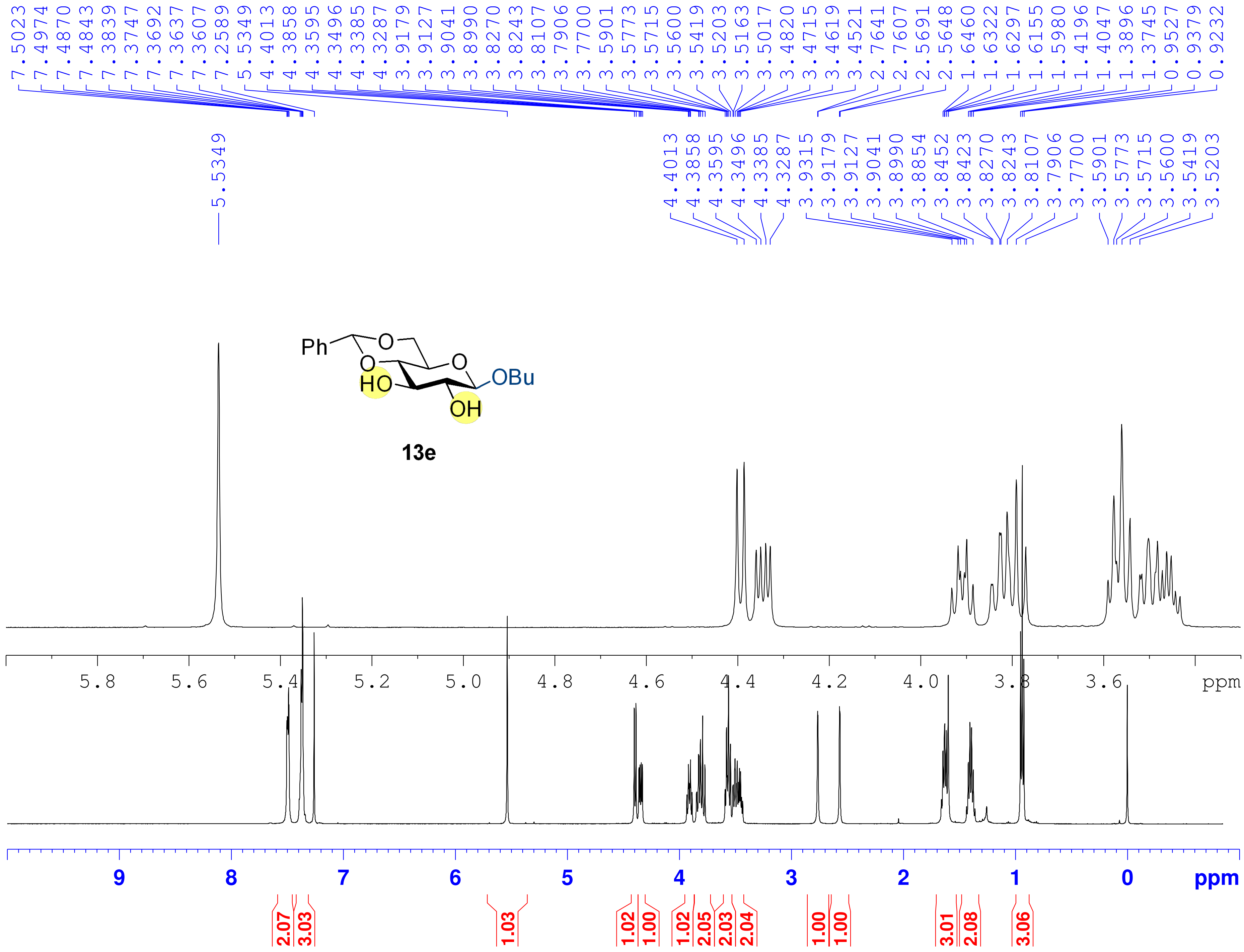

Supplementary Figure 33. 1H-NMR spectrum of compound 13e

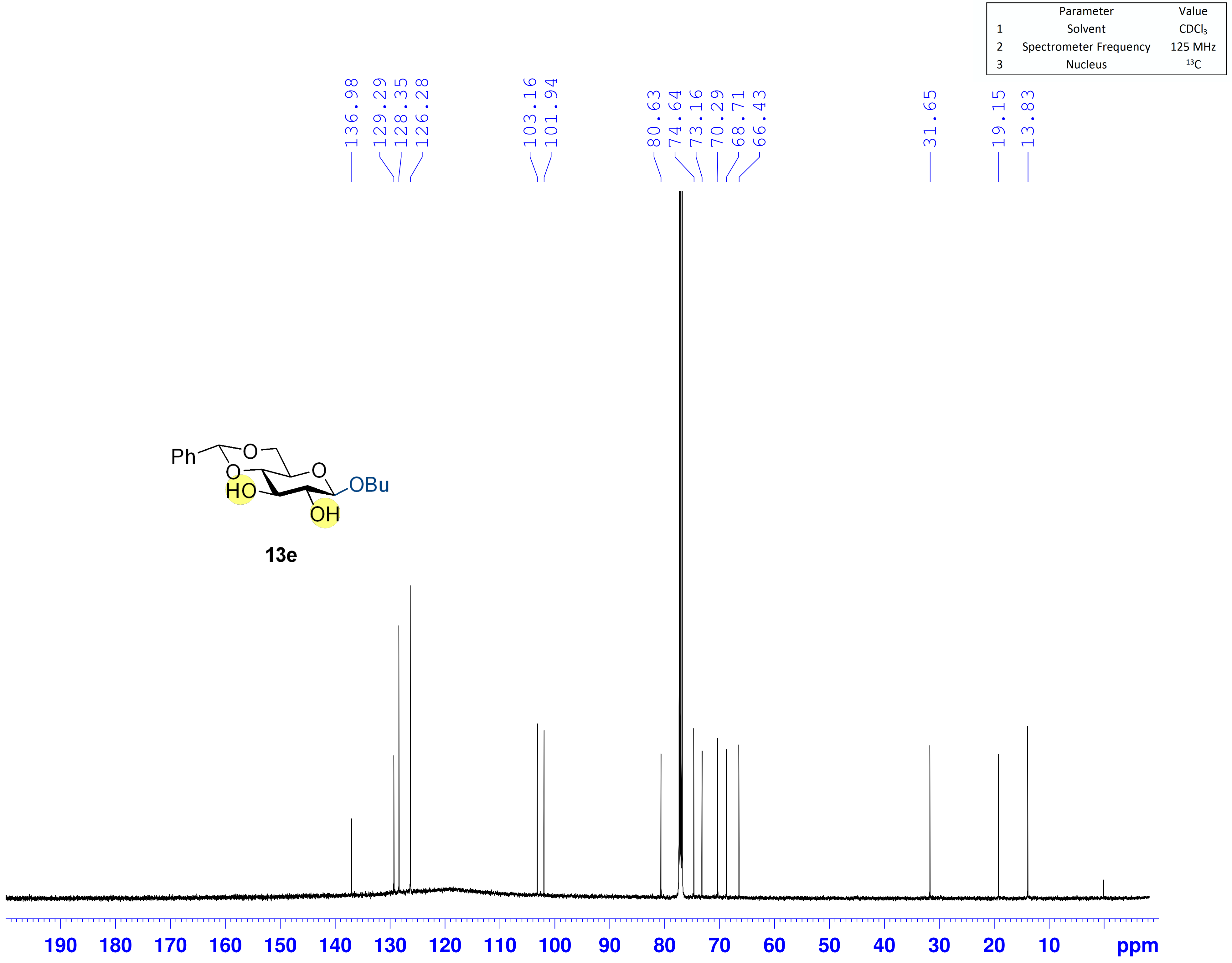

13e

Supplementary Figure 34. 13C-NMR spectrum of compound 13e

	Parameter	Value
1	Solvent	$CDCl_3$
2	Spectrometer Frequency	500 MHz
3	Nucleus	^1H

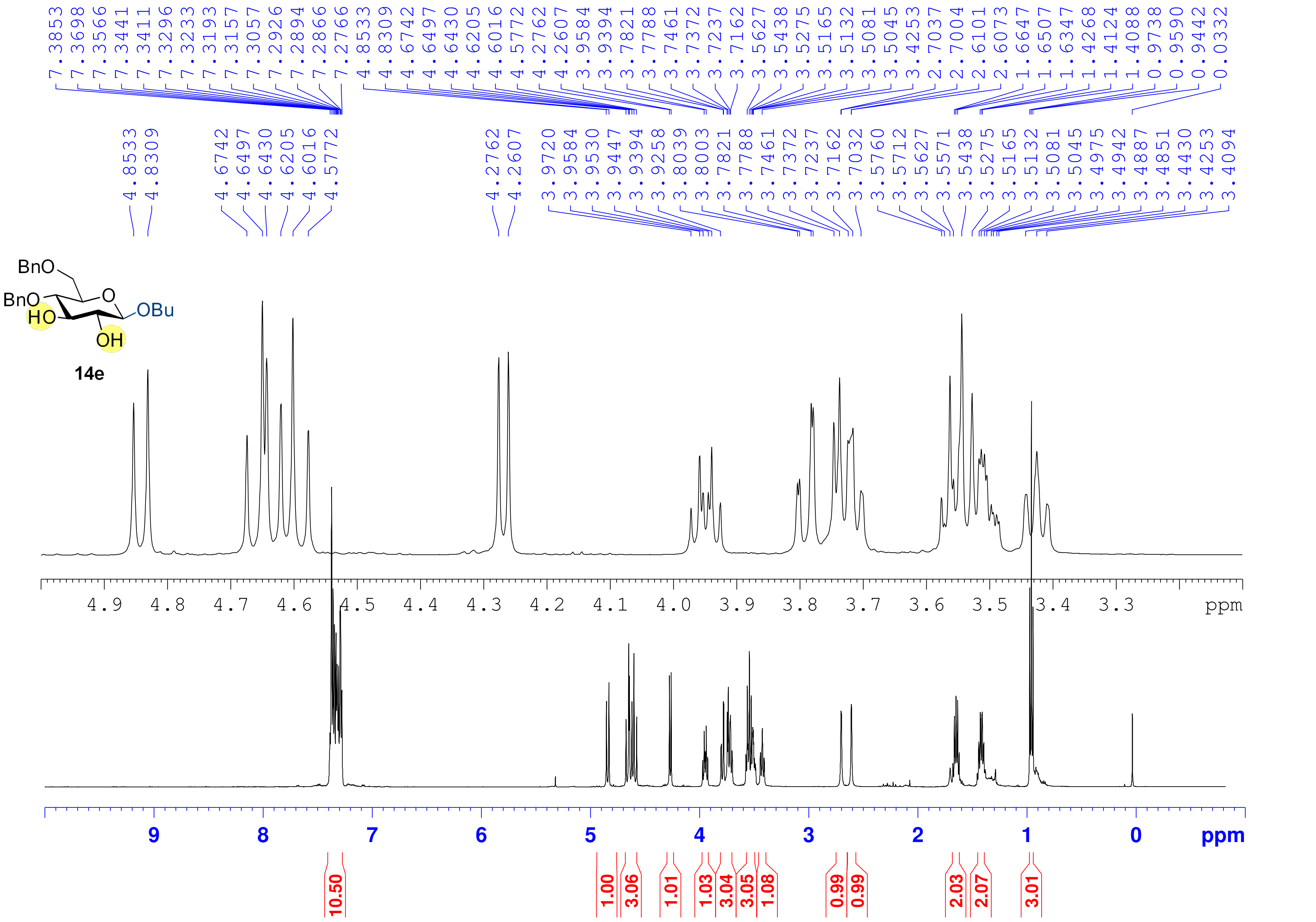

Supplementary Figure 35. 1H-NMR spectrum of compound 14e

	Parameter	Value
1	Solvent	$CDCl_3$
2	Spectrometer Frequency	125 MHz
3	Nucleus	^{13}C

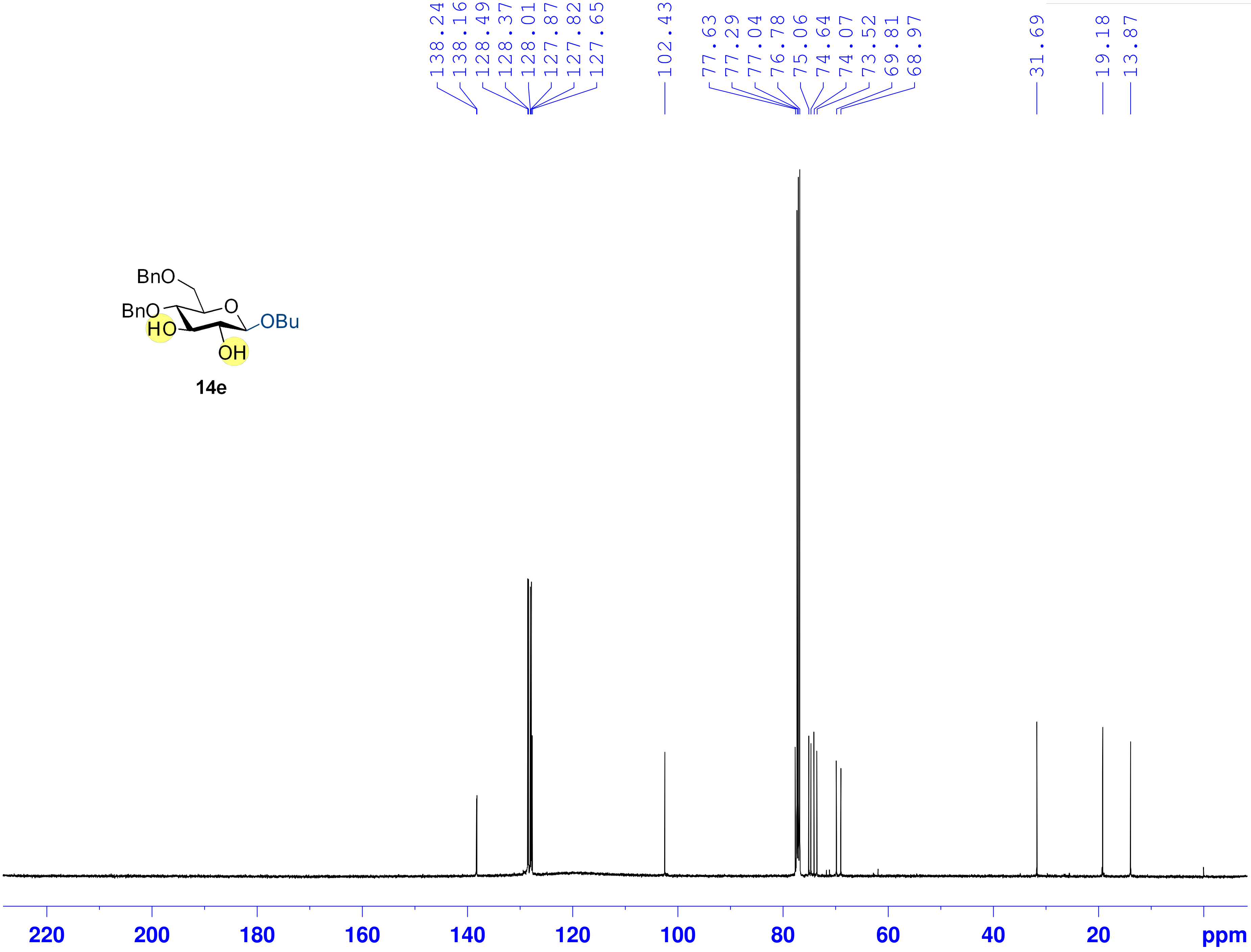

Supplementary Figure 36. 13C-NMR spectrum of compound 14e

	Parameter	Value
1	Solvent	$CDCl_3$
2	Spectrometer Frequency	500 MHz
3	Nucleus	^1H

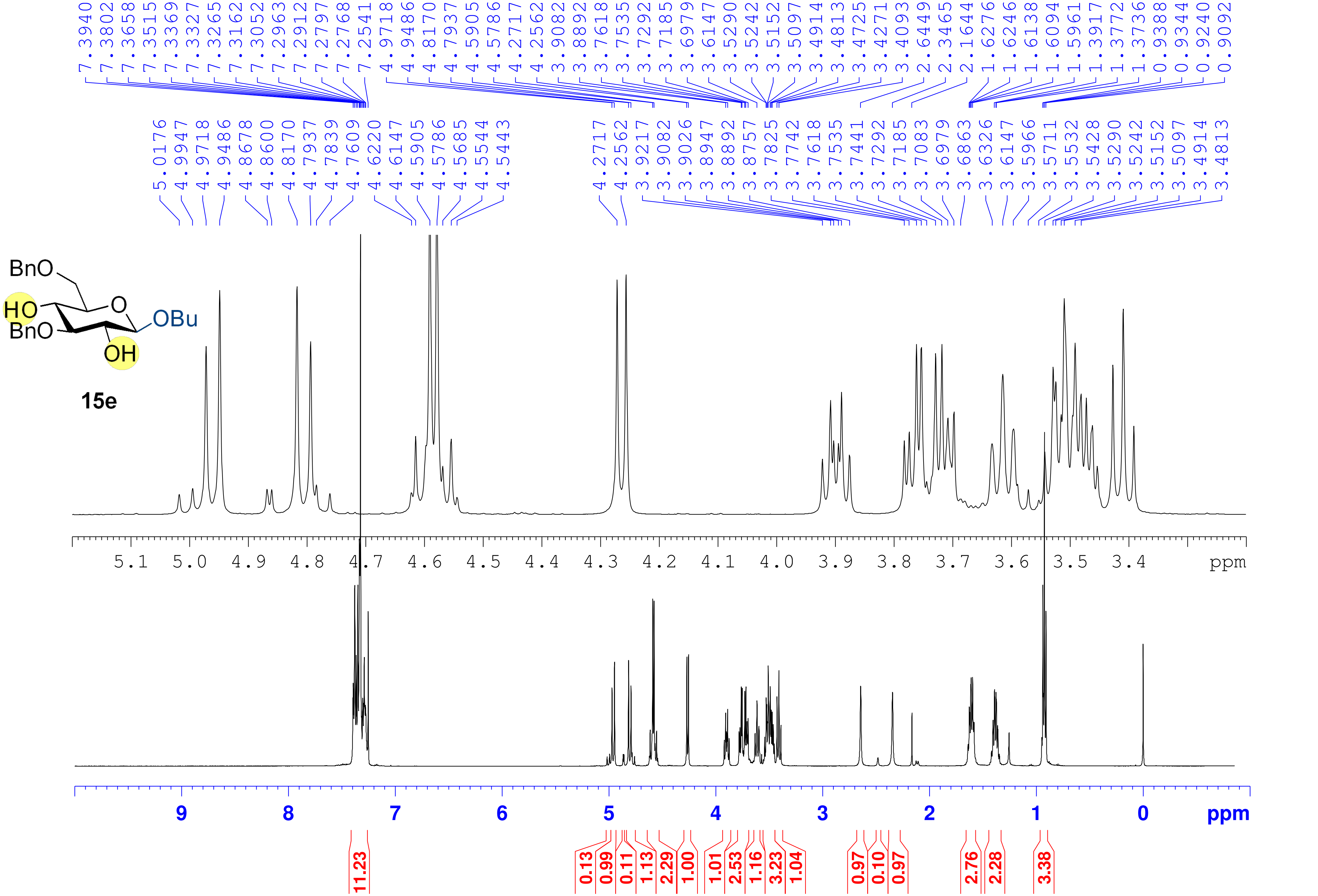

Supplementary Figure 37. 1H-NMR spectrum of compound 15e

	Parameter	Value
1	Solvent	$CDCl_3$
2	Spectrometer Frequency	125 MHz
3	Nucleus	^{13}C

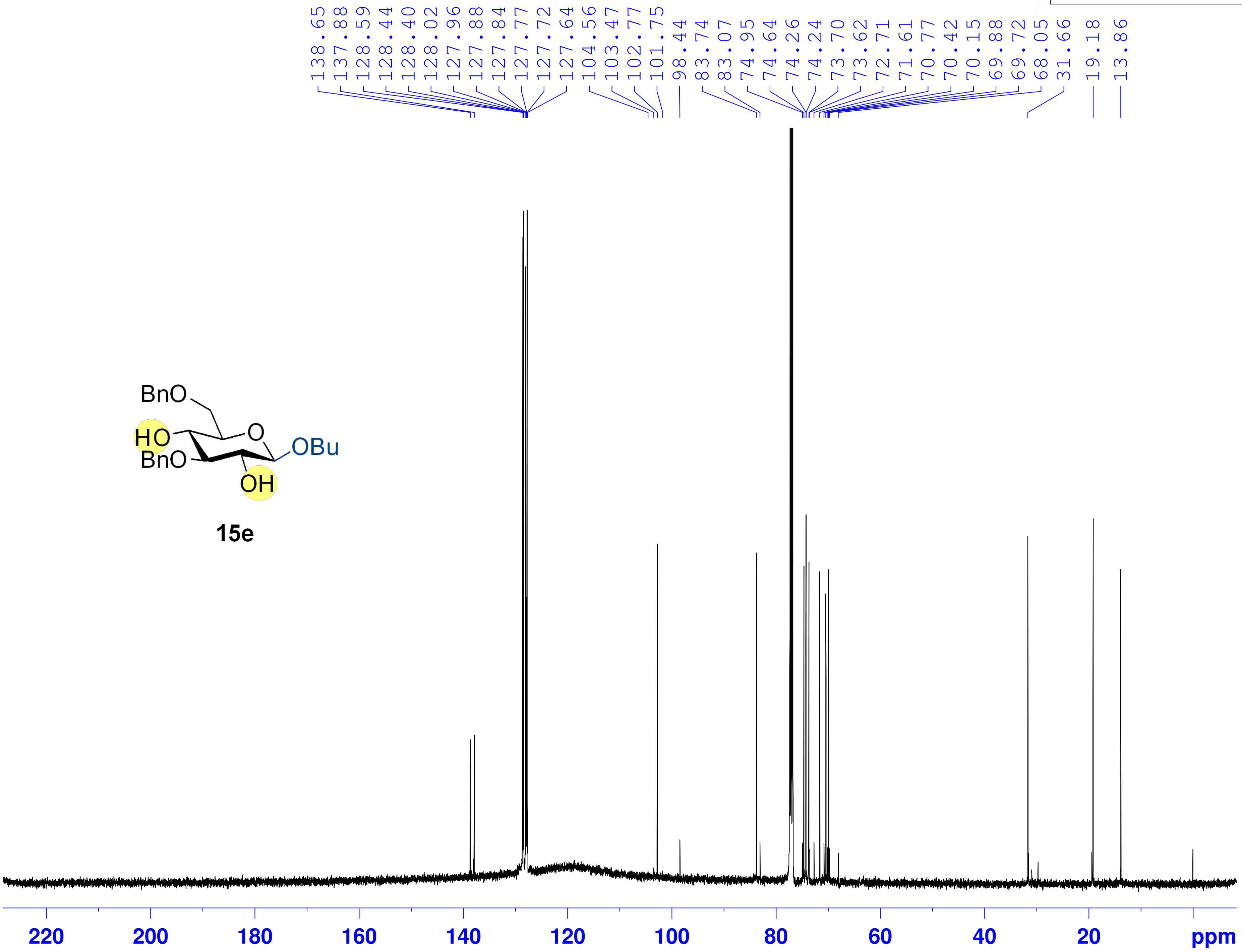

Supplementary Figure 38. 13C-NMR spectrum of compound 15e

	Parameter	Value
1	Solvent	$CDCl_3$
2	Spectrometer Frequency	500 MHz
3	Nucleus	^{1}H

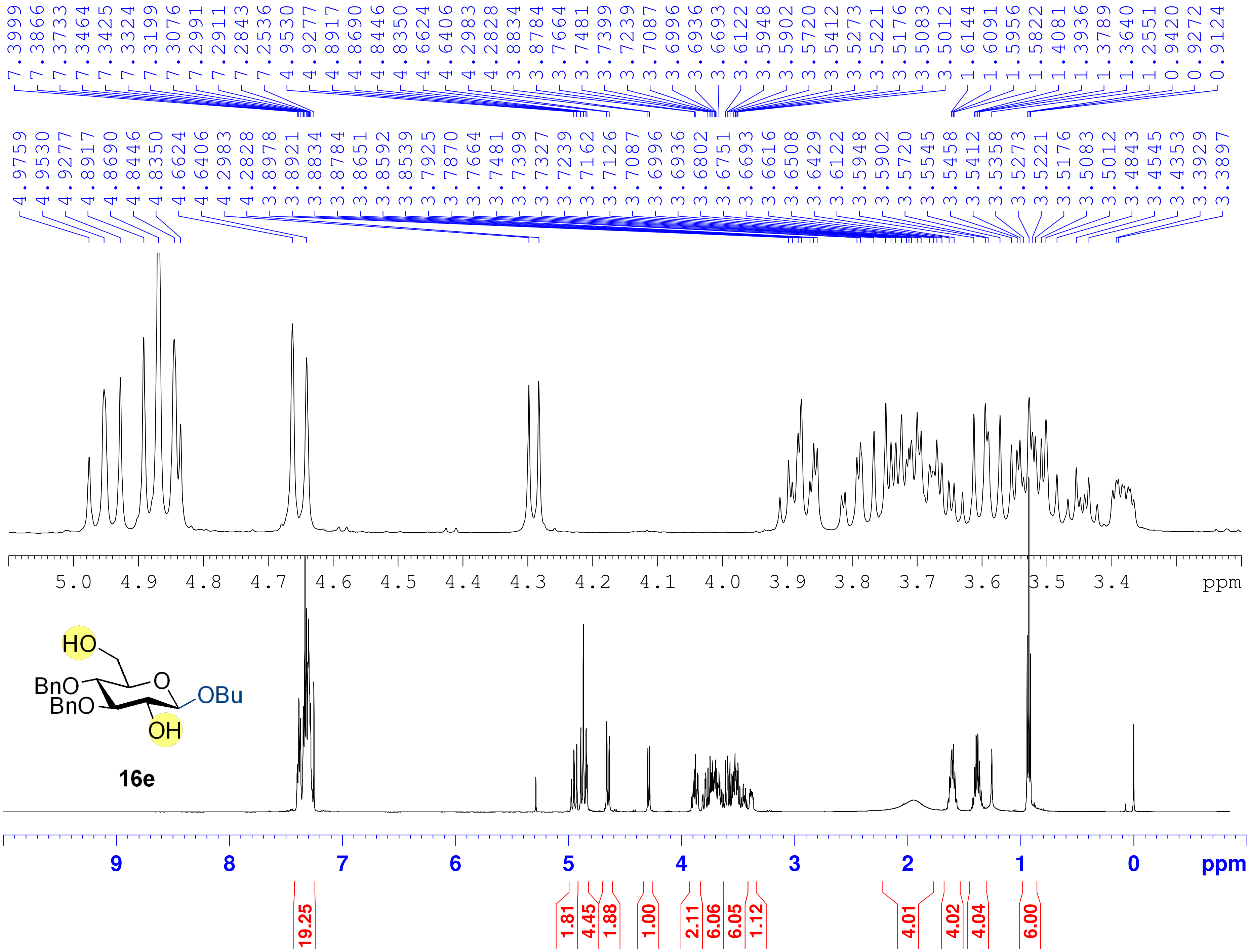

Supplementary Figure 39. 1H-NMR spectrum of compound 16e

	Parameter	Value
1	Solvent	$CDCl_3$
2	Spectrometer Frequency	125 MHz
3	Nucleus	^{13}C

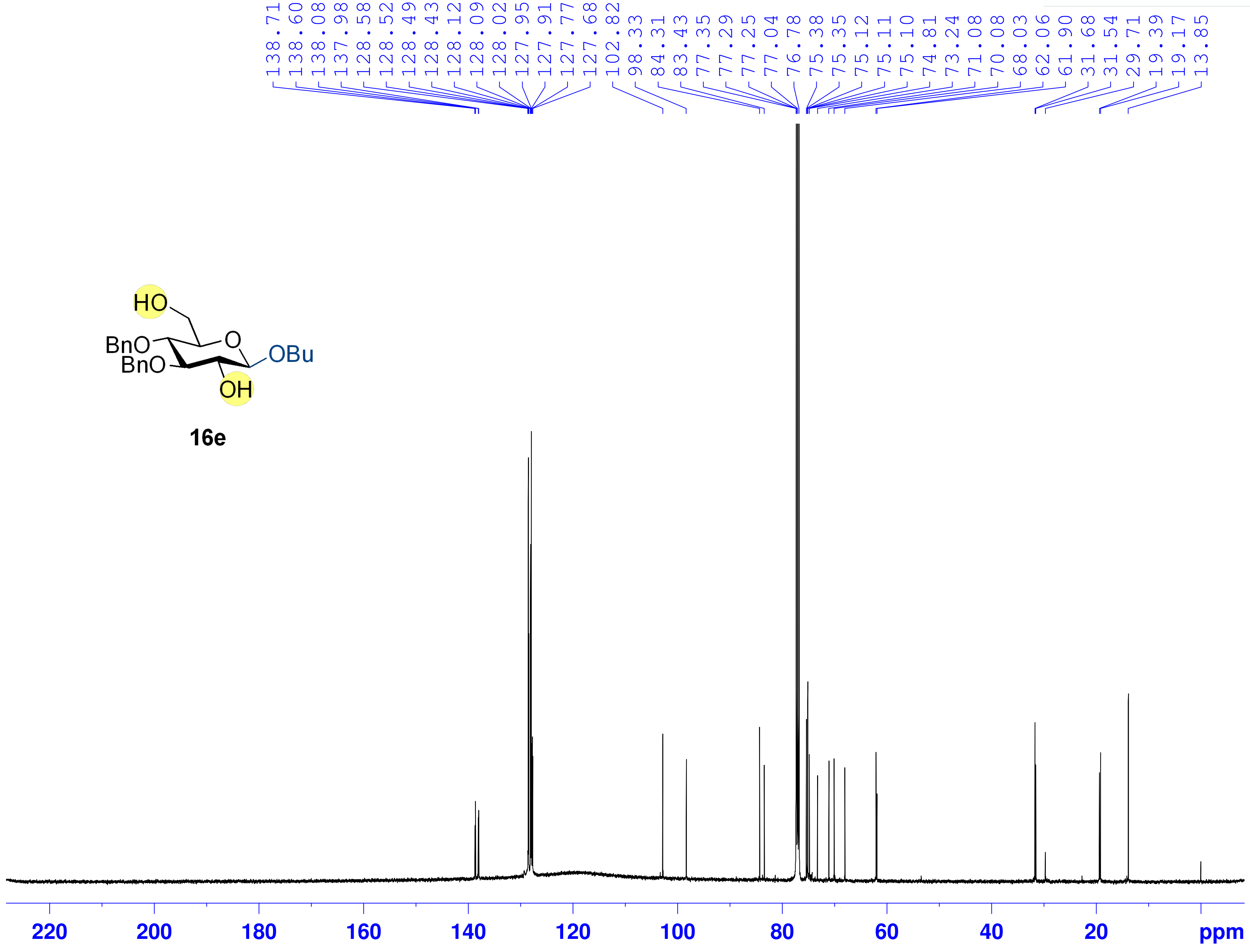

Supplementary Figure 40. 13C-NMR spectrum of compound 16e

	Parameter	Value
1	Solvent	$CDCl_3$
2	Spectrometer Frequency	500 MHz
3	Nucleus	^1H

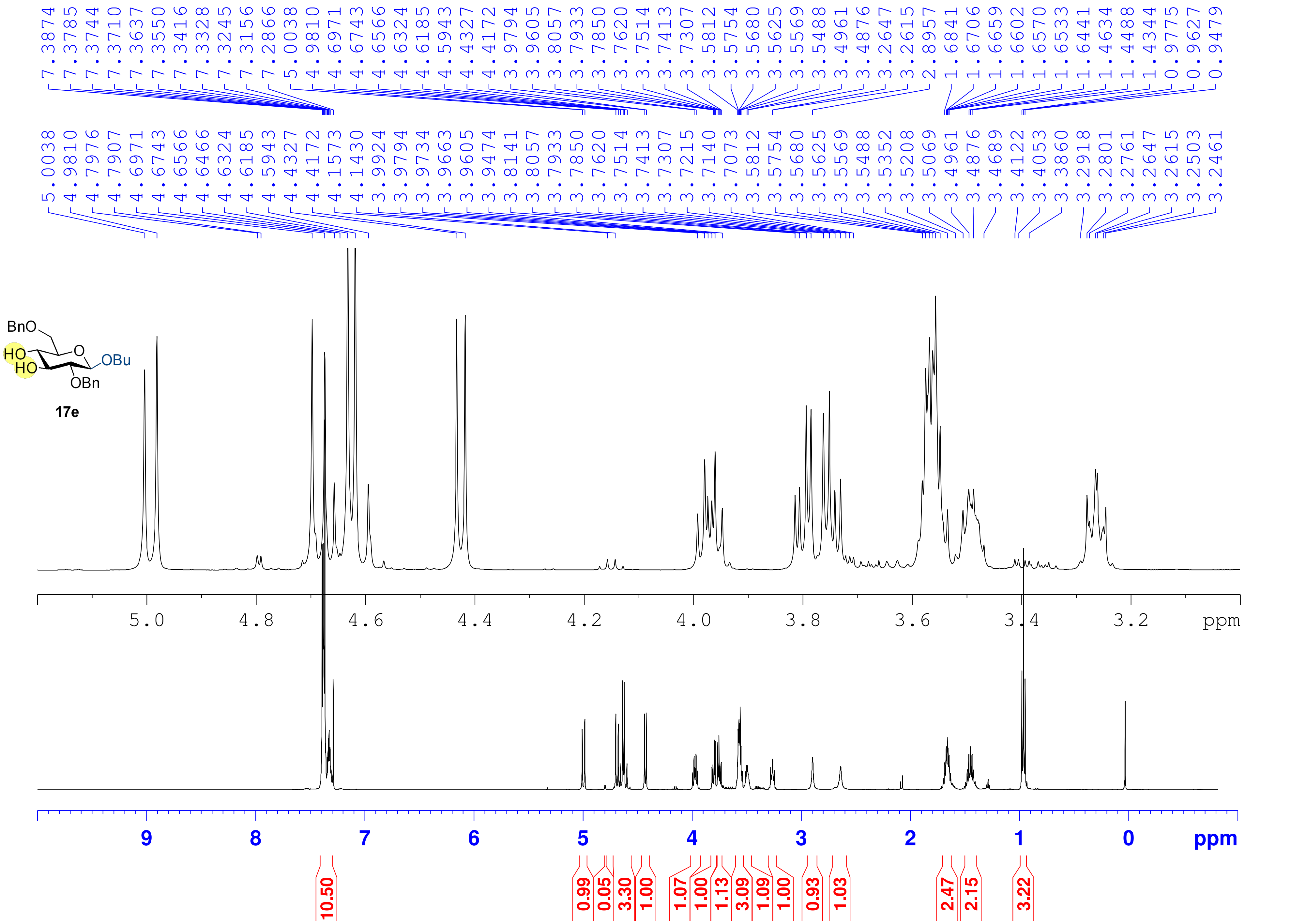

Supplementary Figure 41. 1H-NMR spectrum of compound 17e

	Parameter	Value
1	Solvent	$CDCl_3$
2	Spectrometer Frequency	125 MHz
3	Nucleus	^{13}C

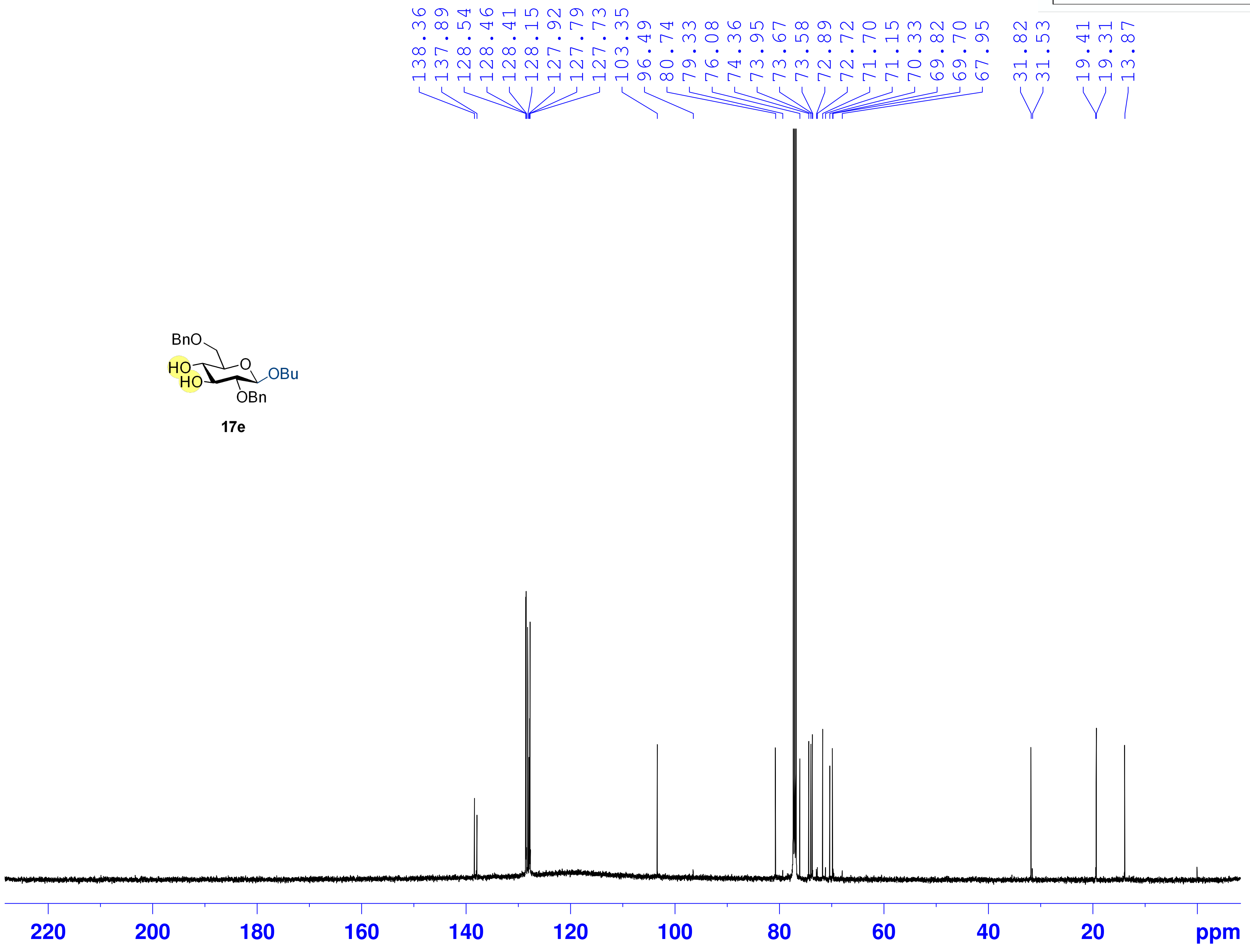

Supplementary Figure 42. 13C-NMR spectrum of compound 17e

	Parameter	Value
1	Solvent	$CDCl_3$
2	Spectrometer Frequency	500 MHz
3	Nucleus	^1H

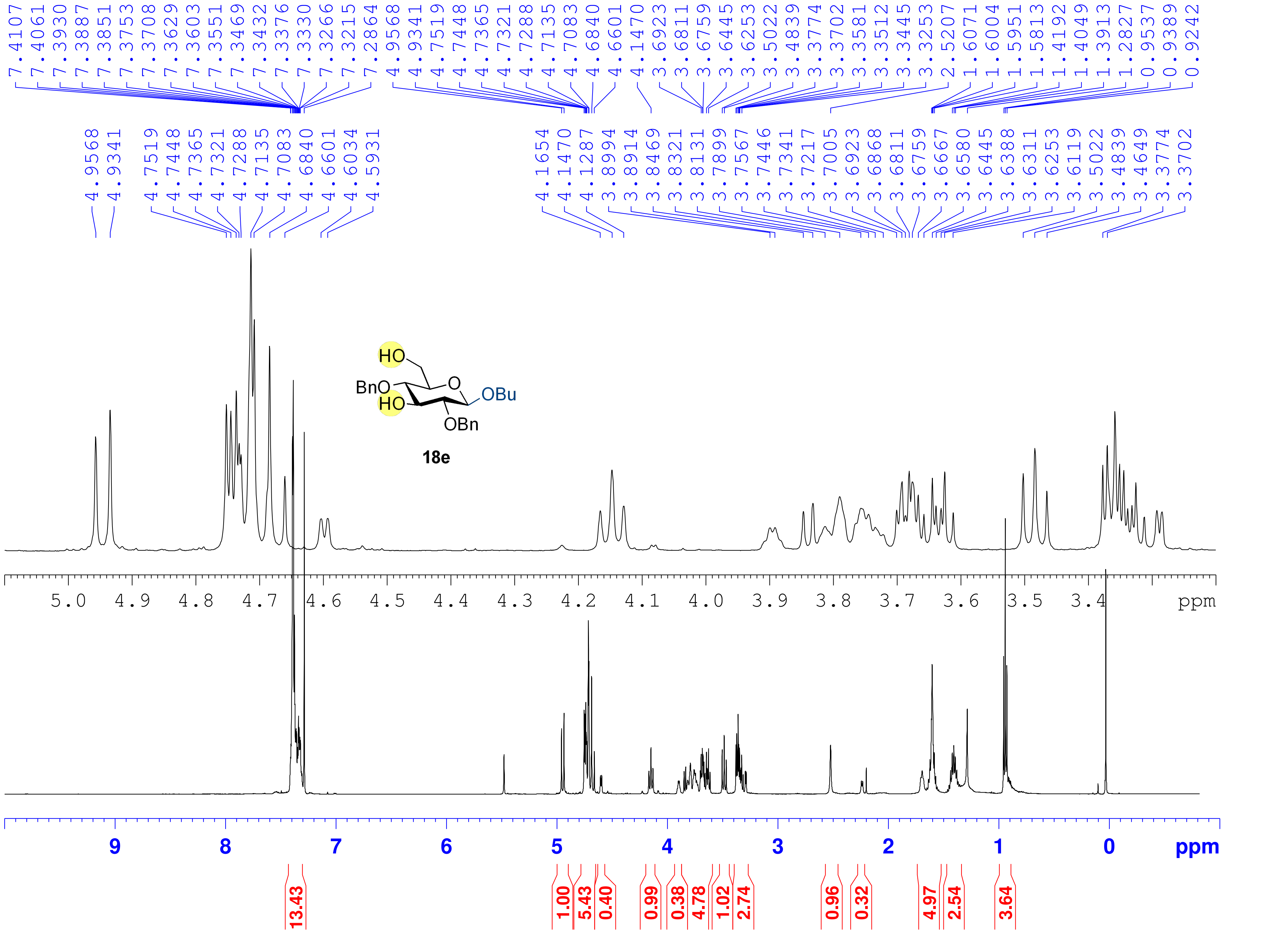

Supplementary Figure 43. 1H-NMR spectrum of compound 18e

	Parameter	Value
1	Solvent	$CDCl_3$
2	Spectrometer Frequency	125 MHz
3	Nucleus	^{13}C

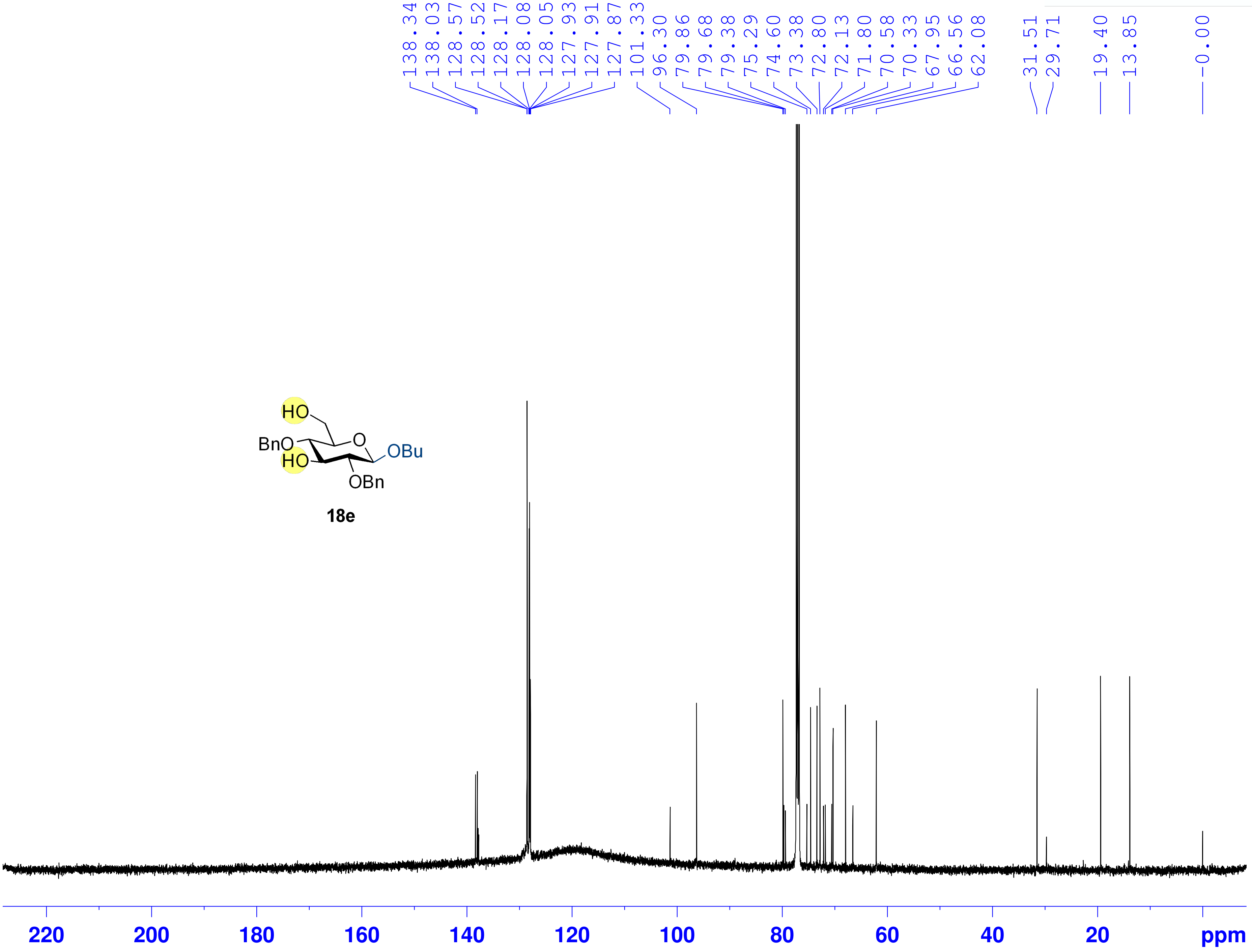

Supplementary Figure 44. 13C-NMR spectrum of compound 18e

	Parameter	Value
1	Solvent	$CDCl_3$
2	Spectrometer Frequency	500 MHz
3	Nucleus	^{1}H

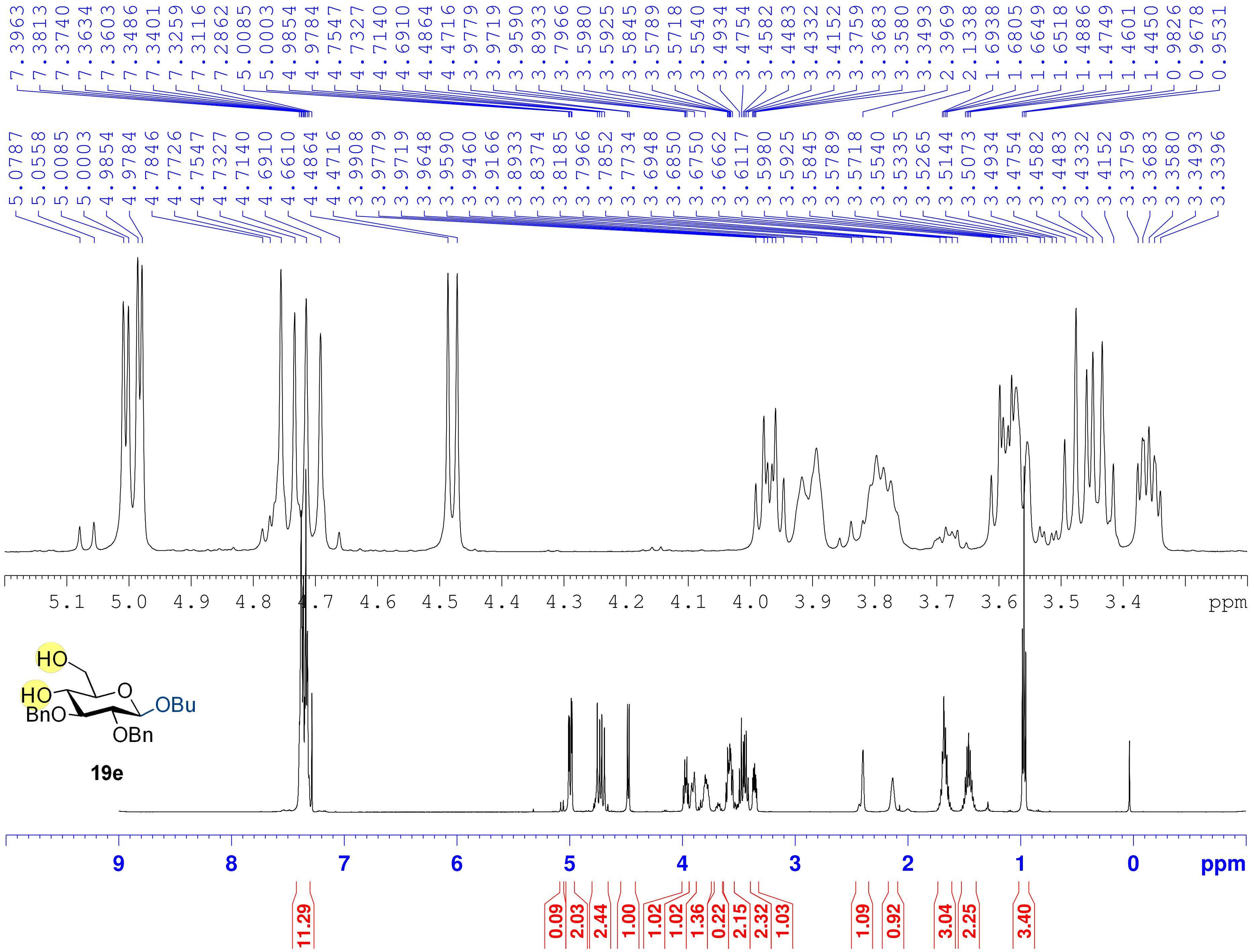

Supplementary Figure 45. 1H-NMR spectrum of compound 19e

	Parameter	Value
1	Solvent	$CDCl_3$
2	Spectrometer Frequency	125 MHz
3	Nucleus	^{13}C

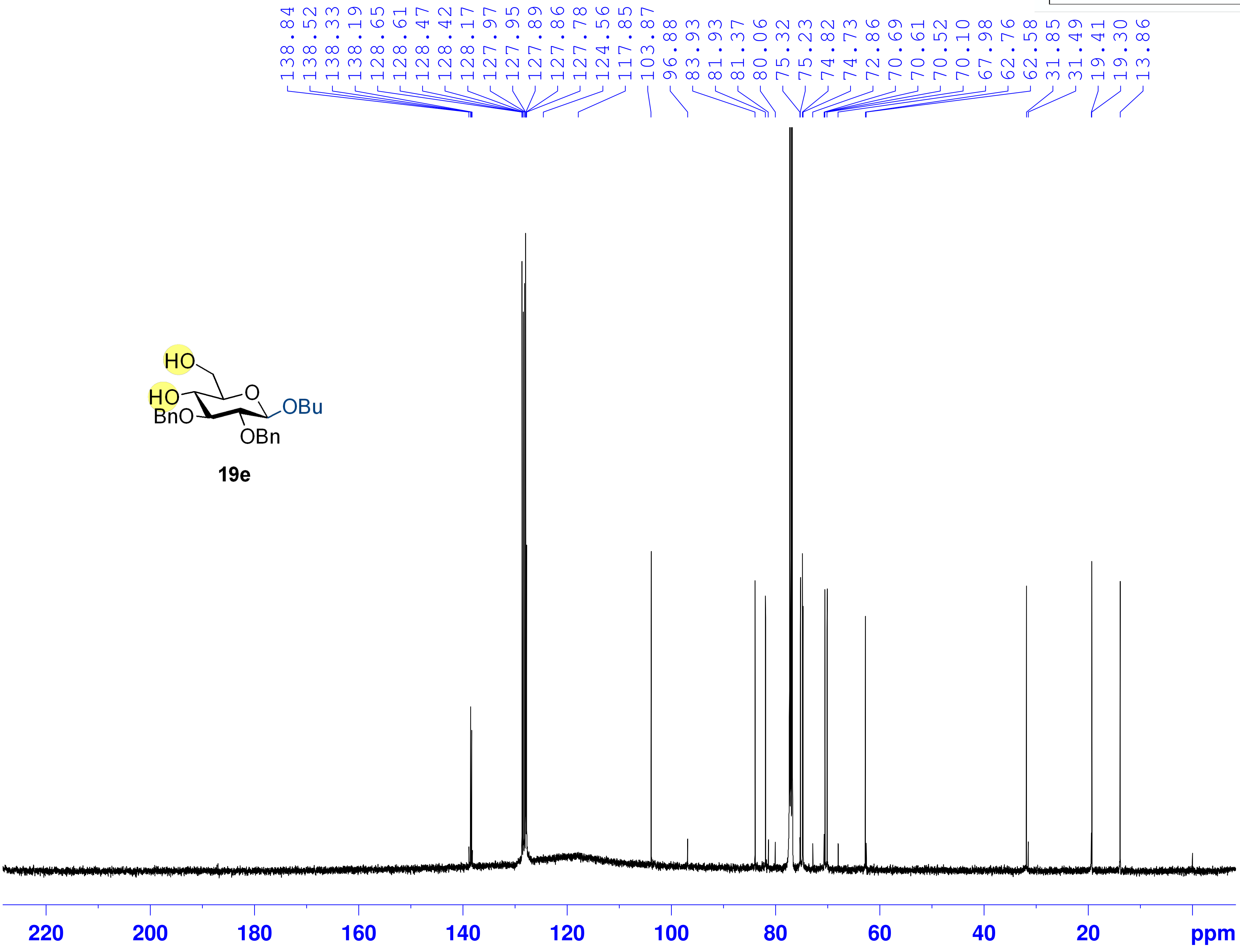

Supplementary Figure 46. 13C-NMR spectrum of compound 19e

	Parameter	Value
1	Solvent	$CDCl_3$
2	Spectrometer Frequency	500 MHz
3	Nucleus	^1H

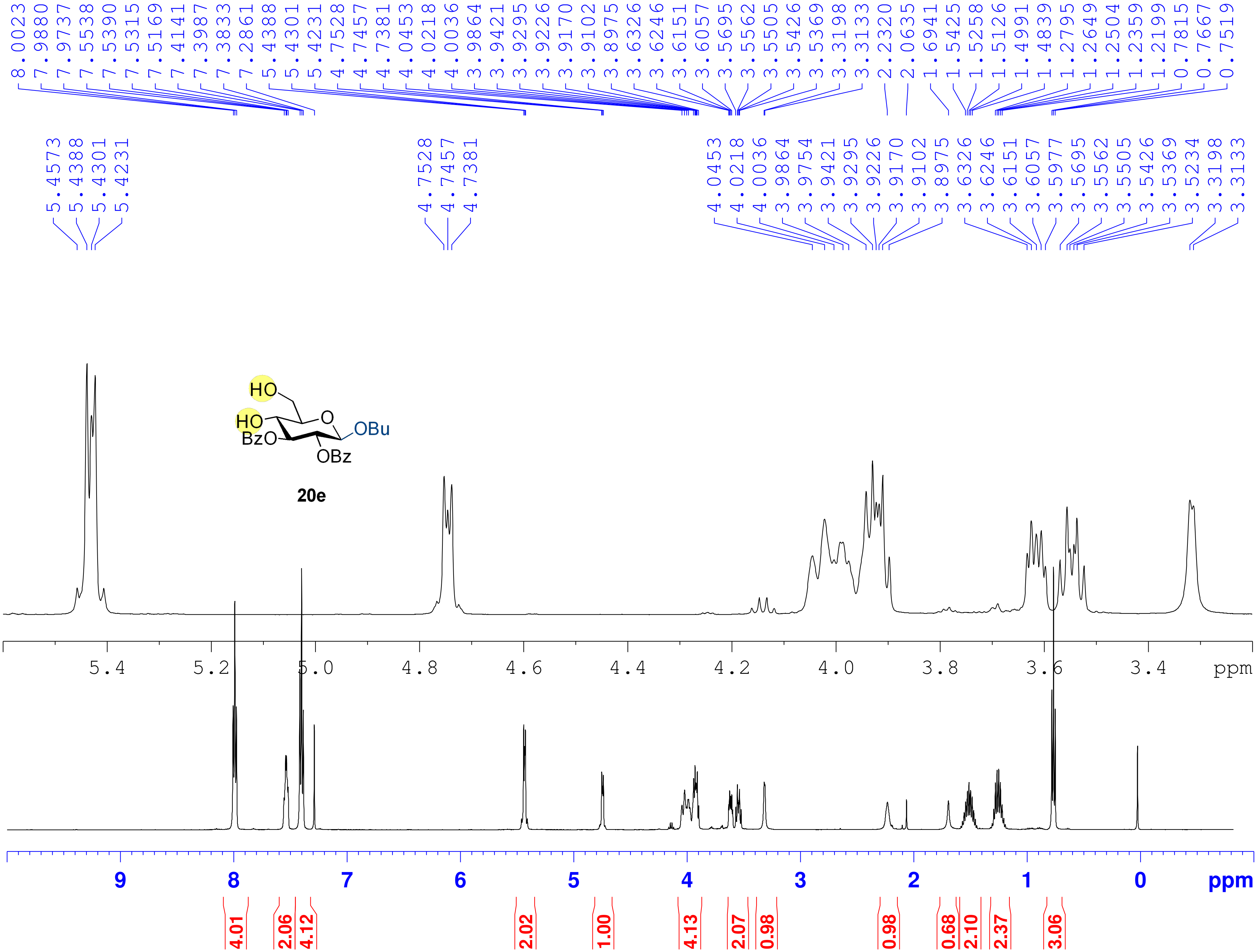

Supplementary Figure 47. 1H-NMR spectrum of compound 20e

	Parameter	Value
1	Solvent	$CDCl_3$
2	Spectrometer Frequency	125 MHz
3	Nucleus	^{13}C

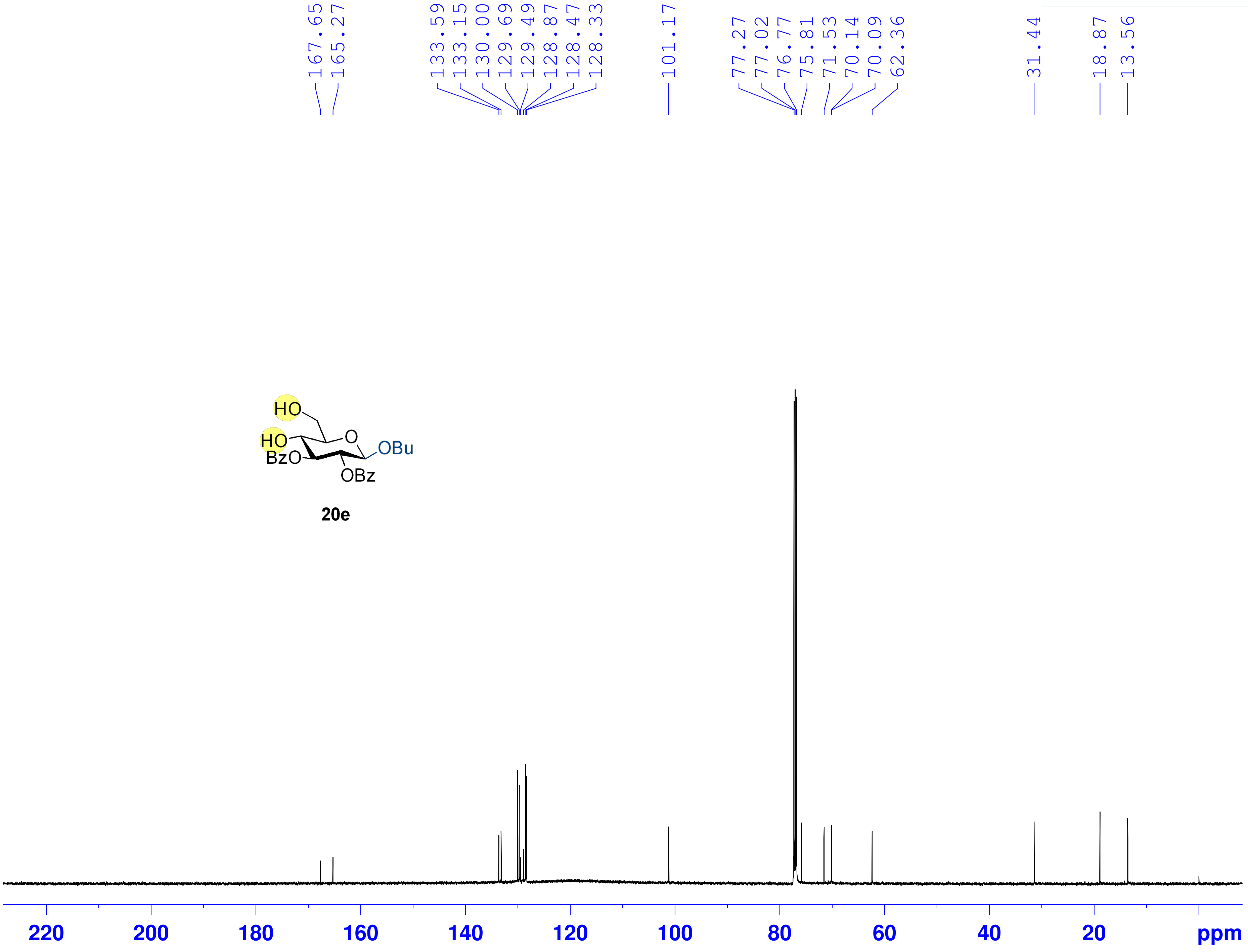

20e

Supplementary Figure 48. 13C-NMR spectrum of compound 20e

	Parameter	Value
1	Solvent	$CDCl_3$
2	Spectrometer Frequency	500 MHz
3	Nucleus	^1H

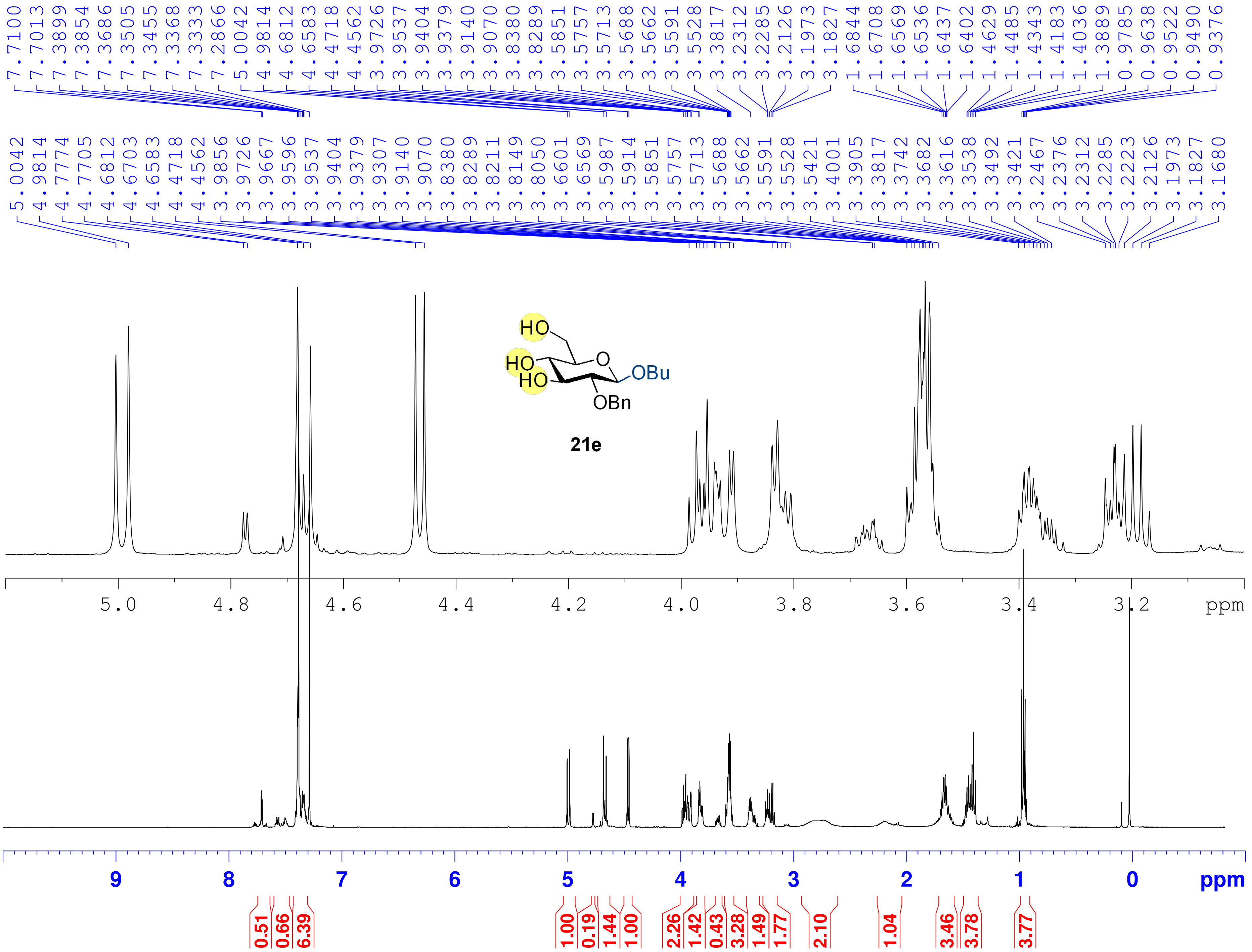

Supplementary Figure 49. 1H-NMR spectrum of compound 21e

	Parameter	Value
1	Solvent	$CDCl_3$
2	Spectrometer Frequency	125 MHz
3	Nucleus	^{13}C

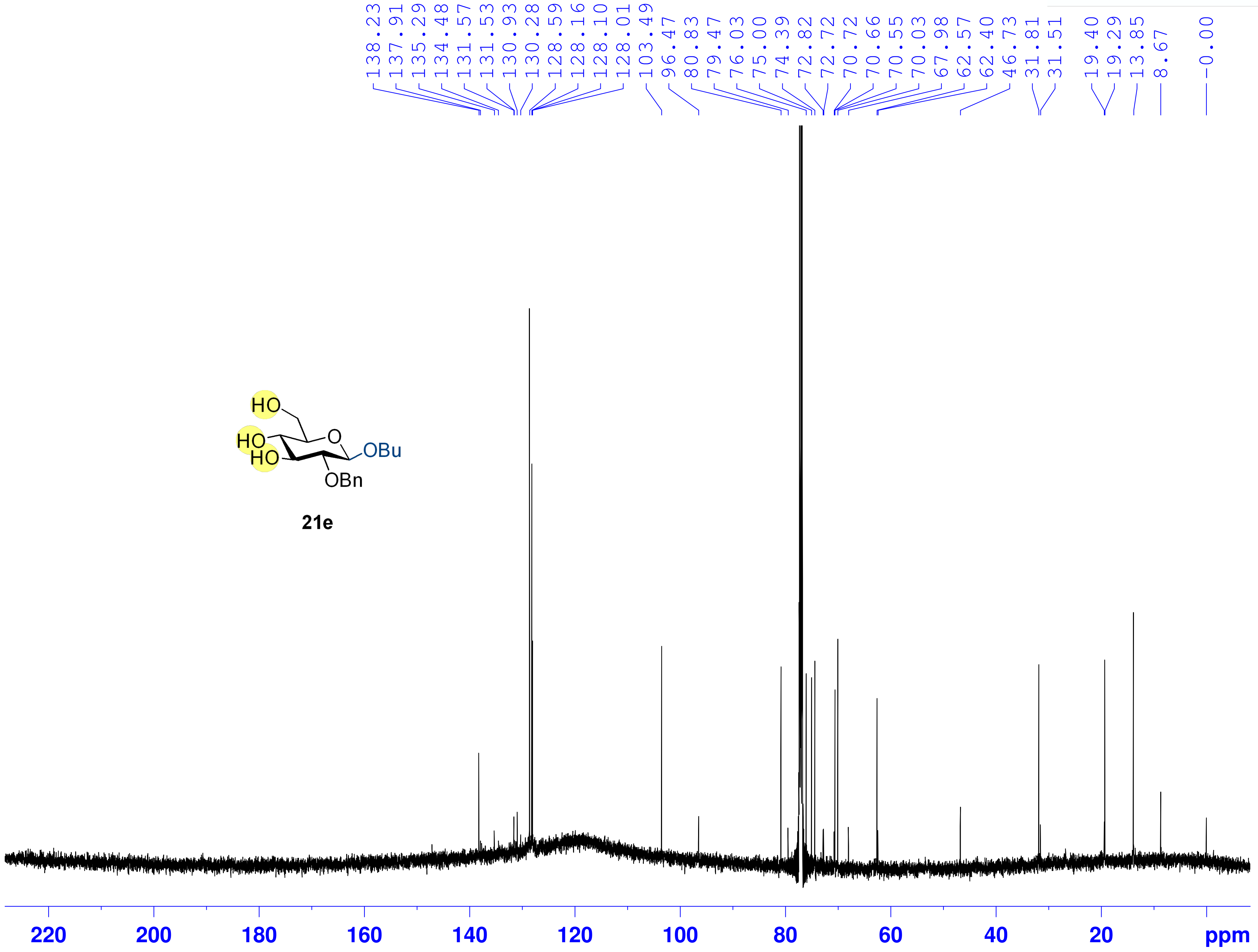

Supplementary Figure 50. 13C-NMR spectrum of compound 21e

	Parameter	Value
1	Solvent	$CDCl_3$
2	Spectrometer Frequency	500 MHz
3	Nucleus	^1H

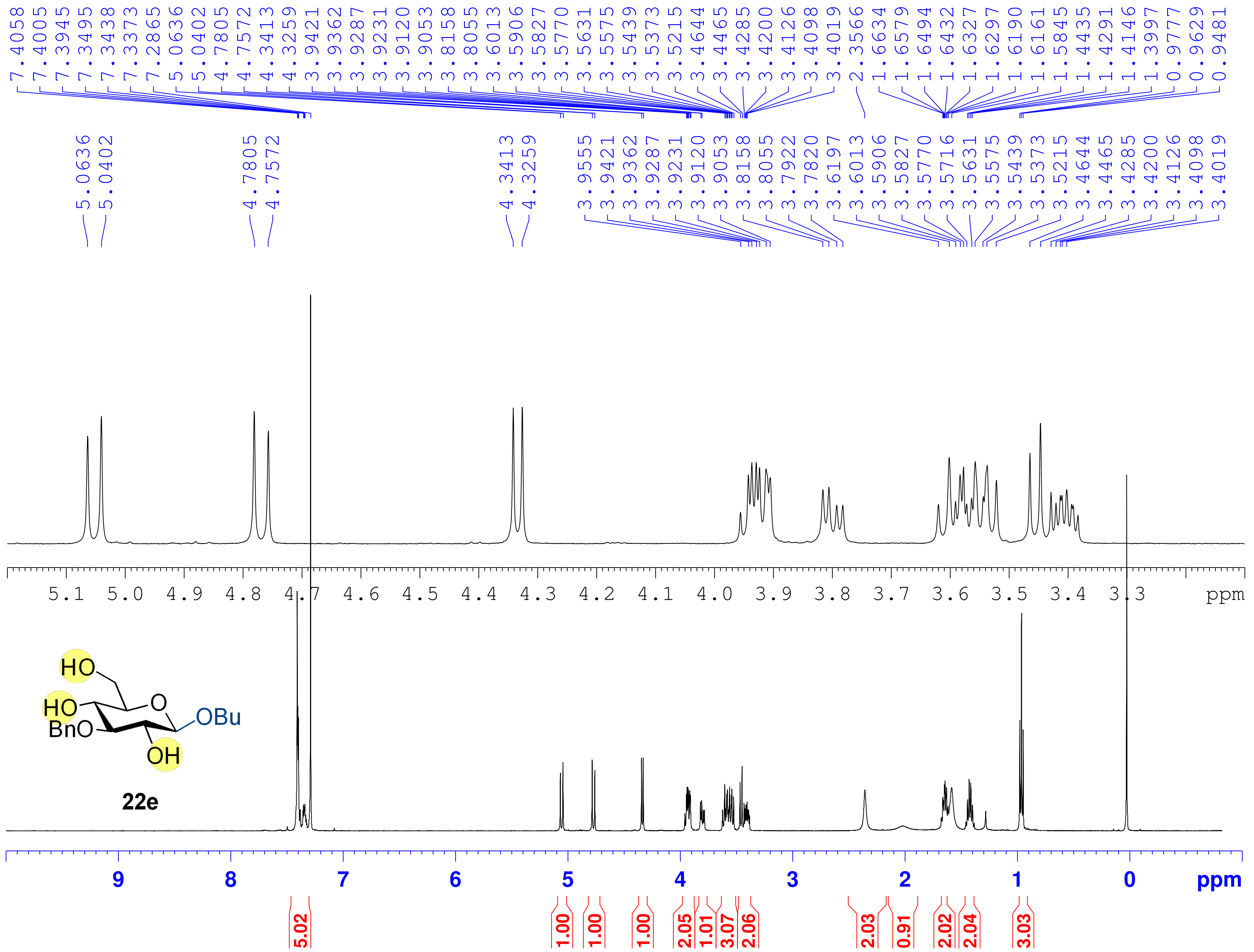

Supplementary Figure 51. 1H-NMR spectrum of compound 22e

	Parameter	Value
1	Solvent	$CDCl_3$
2	Spectrometer Frequency	125 MHz
3	Nucleus	^{13}C

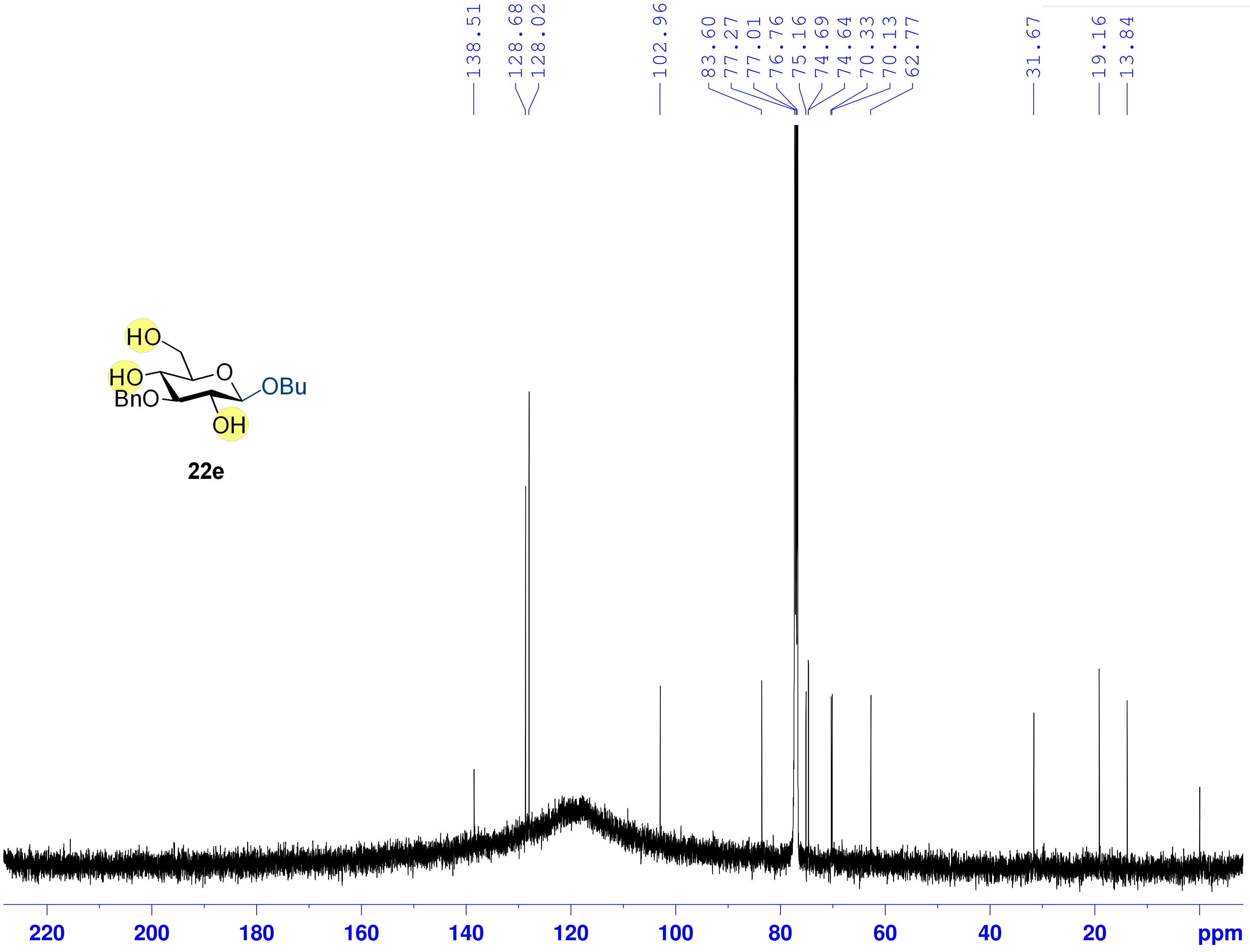

Supplementary Figure 52. 13C-NMR spectrum of compound 22e

	Parameter	Value
1	Solvent	$CDCl_3$
2	Spectrometer Frequency	500 MHz
3	Nucleus	^1H

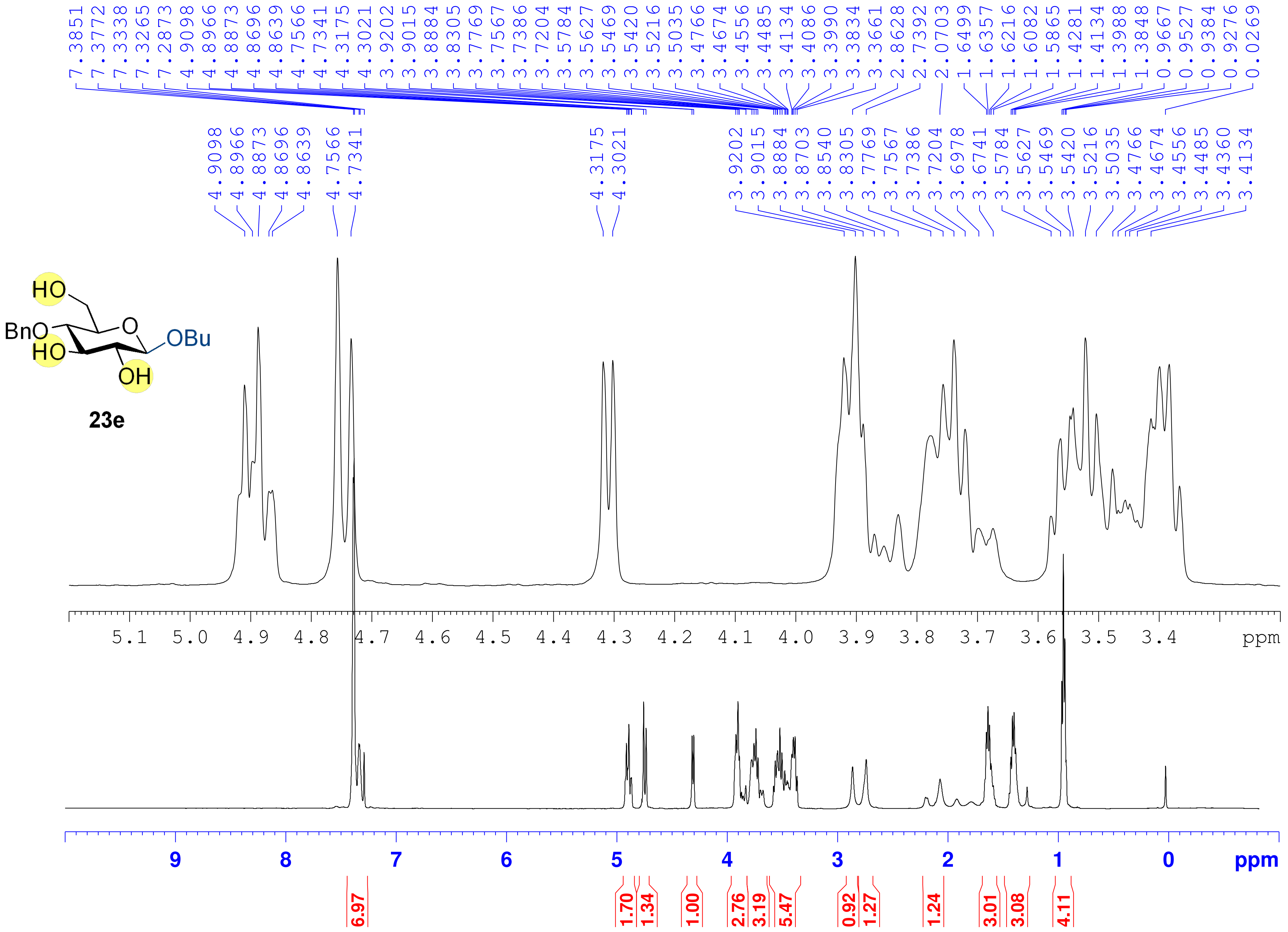

Supplementary Figure 53. 1H-NMR spectrum of compound 23e

	Parameter	Value
1	Solvent	$CDCl_3$
2	Spectrometer Frequency	125 MHz
3	Nucleus	^{13}C

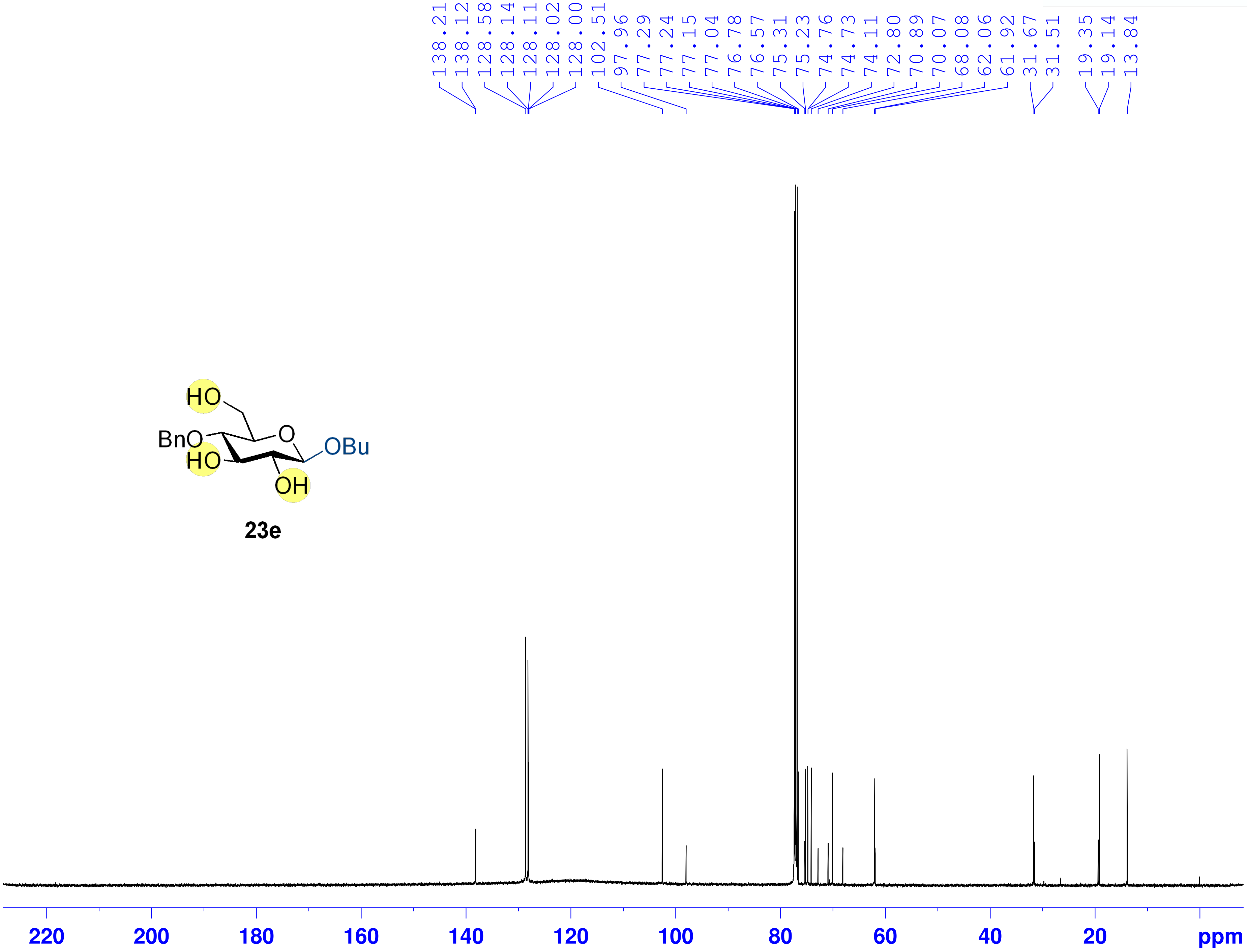

Supplementary Figure 54. 13C-NMR spectrum of compound 23e

	Parameter	Value
1	Solvent	$CDCl_3$
2	Spectrometer Frequency	500 MHz
3	Nucleus	^1H

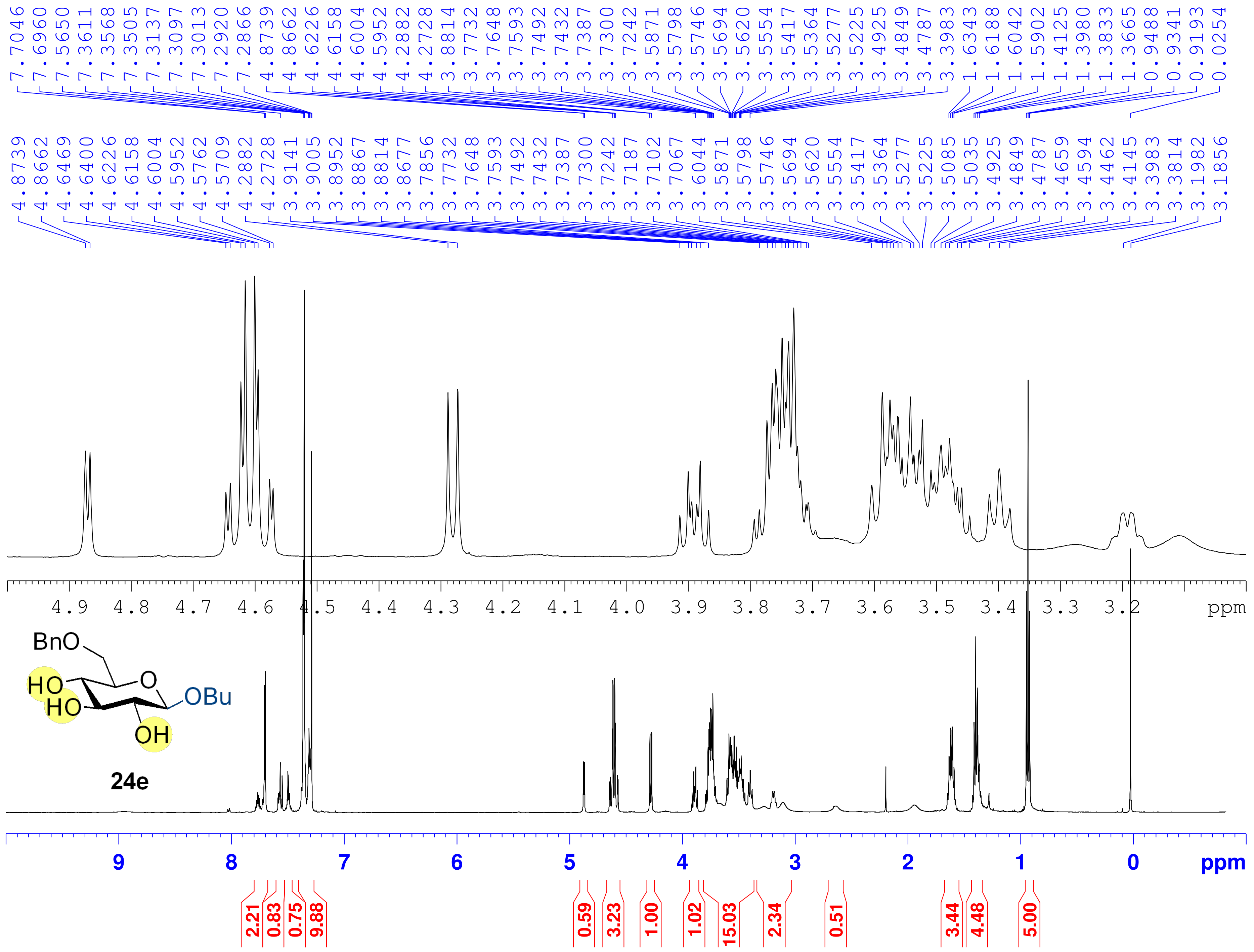

Supplementary Figure 55. 1H-NMR spectrum of compound 24e

	Parameter	Value
1	Solvent	$CDCl_3$
2	Spectrometer Frequency	125 MHz
3	Nucleus	^{13}C

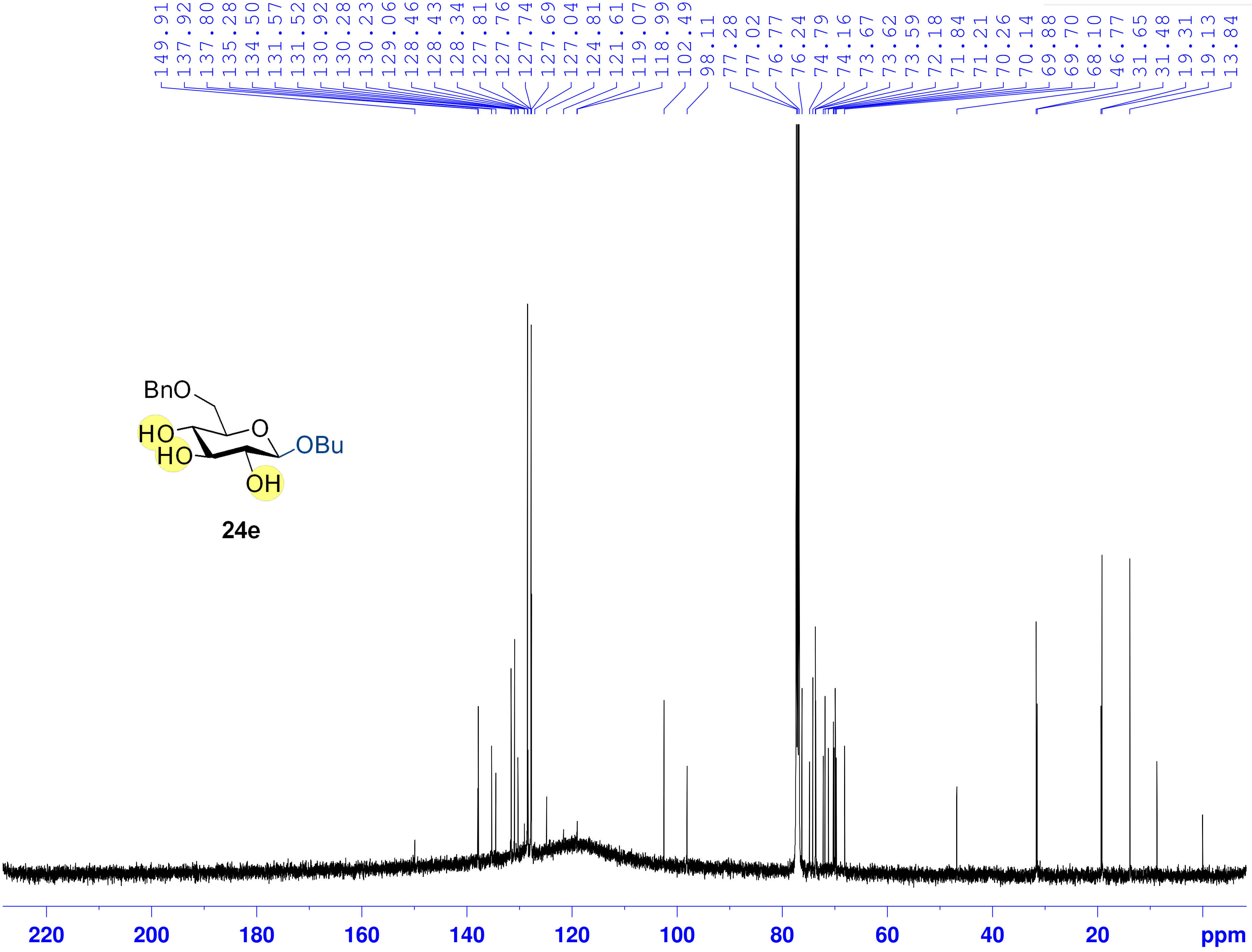

Supplementary Figure 56. 13C-NMR spectrum of compound 24e

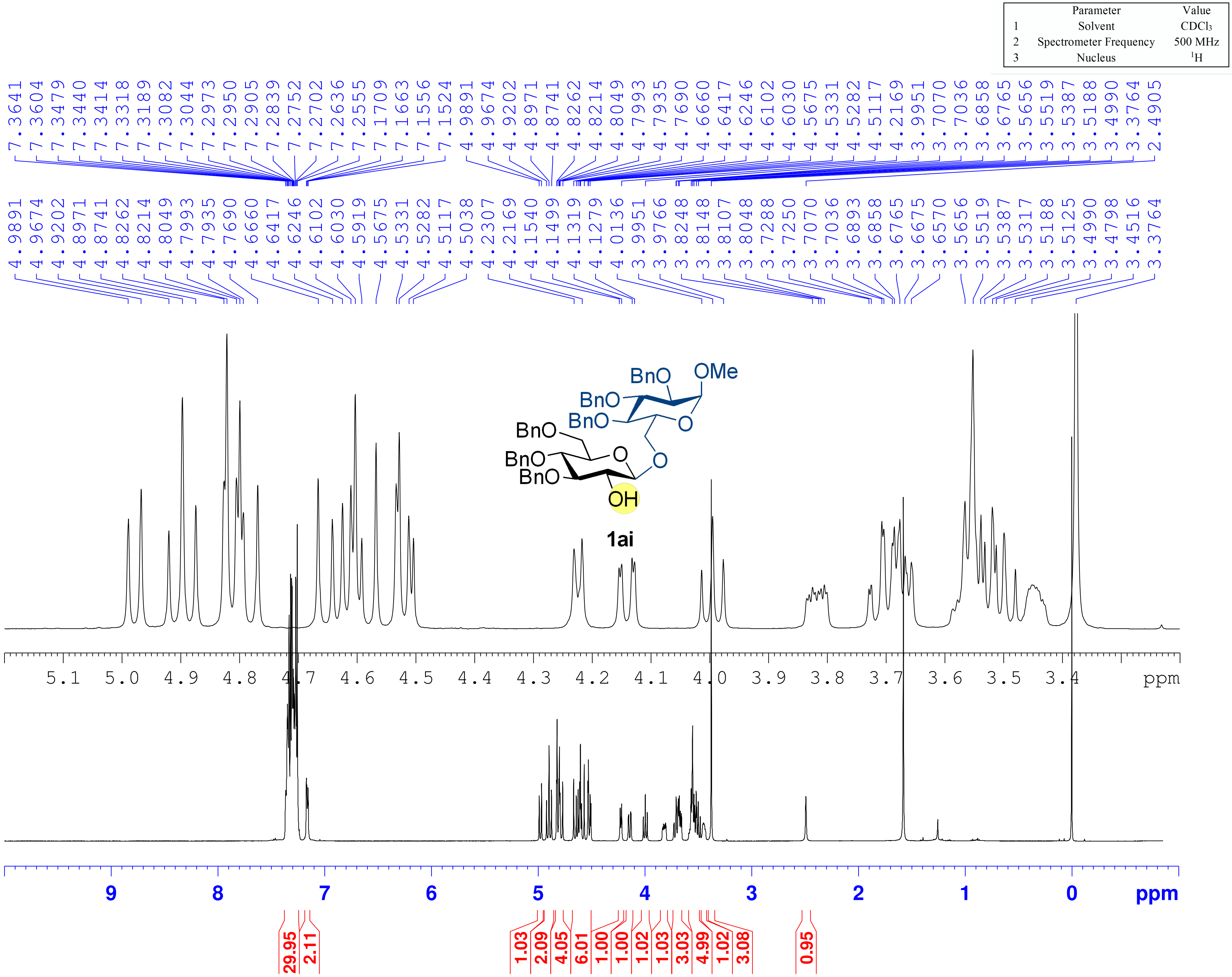

Supplementary Figure 57. 1H-NMR spectrum of compound 1ai

	Parameter	Value
1	Solvent	$CDCl_3$
2	Spectrometer Frequency	125 MHz
3	Nucleus	^{13}C

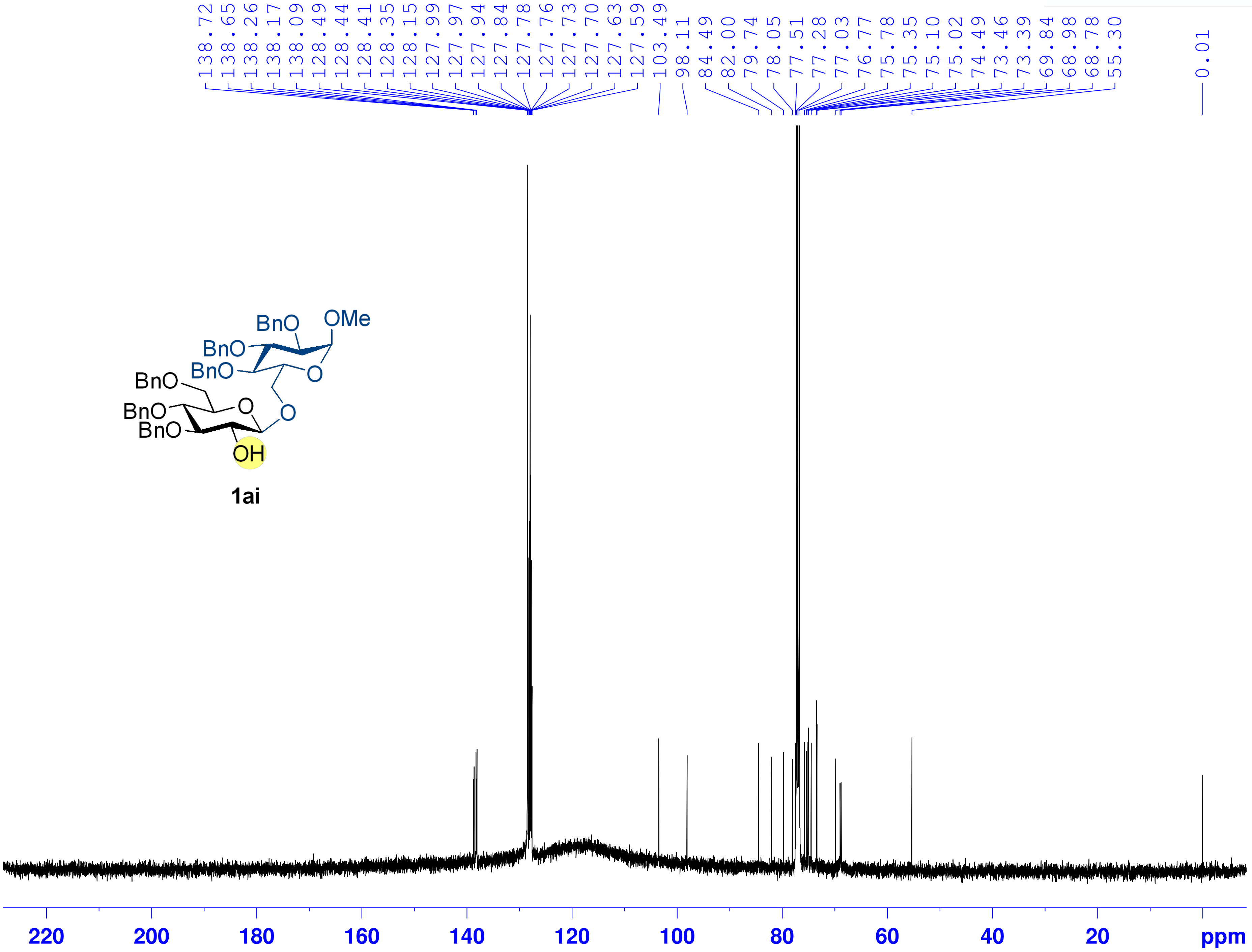

Supplementary Figure 58. 13C-NMR spectrum of compound 1ai

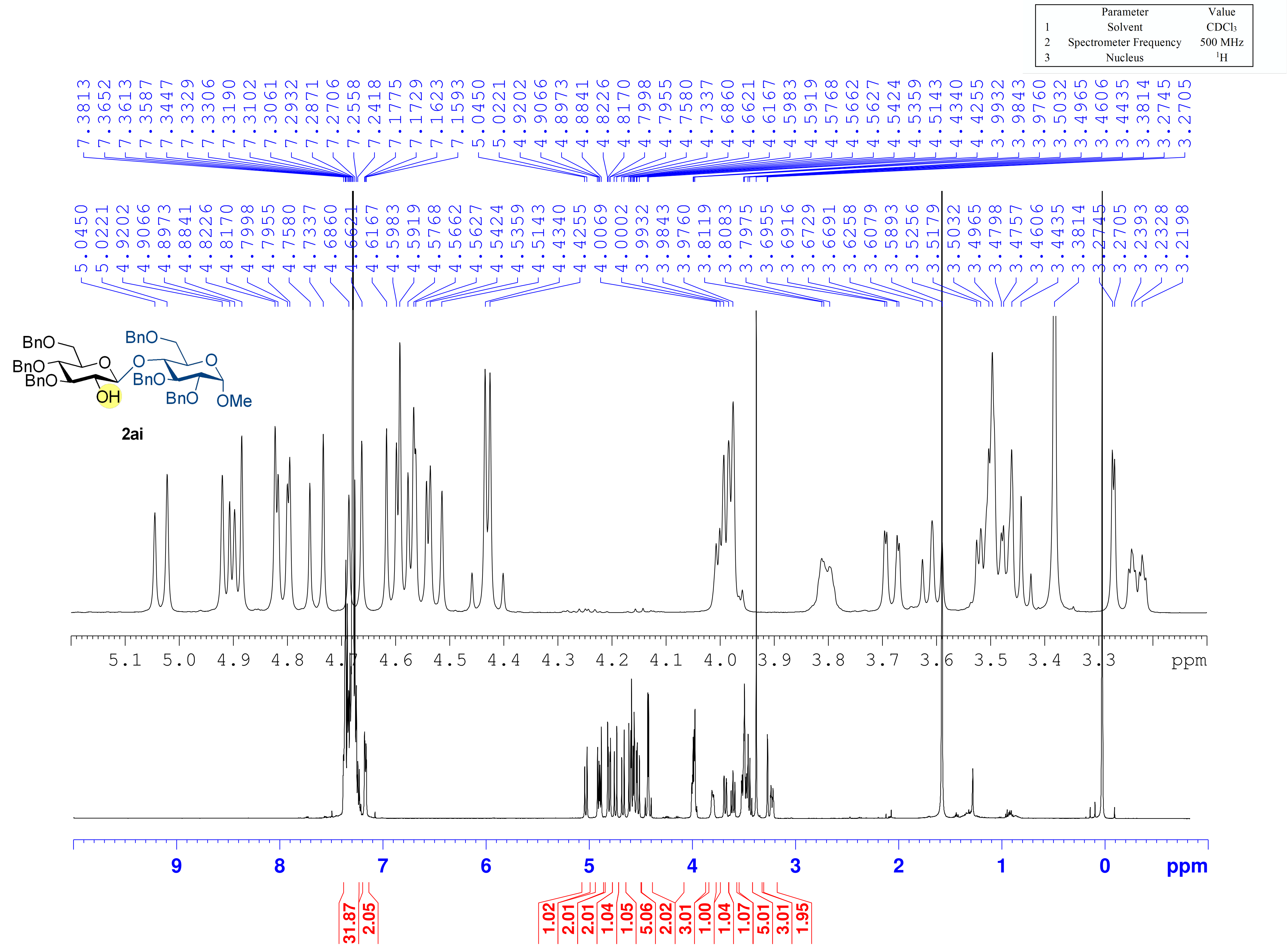

Supplementary Figure 59. 1H-NMR spectrum of compound 2ai

	Parameter	Value
1	Solvent	$CDCl_3$
2	Spectrometer Frequency	125 MHz
3	Nucleus	^{13}C

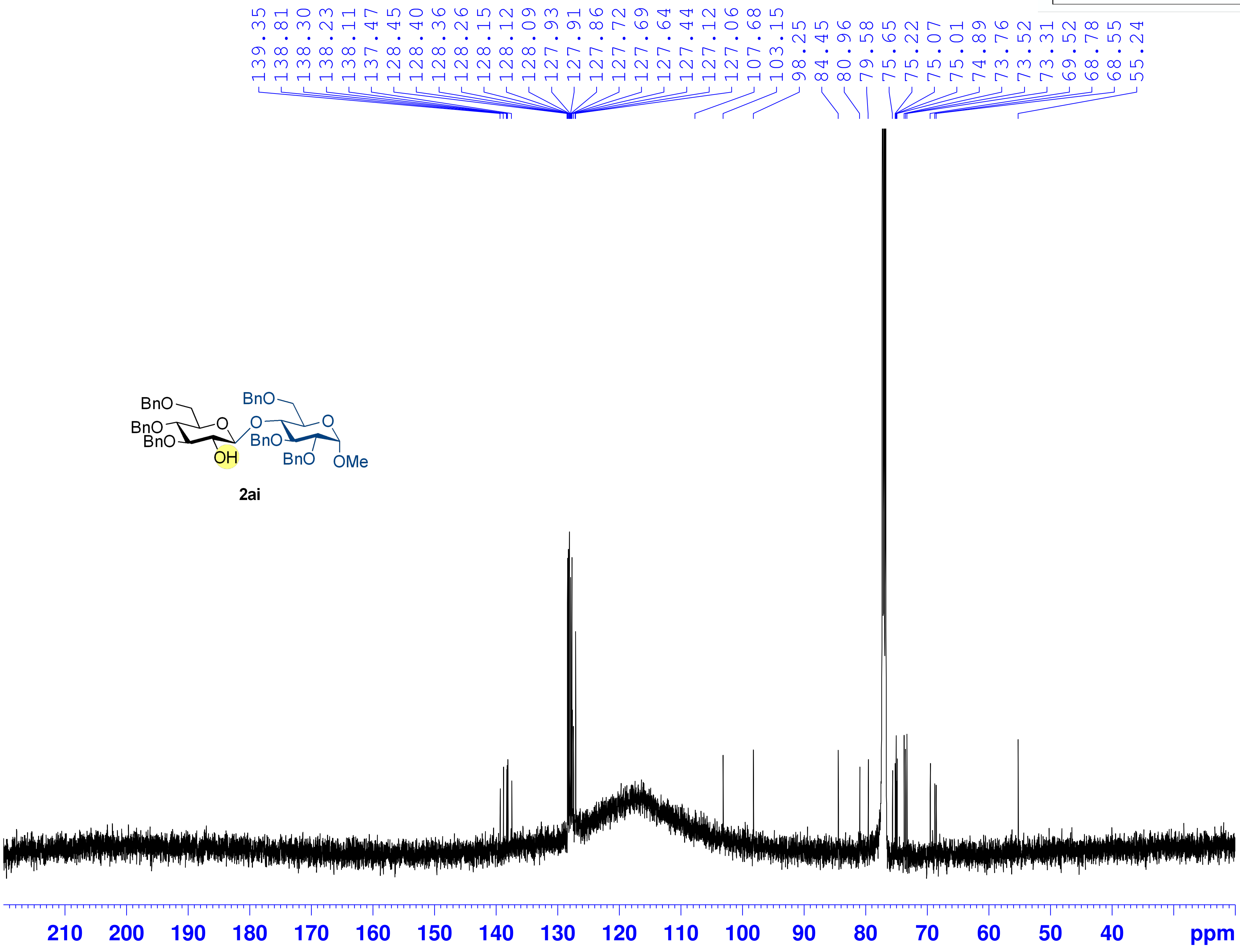

Supplementary Figure 60. 13C-NMR spectrum of compound 2ai

	Parameter	Value
1	Solvent	$CDCl_3$
2	Spectrometer Frequency	400 MHz
3	Nucleus	^{1}H

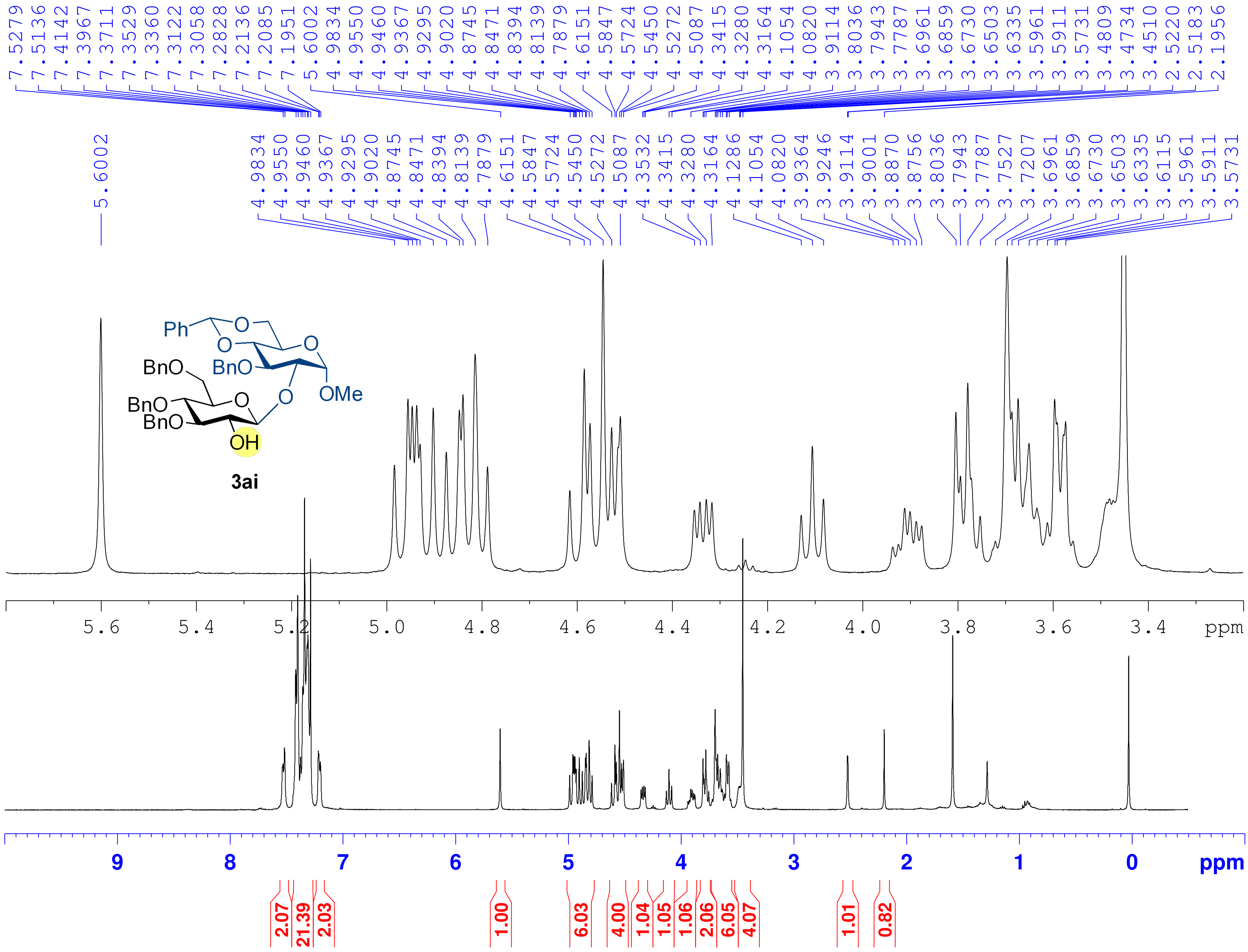

Supplementary Figure 61. 1H-NMR spectrum of compound 3ai

	Parameter	Value
1	Solvent	$CDCl_3$
2	Spectrometer Frequency	100 MHz
3	Nucleus	^{13}C

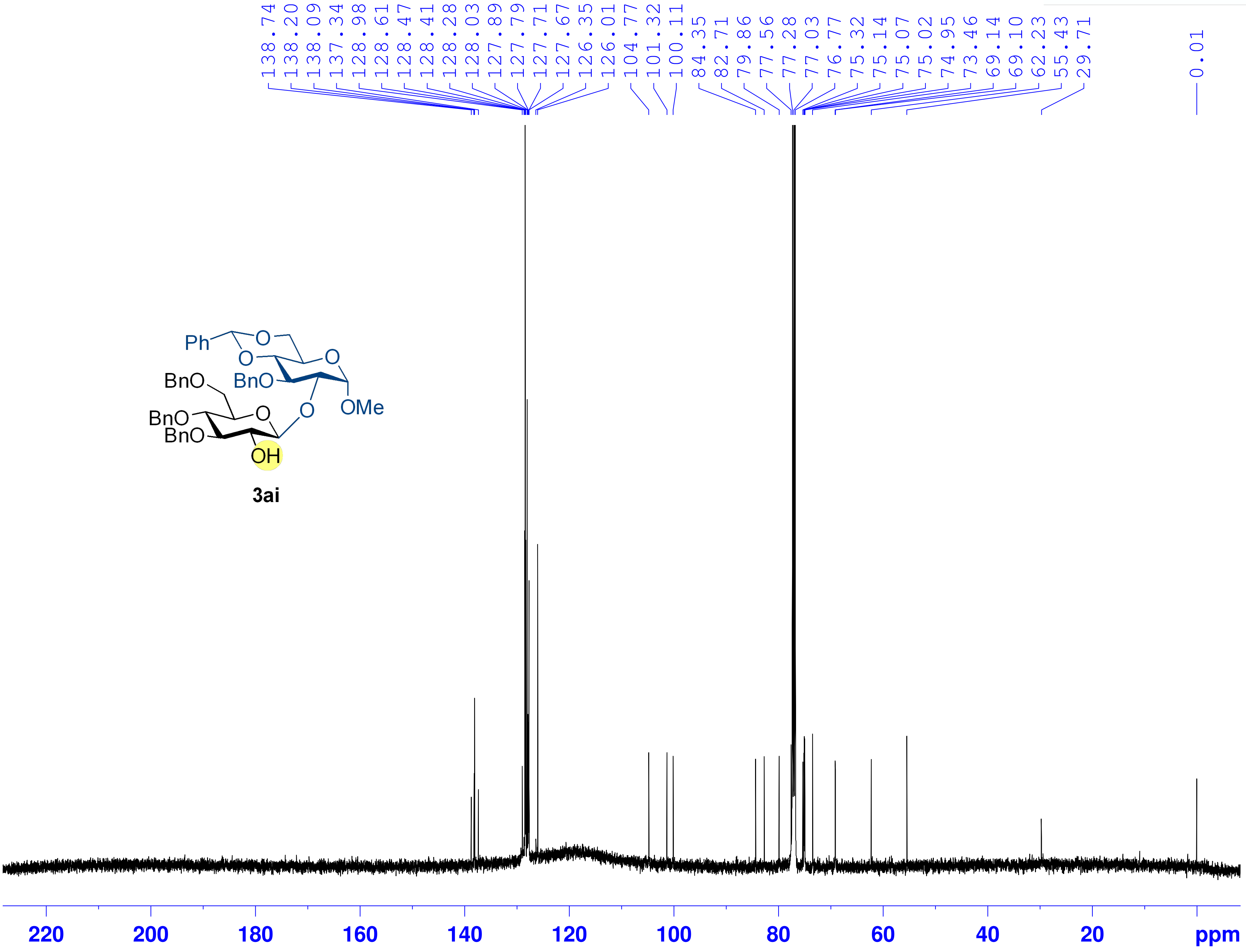

Supplementary Figure 64. 13C-NMR spectrum of compound 3ai

	Parameter	Value
1	Solvent	$CDCl_3$
2	Spectrometer Frequency	500 MHz
3	Nucleus	^1H

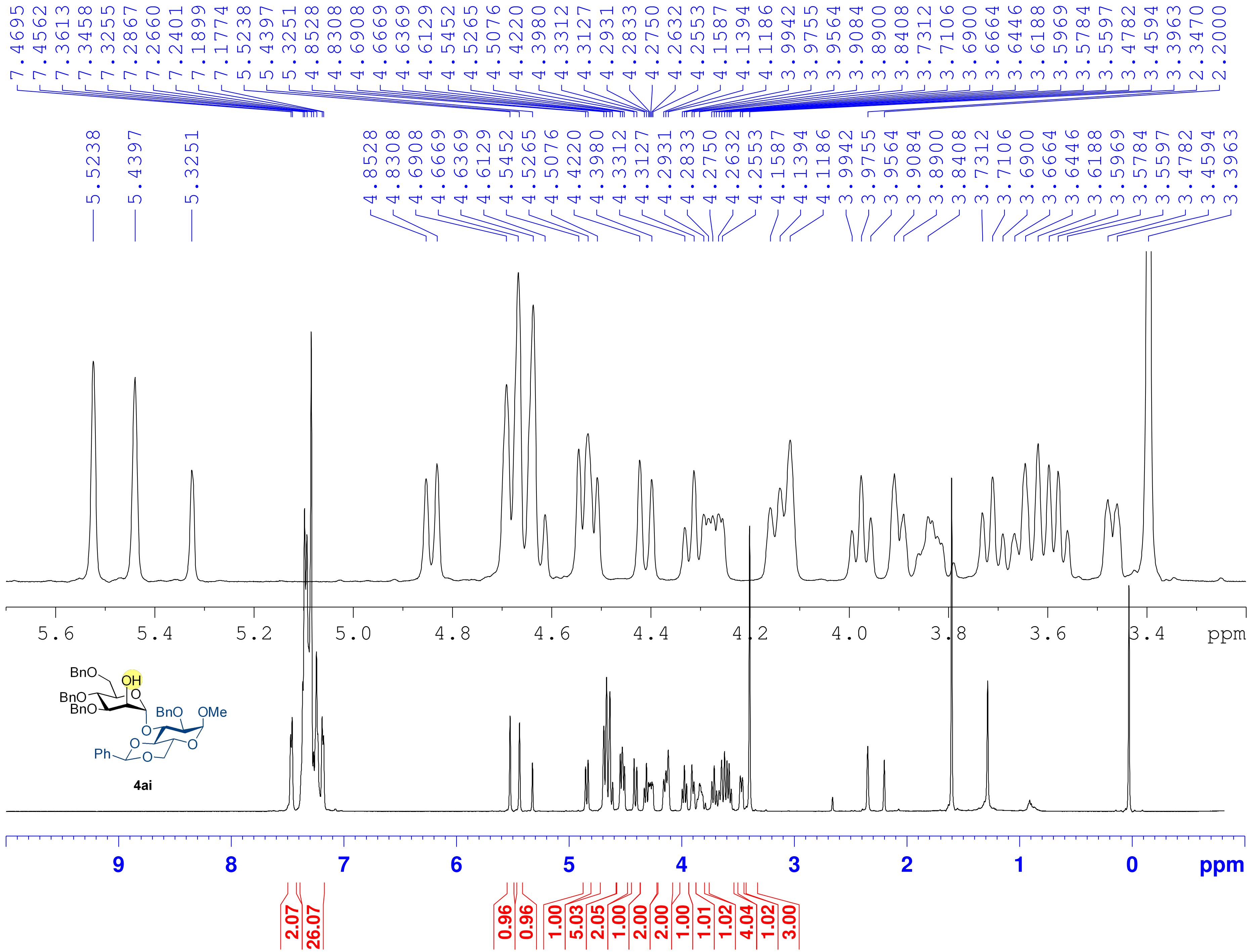

Supplementary Figure 63. 1H-NMR spectrum of compound 4ai

	Parameter	Value
1	Solvent	$CDCl_3$
2	Spectrometer Frequency	125 MHz
3	Nucleus	^{13}C

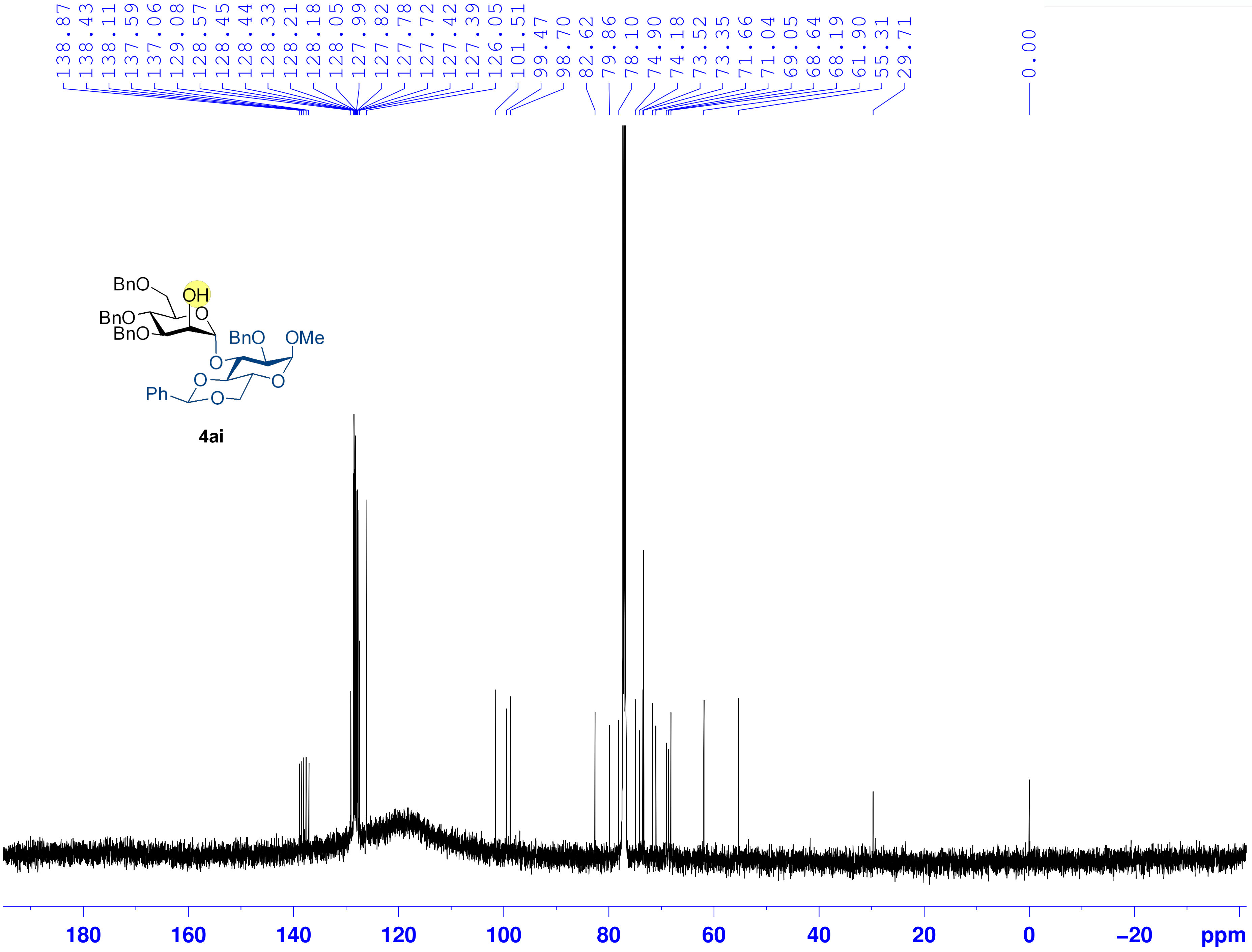

Supplementary Figure 64. 13C-NMR spectrum of compound 4ai

	Parameter	Value
1	Solvent	$CDCl_3$
2	Spectrometer Frequency	500 MHz
3	Nucleus	^{1}H

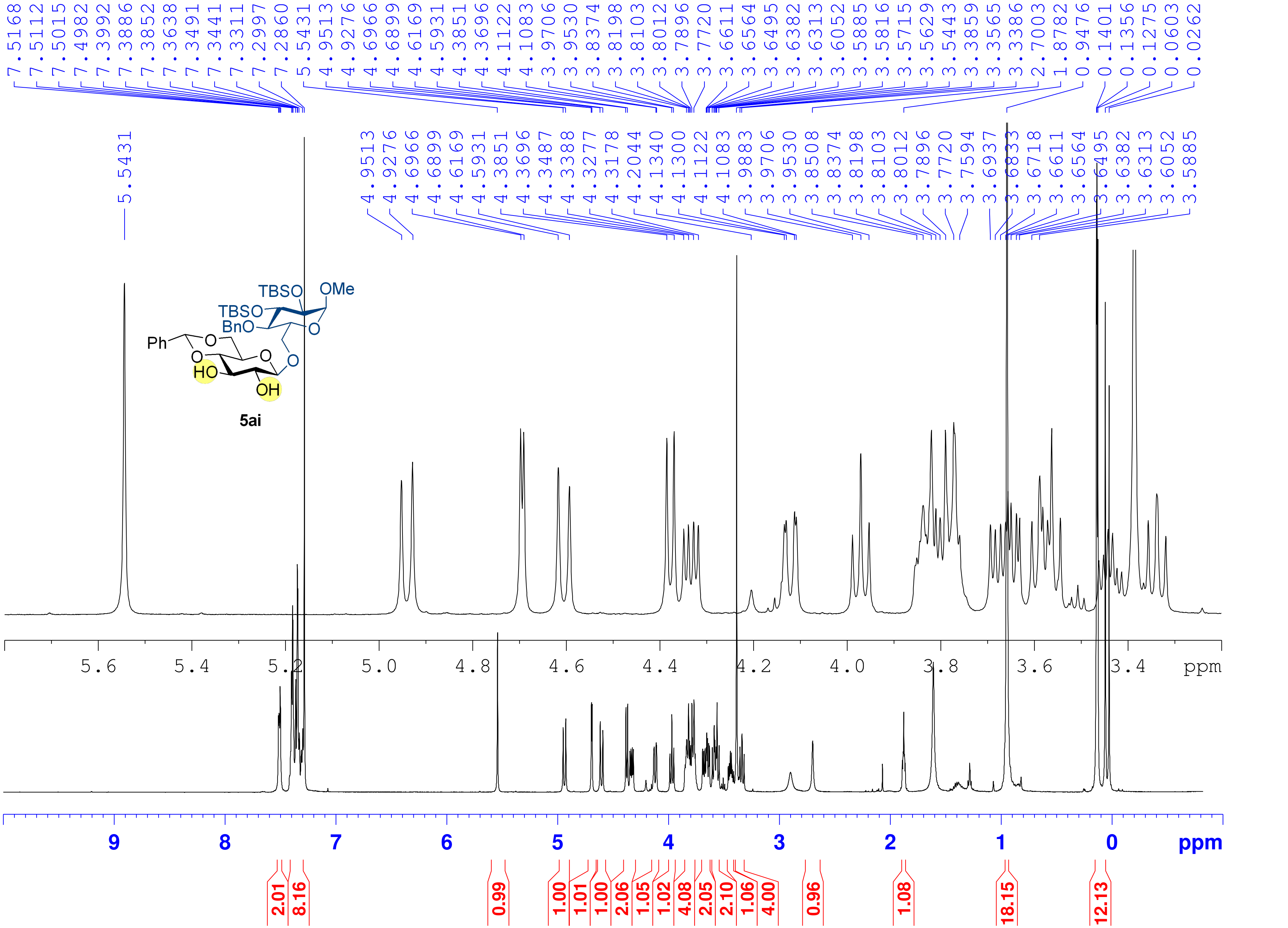

Supplementary Figure 65. 1H-NMR spectrum of compound 5ai

	Parameter	Value
1	Solvent	$CDCl_3$
2	Spectrometer Frequency	125 MHz
3	Nucleus	^{13}C

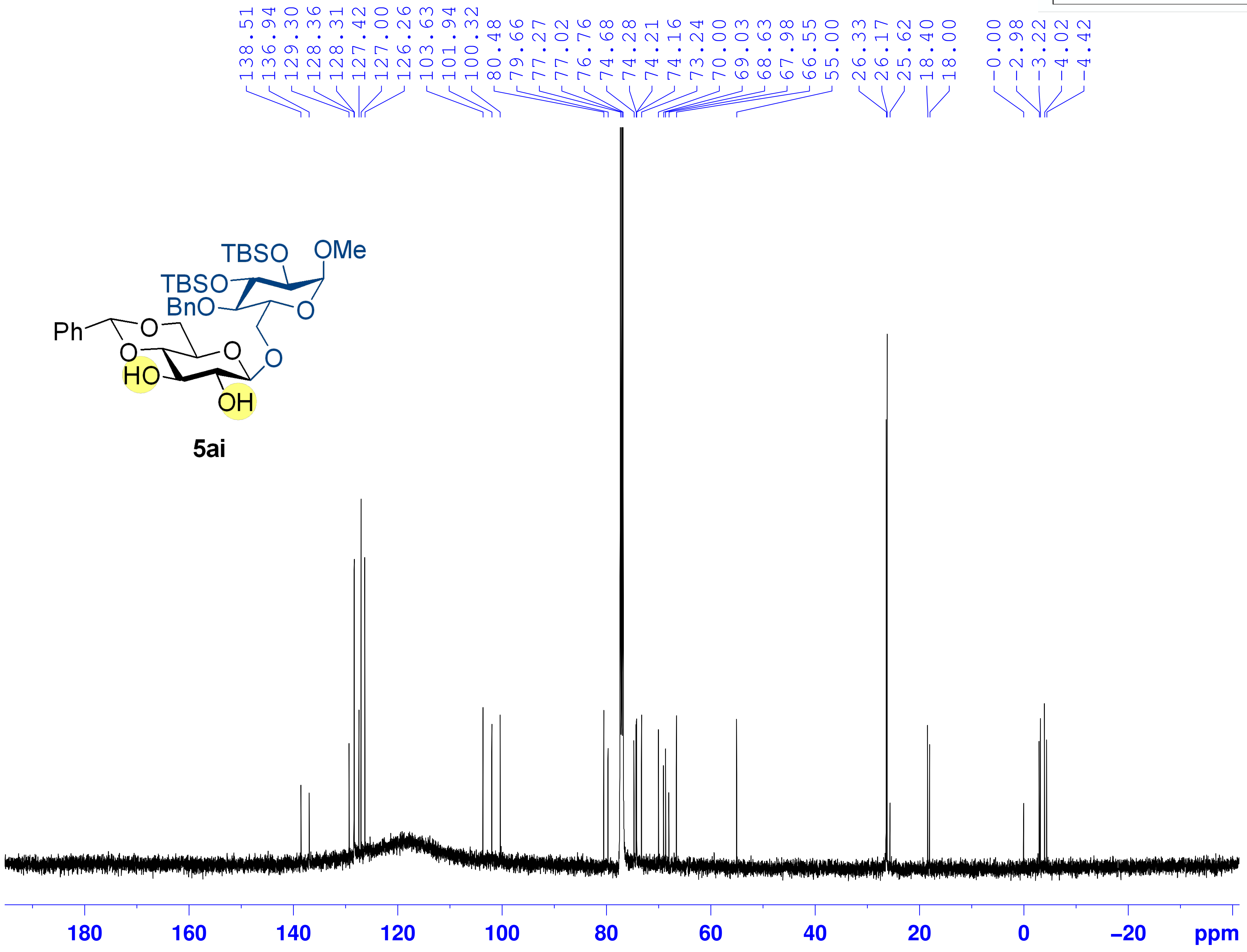

Supplementary Figure 66. 13C-NMR spectrum of compound 5ai

	Parameter	Value
1	Solvent	$CDCl_3$
2	Spectrometer Frequency	400 MHz
3	Nucleus	^1H

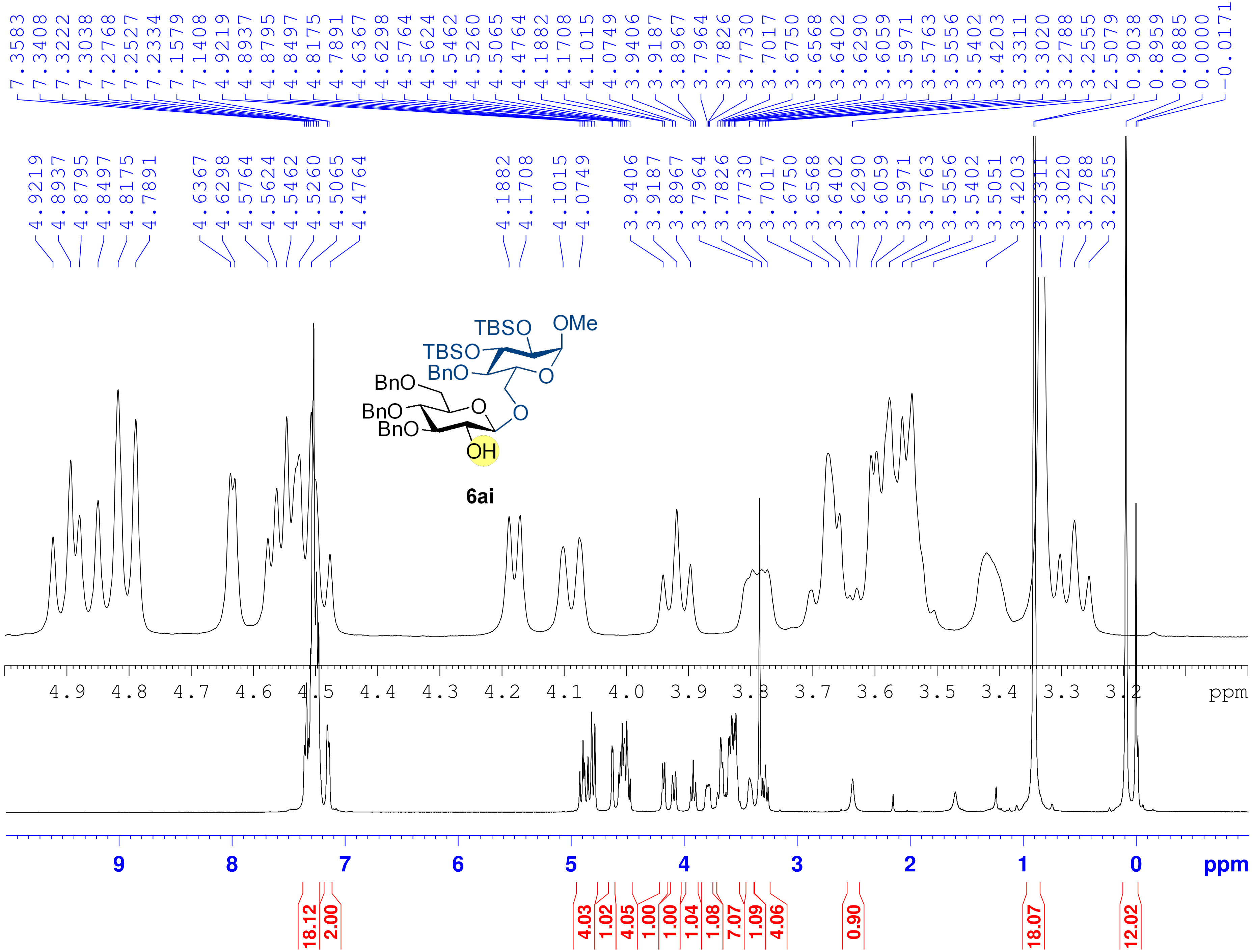

Supplementary Figure 67. 1H-NMR spectrum of compound 6ai

	Parameter	Value
1	Solvent	$CDCl_3$
2	Spectrometer Frequency	100 MHz
3	Nucleus	^{13}C

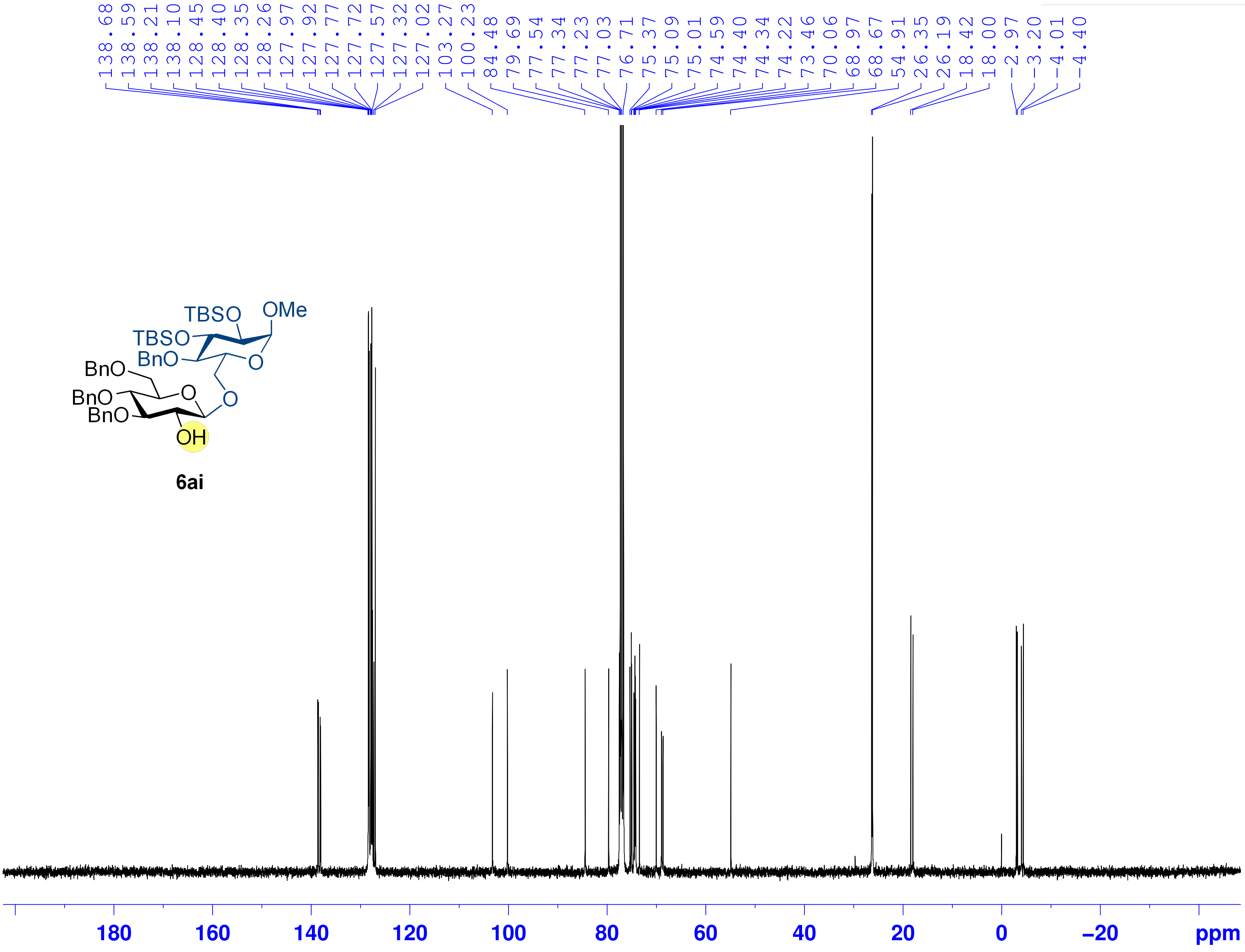

Supplementary Figure 68. 13C-NMR spectrum of compound 6ai

	Parameter	Value
1	Solvent	$CDCl_3$
2	Spectrometer Frequency	500 MHz
3	Nucleus	^{1}H

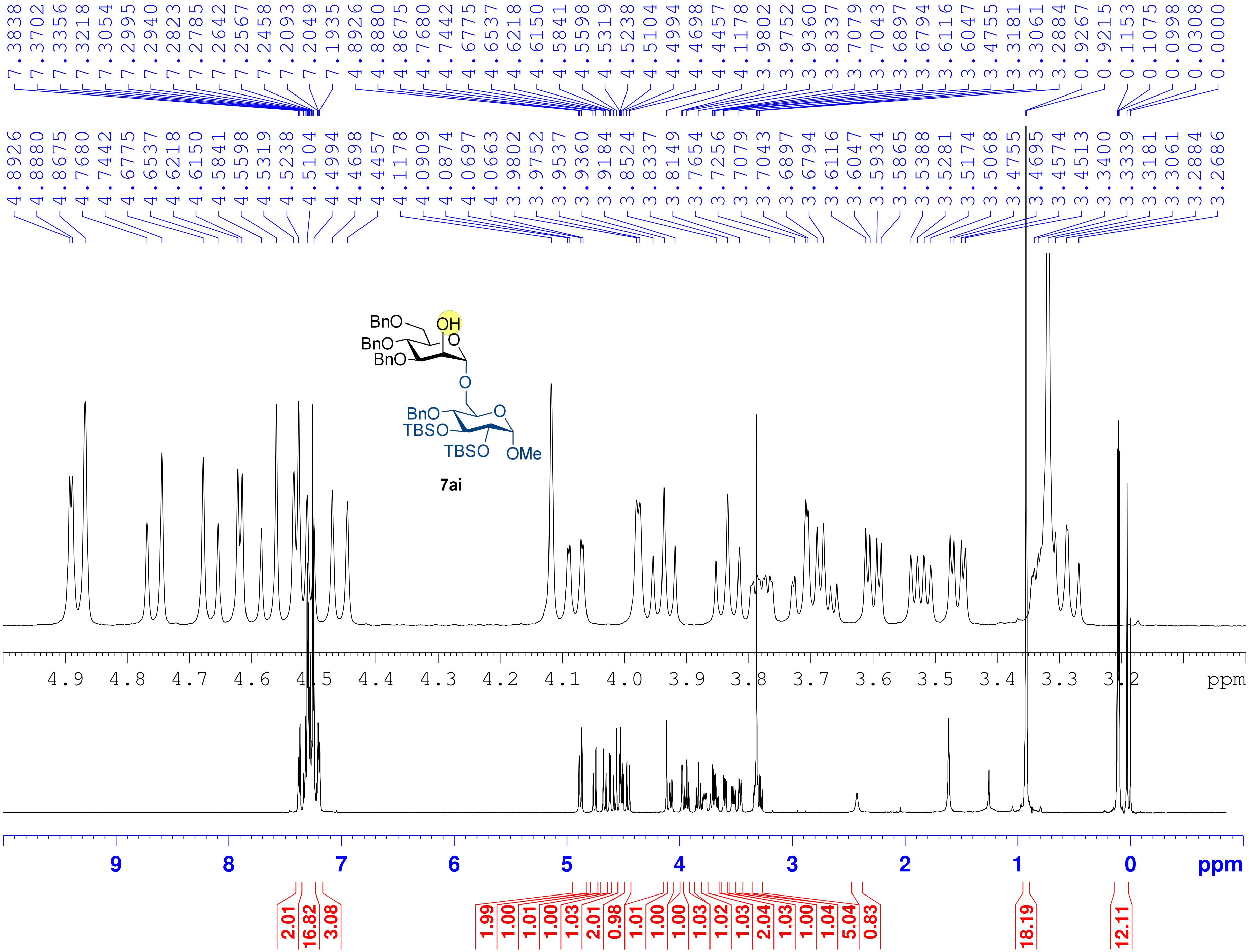

Supplementary Figure 69. 1H-NMR spectrum of compound 7ai

	Parameter	Value
1	Solvent	$CDCl_3$
2	Spectrometer Frequency	125 MHz
3	Nucleus	^{13}C

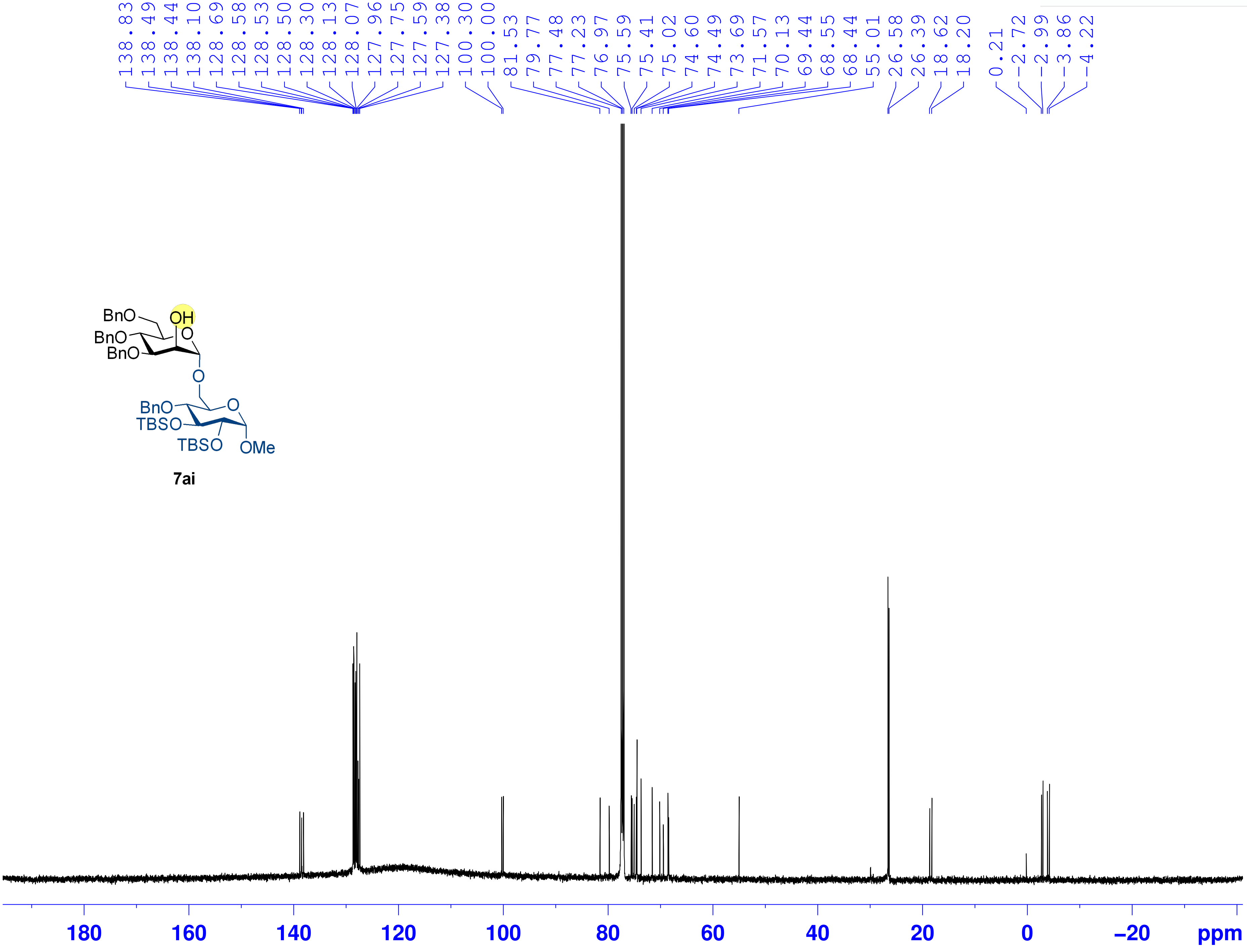

Supplementary Figure 70. 13C-NMR spectrum of compound 7ai

	Parameter	Value
1	Solvent	$CDCl_3$
2	Spectrometer Frequency	500 MHz
3	Nucleus	^{1}H

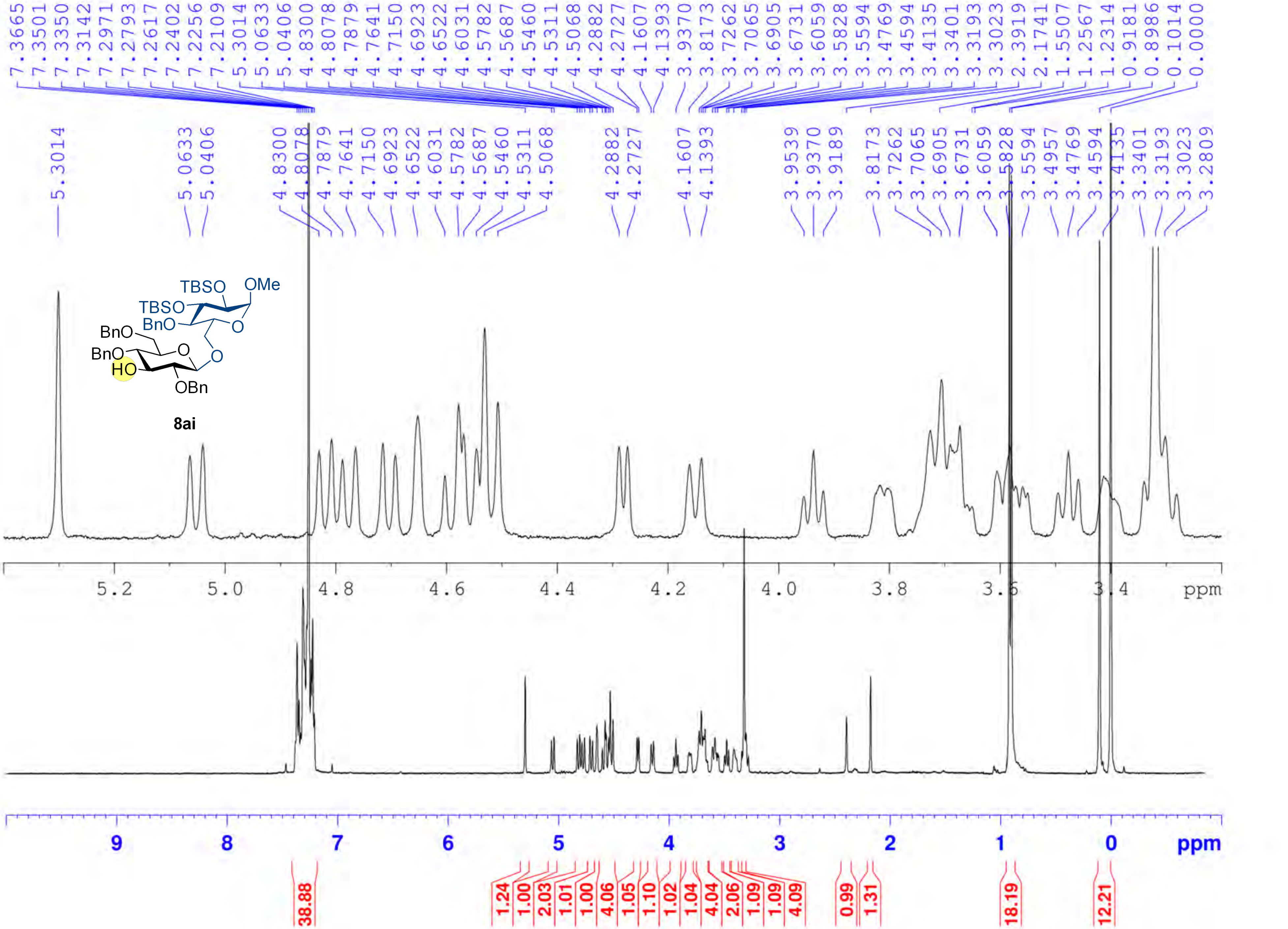

Supplementary Figure 71. 1H-NMR spectrum of compound 8ai

	Parameter	Value
1	Solvent	$CDCl_3$
2	Spectrometer Frequency	125 MHz
3	Nucleus	^{13}C

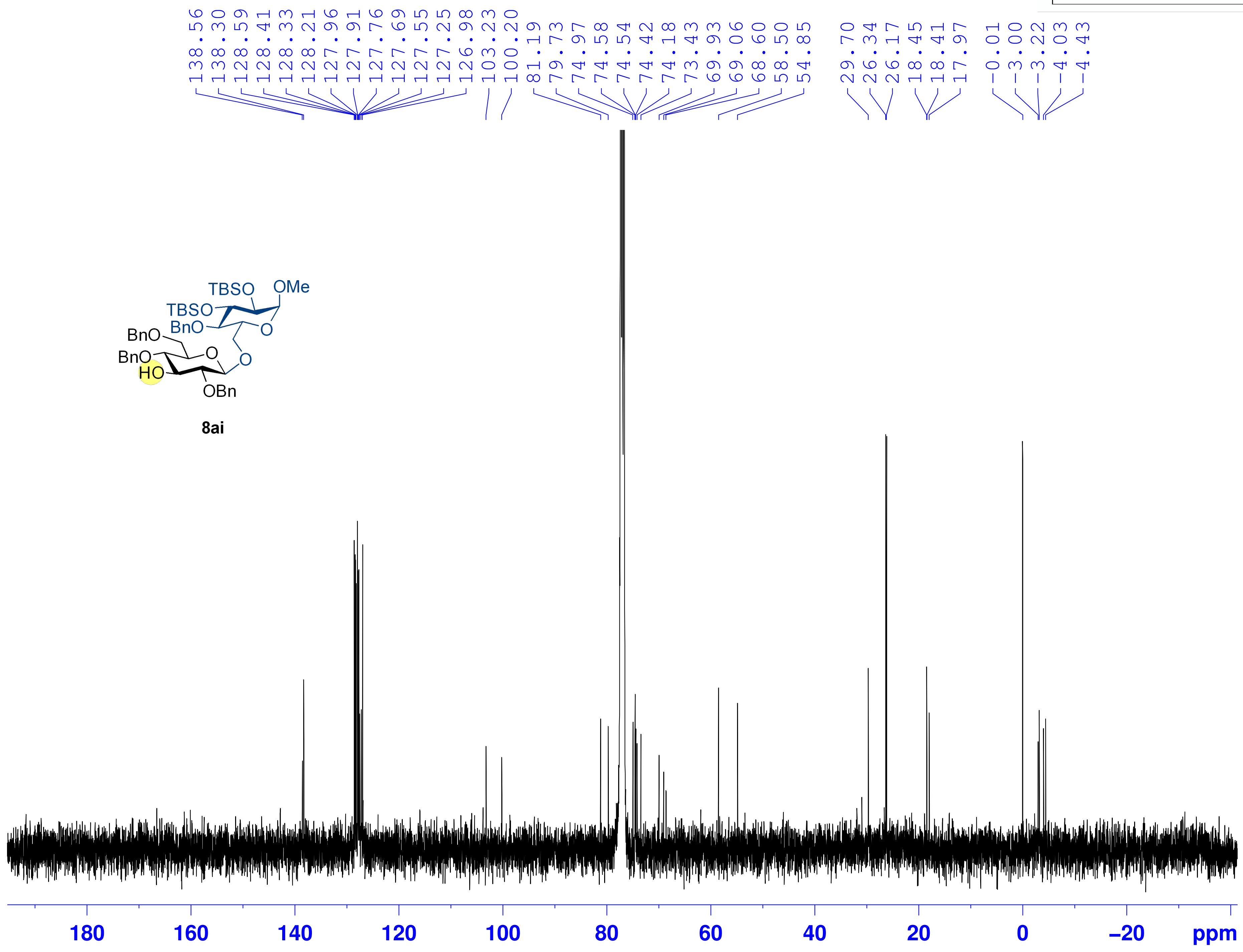

Supplementary Figure 72. 13C-NMR spectrum of compound 8ai

	Parameter	Value
1	Solvent	$CDCl_3$
2	Spectrometer Frequency	400 MHz
3	Nucleus	^{1}H

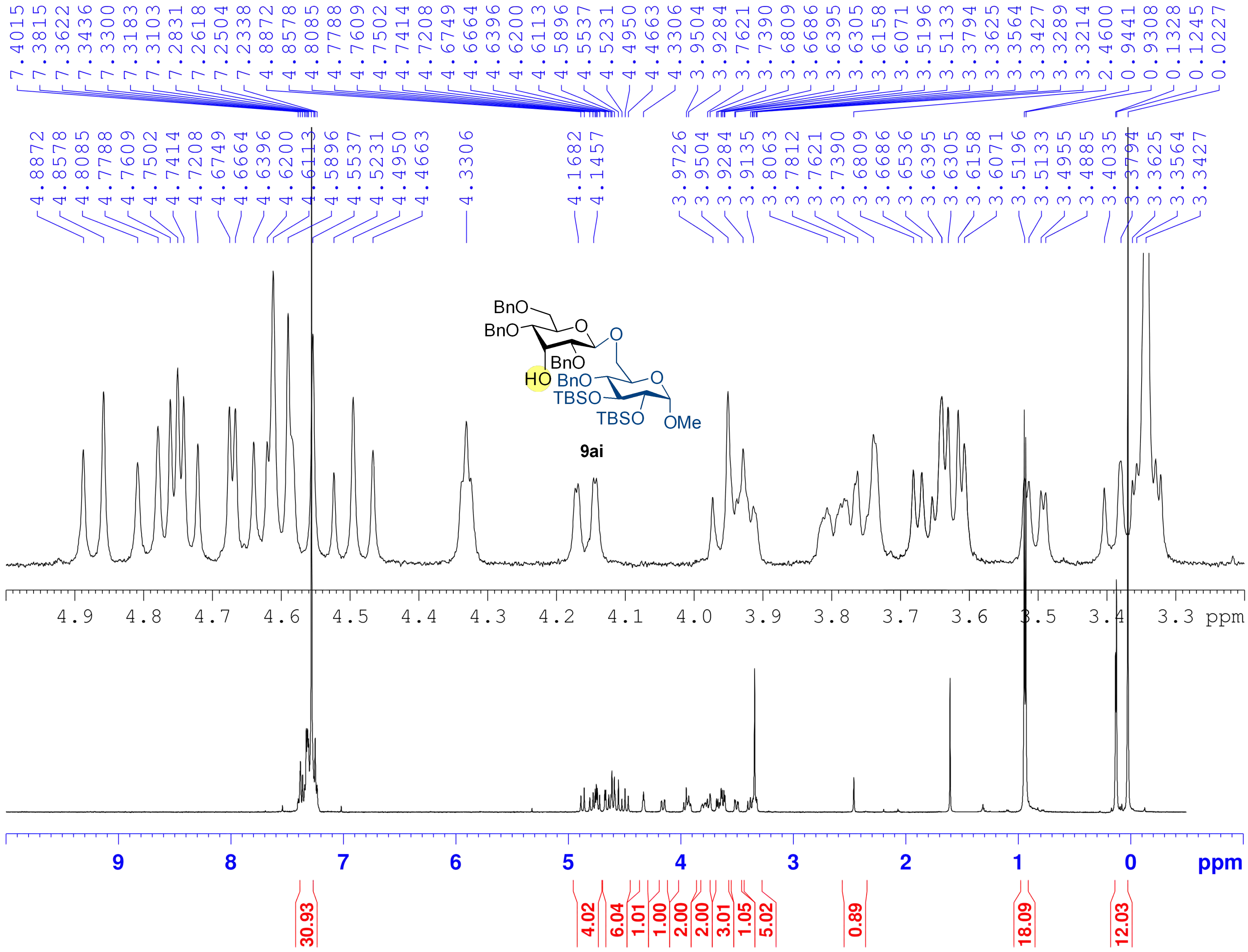

Supplementary Figure 73. 1H-NMR spectrum of compound 9ai

	Parameter	Value
1	Solvent	$CDCl_3$
2	Spectrometer Frequency	100 MHz
3	Nucleus	^{13}C

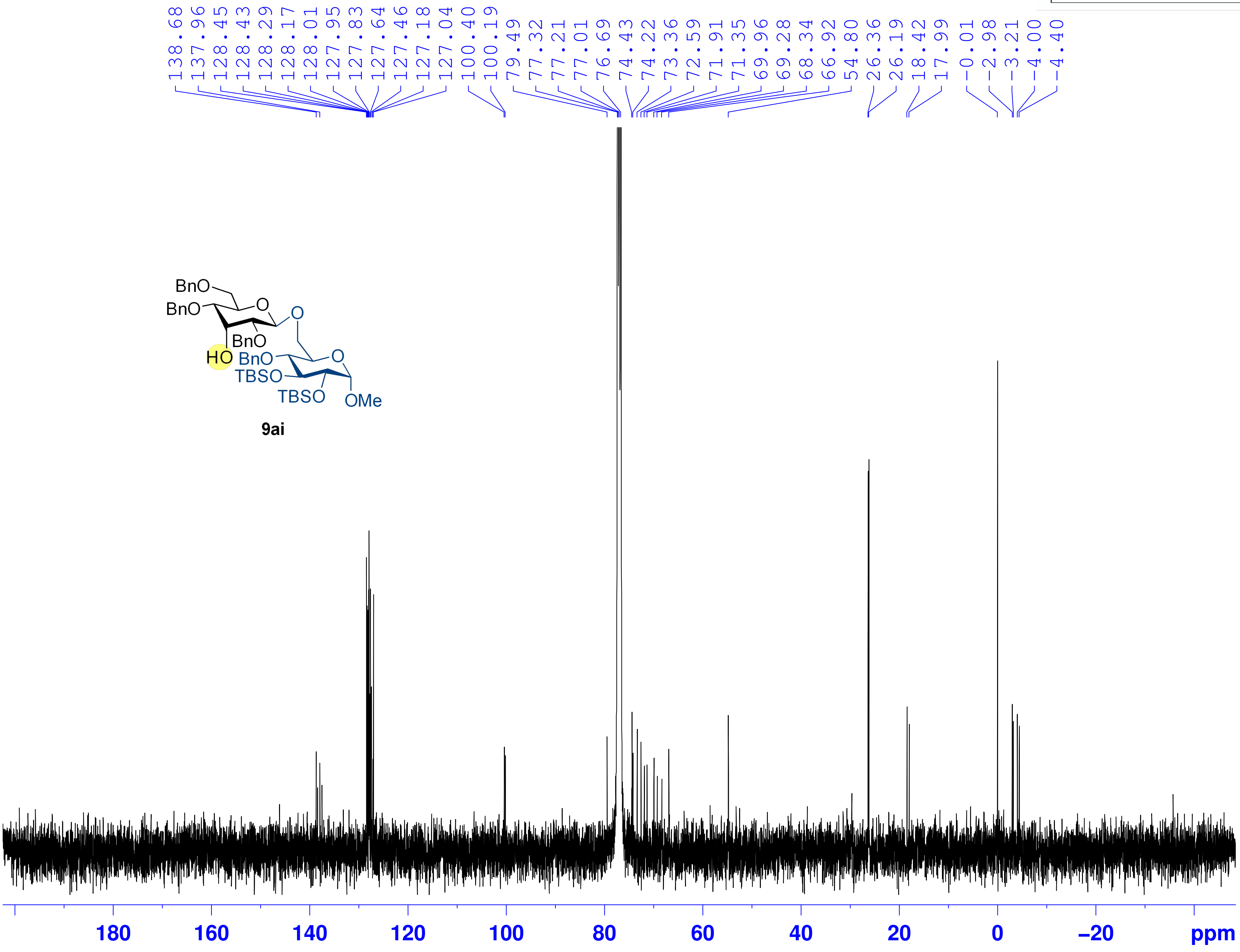

Supplementary Figure 74. 13C-NMR spectrum of compound 9ai

	Parameter	Value
1	Solvent	$CDCl_3$
2	Spectrometer Frequency	400 MHz
3	Nucleus	^{1}H

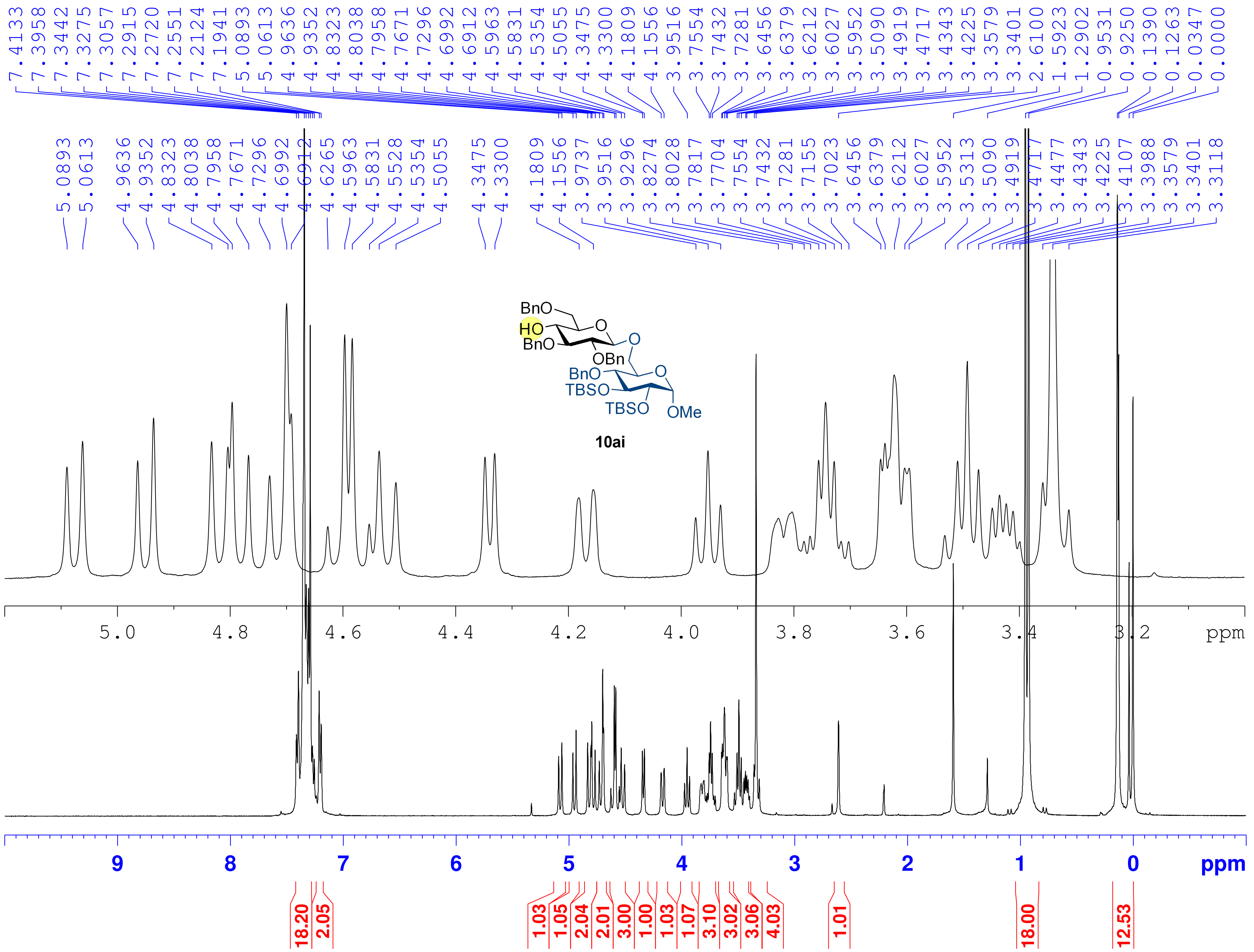

Supplementary Figure 75. 1H-NMR spectrum of compound 10ai

	Parameter	Value
1	Solvent	$CDCl_3$
2	Spectrometer Frequency	100 MHz
3	Nucleus	^{13}C

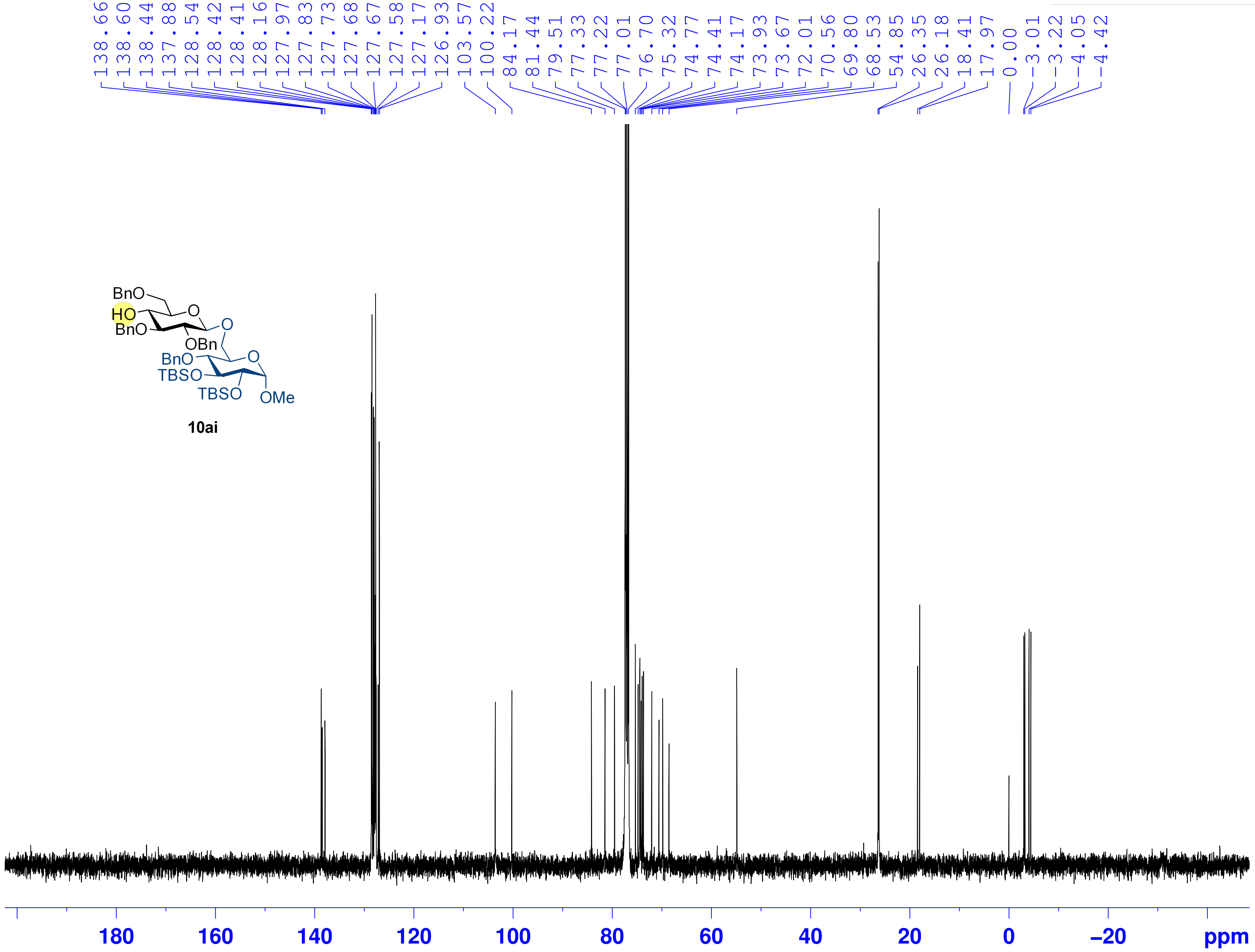

Supplementary Figure 76. 13C-NMR spectrum of compound 10ai

	Parameter	Value
1	Solvent	$CDCl_3$
2	Spectrometer Frequency	500 MHz
3	Nucleus	^{1}H

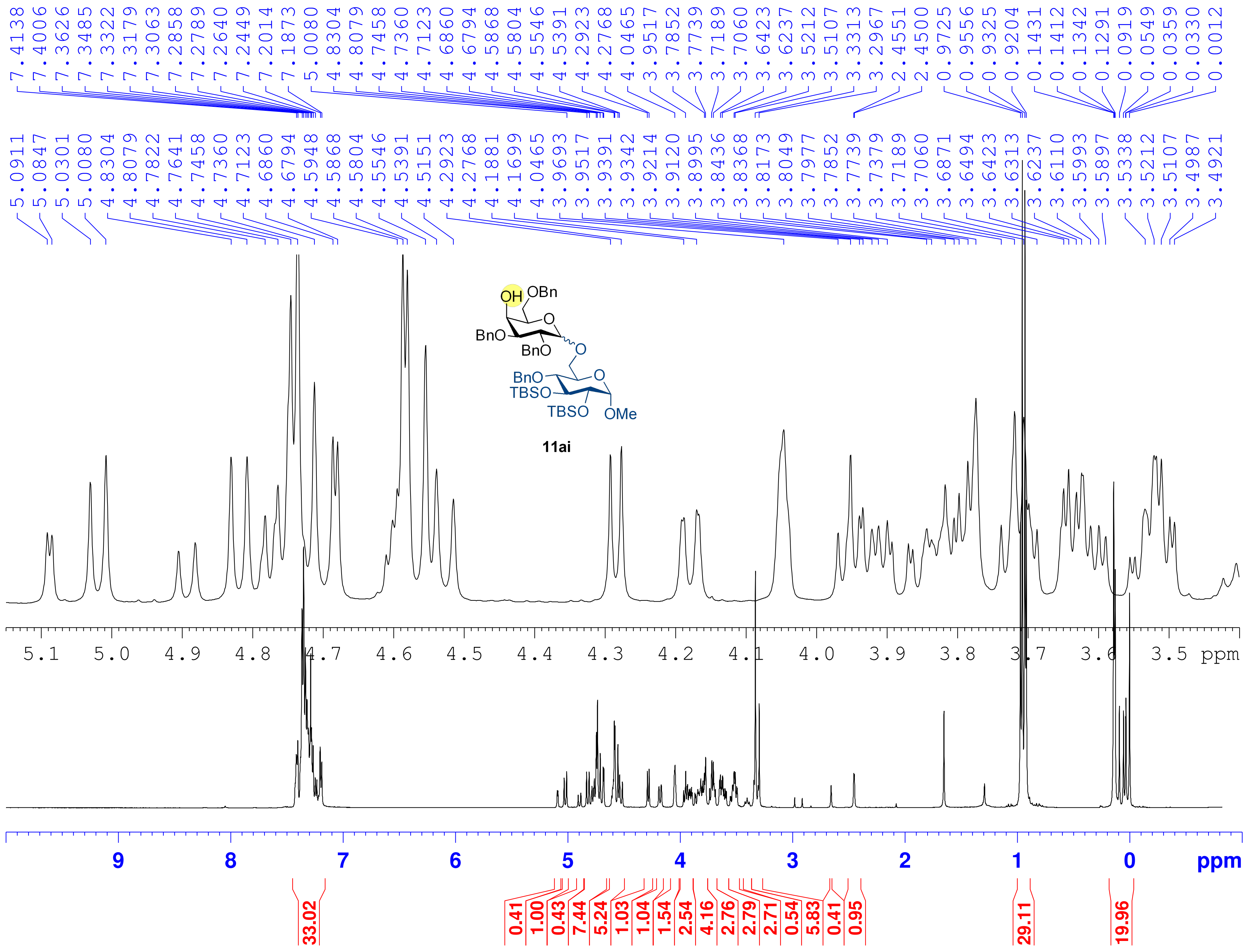

Supplementary Figure 77. 1H-NMR spectrum of compound 11ai

	Parameter	Value
1	Solvent	$CDCl_3$
2	Spectrometer Frequency	125 MHz
3	Nucleus	^{13}C

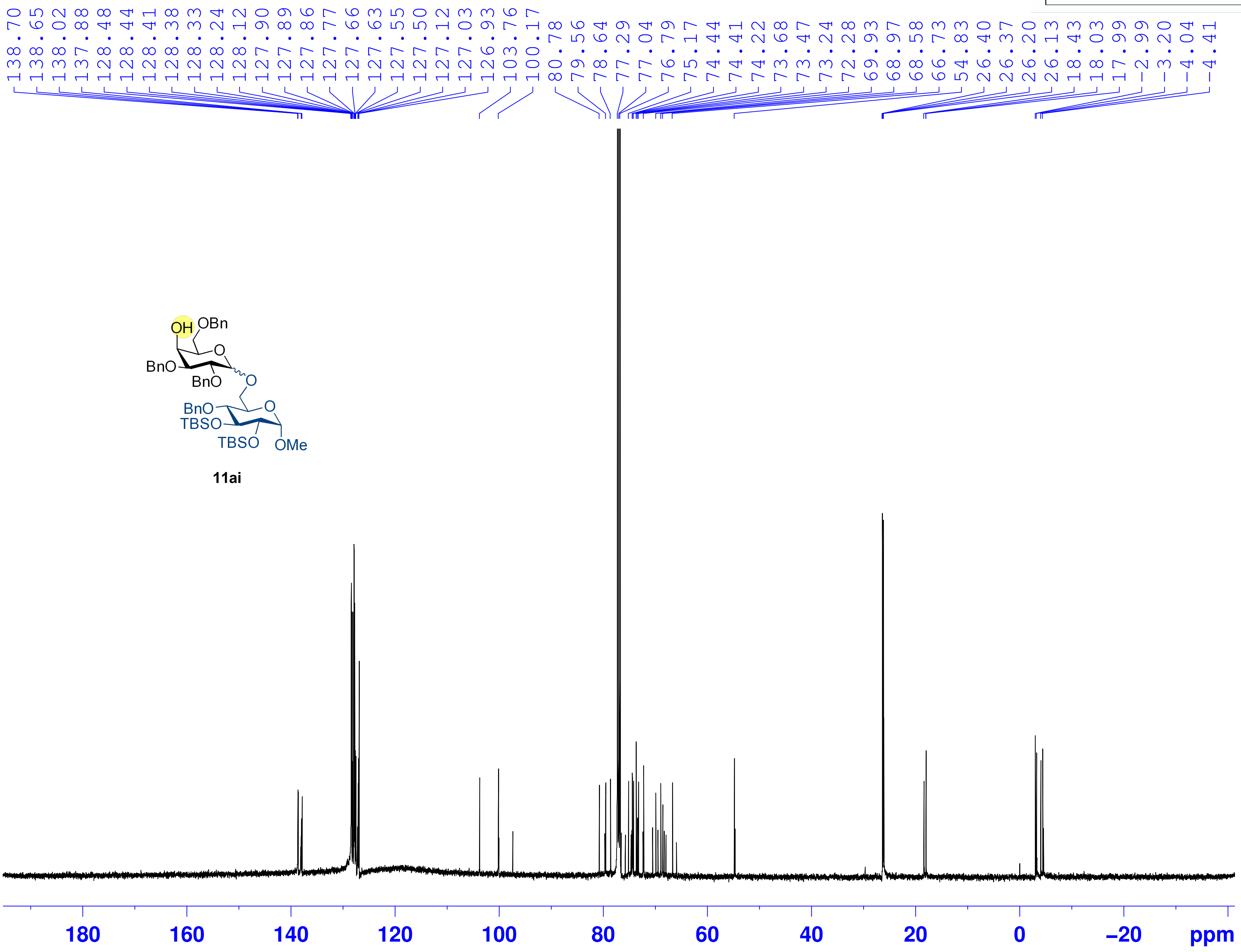

Supplementary Figure 78. 13C-NMR spectrum of compound 11ai

	Parameter	Value
1	Solvent	$CDCl_3$
2	Spectrometer Frequency	500 MHz
3	Nucleus	^1H

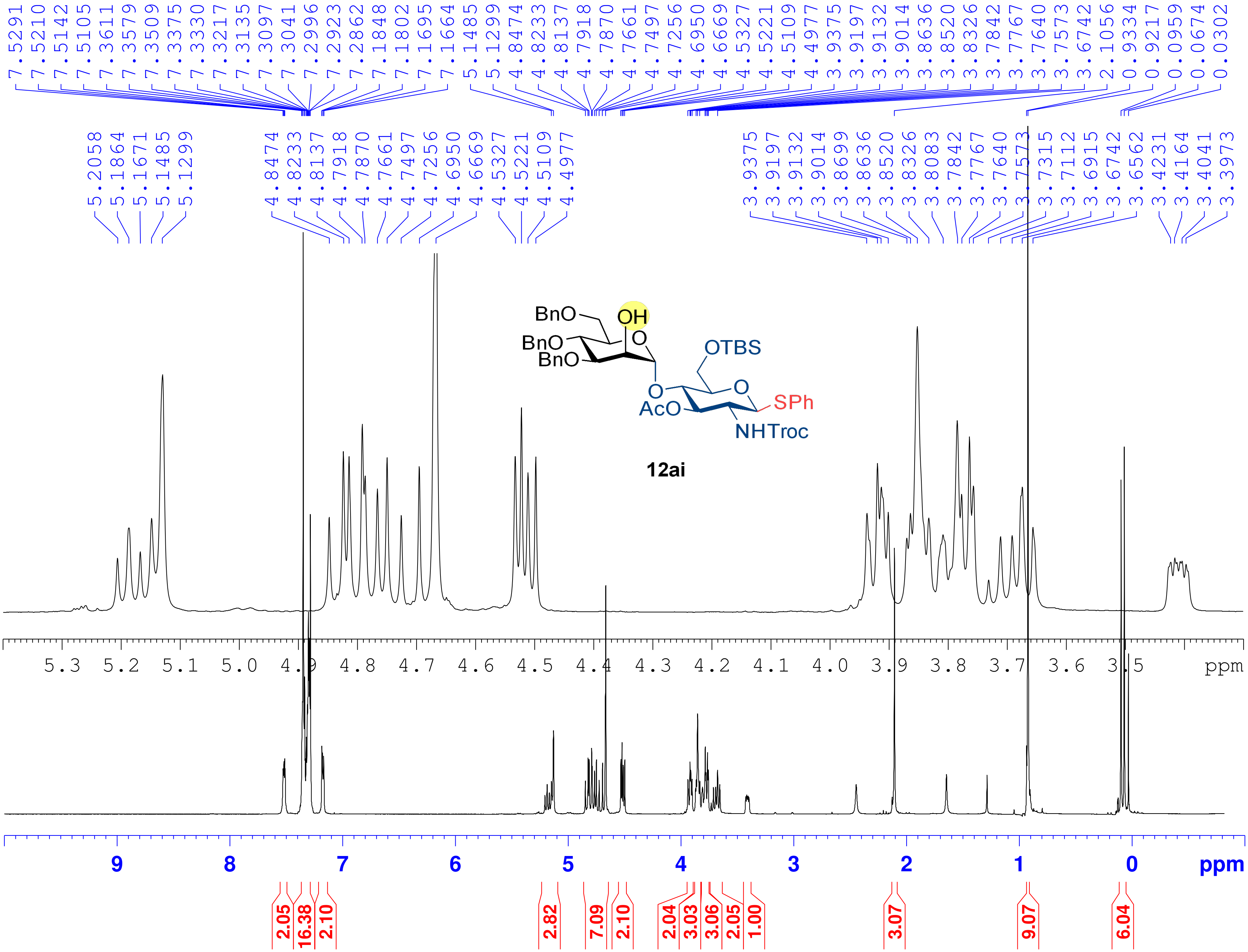

Supplementary Figure 79. 1H-NMR spectrum of compound 12ai

	Parameter	Value
1	Solvent	$CDCl_3$
2	Spectrometer Frequency	125 MHz
3	Nucleus	^{13}C

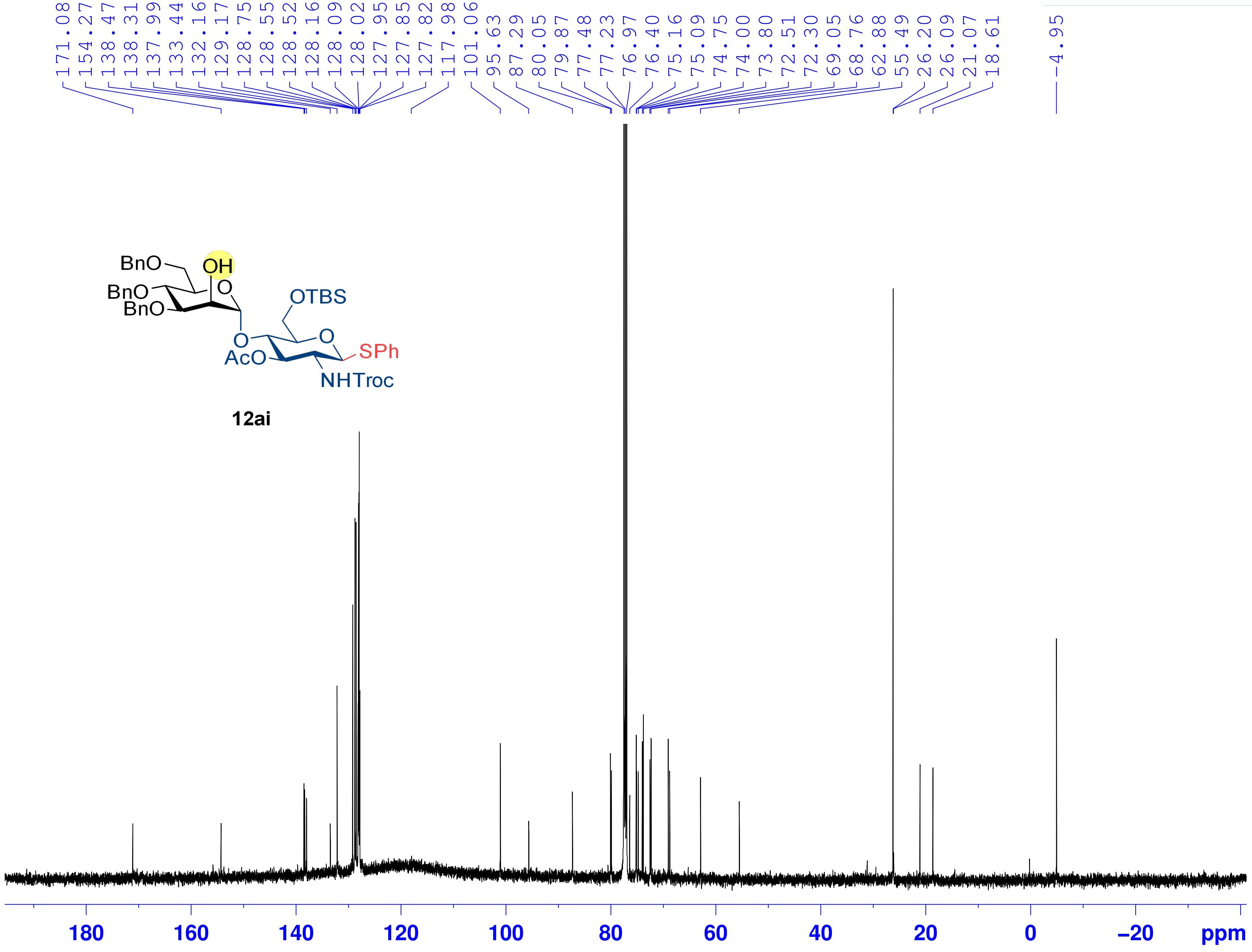

12ai

Supplementary Figure 80. 13C-NMR spectrum of compound 12ai

	Parameter	Value
1	Solvent	$CDCl_3$
2	Spectrometer Frequency	400 MHz
3	Nucleus	^1H

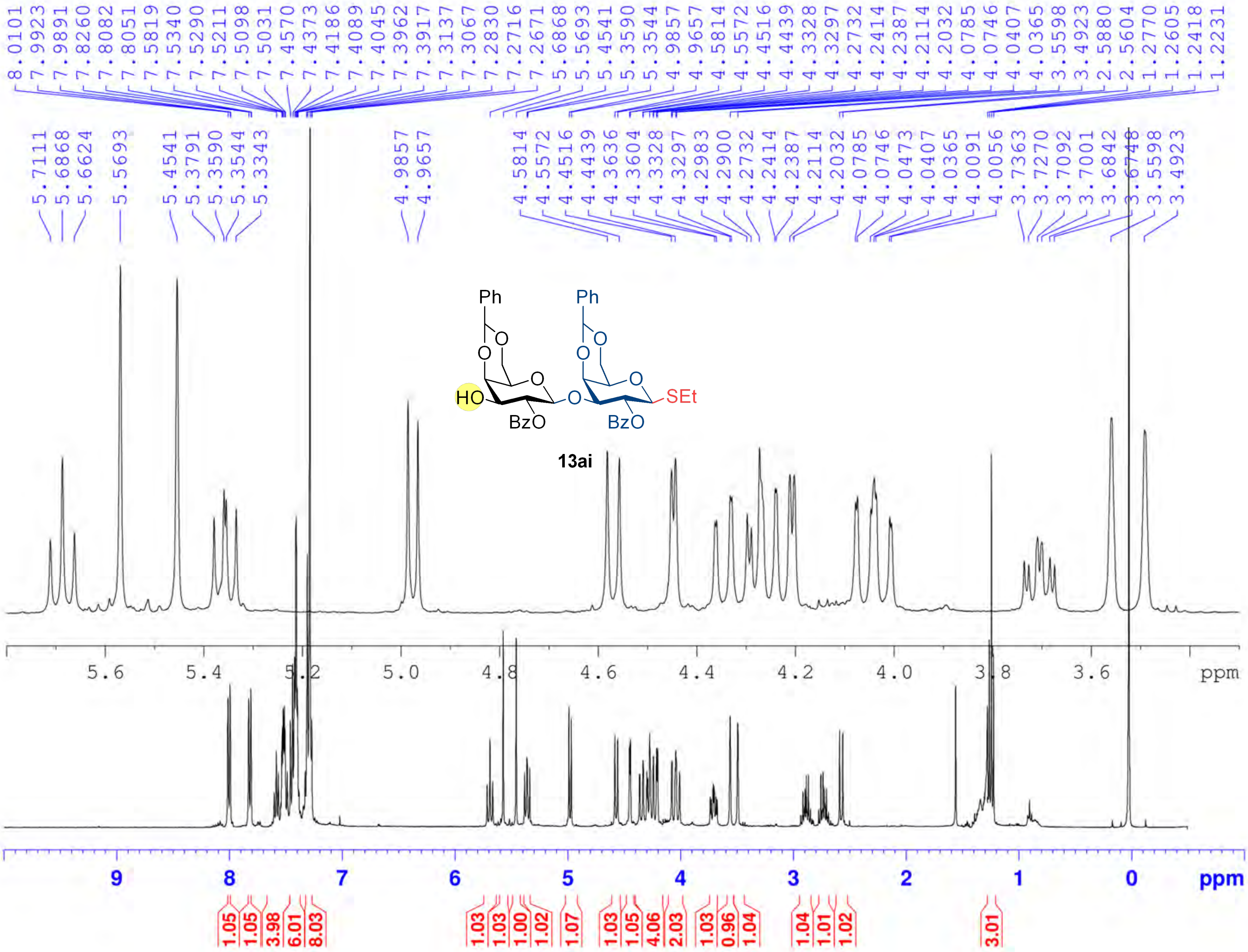

Supplementary Figure 81. 1H-NMR spectrum of compound 13ai

	Parameter	Value
1	Solvent	$CDCl_3$
2	Spectrometer Frequency	100 MHz
3	Nucleus	^{13}C

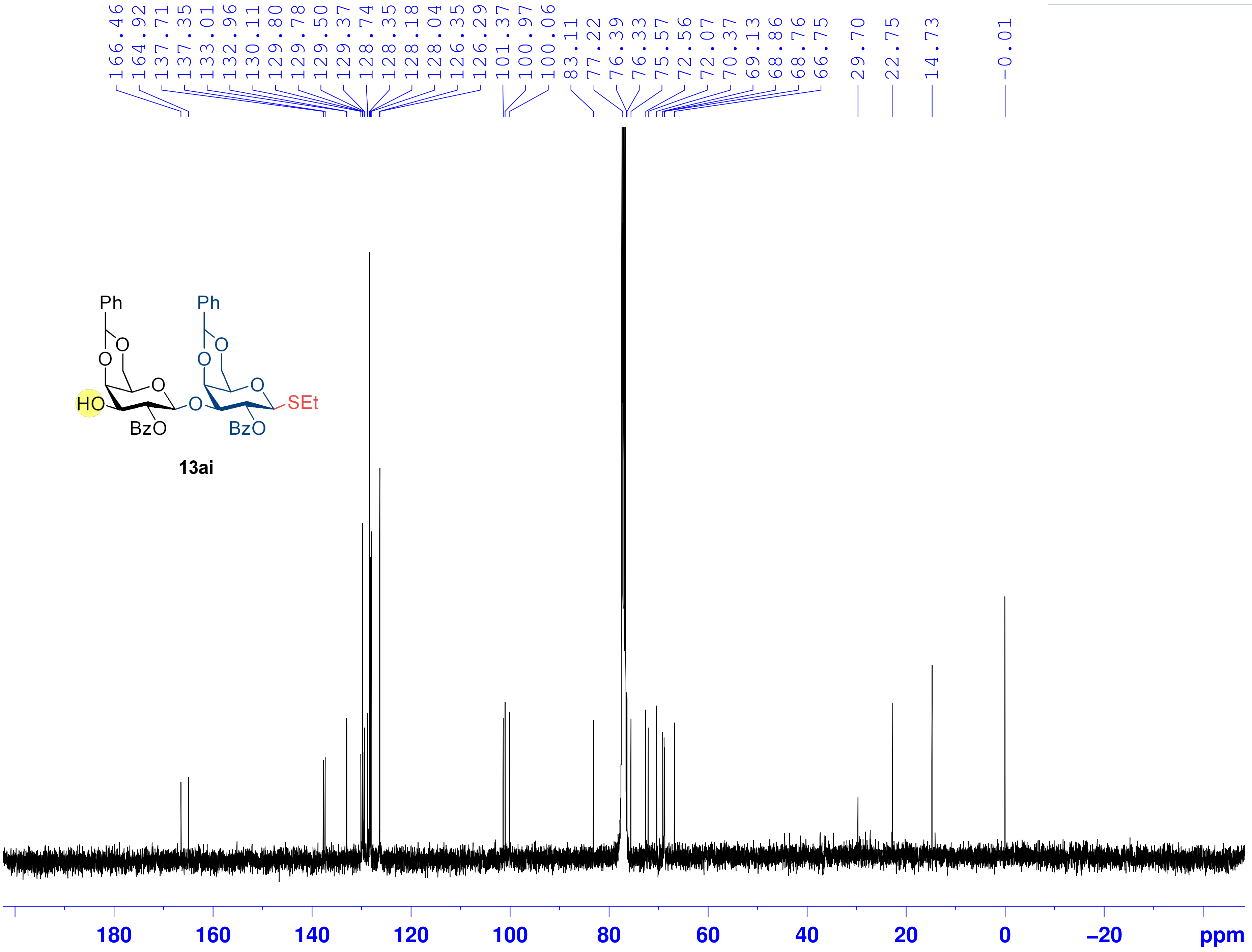

Supplementary Figure 82. 13C-NMR spectrum of compound 13ai

	Parameter	Value
1	Solvent	$CDCl_3$
2	Spectrometer Frequency	400 MHz
3	Nucleus	^{1}H

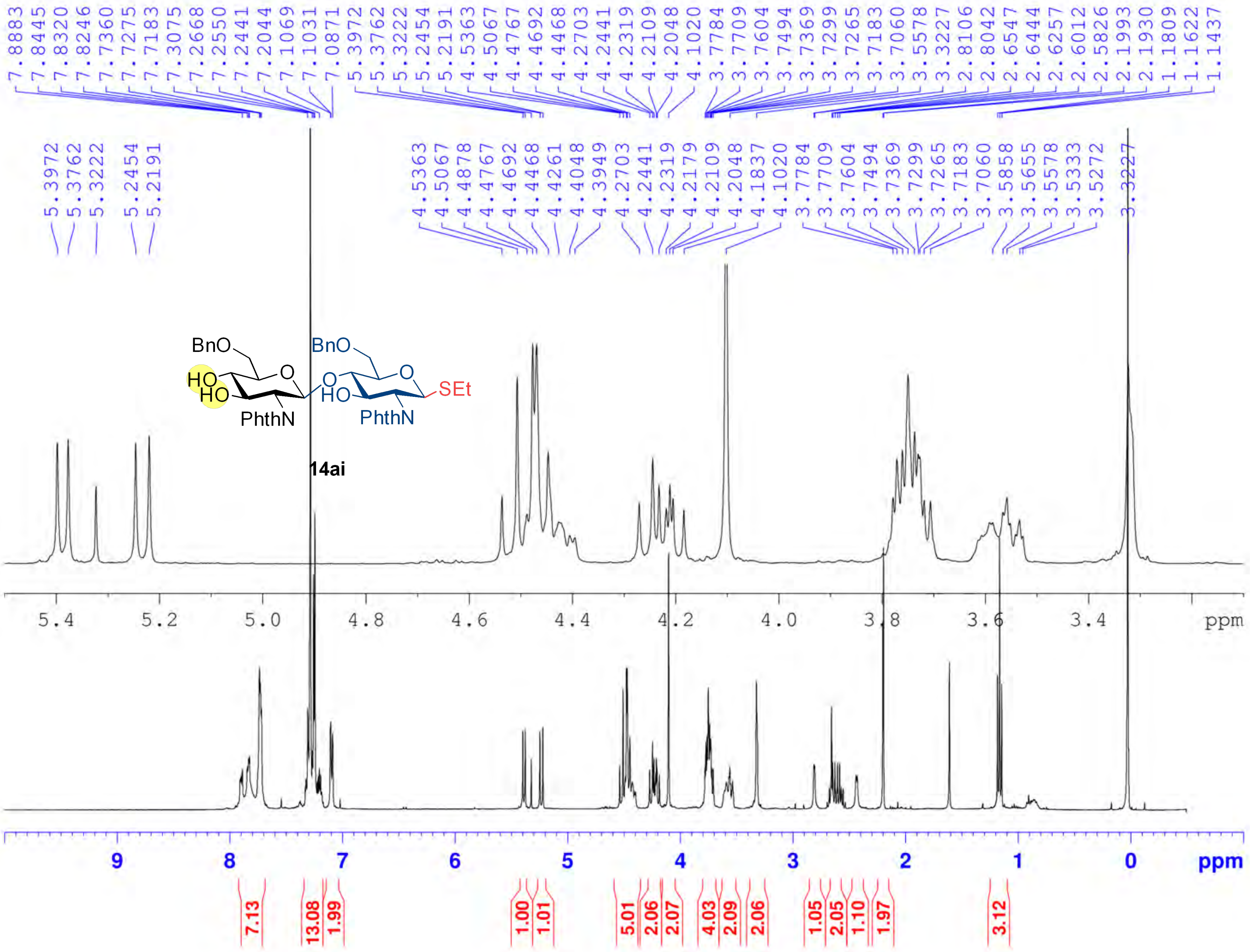

Supplementary Figure 83. 1H-NMR spectrum of compound 14ai

	Parameter	Value
1	Solvent	$CDCl_3$
2	Spectrometer Frequency	100 MHz
3	Nucleus	^{13}C

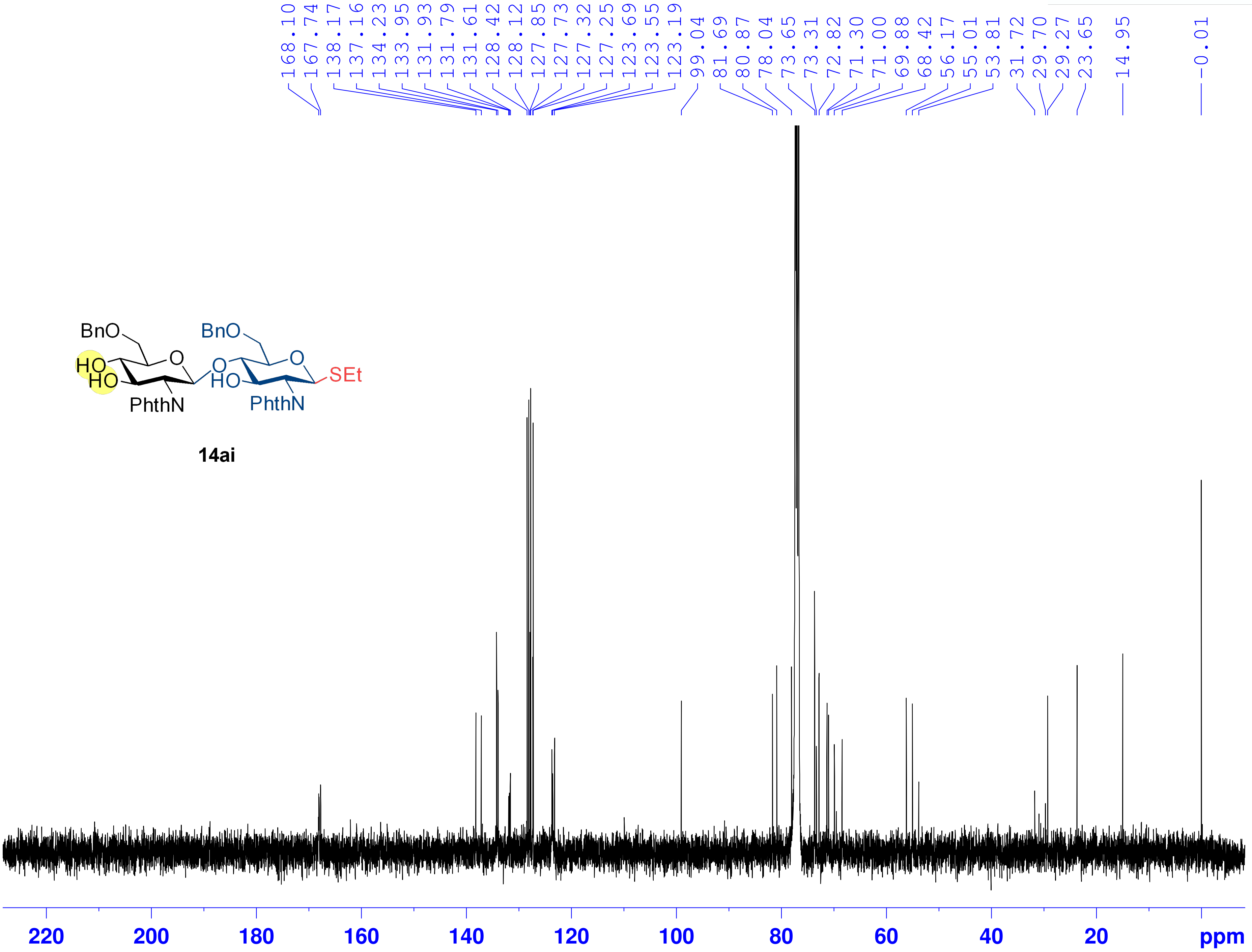

Supplementary Figure 84. 13C-NMR spectrum of compound 14ai

	Parameter	Value
1	Solvent	$CDCl_3$
2	Spectrometer Frequency	400 MHz
3	Nucleus	^{1}H

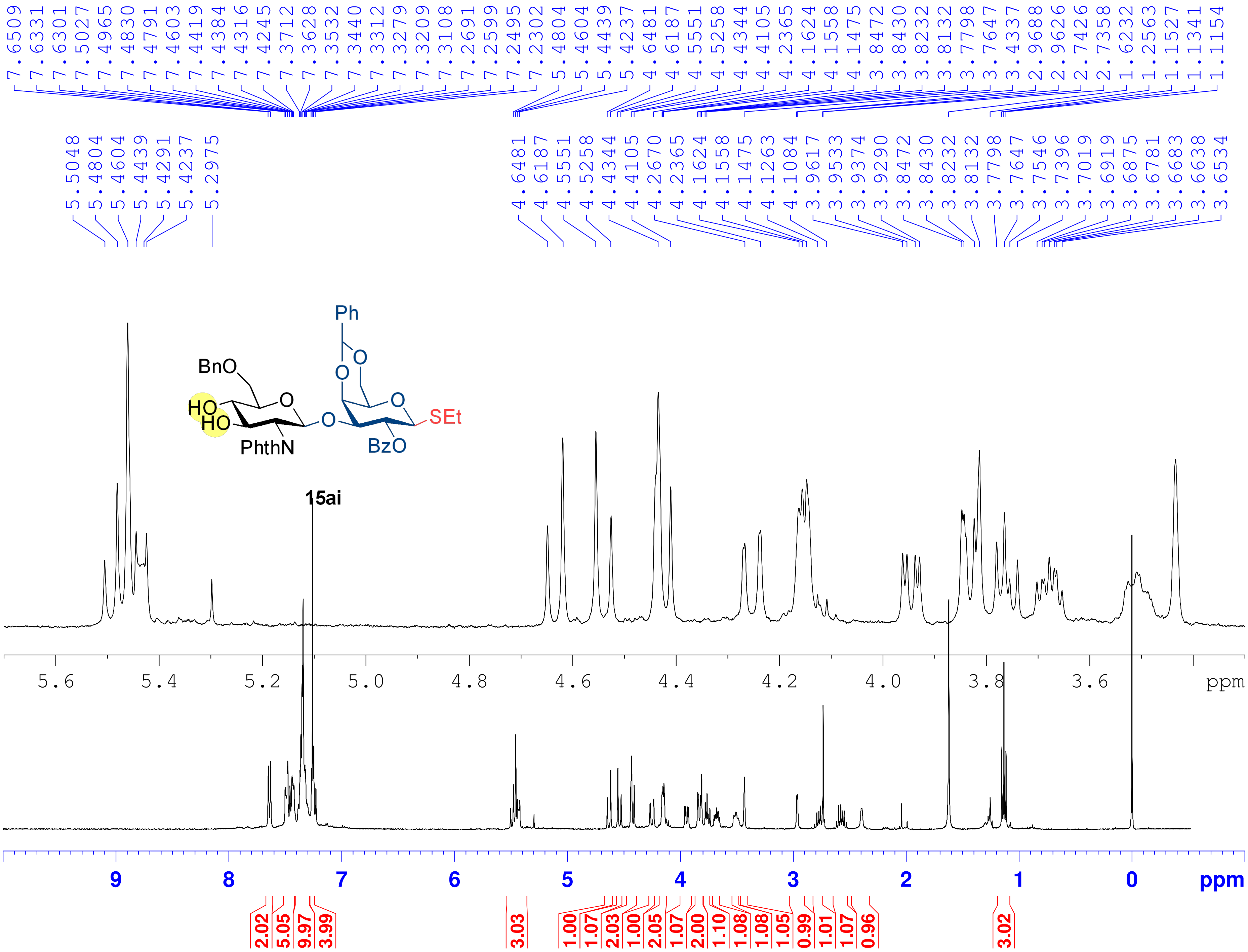

Supplementary Figure 85. 1H-NMR spectrum of compound 15ai

	Parameter	Value
1	Solvent	$CDCl_3$
2	Spectrometer Frequency	100 MHz
3	Nucleus	^{13}C

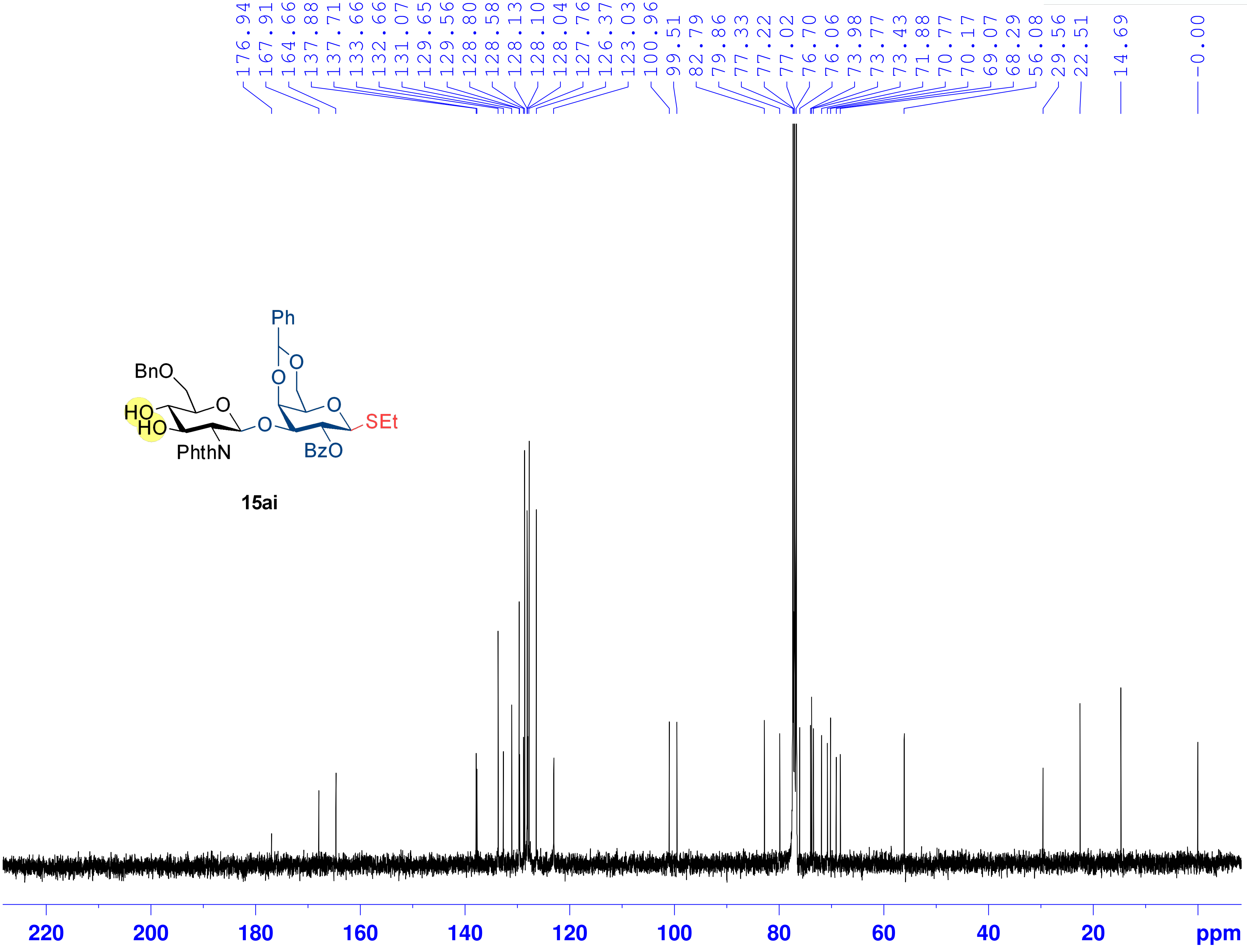

Supplementary Figure 86. 13C-NMR spectrum of compound 15ai

	Parameter	Value
1	Solvent	$CDCl_3$
2	Spectrometer Frequency	400 MHz
3	Nucleus	^1H

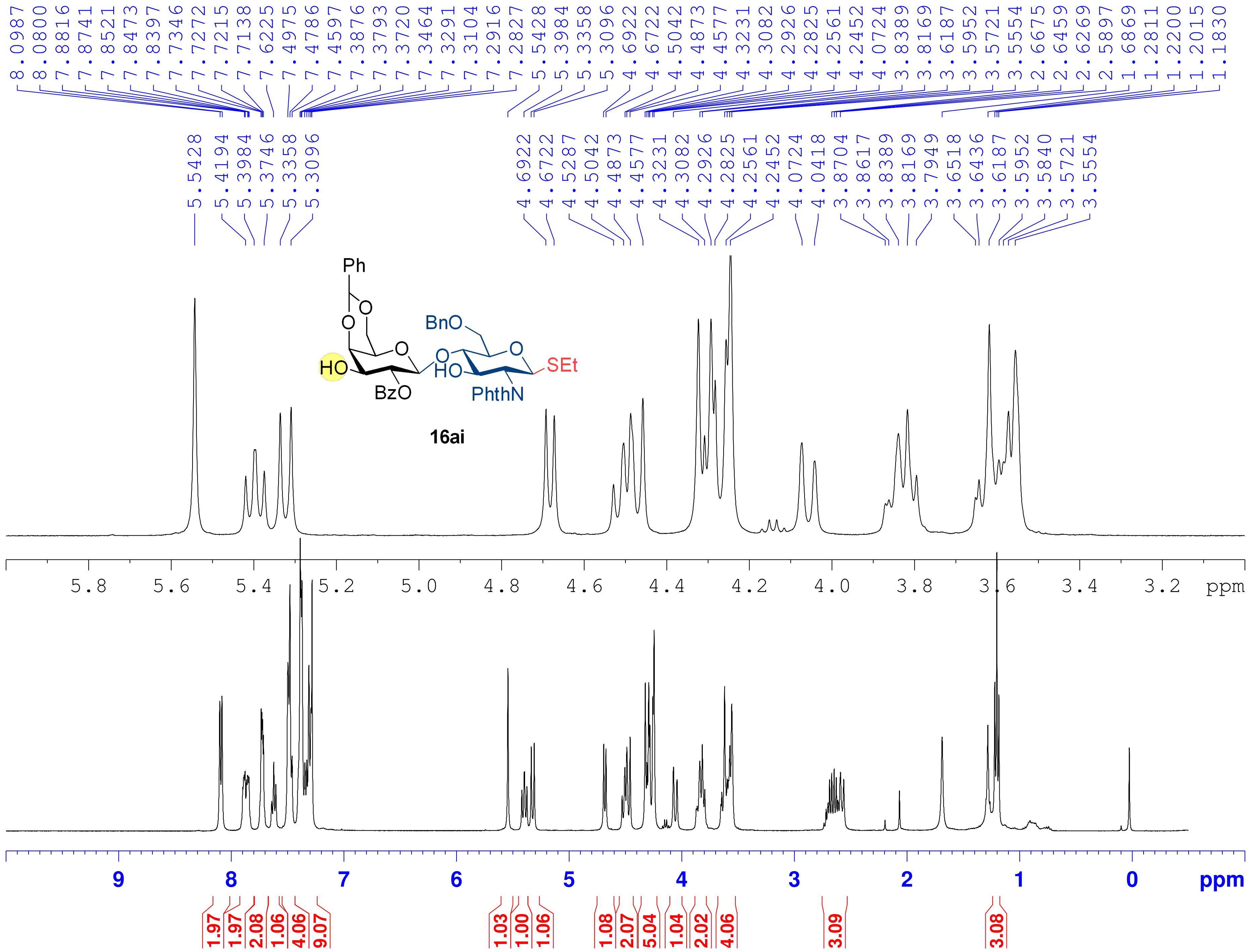

Supplementary Figure 87. 1H-NMR spectrum of compound 16ai

	Parameter	Value
1	Solvent	$CDCl_3$
2	Spectrometer Frequency	100 MHz
3	Nucleus	^{13}C

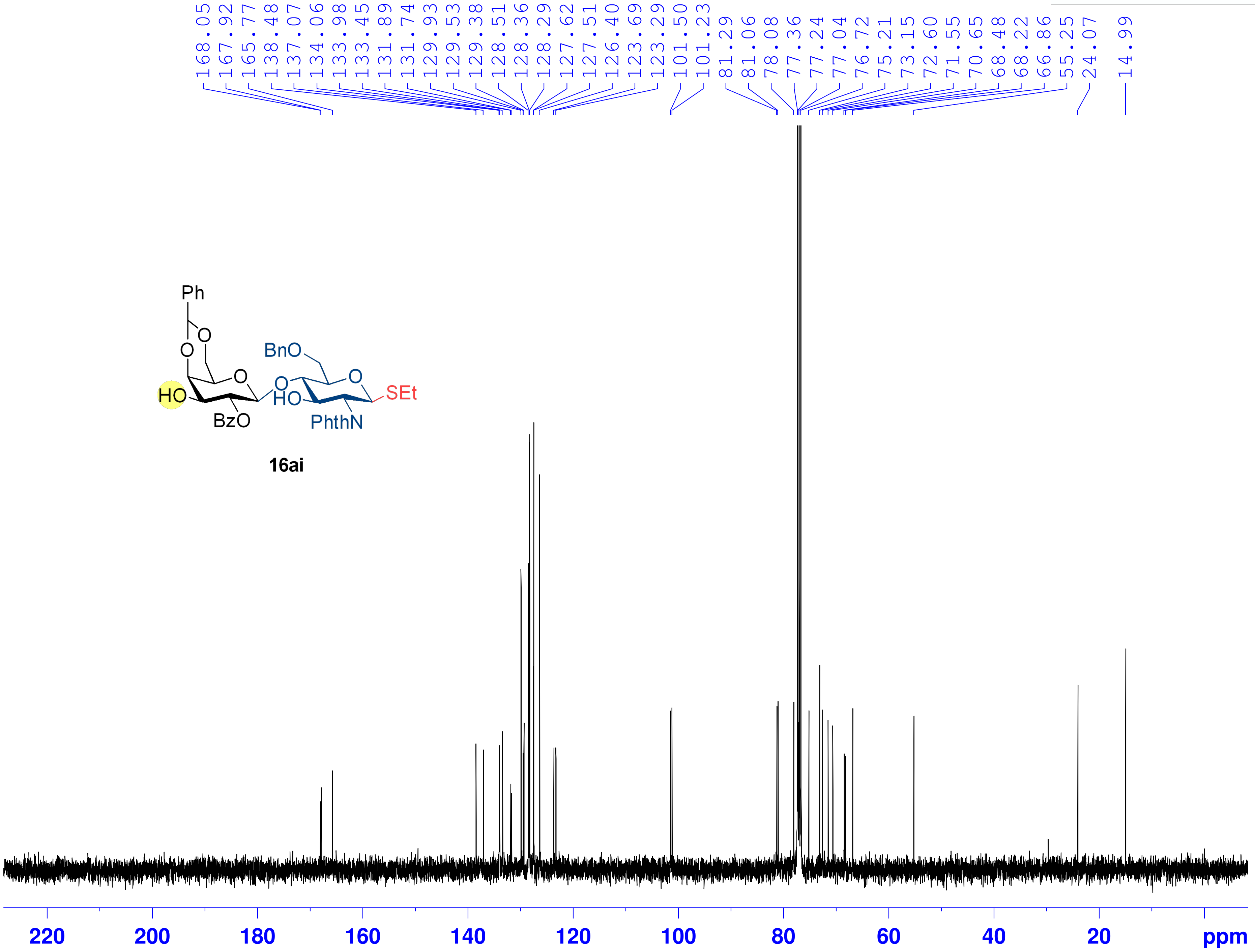

16ai

Supplementary Figure 88. 13C-NMR spectrum of compound 16ai

	Parameter	Value
1	Solvent	$CDCl_3$
2	Spectrometer Frequency	500 MHz
3	Nucleus	^{1}H

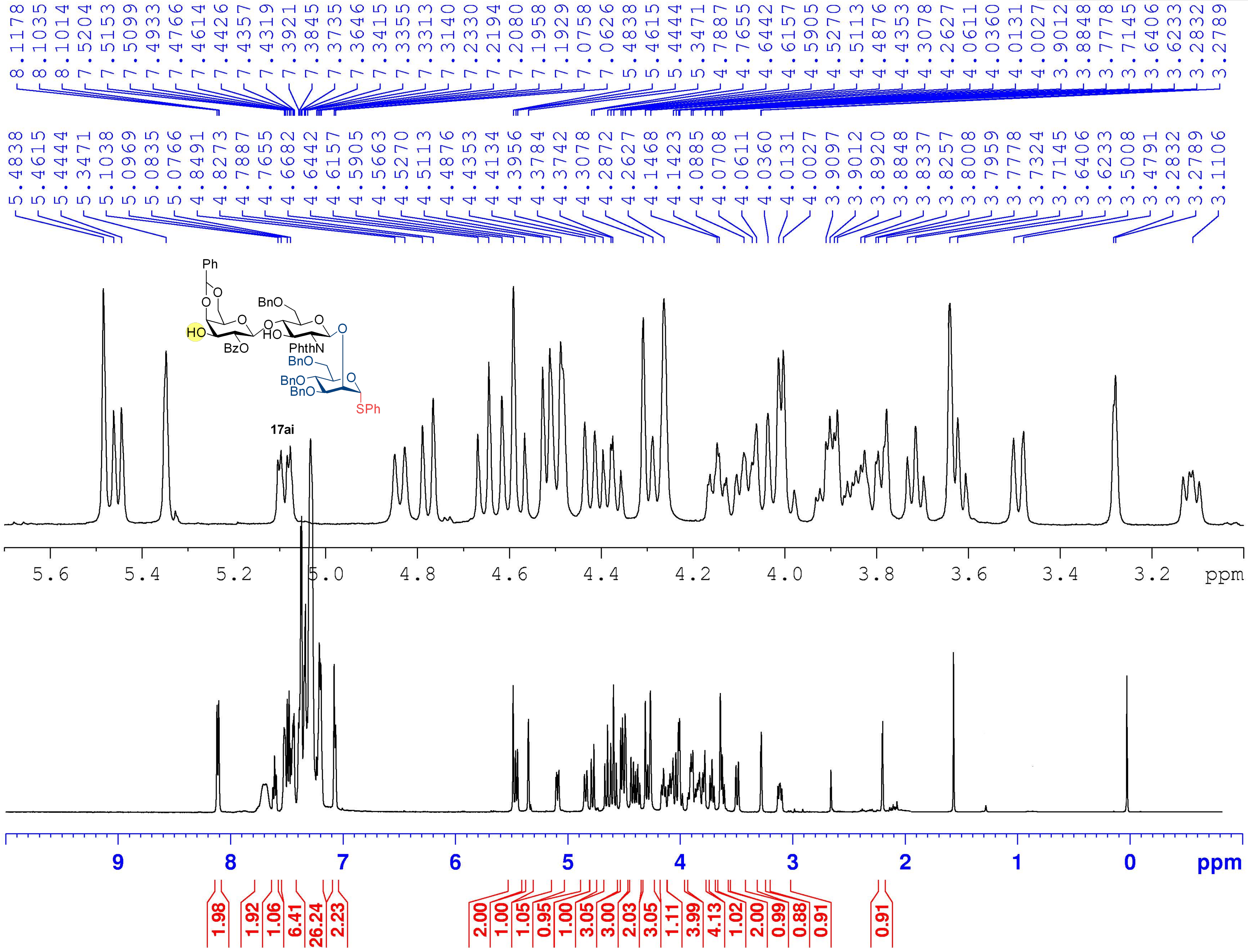

Supplementary Figure 89. 1H-NMR spectrum of compound 17ai

	Parameter	Value
1	Solvent	$CDCl_3$
2	Spectrometer Frequency	125 MHz
3	Nucleus	^{13}C

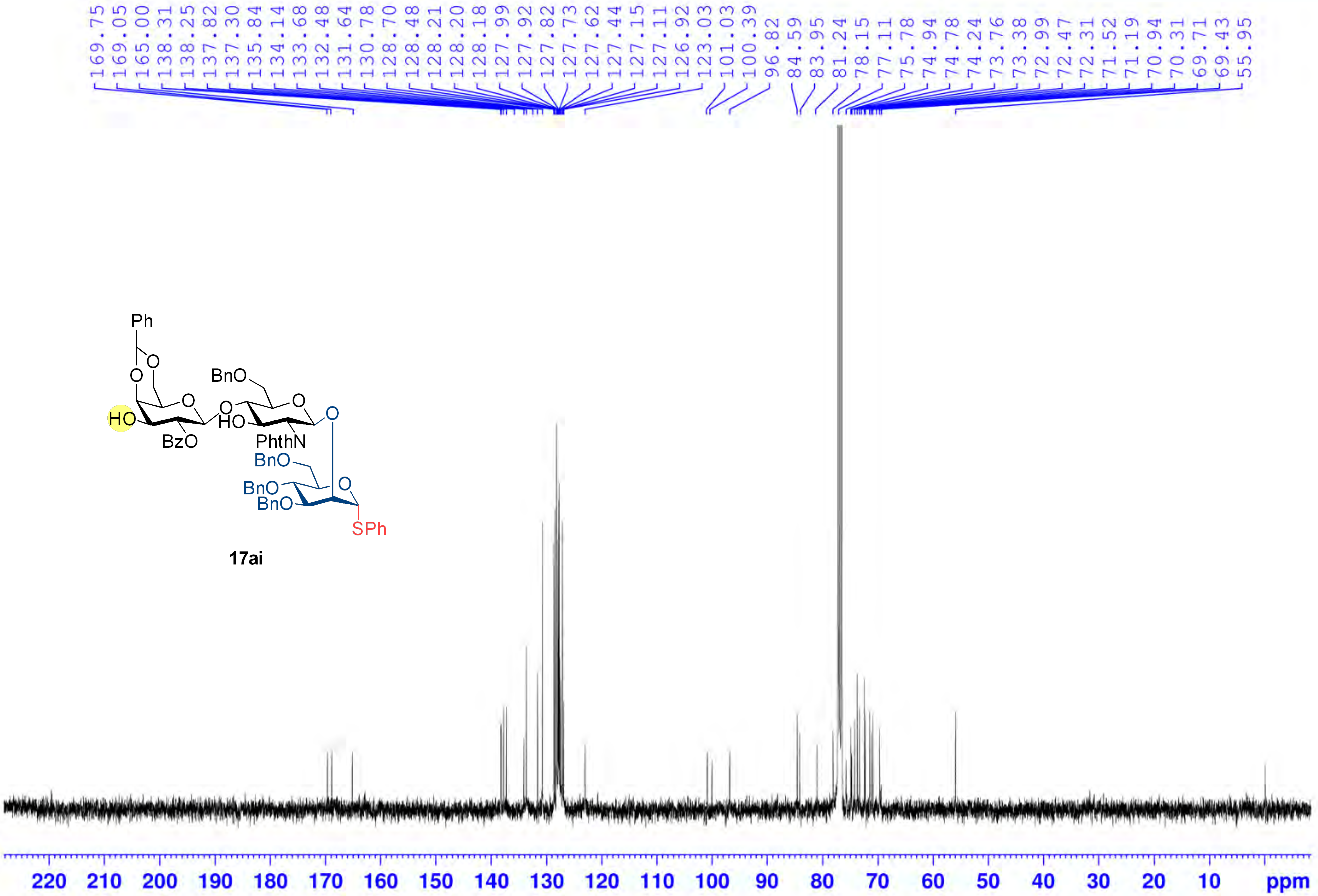

Supplementary Figure 90. 13C-NMR spectrum of compound 17ai

	Parameter	Value
1	Solvent	$CDCl_3$
2	Spectrometer Frequency	400 MHz
3	Nucleus	^{1}H

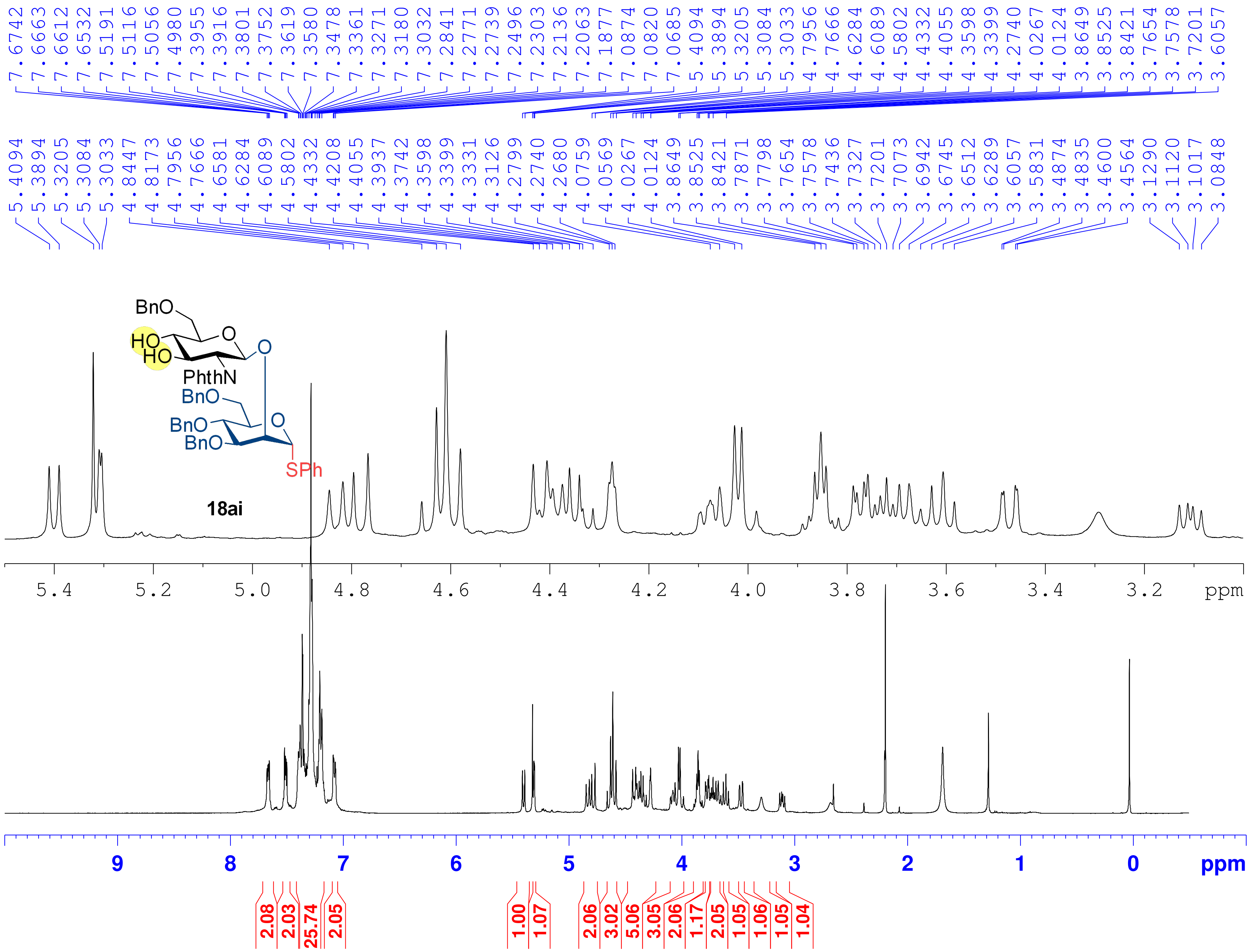

Supplementary Figure 91. 1H-NMR spectrum of compound 18ai

	Parameter	Value
1	Solvent	$CDCl_3$
2	Spectrometer Frequency	100 MHz
3	Nucleus	^{13}C

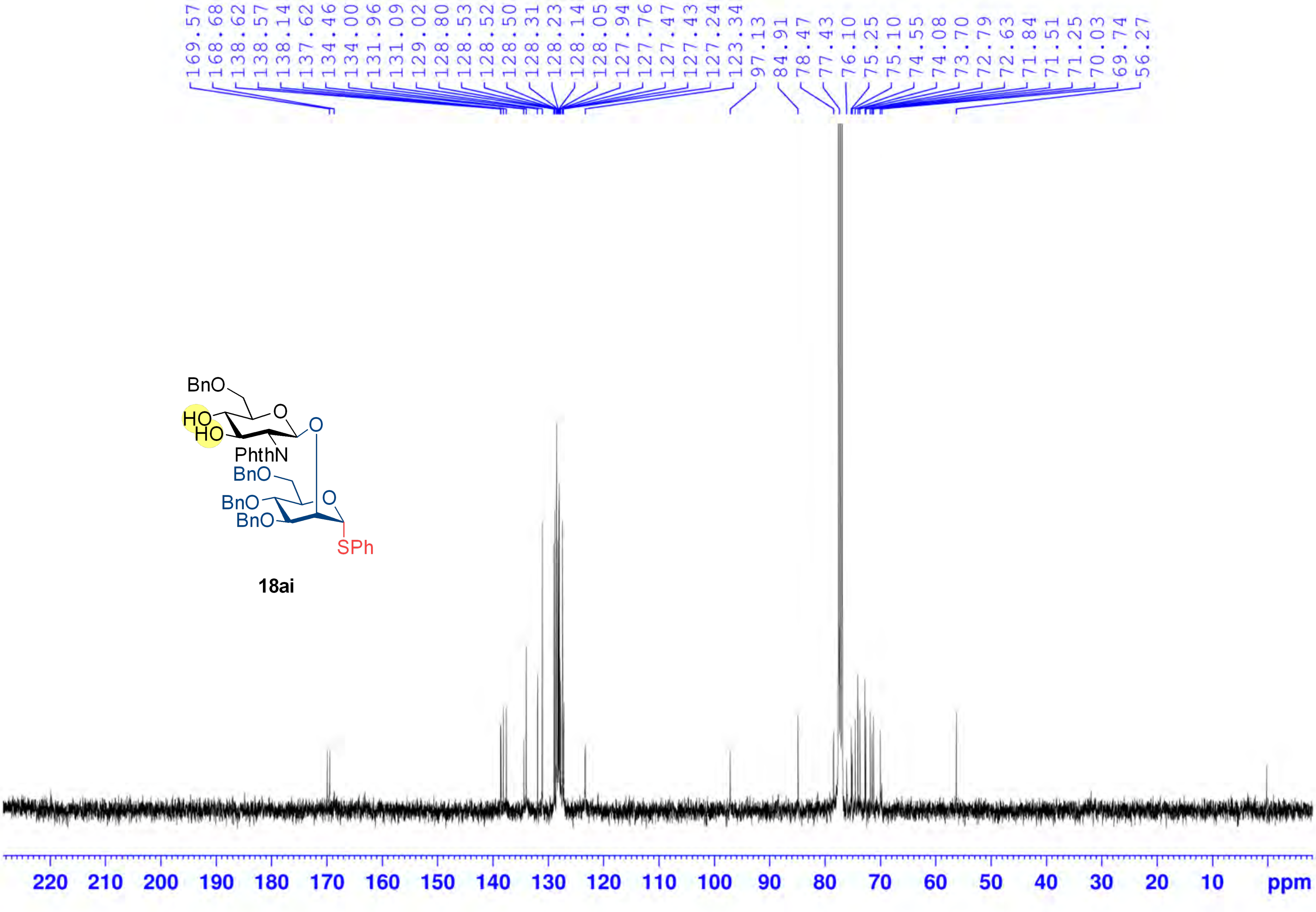

Supplementary Figure 92. 13C-NMR spectrum of compound 18ai

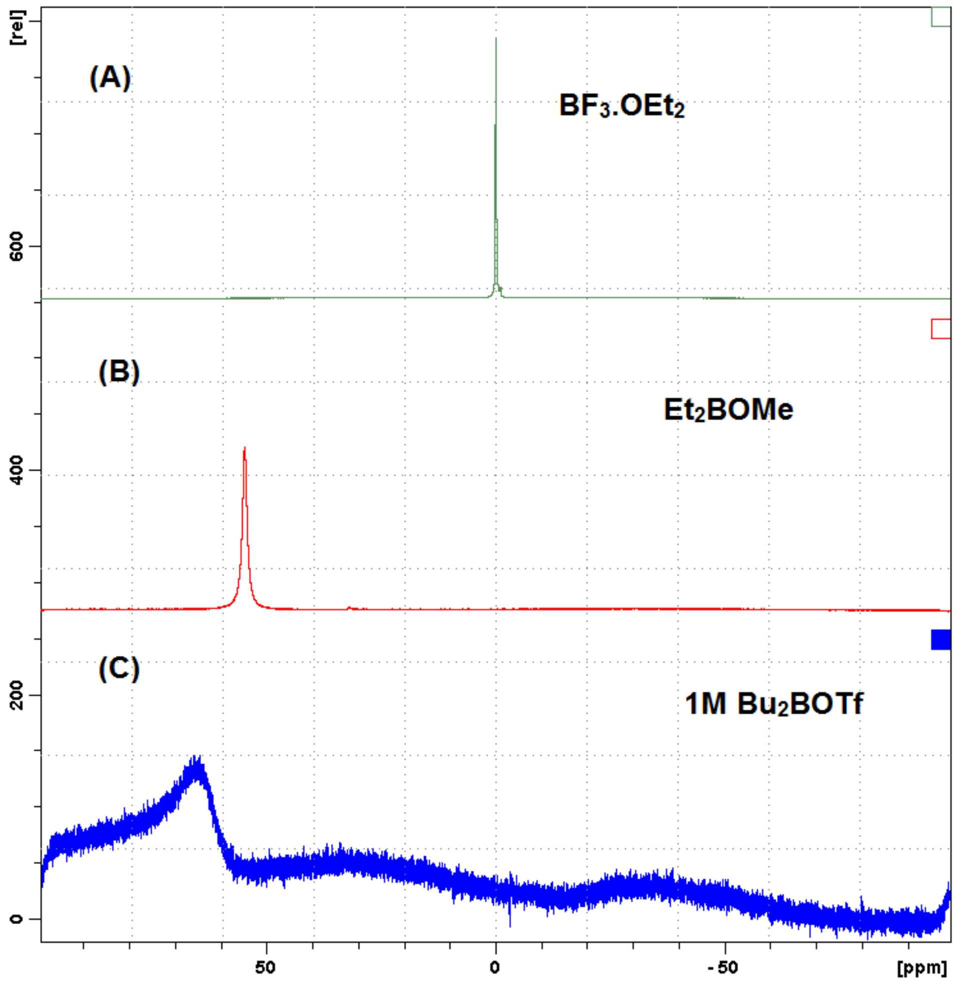

Supplementary Figure 93. Standard references for ^{11}B-NMR experiments: (A) $BF_3.OEt_2$ 0 ppm; (B) Et_2BOMe 54.8 ppm; (C) 1M Bu_2BOTf 64.0 ppm.

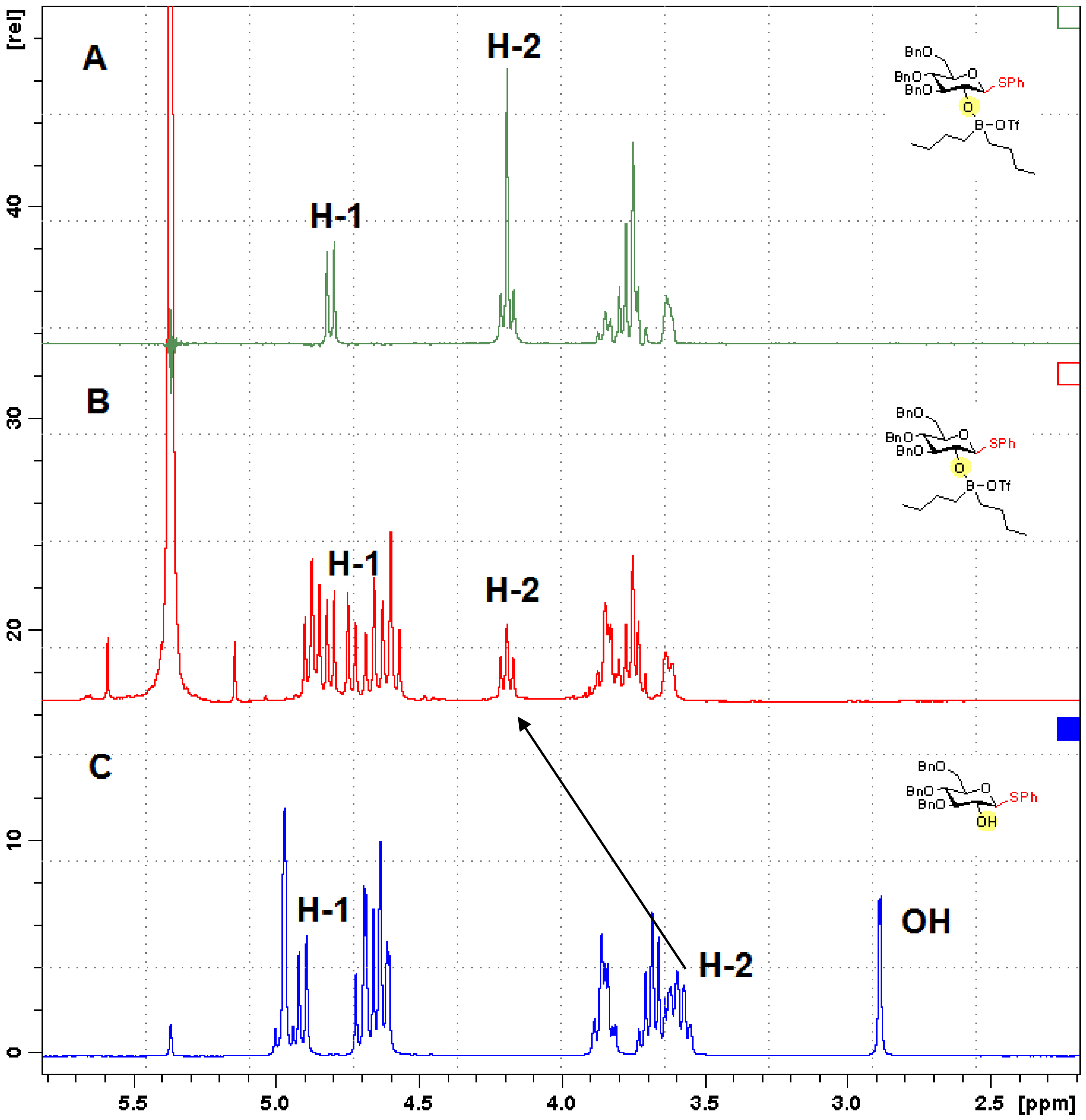

Supplementary Figure 94. ^{1}H-NMR of donor **1a** before and after addition of Bu_2BOTf. (A) 1D-TOCSY (d9=0.2ms, o1p=4.20ppm); (B) after addition of Bu_2BOTf; (C) before addition of Bu_2BOTf

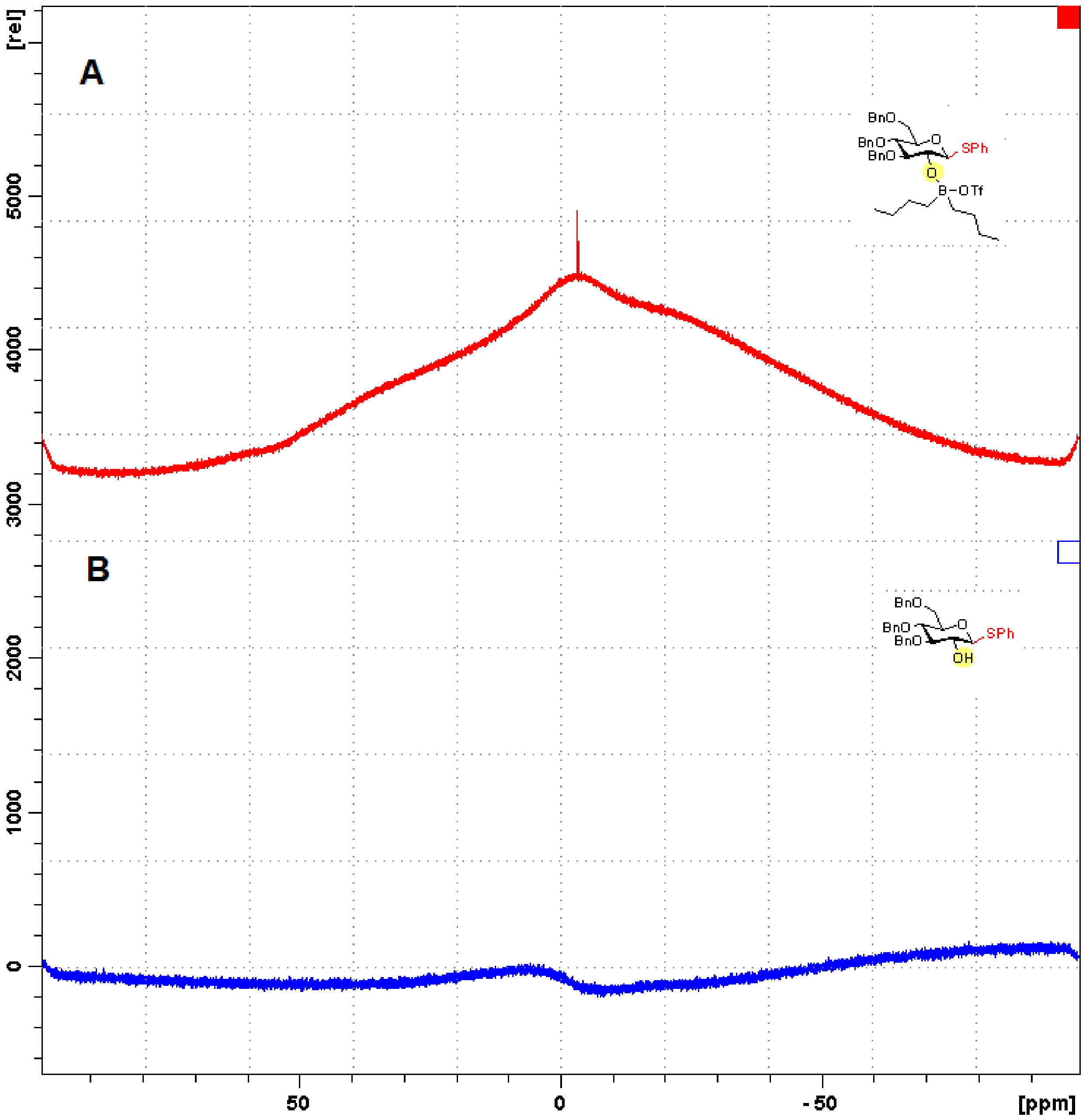

Supplementary Figure 95 ^{11}B-NMR of donor **1a** before and after addition of Bu_2BOTf. (A) after addition of Bu_2BOTf; (B) before addition of Bu_2BOTf.

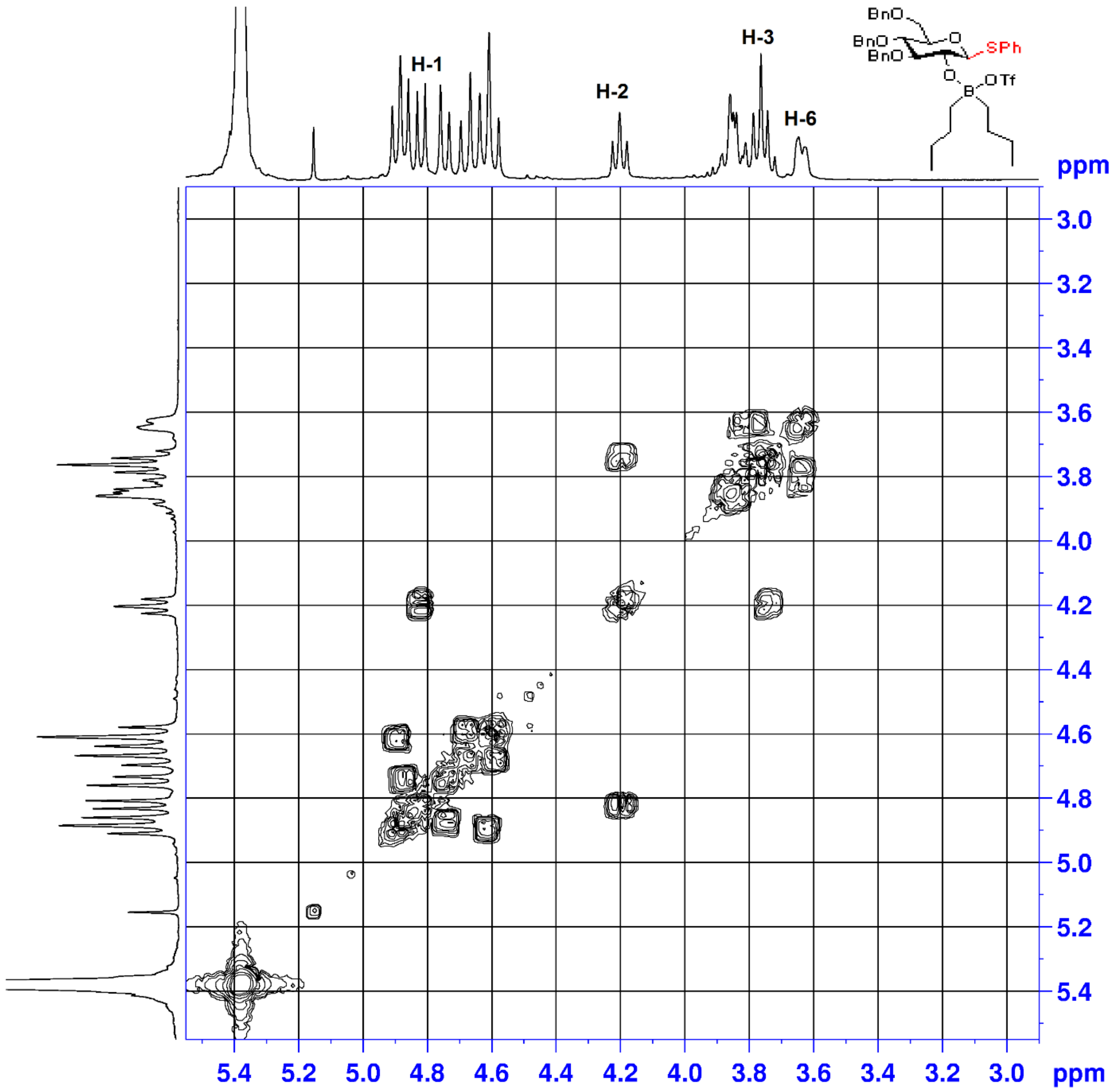

Supplementary Figure 96. 2D-COSY of donor **1a** after addition of Bu_2BOTf.

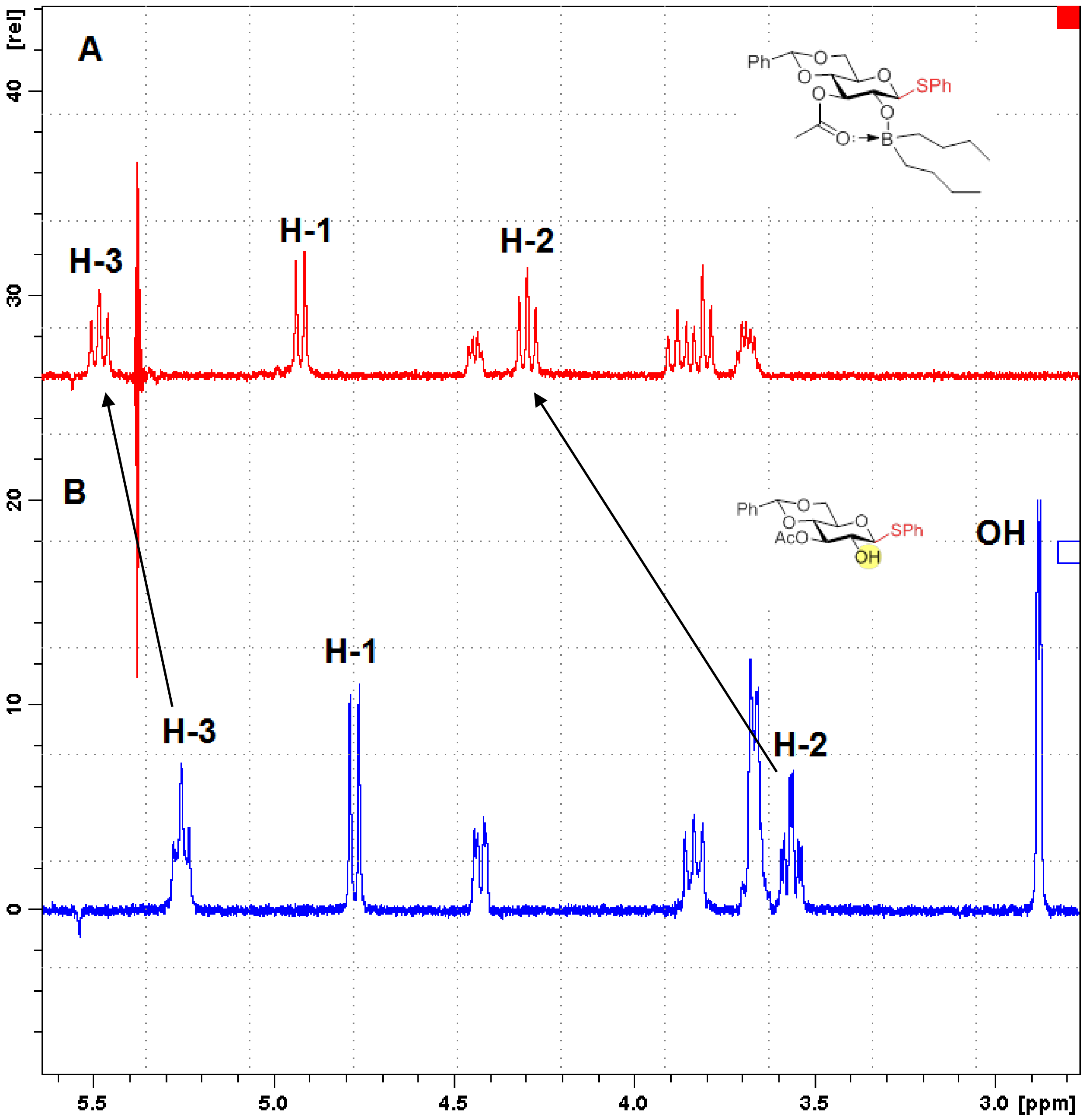

Supplementary Figure 97. (A) 1D-TOCSY of **29a** after addition of Bu_2BOTf; (B) before addition of Bu_2BOTf.

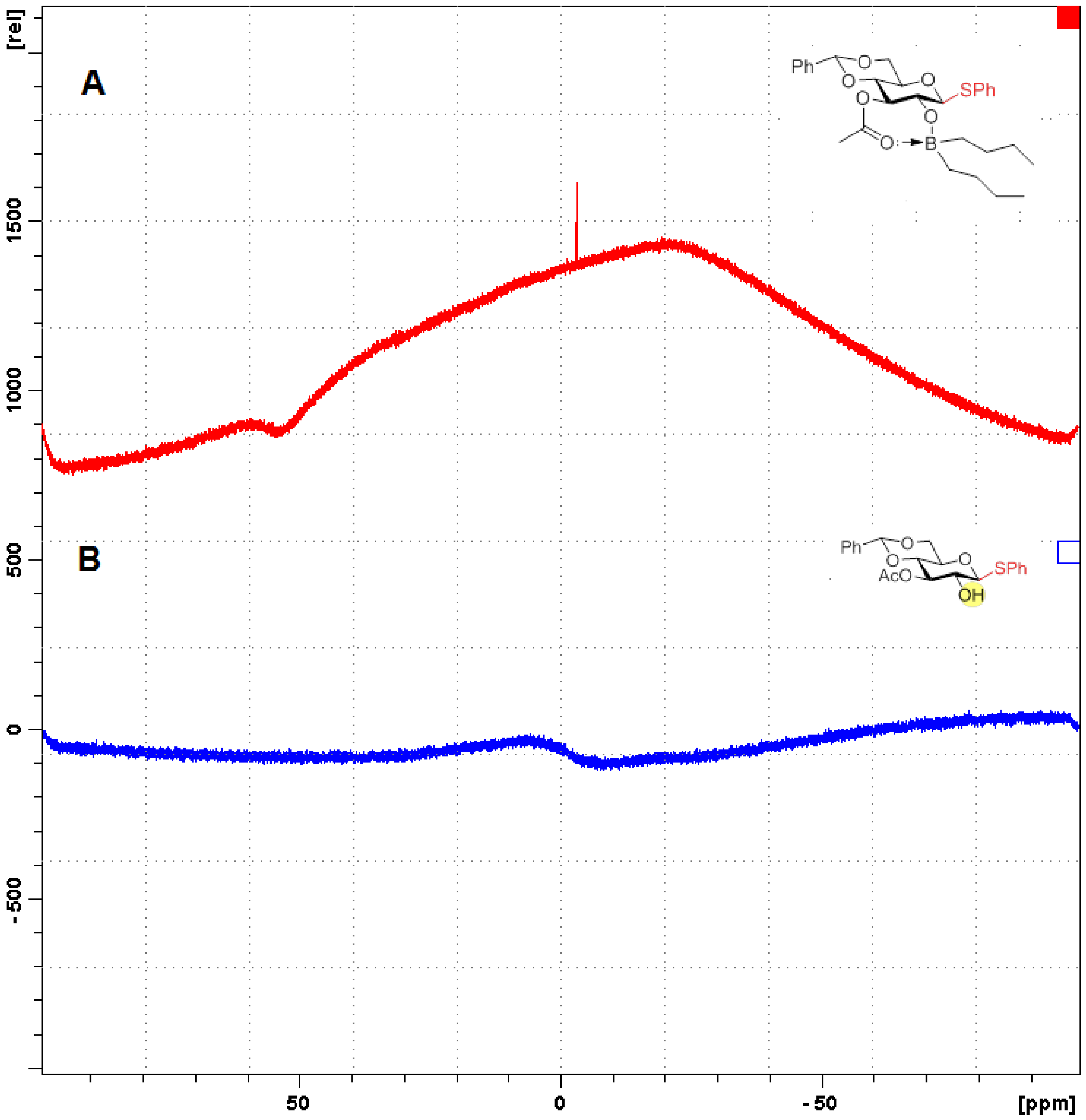

Supplementary Figure 98 ^{11}B-NMR of donor **29a** before and after addition of Bu_2BOTf. (A) after addition of Bu_2BOTf; (B) before addition of Bu_2BOTf.

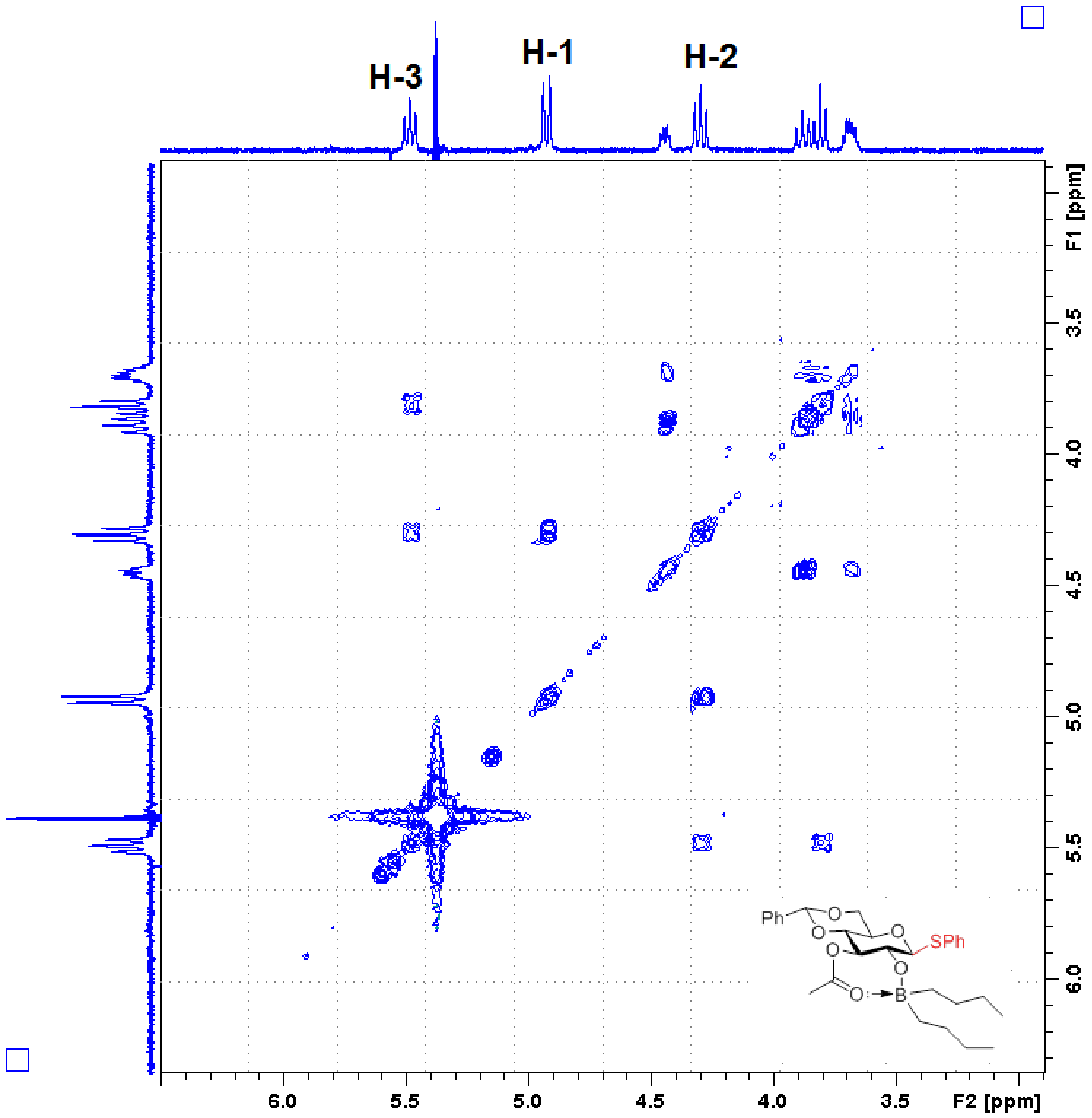

Supplementary Figure 99. 2D-COSY of donor **29a** after addition of Bu_2BOTf.

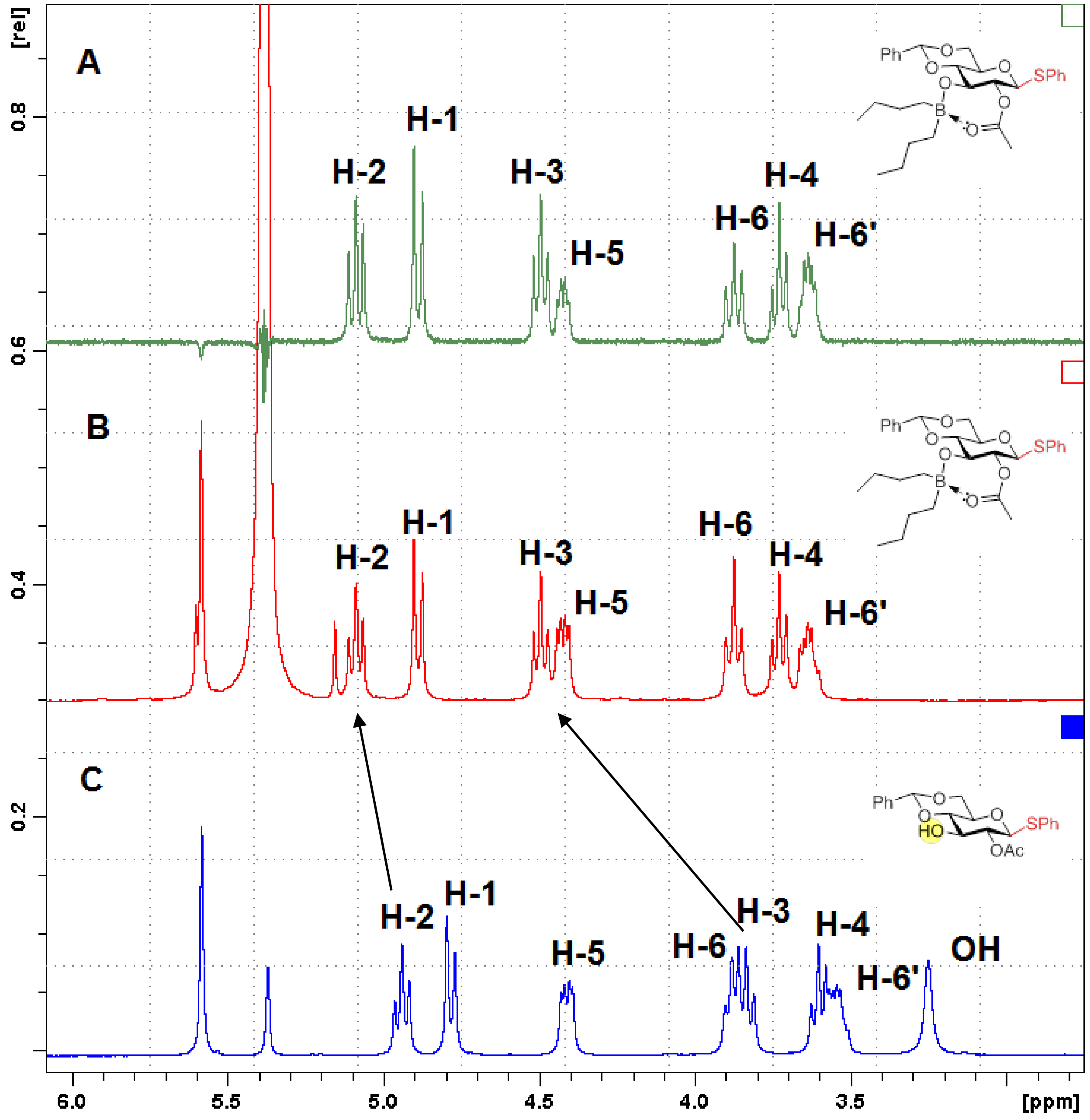

Supplementary Figure 100. (A) 1D-TOCSY of **30a** after addition of Bu_2BOTf; (B) ^1H-NMR of **2.2c** after addition of Bu_2BOTf; (C) ^1H-NMR of **30a** before addition of Bu_2BOTf.

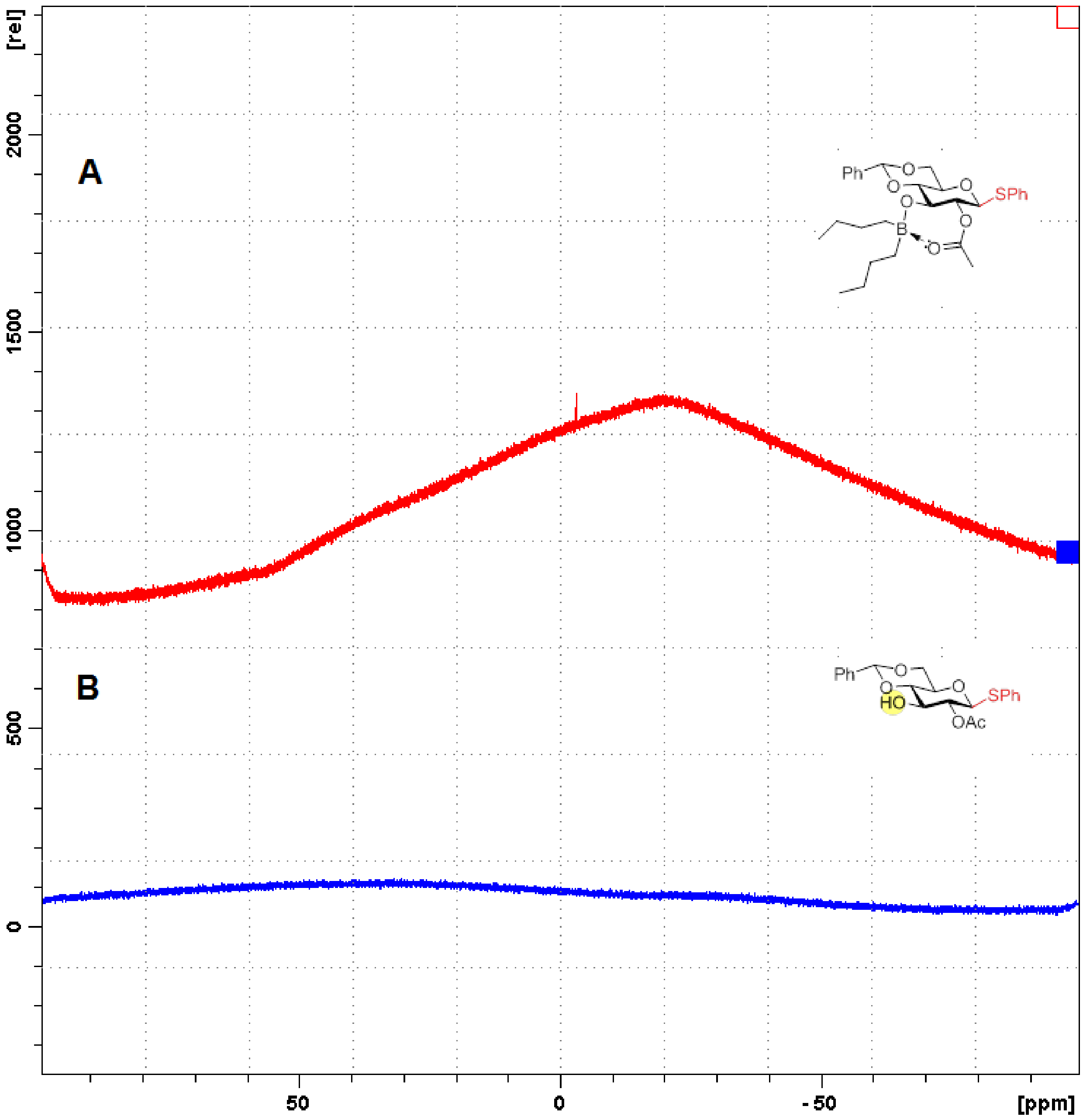

Supplementary Figure 101 ^{11}B-NMR of donor **30a** before and after addition of Bu_2BOTf. (A) after addition of Bu_2BOTf; (B) before addition of Bu_2BOTf.

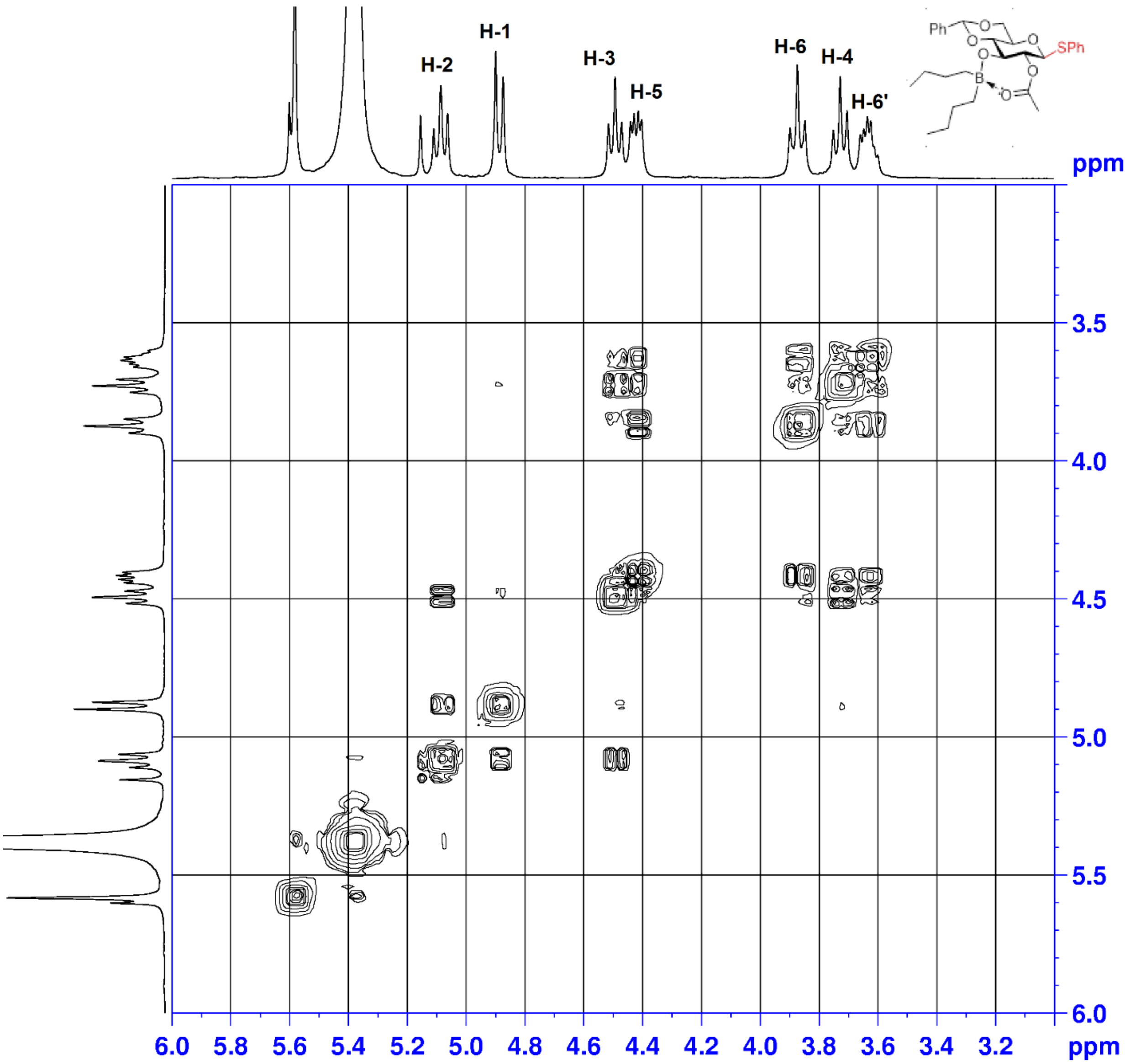

Supplementary Figure 102. 2D-COSY of donor **30a** after addition of Bu_2BOTf.
